# GRP78 Overexpression Triggers PINK1-IP_3_R-Mediated Neuroprotective Mitophagy

**DOI:** 10.3390/biomedicines9081039

**Published:** 2021-08-18

**Authors:** Tatiana Leiva-Rodríguez, David Romeo-Guitart, Mireia Herrando-Grabulosa, Pau Muñoz-Guardiola, Miriam Polo, Celia Bañuls, Valerie Petegnief, Assumpció Bosch, Jose Miguel Lizcano, Nadezda Apostolova, Joaquim Forés, Caty Casas

**Affiliations:** 1 Department of Cell Biology, Physiology and Immunology, Institut de Neurociències (INc), Universitat Autònoma de Barcelona (UAB), 08193 Barcelona, Spain; tatileivarodriguez@gmail.com (T.L.-R.); Mireia.Herrando@uab.cat (M.H.-G.); Caty.Casas@uab.es (C.C.); 2 Centro de Investigación Biomédica en Red Sobre Enfermedades Neurodegenerativas (CIBERNED), 08193 Barcelona, Spain; Assumpcio.Bosch@uab.cat; 3 Laboratory Hormonal Regulation of Brain Development and Functions—Team 8, Institut Necker Enfants-Malades (INEM), INSERM U1151, Université Paris Descartes, Sorbonne Paris Cité, 75015 Paris, France; 4 Department of Biochemistry and Molecular Biology, Institut de Neurociències (INc), Universitat Autònoma de Barcelona (UAB), 08193 Barcelona, Spain; pau.munoz@uab.cat (P.M.-G.); JoseMiguel.Lizcano@uab.cat (J.M.L.); 5 Service of Endocrinology University Hospital Doctor Peset, Foundation for the Promotion of Health and Biomedical Research in the Valencian Region (FISABIO), 46010 Valencia, Spain; miriam.polo@uv.es (M.P.); celia.banuls@uv.es (C.B.); 6 Department of Brain Ischemia and Neurodegeneration, Institute for Biomedical Research of Barcelona (IIBB), Spanish Research Council (CSIC), Institut d’Investigacions Biomèdiques August Pi Sunyer (IDIBAPS), 08036 Barcelona, Spain; valerie.petegnief@iibb.csic.es; 7 CIBERehd, Department de Farmacologia, Facultat de Medicina, Universitat de Valencia, 46010 Valencia, Spain; nadezda.apostolova@uv.es; 8 Hand and Peripheral Nerve Unit, Hospital Clínic i Provincial, Universitat de Barcelona, 08007 Barcelona, Spain; JFORES@clinic.cat

**Keywords:** GRP78/BiP, mitophagy, motoneurons, neurodegeneration, neuroprotection

## Abstract

An experimental model of spinal root avulsion (RA) is useful to study causal molecular programs that drive retrograde neurodegeneration after neuron-target disconnection. This neurodegenerative process shares common characteristics with neuronal disease-related processes such as the presence of endoplasmic reticulum (ER) stress and autophagy flux blockage. We previously found that the overexpression of GRP78 promoted motoneuronal neuroprotection after RA. After that, we aimed to unravel the underlying mechanism by carrying out a comparative unbiased proteomic analysis and pharmacological and genetic interventions. Unexpectedly, mitochondrial factors turned out to be most altered when GRP78 was overexpressed, and the abundance of engulfed mitochondria, a hallmark of mitophagy, was also observed by electronic microscopy in RA-injured motoneurons after GRP78 overexpression. In addition, GRP78 overexpression increased LC3-mitochondria tagging, promoted PINK1 translocation, mitophagy induction, and recovered mitochondrial function in ER-stressed cells. Lastly, we found that GRP78-promoted pro-survival mitophagy was mediated by PINK1 and IP3R in our in vitro model of motoneuronal death. This data indicates a novel relationship between the GRP78 chaperone and mitophagy, opening novel therapeutical options for drug design to achieve neuroprotection.

## 1. Introduction

Disruption of the functional neuronal connectivity is a common early characteristic of neurodegenerative processes [1]. Axonal degeneration isolates neurons that succumb through a retrograde and progressive dysfunctional process. In the face of damage, neurons react, activating endogenous mechanisms of neuroprotection such as the unfolded protein response (UPR), the heat-shock response, the autophagy pathway, the ubiquitin-proteasome system, chaperone expression, the endoplasmic reticulum (ER) associated degradation machinery (ERAD), and the antioxidant defense. Although their precise activation is effective in recovering the cell, excessive damage as well as aging can result in defective functioning of one or more of those programs. We reasoned that mimicking nature and boosting these endogenous mechanisms may be an efficient strategy for neuroprotection [2,3].

GRP78, also known as BiP or heat shock protein 5a (HSP5a), is a multifunctional protein with critical functions in endogenous mechanisms of neuroprotection [4]. GRP78 orchestrates the UPR, which is activated after ER stress, has ATPase activity, and is a Ca^2+^ binding protein [5,6]. It also acts to promote the proper folding of newly synthesized or misfolded proteins and to target disassembled proteins for degradation by the ERAD machinery (reviewed in [7]). Evidence also suggests that GRP78 participates in triggering macroautophagy, which removes both soluble and aggregated forms of unfolded proteins and dysfunctional organelles [8,9,10,11,12,13,14,15]. Overexpression of GRP78 has been proven to be neuroprotective in several models of neurodegeneration [16,17,18,19,20,21,22,23], and a reduction in GRP78 levels has been observed during aging and throughout the progression of degenerative disorders [24].

Some of these studies reported that neuroprotection was mediated by inhibition of apoptosis; however, apoptosis is rarely the main cause of neuronal cell death during neurodegenerative processes [20,25,26,27]. Thus, our main goal is to establish the mechanisms by which GRP78 overexpression leads to neuroprotection using non-transgenic models of neurodegeneration. In particular, exploiting the anatomical and technical advantages of several models of spinal motoneuron (MN) axotomy, we previously reported that nerve root avulsion (RA) initiates a retrograde process of motor neurodegeneration (80% of MN loss over a month post-injury) characterized by the presence of ER stress, autophagy flux blockage, vesicle, and protein trafficking arrest, the concurrence of apoptosis/antiapoptosis/anoikis initiation but the absence of an effective apoptosis execution [20,25,28]. We discovered that the expression of GRP78 is lost around 5 days after RA within damaged MNs and that its forced overexpression allows their survival after axotomy. As we described previously that a correct autophagy resolution increases neuron survival after RA [26], we wonder if it is possible to decipher if selective autophagy may have a protective effect. Hence, we used an RA model to clarify the mechanisms that mediate GRP78 neuroprotection using an unbiased proteomic analysis, and we further validated the resulting hypothesis by in vitro depth analysis using tunicamycin treatment that triggers both ER stress and protein trafficking arrest, two main characteristics of the RA model [26].

## 2. Materials and Methods

### 2.1. Surgical Procedures

Sprague–Dawley female rats aged 12 weeks were kept under standard conditions of light and temperature and given food and water ad libitum. We performed surgical procedures under anesthesia with a cocktail of ketamine/xylazine (0.1 mL/100 g weight) intraperitoneally, essentially as reported previously [26,27]. To perform extravertebral nerve root avulsion of the L4–L5 roots, we made a midline skin incision to identify each side of the sciatic nerve and applied moderate traction on selected roots away from the intervertebral foramina, obtaining the mixed spinal nerves that contained the motor and sensory roots and dorsal root ganglia. The wound was sutured by planes and disinfected with povidone iodine, and the animals were allowed to recover in a warm environment. Sham-operated animals were used as controls. All the procedures that involved animals were performed in accordance with Spanish (Real Decreto 53/2013) and European (2010/63/UE), and were approved by the Departament d’Agricultura, Ramaderia, Pesca, Alimentació i Medi Natural of the Catalan Government (Generalitat de Catalunya) legislation. 

### 2.2. Construction, Purification, and Infection with Viral Vectors

cDNA encoding GRP78 (ATCC, LGC Promochem, Teddington, UK) was cloned into the pAC.CMV shuttle vector. Recombinant adenoviruses were constructed by homologous recombination in HEK293 cells. The control adenovirus expressing bacterial β-galactosidase (Ad-β-gal) was a kind gift of C.B. Newgard (Duke University, Durham, NC, USA). Viruses were purified using the Vivapure AdenoPackTM 20 kit according to the instructions of the manufacturer (Sartorius, Göttingen, Germany). For adeno-associated vector construction, the *GRP78* cDNA was cloned into NheI and HindIII sites between the ITR domains of AAV2 under the regulation of a CMV promoter and the woodchuck hepatitis virus responsive element [29]. The AAVrh10 vector was generated as previously described [30] by triple transfection of HEK293-AAV cells (Stratagene, Bellingham, WA, USA) with branched polyethyleneimine (Sigma-Aldrich, St. Louis, MO, USA) with the plasmid containing the ITRs of AAV2, the AAV helper plasmid containing Rep2 and Cap for rh10 (kindly provided by J.M. Wilson, University of Pennsylvania, Philadelphia, PA, USA), and the pXX6 plasmid containing helper adenoviral genes [31]. Recombinant vectors were clarified after benzonase treatment (50 U/mL, Novagen, Madison, WI, USA) and polyethylene glycol (PEG 8000, Sigma-Aldrich) precipitation. Vectors were purified by iodixanol gradient by the Vector Production Unit at CBATEG-UAB following standard operating procedures. Viral genomes per ml (vg/mL) were quantified using PicoGreen (Invitrogen, Waltham, MA, USA).

Immediately after RA, the animals were injected with 14 µL of either Ad-GRP78 or Ad-β-gal virus (10^8^ pfu/mL) using a 33-gauge needle and a Hamilton syringe into the thecal space at the lumbar site. Alternatively, intrathecal administration of 10 µL of 4 × 10^−10^ viral genomes of AAVrh10 adeno-associated virus to overexpress GFP or GRP78 was slowly injected into the CSF between vertebrae L3 and L4 of isoflurane-anesthetized animals three weeks before RA [32]. We used AAVrh10 because it infects specifically 30% of spinal motoneurons [32]. Appropriate access to the intrathecal space was confirmed by animal tail flick. Needles were held in place at the injection site for 1 min, after which muscle and skin were sutured.

### 2.3. Sample Preparation and Proteomic Analysis

We anesthetized rats (*n* = 4–5) at 7 dpi and obtained L4–L5 spinal cord segments (5-mm length) samples, which were snap-frozen in liquid nitrogen. We homogenized the tissue in lysis buffer (20 mM HEPES, pH 7.7, 250 mM sucrose, 1 mM EDTA, 1 mM EGTA, and a cocktail of protease and phosphatase inhibitors) with a Potter homogenizer on ice. After centrifugation of lysates at 800× *g* for 20 min at 4 °C, we collected the supernatant as the post-nuclear fraction and quantified protein by BCA assay (Pierce Chemical Co., Dallas, TX, USA). For proteomic analysis, we solubilized 75 μg of each sample in 4% SDS, 8 M urea, 0.1 M HEPES, pH 7.7, 0.1 M DTT, added 0.05 M iodoacetamide, and digested with trypsin (1:10 ratio of enzyme:substrate) using the Filter Aided Sample Preparation (FASP) method. All samples were treated in parallel.

We analyzed samples using an LTQ-OrbitrapVelos mass spectrometer (Thermo Fisher Scientific, Waltham, MA, USA) coupled to a ProxeonEasyLC (Thermo Fisher Scientific). We loaded the peptide mixtures directly onto the analytical column (2 μL·min^−1^) and separated peptides by reversed-phase chromatography using a 15-cm column with an inner diameter of 100 μm packed with 5-μm C18 particles (NikkyoTechnos Co., Tokyo, Japan). Chromatographic gradients started at 97% buffer A (0.1% formic acid (FA) in water), and 3% buffer B (acetonitrile, 0.1% FA) with a flow rate of 500 nl·min^−1^, and increased to 85% buffer A + 15% buffer B over 4 min, and to 55% buffer A + 45% buffer B over 120 min. We operated the instrument in TOP20 Data Dependent Acquisition mode with one full MS scan in the Orbitrap at a resolution of 60,000 and a mass range of *m*/*z* 350–2000 followed by MS-MS spectra of the 20 most intense ions. We utilized the ion trap with a collision-induced dissociation method to produce fragment ion spectra and used normalized collision energy at 35%. We acquired all data with Xcalibur software v2.1. We used the Proteome Discoverer software suite (v1.3.0.339, Thermo Fisher Scientific) and the Mascot search engine (v2.3.01, Matrix Science50) for peptide identification and quantitation. We analyzed the data against SwissProt Rat database containing the most common contaminants (599 entries). We used a precursor ion mass tolerance of 7 ppm at the MS1 level and allowed up to three miscleavages for trypsin. The fragment ion mass tolerance was set to 0.5 Da. Oxidation of methionine and N-terminal protein acetylation were set as variable modifications, and cysteine carbamidomethylation was set as a fixed modification. We filtered the peptides based on their false discovery rate (FDR > 5% not considered). For peptide quantification, we considered the chromatographic peak of the peptides calculated by using the Proteome Discoverer and median normalized the areas by log2 transformation using R 3.0.2. We quantified the data using the R package MSstats (v. 2.0.1). For each ratio, we calculated the adjusted *p*-value (*p* < 0.05 for significance). Finally, we performed Gene Ontology and pathway analysis for regulated proteins with DAVID Annotation Web tools (https://david.ncifcrf.gov/ accessed on 28 May 2021) and KEGG (https://www.genome.jp/kegg/pathway.html, accessed on 28 May 2021), as we performed previously (see more details in [25])**.**

### 2.4. Immunohistochemistry and Image Analysis

After deep anesthesia with pentobarbital, we transcardially perfused the animals with a saline solution containing 10 U/mL heparin, followed by 4% paraformaldehyde in a 0.1 M phosphate buffer, pH 7.2 for tissue fixation at 7 dpi (n = 4 for each condition), and removed the L4 and L5 segments (5-mm total length) of the spinal cord, which were post-fixed in the same fixative for 4 h and cryopreserved in 30% sucrose overnight. Serial transverse sections (20-µm thick) were obtained on gelatinized slides using a cryotome (Leica, Wetzlar, Germany) and stored at −20 °C until analysis. For immunohistochemistry, we treated the slides with blocking solution in Tris-buffered saline (TBS) with 0.03% Triton-X-100 and 10% bovine serum for 1 h and incubated thereafter with different primary antibodies: rabbit anti- LC3B (ab51520; Abcam; 1:200) or mouse anti-COXIV (A21355; Life Technologies;1:500). After several washes with TBS, 0.05% Tween-20, the sections were incubated for 2 h with Cy-2 or Cy-3 conjugated donkey anti-rabbit antibodies (Jackson Immunoresearch, West Grove, PA, USA). We counterstained the sections with DAPI (Sigma, St Louis, MO, USA), or NeuroTrace Fluorescent Nissl Stain (Molecular Probes, Leiden, The Netherlands) and mounted the slices with Fluoromount-G mounting medium (Southern Biotech, Birmingham, AL, USA) or hand-made Mowiol. Sections to be compared were processed together on the same slide and on the same day. Images of the spinal cord samples from different treatments and controls were taken under the same exposure time, sensitivity, and resolution for each marker analyzed with the aid of a confocal microscope (Zeiss LSM 700). We analyzed signal intensity with ImageJ software (National Institutes of Health; available at http://rsb.info.nih.gov/ij/, accessed on 28 May 2021).

### 2.5. Western Blot

We deeply anesthetized rats with Dolethal (*n* = 4) at 7 dpi to obtain L4–L5 spinal cord segments (5-mm length) for western blot analysis. We snap-froze the samples in liquid nitrogen. Samples were stored or further processed by homogenization in lysis buffer (20 mM HEPES, pH 7.7, 251 mM sucrose, 1 mM EDTA, 1 mM EGTA, and a cocktail of protease (Sigma-Aldrich) and phosphatase inhibitors (Roche, Basel, Switzerland)) with a Potter homogenizer on ice. After centrifugation of lysates at 800× *g* for 20 min at 4 °C, we collected the supernatant as the post-nuclear fraction, and quantified proteins by BCA assay (Pierce Chemical Co.). For western blotting, we loaded 30 µg of post-nuclear fractions of L4–L5 segments from each animal onto 12% SDS-polyacrylamide gels to perform electrophoretic separation of the proteins followed by transfer to a PVDF membrane in a BioRad cuvette system in 25 mM Tris, pH 8.4, 192 mM glycine, 20% (*v*/*v*) methanol. We blocked the membranes with 5% BSA in PBS plus 0.1% Tween-20 for 1 h at room temperature and then incubated at 4 °C overnight with primary antibody: mouse anti-β-actin (1:10000; Sigma-Aldrich), mouse anti-GRP78 (1:500, Sigma-Aldrich), mouse anti-CVβ (1:1000, Invitrogen), rabbit anti-HSP60 (1:500, Antibodies-online), mouse anti-NDUFA9 (1:1000, Invitrogen), mouse anti-OPA1 (1:1000, BD Biosciences), rabbit anti-Parkin (1:500, Abcam, Cambridge, UK), mouse anti-PINK1 (1:500, Abcam), rabbit anti-NRF2 (1:500, Abcam), anti-MNF2 (1:500, Abcam), rabbit anti-GRP75 (1:500, Abcam)**.** After several washes, membranes were incubated for 2 h with an appropriate secondary antibody conjugated with horseradish peroxidase (1:5000, Vector). The membrane was visualized using a chemiluminescent mix of 1:1 (0.5 M luminol, 79.2 mM p-coumaric acid, 1 M Tris-HCl, pH 8.5) and (8.8 M hydrogen peroxide, 1 M Tris-HCl; pH 8.5), and the images were taken with a Gene Genome apparatus and analyzed with Gene Snap and Gene Tools software (Syngene).

### 2.6. In Vitro Model

NSC34 cells were cultured in modified Eagle’s medium high-glucose (DMEM, Biochrom, Cambridge, UK) supplemented with 10% fetal bovine serum (Sigma-Aldrich), 100 units/mL penicillin, and 0.5 X penicillin/streptomycin solution (Sigma-Aldrich) and maintained in a humidified incubator at 37 °C under 5% CO_2_, essentially as described previously [26]. After 4 days of cell culture without changing the medium, NSC34 cells had a differentiated-like phenotype characterized by the presence of long neurites. On day 4, drugs were added to the cells. Tun (Sigma-Aldrich, Missouri, USA) and EFV (Bristol-Myers Squibb, New York, USA) solutions were prepared at a concentration of 10X and dissolved in DMEM. After 24 h, we assessed cell viability by incubating the cells with 4 mg/mL MTT solution for 3 h. The formed blue formazan crystals were dissolved in DMSO, and absorbance at 570 nm was measured with a microplate reader (Elx800, Bio-tek, Winooski, VT, USA).

### 2.7. Nucleofection

We transfected a million cells with 2 µg plasmid for expression of GFP, GRP78, HA-PARK, mouse sh-eGFP, mouse sh-PINK1 (ID 68943, Tebu-bio), mouse sh-Hspa5 (ID 14828, Tebu-bio), and/or mouse sh-IP_3_R (ID 16440, Tebu-bio) using the Amaxa Nucleofector II TM (Lonza, Basel, Switzerland) and the Nucleofactor V kit (Lonza) following manufacturer’s recommendations. For static cytometric analysis, we seeded the cells in 48-well plates coated with collagen 10%. After culture, we fixed the cells with 4% PFA, rinsed twice with PBS, and stored or subsequently added blocking buffer containing PBS 0.3% (*v*/*v*) Triton X-100 and 10% of fetal bovine serum. After 3 days of cell culture, drugs dissolved in DMEM were added. For these experiments, we used 1 µM Tun (Sigma-Aldrich), 10 µM CCCP (Sigma-Aldrich), 5 µM CsA (Sigma-Aldrich), or 10 µM BAPTA-AM (Sigma-Aldrich). Following 5 h after the treatment, we either fixed the cells for immunocytochemistry with 4% PFA or homogenized them in modified RIPA buffer (50 mM Tris-HCl, pH 7.5, 150 mM NaCl, 1 mM EGTA, 1% NP-40, 0.5% sodium deoxycholate, 0.1% SDS, protease and phosphatase cocktails) for immunoblotting. Immunohistochemistry was performed by rinsing the samples twice with PBS and incubating with blocking buffer containing PBS plus 0.3% (*v*/*v*) Triton X-100 and 10% fetal bovine serum. We incubated with the following primary antibodies: rabbit anti-IP3R (1:200, Abcam), mouse anti-HA (1:2000, Abcam), mouse anti-GRP78 (1:200, Sigma-Aldrich), and rabbit anti-HSP60 (1:500, Antibodies-online). To assess viability by MTT as described above, we left the treatment for 24 h.

### 2.8. Measurement of Mitochondrial Superoxide Production and ΔψM

Cells were incubated with 2.5 µM MitoSOX for superoxide analysis or with 2.5 µM TMRM for ΔψM analysis, both from Molecular Probes, and Invitrogen during the last 30 min of treatment. Fluorescence was detected with an IX81 Olympus microscope and quantified using the static cytometry software.

### 2.9. Electrochemical Measurement of Oxygen Consumption

Cells (1 × 10^6^ per 1 mL in HBSS) were agitated in a gas-tight chamber at 37 °C. Measurements were taken with a Clark-type O_2_ electrode (Rank Brothers, Cambridge, UK) and recorded with the Dup.18 data acquisition device (WPI) immediately after 5-h treatment with vehicle or Tun.

### 2.10. Mitochondrial and Cytosolic Fractionation

Cells were homogenized in lysis buffer (10 mM HEPES, pH 7.4, 1 mM EGTA, 250 mM sucrose, and a cocktail of proteases (Sigma-Aldrich) and phosphatase inhibitors (Roche)) with a Potter homogenizer on ice. After centrifugation of lysates at 2000× *g* for 5 min, the supernatant was centrifuged at 10,000× *g* for 12 min. The supernatant was considered the soluble fraction (S2) and contains cytosolic proteins and microsomes. The pellet (P2) was the enriched mitochondrial fraction, which was resuspended in modified RIPA buffer (50 mM Tris-HCl, pH 7.5, 150 mM NaCl, 1 mM EGTA, 1% NP-40, 0.5% sodium deoxycholate, 0.1% SDS, protease and phosphatase cocktails) and protein concentration was determined by BCA assay. Samples were then processed for western blot.

### 2.11. Transmission Electron Microscopy

We submerged spinal cord L4-L5 segments (1-mm slices) in a fixative solution of 2% (*w*/*v*) PFA and 2.5% (*v*/*v*) glutaraldehyde (EM grade) in 0.1 M PBS, pH 7.4 and placed them on a rocking platform for 2 h, then fixed in 1% (*w*/*v*) PFA and subsequently post-fixed with 1% (*w*/*v*) osmium tetroxide (TAAB Lab) containing 0.08% (*w*/*v*) potassium hexacyanoferrate (Sigma-Aldrich) in PBS for 2 h. We performed four washes with deionized water and sequential dehydration in acetone. All procedures were performed at 4 °C, as previously described [33]. We embedded samples in EPON resin and polymerized at 60 °C for 48 h. Samples were processed to obtain semi-thin sections (1 µm) with a Leica ultracut UCT microtome, stained with 1% (*w*/*v*) aqueous toluidine blue solution, and examined with a light microscope to identify the ventral horn areas enriched with MNs. Ultrathin sections (70 nm) were cut with a diamond knife, placed on coated grids, and contrasted with conventional uranyl acetate and Reynolds’ lead citrate solutions. Finally, we observed the sections with a transmission electron microscope (Jeol 1400) equipped with a GatanUltrascan ES1000CCD Camera. We selected three slices per condition, analyzed three MNs per section, and measured the areas of nuclei compared to the total area of the MN using ImageJ tools.

### 2.12. Bioinformatics and Statistics

We used analyses of variance (ANOVA) to compare the values among different experimental groups for data that met the normality assumption. Differences between groups were analyzed using a one-way ANOVA, followed by Tukey’s post hoc multiple-range test, or using Student’s *t*-test. All statistical analyses were done using GraphPad Prism 5 software (*n* = 3–5; *p* < 0.05 for significance).

## 3. Results

### 3.1. Text

#### 3.1.1. Proteomic Analysis of GRP78 Overexpression Revealed Mitochondria as the Main Target

In order to identify the molecular mechanisms leading to neuroprotection mediated by GRP78 overexpression, we performed a comparative label-free proteomic analysis to identify both quantitative and qualitative differences between RA-injured animals infected with adenoviruses for overexpression of GRP78 (Ad-GRP78), previously found to be neuroprotective of MNs, or beta-galactosidase (Ad-ß-Gal) as control [20]. Seven days post-injury (dpi) was the chosen time-point for the analysis according to previous studies carried out in our laboratory [20,25]. In a previous comparison between a model of regeneration after distal axotomy and suture of sciatic nerve versus our degenerative RA model, we observed that signaling events were similar until 7 dpi, when divergent pro-degenerative events emerged after RA [20,25]. The LC-MS/MS label-free analysis of the post-nuclear fractions from L4-L5 spinal cord segments resulted in the identification of 1420 proteins, with at least two peptides per protein as previously reported for other samples and studies [25]. A total of 566 and 732 proteins or peptides were significantly altered due to Ad-GRP78 or Ad-ß-Gal overexpression, respectively, in RA-injured animals with respect to RA-injured animals not infected with a virus (*p* < 0.05) (Tables S1 and S2). We compared both lists and found that 220 were uniquely altered due to Ad-GRP78 overexpression (Table S3).

**Figure 1:**
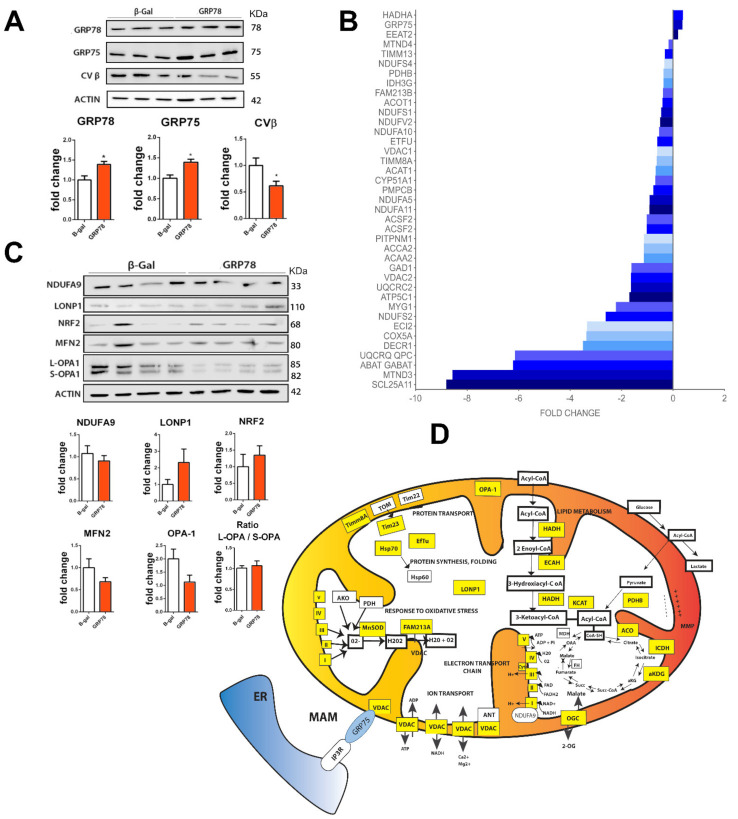
GRP78 overexpression in RA-injured animals targets mitochondrial proteins. (**A**) Immunoblot and bar graphs showing average fold changes (± SEM) of GRP78, GRP75, and CVβ protein levels in L4-L5 spinal cord segments in GRP78 vs. β-Gal overexpressing RA-injured rats by 7 dpi. Levels were normalized to actin values (*n* = 4; * *p* < 0.05 vs. Ad-β-Gal, Student’s *t*-test). (**B**) Histogram of mitochondrial protein fold changes obtained by proteomic analysis of L4-L5 spinal cord segments from in RA-injured animals that overexpress GRP78 compared to the Ad-β-Gal group by 7 dpi. (**C**) Immunoblot and bar graphs showing average fold changes (± SEM) of NDUFA9, LONP1, NRF2, MFN2, OPA-1 normalized to actin. (No significant differences between groups. Student’s *t*-test). (**D**) Schematic mitochondria with proteins altered by overexpression of GRP78 in L4-L5 spinal cord segments from RA-injured animals highlighted in yellow (decreased) or in blue (increased) at 7dpi.

Functional annotation of these proteins using the Database for Annotation, Visualization, and Integrated Discovery (DAVID) tool allowed us to identify the most significant biological functions in the data set (FDR < 0.05) [34,35]. GRP78 overexpression resulted in differences in the levels of proteins involved in mitochondrial processes, function, and components (Table S4A–C, Figure S1A–C). Gene Ontology (GO) terms and KEGG pathway enrichment analysis of these data revealed that in animals infected with Ad-GRP78 there were significant decreases in the levels of many proteins with mitochondrial functions, including proteins from the outer and inner mitochondrial face, proteins of the respiratory complex, and matrix proteins. We confirmed differences in protein levels revealed in the proteomic list, such as GRP75, which was upregulated (*p* < 0.05), and the β subunit of complex V (CVβ), downregulated (*p* = 0.03) in the GRP78 groups as expected (Figure 1A). Across the whole list, the most highly downregulated were the 2-oxoglutarate/malate carrier protein OGC (SCL25A11), MT-ND3 (a tumor suppressor that participates in the malate-aspartate shuttle and regenerates the NADH pool in the mitochondrial matrix to allow complex I function), the NADH dehydrogenase subunit 3 of complex I, the gamma-aminobutyric acid aminotransferase ABAT, and the ubiquinol-cytochrome C reductase subunit of complex III. In contrast, very few proteins were upregulated (Figure 1B). One upregulated protein was GRP78 as expected, and the other upregulated proteins were the amino acid transporter EAAT2, which is primarily located in the plasma membrane and sometimes in mitochondria at excitatory synapses, and the hydroxyacyl-CoA dehydrogenase HADHA, which catalyzes the last three steps of mitochondrial beta-oxidation of long-chain fatty acids.

We extended the analysis to investigate whether other proteins in mitochondria or mitochondria function-related proteins were modified out of the proteomic list in order to confirm functional mitochondria affectation (Figure 1C). We observed that the mitochondrial conserved dynamin-related GTPase optic atrophy 1 (OPA1), which is involved in fusion [36], was downregulated (*p* < 0.1); LONP1, an ATP-dependent protease that mediates the selective degradation of misfolded polypeptides, presented a tendency to be increased, but no differences were observed in the levels of ubiquinone NDUFA9, the transcription factor NRF2, which regulates genes that contain antioxidant response elements, or other dynamin-related GTPase involved in infusion, such as mitofusin 2 (MFN2) [36]. A comprehensive summary of the alterations found within mitochondria is shown in the scheme in Figure 1D.

**Figure 2:**
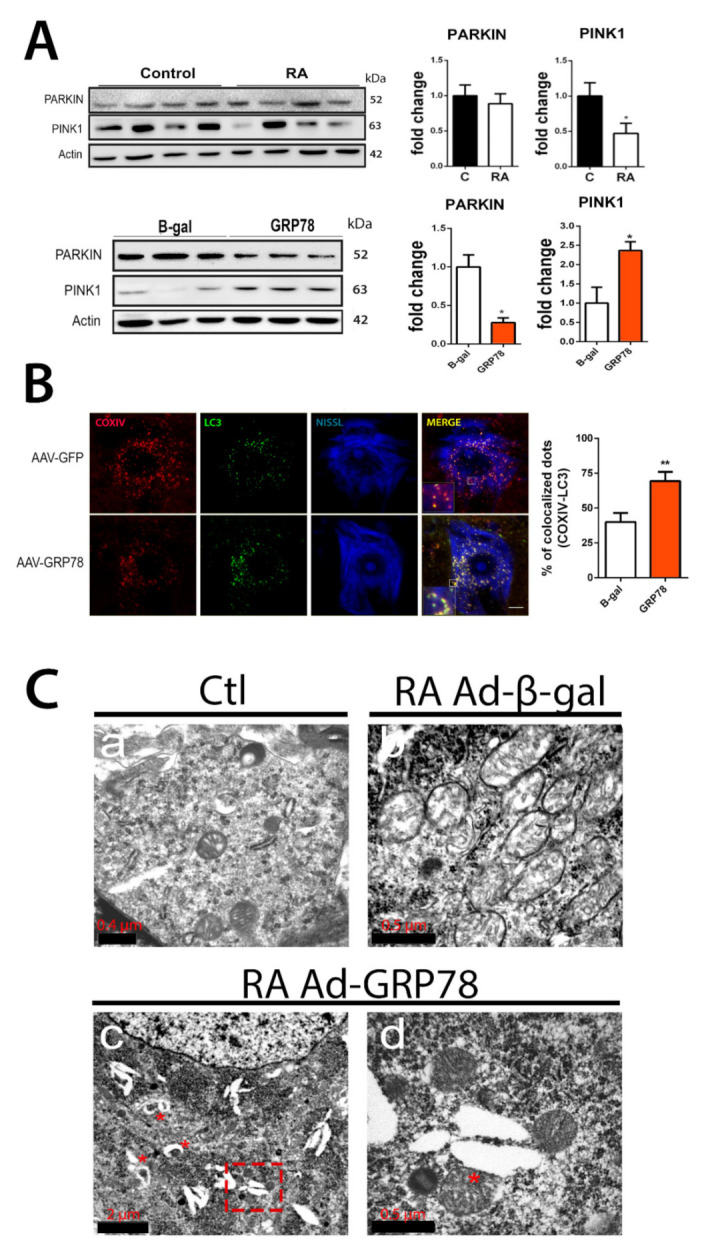
Mitophagy is modulated in vivo after RA in rats that overexpress GRP78. (**A**) Immunoblots and corresponding bar graphs for PINK1 and PARK fold change analysis of protein levels (means ± SEM) normalized to actin levels, in the L4-L5 spinal cord segments of RA-injured rats vs. control (top) or from Ad-β-Gal and Ad-GRP78 groups (bottom) by 7 dpi. (*n* = 4; * *p* < 0.05, Student’s *t*-test). (**B**) Left, microphotographs of representative MNs at the ipsilateral site from RA-injured animals that received an intrathecal injection of AAVrh10-GFP or AAVrh10-GRP78 groups at 7 dpi. Samples were stained for COX IV (red) and LC3 (green) and counterstained with fluorescent Nissl (blue). Scale bar = 10 µm and digital zoom in insets 10X. Note that co-localization is only observed in the AAVrh10-GRP78 group. Right, bar graph showing the analysis of % of co-localization dots of COX IV and LC3. Average ± SEM of COX IV and LC3 co-localization (*n* = 4; ** *p* < 0.01, Student’s *t*-test). (**C**) Representative transmission electron microscopy images where mitochondria are distinguished within MNs of spinal cords: a, image of MN cytoplasm showing mitochondria in control animal; b, image of MN cytoplasm from RA-injured rat from the Ad-β-gal group; c, image of MN cytoplasm from RA-injured rat from the Ad-GRP78 group with some engulfed mitochondria indicated with red asterisks; d, higher magnification image is shown in (**C**) pointing out a large mitochondria and ER contact. Scale bars are indicated in the figures.

Mitochondrial dynamics are important for stress responses as damaged mitochondria can fuse for the exchange of material, and mitochondrial fission allows the segregation of damaged mitochondria and subsequent mitophagy [37]. Considering that the major number of downregulated proteins localize at the mitochondria and no evident changes were observed regarding mitochondria fusion activity by analyzing MFN2 and the ratio of L/S OPA1 forms (Figure 1C), we investigated markers related to mitophagy. Classically, autophagy-dependent degradation of mitochondria occurs following depolarization, and it is usually triggered by the increased accumulation of the phosphatase and tensin homolog (PTEN)-induced putative kinase 1 (PINK1) full length at the outer mitochondrial membrane. Subsequently, the in-between-ring E3 ubiquitin-protein ligase Parkin is recruited to mark mitochondrial outer membrane protein of depolarized mitochondria by ubiquitylation [38,39,40,41]. The top of Figure 2A shows no changes in Parkin levels but a significant reduction of full-length PINK1 produced by RA-injury in the ipsilateral L4-L5 spinal cord segment with respect to the control at 7 dpi. In contrast, the overexpression of GRP78 reduced Parkin but doubled PINK1 levels after RA compared to the control expression of non-related protein β-Gal (Figure 2A, bottom). The upregulation of full-length PINK1 is consistent with the induction of mitophagy [42], and Parkin reduction might be related to an advanced state of organelle dismantle as observed by the rest of mitochondria proteins. We further investigate co-localization of mitochondria and the microtubule-associated proteins 1A/1B light chain 3 (LC3)-positive puncta, a key component of the core macroautophagy machinery and necessary for the mitophagy process [43]. Using confocal microscopy, we observed a significant increase in the co-localization of mitochondria, labeled using anti-COX IV, with LC3-positive puncta after RA only in damaged MNs overexpressing GRP78 at 7 dpi (Figure 2B). We confirmed the existence of mitophagy in avulsed MNs only when overexpressing GRP78 by the analysis of samples using transmission electronic microscopy. We observed abundant vacuoles with engulfed mitochondria, a hallmark of mitophagy [37], resembling mitophagosomes, and several ER cisternae in close connection to mitochondria within MNs from Ad-GRP78 animals, but we did not detect them in Ad-β-Gal RA-injured rat samples at 5 dpi (Figure 2C).

Altogether, these results suggested that GRP78 overexpression may induce mitophagy in disconnected MNs after RA, and it could be promoting neuroprotection.

#### 3.1.2. GRP78 Overexpression Restores Damaged Mitochondrial Function

**Figure 3:**
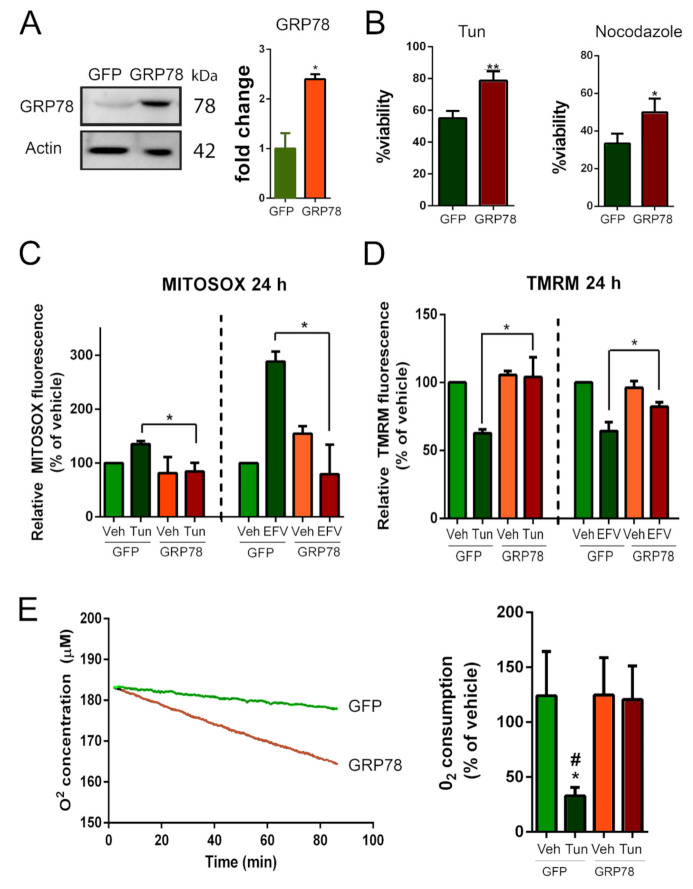
GRP78 overexpression rescues mitochondrial dysfunction in stressed NSC34 cells. (**A**) Immunoblot and bar graph showing the levels of GRP78 in NSC34 cells nucleofected with plasmid vector to overexpress GRP78 or a non-related protein as control (GFP). (**B**) Percentage of survival of GFP- or GRP78-expressing NSC34 cells (means ± SEM) after 24 h in medium containing 1 µg/mL tunicamycin (Tun) (left) or 10 µM nocodazole (right) determined using an MTT assay (*n* = 4; * *p* < 0.005, ** *p* < 0.001 vs. GFP, Student’s *t*-test). (**C**) Quantitative analysis of MitoSOX fluorescence in cells overexpressing GFP or GRP78 and treated with Tun or vehicle (left) or efavirenz (EFV) as control or vehicle (right). (**D**) Quantitative analysis of TMRM fluorescence in cells overexpressing GFP or GRP78 and treated with Tun or vehicle (left) or EFV or vehicle (right) (GFP-Veh is the control group). (**E**) Left: Representative analysis of O2 concentration (using a Clark-type O2 electrode) as a function of time in cells overexpressing GFP or GRP78 and treated with Tun. Right: O2 consumptions after 5 h of Tun treatment in cells overexpressing GFP or GRP78 (GFP-Veh is the control group) (*n* = 4; * *p* < 0.05 vs. Veh-GFP, # *p* < 0.05 vs. GRP78, one-way ANOVA).

We then further investigated the effect of GRP78 overexpression on mitochondria using an in vitro model of ER stress since this insult appears early after RA injury in vivo as reported previously [20,26], and it is a reliable method to screen novel neuroprotective agents [2,44]. This model employs tunicamycin (Tun), which is an ER-stress inducer that causes the formation of N-acetylglucosamine-lipid intermediates, thereby preventing the glycosylation of newly synthesized proteins and leading to proteins misfolding. We used that because a halted secretory pathway has also been reported to be key in the neurodegenerative process after RA [26]. We first verified that nucleofection of the NSC34 motoneuron-like cells with an appropriate vector resulted in the production of GRP78 (Figure 3A). In these cells, the expression of GRP78 significantly enhanced survival in medium containing Tun (Figure 3B). Although Tun treatment normally induces GRP78 itself, this endogenous overexpression is quite late (by 24 h) (Figure S2). We also wondered if we could determine if GRP78 overexpression has neuroprotective effects in another in vitro model that we previously reported to reproduce several characteristics of MN death after RA injury [26]. This model is based on cytoskeletal damage induced by nocodazole, which interferes with the polymerization of microtubules. GRP78 expression increased the viability of cells cultured in the presence of nocodazole as well (Figure 3B).

Then, we assessed mitochondrial function by measuring the generation of reactive oxygen species (ROS) in mitochondria using MitoSOX, the mitochondrial membrane potential (Δψ_m_) using tetramethylrhodamine methyl ester (TMRM), and the O_2_ consumption rate, in our NSC34 cells subjected to ER stress with or without GRP78 overexpression. As a positive control, we used efavirenz (EFV), a mitotoxic agent that alters all three parameters [45]. We observed that MitoSOX levels progressively increased after Tun addition, reaching statistical significance by 24 h and that ROS generation was increased significantly in EFV-treated cells from the first-hour post-treatment with respect to vehicle-treated controls (Figure S3A). Overexpression of GRP78 resulted in significantly lower levels of ROS in mitochondria with respect to GFP-expressing cells at 24 h after Tun or EFV treatment (Figure 3C). The Δψ_m_ was collapsed after 5 and 24 h of Tun treatment and after 24 h of EFV treatment (Figure S3B). Recovery of this parameter was achieved by GRP78 overexpression in Tun- or EFV-treated cells (Figure 3D). The rate of O_2_ consumption was diminished during 80 min of analysis after Tun addition in the cells that express GFP (slope −0.063, R^2^ = 0.98), but O_2_ consumption was stable in GRP78-overexpressing cells despite the presence of Tun (slope −0.221, R^2^ = 0.99) (Figure 3E, left). After 5 h, respiration was normal in GRP78-overexpressing cells and severely compromised in the GFP-expressing cells (Figure 3E, right). Altogether, these results suggest that GRP78 attenuates mitochondria dysfunction caused by ER stress or mitotoxicity.

#### 3.1.3. Mitophagy Induction by GRP78 Overexpression Mediates Neuroprotection

**Figure 4:**
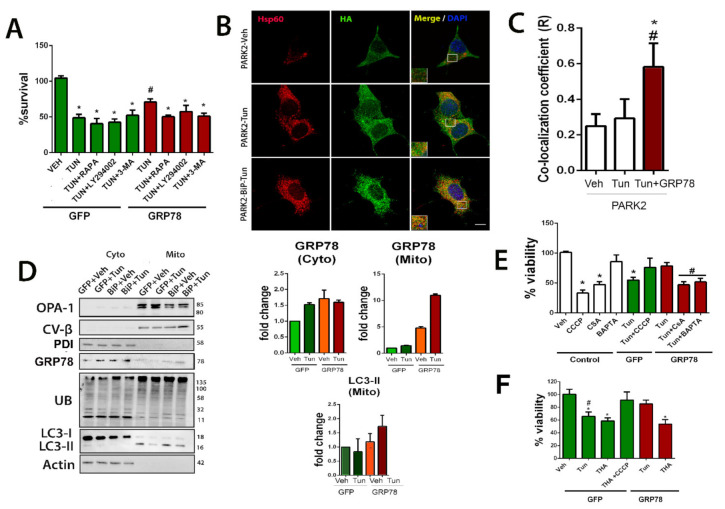
Mitochondria tagging for mitophagy is privileged by GRP78 overexpression. (**A**) Bar graph of the percentage of viable NSC34 cells (mean ± SEM) nucleofected with either GFP or GRP78 plasmids and treated with Tun or combination of Tun with autophagy modulators: rapamycin (RAPA), 3MA, or LY-902, with respect to control vehicle (Veh), determined by MTT assay 24 h after treatment (GFP-Veh is the control group) (*n* = 4–8, * *p* < 0.05 vs. Veh-GFP; # *p* < 0.05 vs. Tun-GFP). (**B**) Representative confocal images of cells nucleofected with PARK2-HA plasmid alone (top, middle panels, control) or with GRP78 plasmid (bottom panels) treated with either vehicle (Veh) or Tun (5 h) and immunostained for HA (green) and HSP60 (red); scale bar = 10 µm. (**C**) Average ± SEM of Pearson’s correlation coefficient of Parkin and Hsp60 co-localization from Veh, Tun and Tun+GRP78 groups (*n* = 4; * *p* < 0.05 vs. Parkin-Veh, # *p* < 0.05 vs. Parkin-Tun, one-way ANOVA). (**D**) **Left**, Western blots for indicated proteins in the mitochondrial (mito) and cytosolic (cyto) pooled fractions from GFP- or GRP78-overexpressing cells treated with vehicle or Tun for 5 h. The proteins analyzed are: OPA-1, CV-β, PDI, GRP78, Ubiquinated residues, LC3 and actin. **Right**, bar graphs of the average fold change of GRP78 in both pooled fractions and LC3-II in the mitochondrial fraction relative to actin (cytosol) or the beta subunit of the complex V(CV-B) (mitochondria) in the GFP group. (**E**) Bar graph of the percentage of viable cells (means ± SEM) overexpressing GFP or GRP78 proteins and treated with Tun alone or in combination with CCCP, CSA, or BAPTA-AM, determined by MTT assay 24 h after treatment with respect to control vehicle-treated cells. (**F**) Similar bar graph of cell viability for cells treated with thapsigargin (THA) with or without CCCP (GFP-Veh is the control group) (*n* = 4–8, in 3 different experiments, * *p* < 0.05 vs. control-Veh; # *p* < 0.05 vs. GRP78-Tun).

We hypothesized that GRP78-mediated neuroprotection may be through inducing selective autophagy of mitochondria to accelerate the removal of defective organelles. In support of this hypothesis, GRP78 has been proven to promote macroautophagy [4] (hereafter referred to as autophagy). We sought to determine whether autophagy was necessary for GRP78-induced neuroprotection using several well-characterized autophagy modulators: rapamycin, which activates autophagy by inhibiting mTORC1, and two inhibitors of the autophagy flux: 3-methyladenine (3-MA), an inhibitor of class III phosphatidylinositol kinase PI3K, and LY294002, an inhibitor of class I PI3K. All three autophagy modulators decreased survival in both GFP and GRP78 groups in Tun-treated cells compared to vehicle (Figure 4A). These results suggested that the correct initiation of autophagy, through the PI3K-Beclin1 pathway, and late flux was necessary, although not sufficient, for the neuroprotective effects of GRP78 overexpression in ER-stressed cells.

Parkin does not always participate in mitophagy since it can also be triggered through alternative mechanisms [46]. To ascertain whether Parkin is involved in the GRP78-mediated effect, we determined the subcellular location of HA-tagged Parkin expressed in NSC34 cells with and without ER stress. By confocal microscopy, we observed that Parkin was distributed throughout the NSC34 cell after 5 h of vehicle or Tun treatment (Figure 4B). In contrast, in cells overexpressing GRP78, there was an increase of the co-localization of Parkin with HSP60, a mitochondrial protein, suggesting targeting of this organelle for mitophagy (Figure 4B,C).

In addition, we isolated the mitochondrial and cytosolic fractions from cells that overexpressed either GFP or GRP78 and were treated with Tun or vehicle as control at 5 h after the insult. The purity of pooled mitochondrial fractions was confirmed by analyzing the presence of OPA1 and CVβ, and the absence of an ER-resident protein, the protein disulfide isomerase (PDI) (Figure 4D). Interestingly, we also observed a tendency to increase GRP78 abundance in the pooled mitochondrial fractions of ER-stressed cells compared to control. Note that although Tun stimulus might also induce an increase in GRP78 levels in cytosol, this was not seen at the mitochondrial fraction. Forced overexpression of GRP78 promoted a tendency to increase both PINK1 and Parkin accumulation in the mitochondrial fraction (Figure 4D and Figure S4). It is reported that the presence of Parkin amplifies the accumulation of Ub and enhances mitophagy compared to the presence of PINK alone [42,47]. Accordingly, Ubiquitin (Ub) immunoblotting revealed differences in its profile in mitochondrial fractions from GRP78-expressing cells compared to the GFP group (Figure 4D). Under stressful conditions, mitochondrial protein ubiquitylation leads to the recruitment of autophagosome machinery components that begins with the accumulation of the lipidated isoform of LC3, LC3II [42,48]. Abundant LC3II protein was observed in the mitochondrial fraction with a tendency to increase higher presence in the stressed cells that overexpress GRP78 with respect to those with GFP at similar conditions (Figure 4D). Taken together, these data suggested that the forced expression of GRP78 could facilitate its mitochondrial translocation and tagging for mitophagy.

To determine the possible relevance of mitophagy induction to the neuroprotective effect mediated by GRP78 overexpression, we assessed the alterations induced by activators and inhibitors of mitophagy. We used carbonyl cyanide m-chlorophenyl hydrazine (CCCP) to chemically uncouple oxidative phosphorylation and induce mitophagy by collapsing the mitochondrial membrane potential [49,50], as well as two mitophagy inhibitors: BAPTA-AM, a cell-permeant chelator of intracellular Ca^2+^ and cyclosporin A (CSA), the mitochondrial permeability transition pore (mPTP) inhibitor that blocks Ca^2+^ efflux from mitochondria [51]. Curiously, although CCCP treatment in NSC34 was sufficient to reduce notably viability, as widely reported, we found that the same treatment on ER-stressed cells increased its survival, independently of whether or not GRP78 was overexpressed (Figure 4E). Importantly, the neuroprotective effect promoted by GRP78 overexpression on ER stressed cells was abolished in the presence of either CSA or BAPTA-AM (Figure 4E). These findings indicate that mitophagy induction allows cells to cope with ER stress and Ca^2+^-flux is important for the neuroprotective effect promoted by GRP78.

One possibility for GRP78 to induce mitophagy might be facilitating the necessary Ca^2+^-mediated action, perhaps through the action of the ER-resident inositol 1,4,5 triphosphate receptor (IP_3_R) whose opening allows Ca^2+^ flow from the ER to the mitochondria. We wanted to explore this possibility by impeding the activation of IP_3_R using thapsigargin (THA), a Ca^2+^ ATPase inhibitor [52]. Thapsigargin is also known to produce ER stress to the cells since this treatment depletes Ca^2+^ stores from ER, and therefore increases GRP78 expression as part of the canonical unfolded protein response [53]. Figure 4F shows that thapsigargin affects cell viability similarly to tunicamycin treatment in GFP-control cells and that CCCP concomitant treatment prevents cell death as observed with Tun. However, forced overexpressing of GRP78 was not capable of blocking the detrimental thapsigargin effect as it did with the tunicamycin insult. This observation confirms the necessary involvement of Ca^2+^ flux in the neuroprotective effect of GRP78, probably through IP_3_R.

#### 3.1.4. Neuroprotection Mediated by GRP78 Depends on PINK1 and IP3R

**Figure 5:**
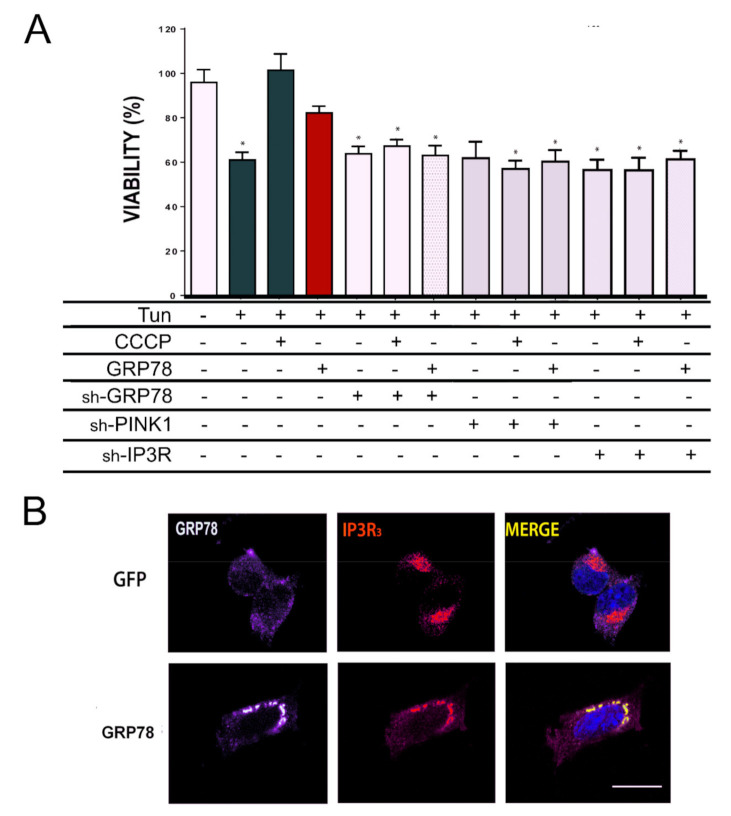
IP3R and PINK1 are necessary for GRP78-mediated neuroprotection. (**A**) The percentage of cell survival (means ± SEM) in NSC34 cells treated with indicated solutions (Tun or CCCP) or submitted to nucleofection of specified plasmids (GFP as a control, GRP78 or shRNA against GRP78, PINK1, IP3R). Cells that did not overexpress GRP78 were GFP positive. Viability was determined by MTT assay after 24 h (GFP-Veh is the control group) (*n* = 4–8, * *p* < 0.05 vs. GFP-expressing vehicle-treated cells). (**B**) Representative confocal images of GRP78 (red) and IP3R (green) in cells that overexpress GFP or GRP78. Scale bar = 10 µm.

To initiate the identification of the mediators of the GRP78 neuroprotective effect, we used shRNA technology. First, we verified that the shRNA chosen reduced the expression of GRP78, PINK1, or IP3R, respectively (Figure S4A–C) and that they did not compromise the viability of control cells (Figure S4D). We first confirmed that the expression of GRP78 itself was necessary for both GRP78- and CCCP-mediated neuroprotection to face Tun-induced ER stress (Figure 5A). Next, silencing either PINK1 or IP_3_R blocked the neuroprotective effect promoted by GRP78 overexpression or CCCP treatment (Figure 5A). Furthermore, we found the GRP78 shift, normally distributed in a smooth spotty distribution around the nucleus, to be totally co-localized to IP_3_R in speckle foci when it was overexpressed (Figure 5B).

Together these results suggested that GRP78 modulates protective mitophagy in our in vitro model, and it exerts this neuroprotection in a PINK1- and IP_3_R-dependent manner.

## 4. Discussion

Boosting the endogenous mechanisms of neuroprotection may yield efficient therapeutic tools to prevent neurodegenerative processes after traumatic lesions or disease [3,54,55]. The ER-resident chaperone GRP78 is at the crossroad of several of these mechanisms promoting neuroprotection when overexpressed in several disease models [4]. To determine the underlying mechanisms, we used a model of spinal root avulsion [55] that disrupts motoneuron connectivity causing a retrograde neurodegenerative process [20]. Motoneuron death in this model is characterized by ER-stress, a blocked autophagy flux, and is non-apoptotic since no active forms of caspase 3 or 12 were observed, indicating that apoptosis is not the final executor of neuronal demise [20]. In accordance, we previously verified with proteomics approaches that anti-apoptotic features occur in parallel to apoptotic ones after RA, blocking an effective apoptosis execution [25]. In that neurodegenerative context, the overexpression of GRP78 exerted motor neuroprotection [20,26,27]. Here, initially, we used unbiased comparative and quantitative analysis of the proteome to uncover the primary basis for this neuroprotection. Unexpectedly, mitochondria were the main target, with a reduced organelle protein content accompanied with decorations of engulfed mitochondria into vesicles within damaged motoneurons that overexpressed GRP78. These observations suggested the presence of mitophagy as a key element for neuroprotection. Deeper in vitro experiments validated this hypothesis since GRP78 mediated neuroprotection by (i) restoring mitochondria respiration and ROS levels, (ii) stimulating PINK1/PARKIN mitochondria translocation, tagging the organelle for mitophagy, and (iii) depending on IP_3_R function as an essential mediator. To our knowledge, this is the first study showing that GRP78 overexpression promotes protective mitophagy.

In addition to the GRP78 role in chaperoning misfolding proteins and other moonlighting functions as being a calcium-binding protein [4,56], we added its pro-active action to stimulate mitophagy. Moreover, our study joins a set of recent studies linking ER stress to mitochondrial dysfunction [57,58,59]. We found hallmarks for mitophagy in vivo when GRP78 was overexpressed and promoted neuroprotection. The same was observed using in vitro models that mimic some traits of the neurodegenerative process that occurs after RA such as ER stress [20,26]. Quality control of mitochondrial by mitophagy is crucial to monitor the mitochondrial content and metabolism homeostasis. In the literature, it is still controverted whether mitophagy induction promotes survival or cell death in several diseases due to its extensive crosstalk with apoptosis signaling [60], although it has been described that the clearance of damaged mitochondria has a fundamental role in neurodegenerative diseases such as Alzheimer‘s disease, Parkinson’s disease, or in aging [61]. Previously, it has been reported that GRP78 inhibits apoptosis triggered by ER stress by preventing *CHOP* induction [62,63], the activation of caspase 7 [5], or by activating PI3K-AKT-mTOR signaling axis [4]. It has also been demonstrated to maintain low levels of oxidative stress and DNA damage [4,64]. In particular, in cancer cells, it has been demonstrated that GRP78 attenuates ROS by activating protein kinase RNA-like endoplasmic reticulum kinase (PERK)-NRF2 signals [65,66], leading to upregulation of antioxidant-related genes as well as enhancing the protein levels of glycolytic enzymes [67]. Instead, we have found that GRP78 overexpression downregulated glycolytic enzymes (e.g., ENO1 and ENO2) (Supplemental tables). These results may suggest that in our model, the observed attenuation of ROS might be rather related to increased mitophagy, although details of the mechanism involved should be further investigated.

Mitochondrial dysfunction is a common condition in neurodegenerative diseases [68], and mitophagy have been involved in Parkinson’s [49,69], Huntington’s [70,71] and Alzheimer’s diseases and Tauopathies [72]. Several authors pointed out that by proper regulation of mitophagy pathways, the body can avoid harmful oxidative species, regulate the redox balance and homeostasis [73]. Thus, GRP78 overexpression through this controlled mitophagy induction might be seriously considered as a neuroprotective strategy in these diseases.

Neuroprotection mediated by GRP78 overexpression appears to be dependent on a Ca^2+^ flux since it was blocked by BAPTA-AM. BAPTA-AM chelating of Ca^2+^ might intervene at a number of different steps along the autophagic flux pathway, blocking not only the triggering of autophagy but also inhibiting steps in the formation and processing of autophagosomes [74]. In contrast, the use of another agent affecting Ca^2+^ flux, such as thapsigargin, yielded surprising results. Thapsigargin blocks sarco-endoplasmic reticulum Ca^2+^ ATPases (SERCAs), sustaining cytosolic Ca^2+^ elevation, but it also triggers ER stress through chronic depletion of intracellular Ca^2+^ stores and the accumulation of unfolded proteins. In this situation, we observed that GRP78 overexpression could not rescue dying cells, and we suspected that it might be because thapsigargin also impedes IP_3_R action [52]. Basal IP_3_Rs activity and continuous low-level Ca^2+^ flux from ER to mitochondria are essential to promote mitochondrial respiration and cell bioenergetics [75]. GRP78 interacts at MAMs with a complex formed by sigma-1 receptor (SIGR1) and IP3R [76,77]. Upon ER stress, the SIGR1-GRP78 interaction decreases caused by Ca^2+^ depletion, and subsequently boosts Ca^2+^ flux from the ER to mitochondria through IP_3_Rs [76]. In addition, it was also demonstrated that IP_3_R was necessary for Parkin-induced mitophagy in particular to allow mitochondrial clustering downstream Parkin recruitment [78]. Our results agree with that since the reduction of IP_3_R by shRNA technology abolished neuroprotection exerted by GRP78 overexpression in ER-stressed cells.

The observation that GRP78 might increase at the mitochondria fraction itself is interesting as well. GRP78 has been detected within the inner membrane intermediate space and matrix of the mitochondria, although its function in there remains to be elucidated [79]. Nevertheless, and regarding its several functions, we speculate that it can be buffering Ca^2+^. If this was the case, its increased presence within mitochondria in ER-stressed cells when overexpressed might attenuate massive Ca^2+^ influx to avoid drive apoptosis. Further experimentation on that would be very valuable.

### Study Limitations and Future Research

This study paves the way for future analyses on the neuroprotective role of GRP78-dependent mitophagy in neuronal death after neurotrauma. The first limitation of this study is that mitophagy was analyzed in a specific time window after RA, and since mitophagy is a dynamic process, it would be interesting to decipher its flow and which molecules are involved. Thus, further experiments modulating mitophagy (through pharmacological or genetic approaches) are needed to decipher whether the effects observed in vivo are purely dependent on mitophagy. This will allow us to confirm that overexpression of GRP78 promotes MN survival through mitophagy. Since there is a well-described crosstalk between mitochondria and apoptosis, further in vitro and in vivo manipulations are essential to deciphering which death mechanism triggers MN death in our model. This will help to elucidate whether GRP78 directly modulates that cell death mechanism by blocking it or whether its presence allows MNs to cope with it and survive.

## 5. Conclusions

The present study was the first to describe a novel role for GRP78 in modulating mitophagy to achieve motoneuronal protection. Our results reveal that GRP78 could drive mitophagy to promote neuroprotection of degenerating motor neurons following severe traumatic nerve injury, restoring damaged mitochondrial function in neuronal cells. Moreover, this GRP78-mediated neuroprotection is dependent on PINK1 and IP_3_R. Therefore, the activation of fine-tuned mitophagy, through gene therapy with GPR78 or other candidates, may be used as a novel therapeutic approach for traumatic injuries of the nervous system.

## Data Availability

All data generated or analyzed during this study are included in this published article and its supplementary information files.

## Supplementary Materials

The following are available online at https://www.mdpi.com/article/10.3390/biomedicines9081039/s1. Figure S1: Bioinformatic analysis. Figure S2: Endogenous GRP78 levels after Tun treatment. Figure S3: Tun and EFV cause mitochondrial dysfunction in NSC34 cells. Figure S4: No title. Figure S5: Cell viability analysis after shRNA nucleofection The supplementary materials also include the tables with all the proteins detected in the proteomic analysis.

## Special Statement

The authors wish to dedicate a special mention in memory of Caty Casas, an influential scientist, and a wonderful and brave person, who died prematurely at the age of 54 years. It has been an honor and a privilege to share part of her life, her professional capacity, and her ethical and human values.
