# Supplementary material for: GRP78 Overexpression Triggers PINK1-IP3R-Mediated Neuroprotective Mitophagy
Source: Biomedicines. 2021 Aug 18;9(8):1039. doi: 10.3390/biomedicines9081039 (PMC8391647; doi:10.3390/biomedicines9081039)
Supplement: Supplementary file 1 [file biomedicines-09-01039-s001.zip › biomedicines-1236696-supplementary.pdf]

GRP78 Overexpression Triggers PINK1/IP$_3$R-Mediated Neuroprotective Mitophagy

Tatiana Leiva-Rodríguez[1,2*], David Romeo-Guitart[1,2,8 *,#], Pau Muñoz-Guardiola[3], Miriam Polo[4], Celia Bañuls[4], Mireia Herrando-Grabulosa[1,2], Valerie Petegnief[5], Assumpció Bosch[2,3], Jose Miguel Lizcano[3], Nadezda Apostolova[6], Joaquim Forés[7], Caty Casas[1,2,9 *]

Affiliations

[1] Institut de Neurociències (INc) and Department of Cell Biology, Physiology and Immunology, Universitat Autònoma de Barcelona (UAB)

[2] Centro de Investigación Biomédica en Red sobre Enfermedades Neurodegenerativas (CIBERNED), Bellaterra, Barcelona, Spain

[3] INc and Department of Biochemistry and Molecular Biology, UAB, Bellaterra, Barcelona, Spain

[4] Service of Endocrinology. University Hospital Dr. Peset, Foundation for the Promotion of Health and Biomedical Research in the Valencian Region (FISABIO), Valencia, Spain

[5] Department of Brain Ischemia and Neurodegeneration, Institute for Biomedical Research of Barcelona (IIBB), Spanish Research Council (CSIC), Institut d'Investigacions Biomèdiques August Pi Sunyer (IDIBAPS), Barcelona, Spain

[6] Department de Farmacologia, Facultat de Medicina, Universitat de Valencia; CIBERehd, Valencia, Spain

[7] Hand and Peripheral Nerve Unit, Hospital Clínic i Provincial, Universitat de Barcelona, Barcelona, Spain Biotech, S.L, Barcelona, Spain

[8] Present address: Institut Necker Enfants-Malades (INEM), INSERM U1151, Laboratory "Hormonal regulation of brain development and functions"—Team 8, Université Paris Descartes, Sorbonne Paris Cité, 75015 Paris, France.

[9] Died June 29, 2020.

* These authors contributed equally.

# Correspondence should be addressed to:

David Romeo-Guitart, david.romeo-guitart@inserm.fr; Tel: +33-01 40 61 53 57

Caty Casas Louzao, Caty.Casas@uab.es, Tel: +34-935811324

Running title: GRP78 triggers mitophagy

SUPPLEMENTARY INFORMATION

- **Supplementary Figures and Legends**
- **Supplementary Tables and Headings**

SUPPLEMENTARY FIGURES AND LEGENDS

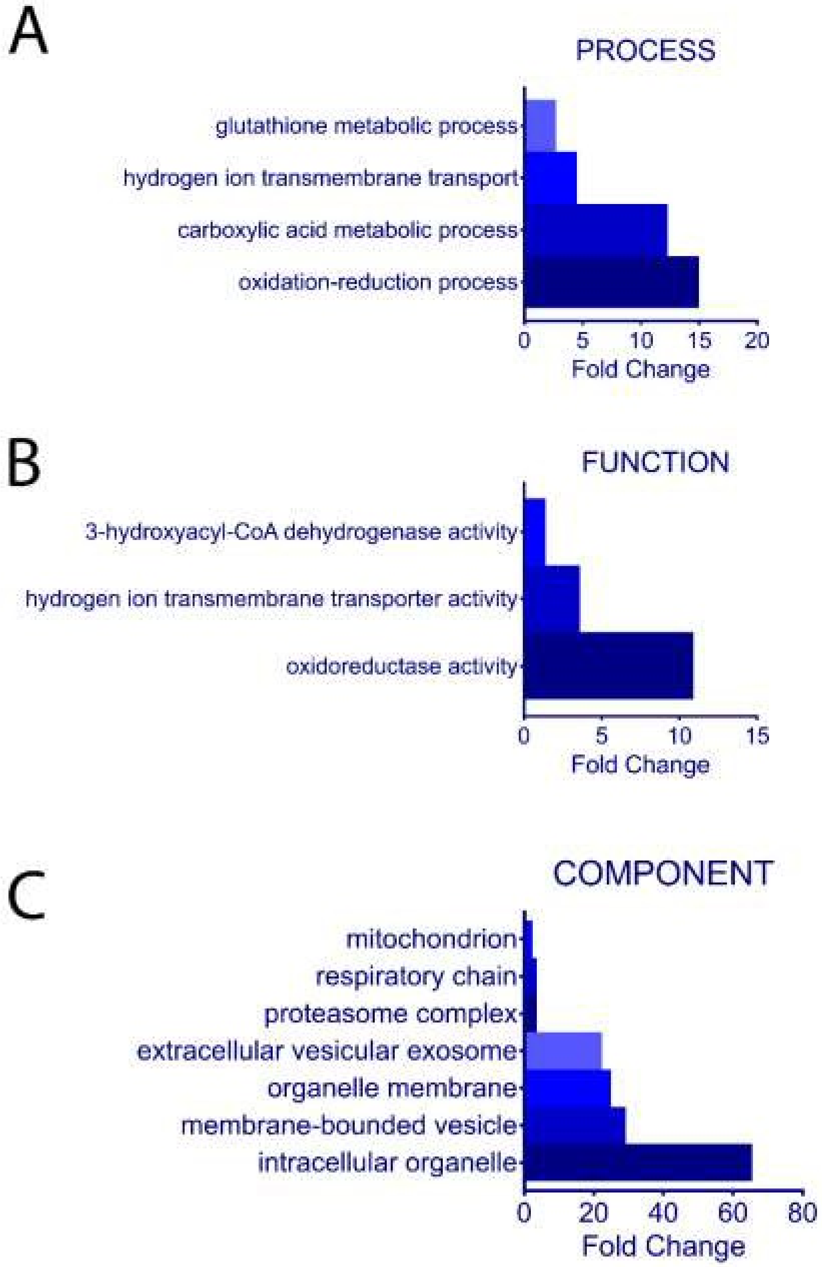

Supplemental Figure 1. Bioinformatic analysis. DAVID bioinformatic analysis of comparative proteomic data from signature proteins specifically modulated by GRP78 overexpression in L4-L5 spinal cord segments from RA-injured model revealed enrichment in mitochondrial processes (**A**), function (**B**), and components (**C**) at 7dpi.

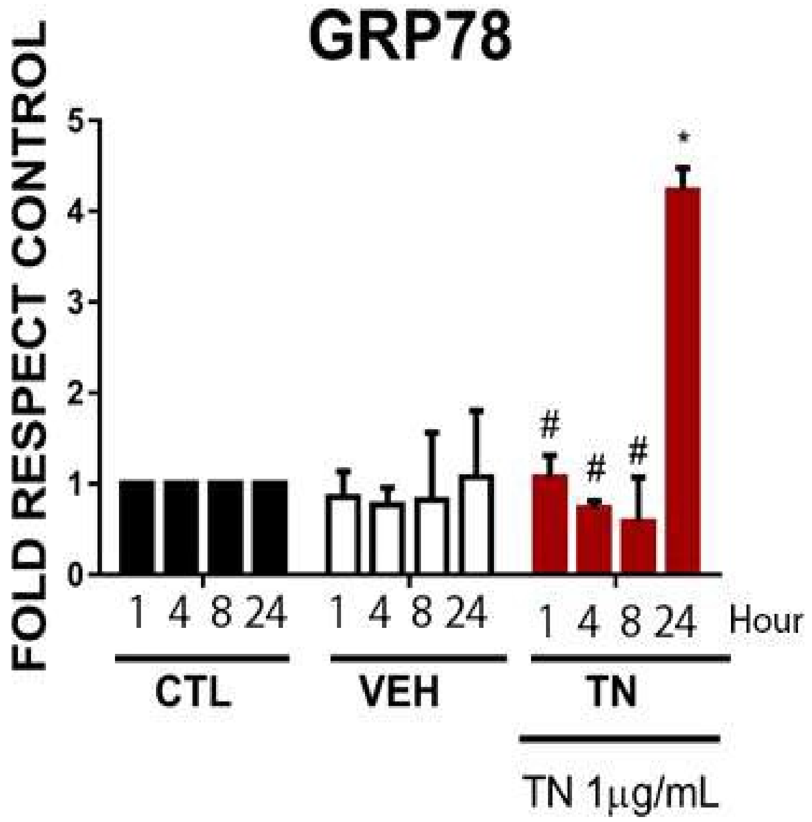

Supplemental Figure S2. Endogenous GRP78 levels after Tun treatment. Bar graft representing average fold change (± SEM) of GRP78 level in NSC-34 cells after 1, 4, 8 and 24 h of Tun treatment by immunoblotting (*$p < 0.05$, Student's t-test vs Veh; #$p < 0.05$, Student's t-test vs TN 24h).

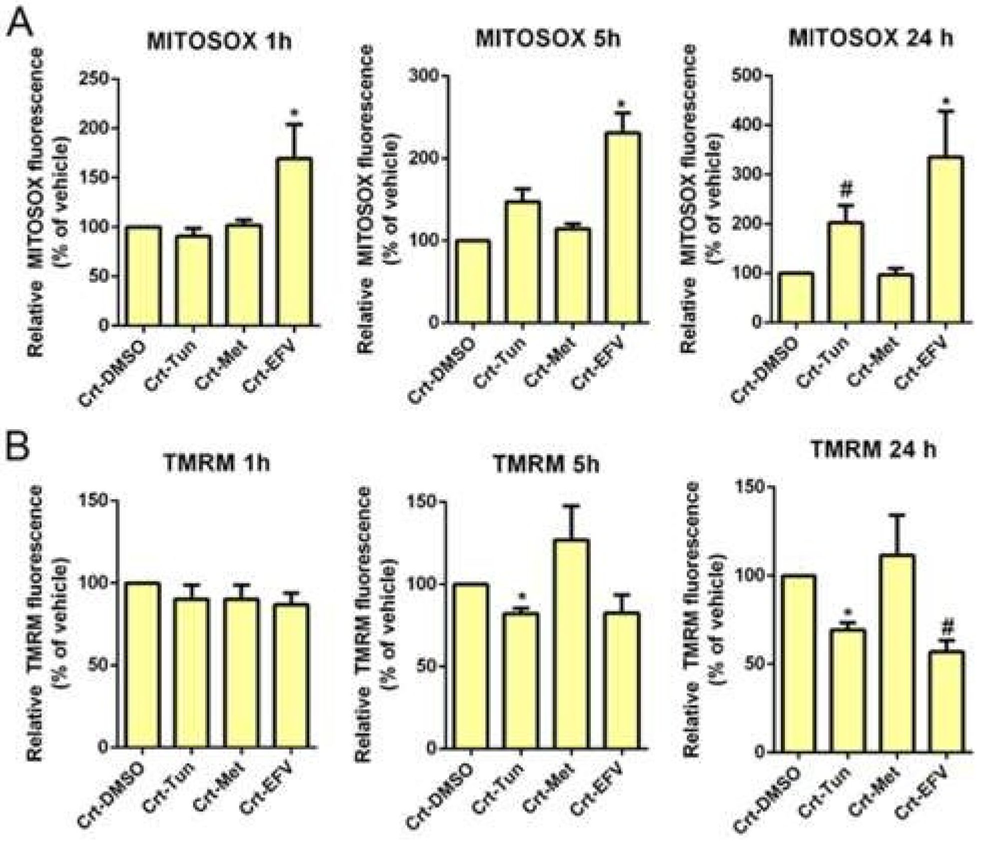

Supplemental Figure S3. Tun and EFV cause mitochondrial dysfunction in NSC34 cells. (A) Quantitative analysis of mitochondrial superoxide production (MitoSOX fluorescence) by NSC34 cells after 1, 5, and 24 h in the presence of 1 μg/mL Tun or 10 μM EFV**. (n=4;** *$p < 0.05$, Student's t-test vs Control-Methanol, #$p < 0.05$, Student's t-test vs Control-DMSO) **(B)** Quantitative analysis of $\Delta\psi m$ (TMRM fluorescence) in NSC34 cells after 1, 5, and 24 h in the presence of 1 μg/mL Tun or 10 μM EFV **(n=4;** #$p < 0.05$, Student's t-test vs Control-Methanol, *$p < 0.05$, Student's t-test vs Control-DMSO)

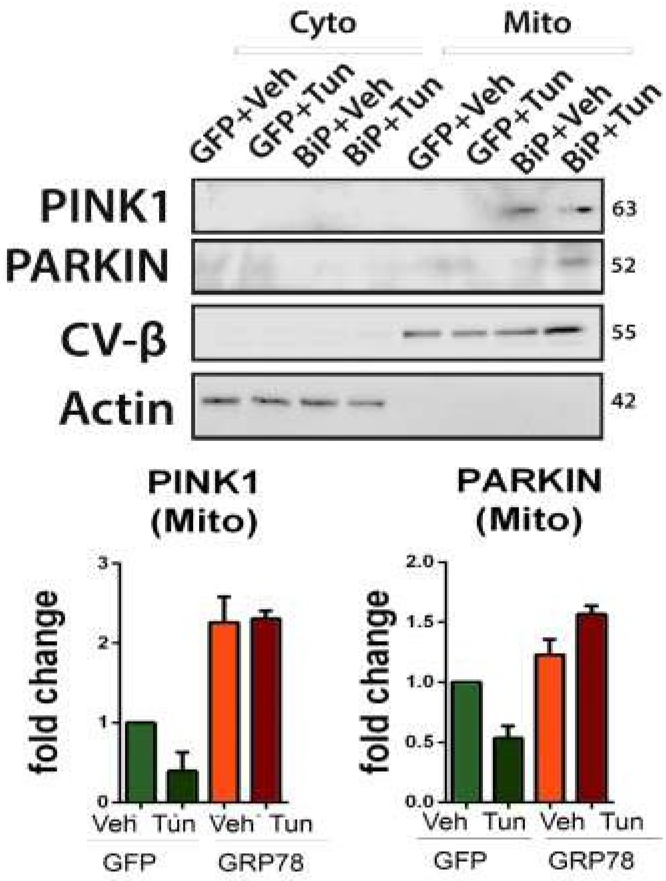

Supplemental Figure S4. *Top*, Western blots for indicated proteins in the mitochondrial (mito) and cytosolic (cyto) pooled fractions from GFP- or GRP78-overexpressing cells treated with vehicle or Tun for 5 hours. *Bottom*, Bar graphs of the average fold change of PINK1 and PARKIN, in both pooled fractions relative to actin (cytosol) or the beta subunit of the complex V (CV-β) (mitochondria) in GFP group.

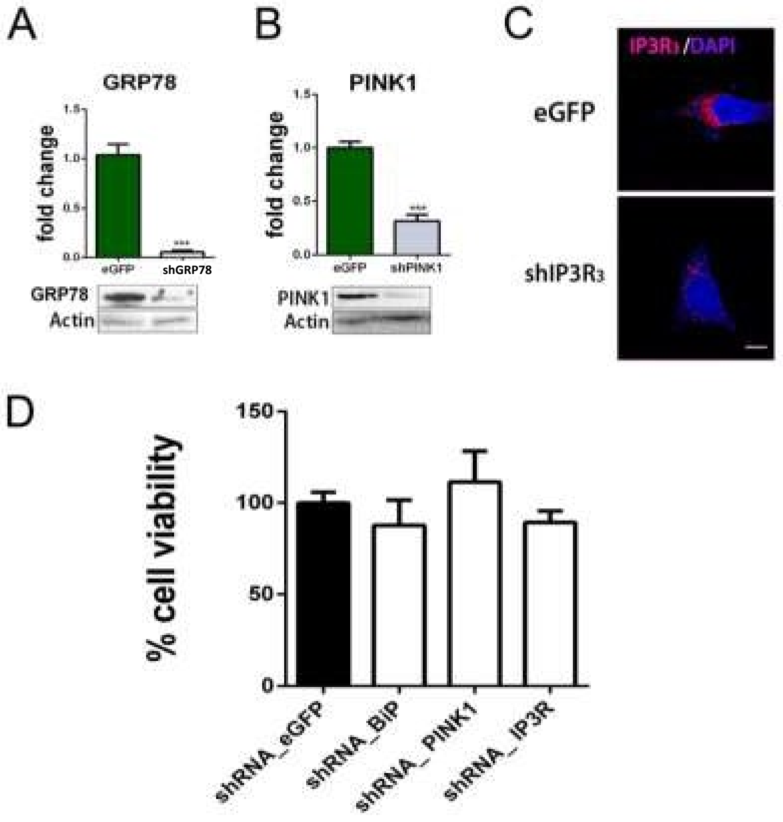

Supplemental Figure S5. Cell viability analysis after shRNA nucleofection. **(A)** Western blot and quantitation of GRP78 levels in cells nucleofected with sh-GRP78 compared to sh-eGFP cells (n =3; *p < 0.05, Student's t-test). **(B)** Western blot and quantitation of PINK1 levels in cells nucleofected with sh-PINK1 cells compared to sh-eGFP cells (n =3; *p < 0.05, Student's t-test). **(C)** Representative microphotographs of cells stained for IP_3R (red) that express sh-IP3R or sh-eGFP. Scale bar = 10 μm. **(D)** NSC-34 were nucleofected with different shRNA and cell viability was assessed by MTT assay. No significant differences were observed.

SUPPLEMENTARY TABLES AND HEADINGS

Supplementary Table SI: Altered proteins by Ad-GRP78-dr iven overexpression after RA

Protein	Description	Gene Name	Entry Name	1ate Fold Ch	Std . Error	t value	DF	p value	adj. p val
B2RYW9	Fumarylacetoacetate hydrolase domain-containing protein 2	Fahd2Fahd2a	FAHD2_RA T	-2,1812718	0,118700273	-18,376299	94	0	
035095	Neurochondrin (Neurite outgrowth-related protein from the rat brain) (Norbin)	Ncdn	NCDN_RAT	-1,0528397	0,110281922	-9,5468018	326		
035112	CD166 antigen {Activated leukocyte cell adhesion molecule)	Alcam	CD166_RAT	-4,9319568	0,161832012	-30,47578	36		
035964	Endophilin-A2 {Endophilin-2)	Sh3gll Sh3p8	SH3GI_RAT	-1,820209	0,082638727	-22,026102	56		
P01026	Complement C3	C3	C03_RAT	0,54585685	0,062336647	8,75659622	328		
P05506	NADH-ubiquinone oxidoreductase chain 3 (EC 1.6 .5 .3) (NADH dehydrogenase subunit3)	Mtnd3 mt-Nd3 Nd3	NU3M_RAT	-8,5750847	0,057716091	-148,57355	11		
P05545	Serine protease inhibitor A3K (Serpin A3K)	Serpina3k Spin2b	SPA3K_RA T	3.4323342	0,10775417	31,8533771	116		
P09812	Glycogen phosphorylase, muscle form (EC 2.4.1.1) {Myophosphorylase)	Pygm	PYGM_RAT	-1,0857768	0,072539943	-14,967986	239		
P11442	Clathrin heavy chain 1	Cite	CLHI _RA T	-0,3627843	0,040986509	-8,8513094	1787		
P13437	3-ketoacyl-CoA thiolase, mitochondrial (EC 2.3.1.16) (Acetyl-CoA acyltransferase) (Beta-ketothiolase) (Mitochondrial 3-oxoacyl-CoA thiolase)	Acaa2	THIM_ RA T	-1,1426851	0,070237659	-16,268838	168		
P16036	Phosphate carrier protein, mitochondrial (Phosphate transport protein) (PTP) (Solute carrier family 25 member 3)	Slc2Sa3Phc	MPCP_RAT	-2,6860151	0,230222996	-11,667015	65		
P16086	Spectrin alpha chain, non-erythrocytic 1 (Alpha-II spectrin) (Fodrin alpha chain)	Sptanl Spna2 Spta2	SPTNI_RAT	-1,0248172	0,032157297	-31,868885	1298		
P19527	Neurofilament light polypeptide (NF-L) (68 kDa neurofilament protein) (Neurofilamenttriplet Lprotein)	Nefl Nf68 Nfl	NFL_RAT	-1.4169231	0,060766211	-23,317615	410		
P26284	Pyruvate dehydrogenase El component subunit alpha, somatic form, mitochondrial (EC 1.2.4 .1) (PDHE1-A type I)	Pdhal	ODPA_RAT	-0,9490399	0,086269588	-11,000863	419		
P34067	Proteasome subunit beta type-4 (EC 3.4.25.1)	Psmb4	PSB4_RAT	1,38238426	0,12870326	10,7408644	137		
P35281	Ras-related protein Rab-10	RablO	RABI O_RA T	-1,7649011	0,115035137	-15,342278	58		
P60901	Proteasome subunit alpha type-6 (EC 3.4.25.1)	Psma6	PSA6_RAT	-1,0130042	0,079516503	-12,739547	133		
P62762	Visinin-like protein 1 (VILIP) (21 kDa CABP) (Neural visinin-like protein 1) (NVL-1) (NVP-1)	Vsnll Visll	VISLI _RAT	-1,5839876	0,095112227	-16,65388	228		
P62824	Ras-related protein Rab-3C	Rab3c	RAB3C_RAT	-2,8410711	0,112469159	-25,26089	53		
Q3MIE4	Synaptic vesicle membrane protein VAT-1 homolog (Mitofusin-binding protein) {Protein MIB)	Vatl	VATI_RAT	-1,211927	0,099689021	-12,157076	238		
Q63041	Alpha-1-macroglobulin (Alpha-1-M)	Alm Pzp	AI M_RA T	0,57974211	0,05303946	10,9303925	429		
Q64057	Alpha-aminoadipic semialdehyde dehydrogenase (Alpha-AASA dehydrogenase) (EC l. 2.1.31)	Aldh7a1 Ald7al	AL7AI _RA T	-1,242213	0,048861086	-25,42336	191		
Q641W2	UPF0160 protein MYGl, mitochondrial	Mygl C12orf10	MYGI _RAT	-2,2235785	0,103885872	-21,40405	42		
Q641Y2	NADH dehydrogenase [ubiquinone] iron-sulfur protein 2, mitochondrial (EC 1.6.5.3) (EC 1.6.99.3) (Complex I-49kD) (CI-49kD) (NADH-ubiquinone oxidoreductase 49 kDa subunit)	Ndufs2	NDUS2_RA T	-2,624384	0,11807109	-22,227152	55		
Q64537	Calpain small subunit 1 (CSS1) (Calcium-activated neutral proteinase small subunit)	Capnsl Capn4 Cssl	CPNSI_RAT	-1,0204072	0,106567993	-9,5751749	135		
Q80230	Protein phosphatase lE (EC 3.1.3 .16) (Ca(2+)/calmodulin-dependent protein kinase phosphataseN)	PpmleCamkn	PPMIE_RAT	2,82690387	0,161772097	17,4746073	31		
Q8VHF5	Citrate synthase, mitochondrial (EC 2. 3. 3.1)	Cs	CISY_RAT	-1,5929535	0,098764169	-16,128861	162		
Q9QWN8	Spectrin beta chain, non-erythrocytic 2 (Beta Spill sigma 1)	Sptbn2Spnb3	SPTN2_RA T	-2,0557489	0,098883861	-20,789529	92		
P05708	Hexokinase-1 (EC 2.7.1.1) (Brain form hexokinase)	Hkl	HXKI _RAT	-0,8129915	0,057370355	-14,170934	372		
P11240	Cytochrome *c* oxidase subunit SA, mitochondrial (Cytochrome *c* oxidase polypeptide Va)	CoxSa	COX5A_RAT	-3,3731214	0,185112385	-18,222019	104		
P12785	Fatty acid synthase (EC2.3 .l. 8S)	Fasn	FAS_RAT	0,51732605	0,047031979	10,9994533	1042		
P15589	Steryl-sulfatase (EC3 .l. 6.2)	Sts	STS_RAT	-4,6862027	0,117625205	-39,840124	26		
P16884	Neurofilament heavy polypeptide (NF-H) (200 kDa neurofilament protein) (Neurofilament triplet H protein)	Nefh Nfh	NFH_RA T	-2.4832361	0,127072683	-19,541856	251		
P19139	Casein kinase II subunit alpha (CK II alpha) (EC 2.7.11.l)	Csnk2al	CSK21_RAT	-1,1041471	0,106336343	-10,383535	134		
P21396	Amine oxidase [flavin-containing] A (EC 1.4 . 3.4) (Monoamine oxidase type A) (MAO-A)	Maoa	AOFA_RA T	-3,0188276	0,083800037	-36,02418	35		
P23565	Alpha-internexin (Alpha-lnx)	Inalnexa	AINX_R AT	-1.4622248	0,084515969	-17,301166	189		
P23978	Sodium- and chloride-dependent GABA transporter 1 (GAT-1) (Solute carrier family 6 member 1)	Slc6al Gabtl Gat-1 Gatl	SC6AI_RAT	-2,6817129	0,123585537	-21,699246	54		
P25093	Fumarylacetoacetase (FAA) (EC 3.7.1.2) (Beta-diketonase) (Fumarylacetoacetate hydrolase)	Fah	FAAA_RAT	3.48656621	0,076693655	45,4609472	38		
P31016	Disks large homolog 4 (Postsynaptic density protein 9S) (PSD-95) (Synapse-associated protein 90) (SAP-90) (SAP90)	Dlg4 Dlgh4 Psd95	DLG4_RAT	-3,8855696	0,205595114	-18,899134	26		
P32551	Cytochrome b-cl comple x subunit 2, mitochondrial (Comple x Ill subunit 2) (Core protein 11) (Ubiquinol-cytochrome-c reductase complex core protein 2)	Uqcrc2	QCR2_RAT	-1,6429346	0,057175893	-28,734743	151		
P38659	Protein disulfide-isomerase A4 (EC S. 3.4 .1) (CaBP2) (ERp72)	Pdia4 Cabp2 Erp70	PDIA4_RA T	-1.4699363	0,070408879	-20,877144	52		
P46844	Biliverdin reductase A (BVR A) (EC 1.3.1.24) (Biliverdin-IX alpha-reductase)	Blvra Blvr	BIEA_RA T	1,2923408	0,112095568	11,5289196	130		
P62243	40S ribosomal protein S8	Rps8	RS8_RAT	-1,7725098	0,059413326	-29,83354	113		
P63047	Sulfotransferase 4Al (ST4Al) (EC 2.8. 2.-) (Brain sulfotransferase-like protein) (rBR-STL) (Nervous system sulfotransferase) (NST)	Sult4a1 Sult x3	ST4Al _RA T	-1.4767043	0,117736449	-12,542457	97		
P81155	Voltage-dependent anion-selective channel protein 2 (VDAC-2) (B36-VDAC) (Outer mitochondrial membrane protein porin 2)	Vdac2	VDAC2_RAT	-1,6426814	0,065908766	-24,923565	169		
P85969	Beta-soluble NSF attachment protein (SNAP-beta) (N-ethylmaleimide-sensitive factor attachment protein beta)	NapbSnapb	SNAB_RAT	-0,9268229	0,058569202	-15,824407	267		
Q00981	Ubiquitin carboxyl-terminal hydrolase isozyme Ll (UCH-Ll) (EC 3.4.19.12) (EC 6.- .-.-) (Neuron cytoplasmic protein 9.5) (PGP 9.5) (PGP9.S) (Ubiquitin thioesterase Ll)	Uchll	UCHLI _RA T	-0,9831311	0,107885145	-9,1127569	353		
Q562C6	Leucinezippertranscription factor-like protein 1	Lztfll	LZTLI _RA T	-2,2173938	0,078741878	-28,160286	70		
Q5Xl72	Eukaryotic translation initiation factor 4H (eIF-4H) (Williams-Beuren syndrome chromosomal region 1 protein homolog)	Eif4h Wbscrl	IF4H_RA T	2,00629568	0,086560472	23,177966	92		
Q63377	Sodium/potassium-transporting ATPase subunit beta-3 (Sodium/potassium-dependent ATPase subunit beta-3) (ATPB-3) (CD antigen CD298)	Atp1b3	AT1B3_RA T	-2,5703051	0,192577582	-13,346855	56		
Q6AYT5	UPF0364 protein C6orf211 homolog		CF211_RA T	-1,5743413	0,089358356	-17,618288	55		
Q7TP36	Protein Shroom2 (Liver regeneration-related protein LRRG167) (Protein Apxl)	Shroom2 Apxl Ab2-404	SHRM _2 RAT	3,05766396	0,13842498	22,0889608	56		

	Vesicle-fusing ATPase (EC 3.6.4.6) (N-ethylmaleimide-sensitive fusion					
Q9QUL6		prote	n	d	(41 kDa	mitochondrial (EC 2. 3.1.9) (Acetoacetyl-CoA thiolase)
		in)	prote	prot	phosphoribosypyro	60S acidic ribosomal protein PO (60S ribosomal protein LIOE)
	P63039 P67874 P84092 Q3B8QO	(NE	in	ein 2	phosphate	Hemopexin
008618		M-	NSF)	(PR	synthetase-	ATP synthase subunit gamma, mitochondrial (F-ATPase gamma subunit)
	Q4KM49	sensi	Phos	PP	associated protein)	Rabphilin-3A (Exophilin-1)
P07340		tive	phori	synt	(PAP41)	ATP-sensitive inward rectifier potassium channel 10 (BIRIO) (BIRKI)
	Q63560Q6P7B0	fusio	bosyl	has	Sodium/potassium-	60S ribosomal protein L7a
P17764		n	pyrop	e-	transporting ATPase	60 kDa heat shock protein, mitochondrial (60 kDa chaperonin) (Chaperonin60)
P19945		prote	hosp	asso	subunit beta-1	(CPN60) (HSP-6S) (HSP-60) (Hsp60) (Mitochondrial matrix protein Pl)
P20059		in)	hate	ciate	(Sodium/potassium-	
P35435		(Vesi	synth	d	dependent ATPase	Casein kinase II subunit beta (CK II beta) (Phosvitin)
P47709		cular	ase-	prot	subunit beta-1)	AP-2 complex subunit mu (AP-2 mu chain)
P49655		-	asso	ein	Acetyl-CoA	Microtubule-associated protein RP/EB family member 2 Tyrosine-
P62425		fusio	ciate	2)	acetyltransferase,	tRNA ligase, cytoplasmic (EC 6.1.1.l) (Tyrosyl-tRNA synthetase)

(TyrRS) Microtubule-associated protein 6 (MAP-6) (14S-kDa STOP) (STOP145) (Stable tubule-only polypeptide) (STOP) Tryptophan-tRNA ligase, cytoplasmic (EC 6.1.1 . 2) (Tryptophanyl-tRNA synthetase)(TrpRS)		NsfErgl	NSF_RAT	-0,6299	0,059112146 - 10,656016		821
		Prpsap2	KPRB_RAT	-1.4873079			111
		Atplbl	ATI BI _RAT	-1,5939774	0,1054526 - 14,104042		315
		Acatl RplpO Arbp	THIL_RAT	-0,6871126			265
		Hp,	RLAO_RAT	-2,7887844			75
		AtpScl Atp5cRph3a KcnjlOKab-2 Rpl7a	HEMO_RAT	0,90320598	0,090186498 - 17,674236		180
			ATPG_RAT	-1,7067515			107
			RP3A_RAT	-1,1980596			183
		Hspdl Hsp60	KO I O_RA T	-2,629205	0,073727567 - 9,3196153		59
			RL7A_RAT	-3,0459986			52
		Csnk.2bCk2nAp2m1 Mapre2			0,171143596 -		
		Yars	CH60_RAT	-0,9367388	16,294997		645
		Map6Mtap6 Wars	CSK2B_RAT	-2,8849329	0,080406849 11,2329484		32
			AP2MI_RAT	-0,9617552			235
			MARE2_RA T	-2,124534	0,163544051 - 10,436036		62
			SYYC_RAT	-0,8841412	0,067943615 -17,633145		292
			MAP6_RAT	0,60824776	0,188130806 - 13,975409		280
			SYWC_RA T	-0,7719696	0,101828493 - 29,913029		230
					0,071580507 - 13,086506		
					0,142859978 - 20,194129		
					0,066633601 - 14,433486		
					0,090375053 -23,50797		
					0,062936153 -14,048225		
					0,06221259 9,7769239 1		
					0,07865274 - 9,8149104		
Q8K4YS	Leucine-rich glioma-inactivated protein 1	Lgil	LGll_RAT	-2,6193993	0,160654589	-16,304541	42
035179	Endophilin-Al (Endophilin-1) (SH3p4)	Sh3gl2 Sh3d2a Sh3p4	SH3G2_RAT	-1,1540809	0,058548727	-19,71146	323

P08461	Dihydrolipoyllysine-residue acetyltransferase component of pyruvate dehydrogenase complex, mitochondrial (EC 2.3.1.12) (PDC-E2) (PDCE2)	Dlat	ODP2_RAT	-1,1032801	0,072612382	-15,194104	256		
P17425	Hydroxymethylglutaryl-CoA synthase. cytoplasmic (HMG-CoA synthase) (EC 2.3.3.10)	Hmgcs1Hmgcs	HMCS1_RAT	-1,46S136	0,110540937	-13,254239	212		
Q01205	Dihydrolipoyllysine-residue succinyltransferase component of 2-oxoglutarate dehydrogenase complex, mitochondrial (EC 2.3.1.61) (E2K)	Dist	0D02_RA T	1,346927S6	0,062837025	21,4352534	133		
Q05962	ADP/ATP translocase 1 (ADP,ATP carrier protein 1) (Adenine nucleotide translocator 1) (ANT 1) (Solute carrier family 25 member 4)	Slc25a4Ant1	ADT1_RAT	-0,868180S	0,074579109	-11,641069	87		
Q499N5	Acyl-CoA synthetase family member 2, mitochondrial (EC 6.2.1.-)	Acsf2	ACSF2_RAT	-1,0240636	0,063991128	-16,003212	224		
Q5U2N3	Membrane-associated phosphatidylinositol transfer protein 1 (Phosphatidylinositol transfer protein, membrane-associated 1) (PITPnm 1) (Pyk2 N-terminal domain-interacting receptor 2) (NIR-2)	Pitpnm1 Nir2 Pitpnm	PITM I _RAT	-1,1390133	0,0741904	-15,35257	58		
035458	Vesicular inhibitory amino acid transporter (GABA and glycine transporter) (Solute carrier family 32 member 1) (Vesicular GABA transporter) (rGVAT)	Slc32a1 VgatViaat	VIAAT_RAT	-1,6727S28	0,125831851	-13,293556	156		
P13383	Nucleolin (ProteinC23)	Ncl Nuc	NUCL_RAT	-1,096616S	0,078239447	-14,016159	192		
Q63692	Hsp90 co-chaperone Cdc37 (Hsp90 chaperone protein kinase-targeting subunit) {p50Cdc37)	Cdc37	COC37_RA T	0,84931826	0,07230529	11,746281	246		
Q99N27	Sortingnexin-1	Snx1	SNX1_RAT	-1,7030S62	0,056556334	-30,112564	88		
Q09073	ADP/ATP translocase 2 (ADP.ATP carrier protein 2) (ANT 2)	Slc25a5Ant2	ADT2_RAT	-0,844889S	0,069173475	-12,214068	110		
Q68FYO	Cytochrome b-c1 complex subunit 1, mitochondrial (Complex III subunit 1) (Core protein I) (Ubiquinol-cytochrome-c reductase complex core protein 1)	Uqcrc1	QCR1_RAT	-1,3491084	0,135806814	-9,9340262	174		
P07895	Superoxide dismutase [Mn]. mitochondrial (EC 1.15.1.1)	Sod2	SODM_RAT	-2,1440308	0,104980626	-20,423109	103		
P18445	60S ribosomal protein L27a	Rpl27a	RL27A_RAT	-4,3S98299	0,097389886	-44,766762	30		
Q5U2RO	Methionine adenosyltransferase 2 subunit beta (Methionine adenosyltransferase II beta) (MAT II beta)	Mat2b	MAT2B_RAT	-3,6477744	0,156321179	-23,335126	28		
P24268	Cathepsin D {EC3.4.23.5)	Ctsd	CATD_RAT	0,68347913	0,068775236	9,93786662	145		
Q922LO	Voltage-dependent anion-selective channel protein 1 (VDAC-1) (rVDAC1) (Outer mitochondrial membrane protein porin 1)	Vdac1	VDAC1_RAT	-0,628027S	0,040551389	-15,487201	191		
Q80W89	NADH dehydrogenase [ubiquinone] 1 alpha subcomplex subunit 11 (Complex I-B14.7) (CI-B14.7) {NADH-ubiquinone oxidoreductase subunit B14.7)	Ndufa11	ND UAB_RAT	-0,9288S99	0,058117741	-15,982381	65		
035509	Ras-related protein Rab-11B	Rab11b	RB11B_RAT	-0,7638187	0,056453291	-13,5301	43		
P14841	Cystatin-C(Cystatin-3)	Cst3	CYTC_RAT	O,S8346S6S	0,036527762	15,9732111	43		
Q62667	Major vault protein (MVP)	Msp	MVP_RAT	0,546498S	0,058925174	9,27444871	152	2,22E-16	3,48E-15
P29266	3-hydroxyisobutyrate dehydrogenase, mitochondrial (HIBADH) (EC 1.1.1.31)	Hibadh	3HIDH_RA T	-1,242493	0,13406841	-9,2676048	142	4,44E-16	6,61E-15
Q4KMA2	UV excision repair protein RAD23 homolog B	Rad23b	RD23B_RA T	1,731SS3S7	0,158908155	10,8965683	63	4,44E-16	6,61E-15
Q58FK9	Kynurenine-oxoglutarate transaminase 3 (EC 2.6.1.7) (Cysteine-S-conjugate beta-lyase 2) (EC 4.4 .1.13) (KATIII) (Kynurenine--glyoxylate transaminase) (EC 2.6.1.63)	Ccbl2 Kat3	KAT3_RAT	-7,4696132	0,01035854	-721,10679		4,44E-16	6,61E-15
Q9ESB5	N-terminal EF-hand calcium-binding protein 1 (EF-hand calcium-binding protein 1) {Neuronal calcium-binding protein 1) (Synaptotagmin-interacting protein 1) (Stip-1)	Necab1 Efcbp1 Stip	NECA1_RAT	4,872901S7	0,068260497	71,3868459	11	4,44E-16	6,61E-15
P18088	Glutamate decarboxylase 1 (EC 4.1.1.15) (67 kDa glutamic acid decarboxylase) (GAD-67) (Glutamate decarboxylase 67 kDa isoform)	Gad1Gad67	DCE1_RAT	-1,6270022	0,168265692	-9,669245	101	4,44E-16	6,61E-15
Q5BJT7	Coiled-coil domain-containing protein 93	Ccdc93	CCD93_RA T	-3,6337384	0,194796264	-18,654045	24	8,88E-16	1,31E-14
Q6P7A9	Lysosomal alpha-glucosidase (EC 3.2.1.20) (Acid maltase)	Gaa	LYAG_RAT	1,87811S6	0,158141485	11,8761728	45	1,78E-15	2,59E-14
Q5XICO	Enoyl-CoA delta isomerase 2, mitochondrial (EC 5.3.3.8) (Delta(3),delta(2)-enoyl-CoA isomerase) (D3,D2-enoyl-CoA isomerase) (Dodecenoyl-CoA isomerase) (Peroxisomal 3,2-trans-enoyl-CoA isomerase) (pECI)	Eci2Peci	ECl2_RAT	-3,34188S2	0,092471561	-36,1396	14	3,11E-15	4,44E-14
Q5U2PO	DIS3-like exonuclease 1 (EC 3.1.13.-)	Dis3l	Dl3L1_RAT	-1,31073S1	0,045417834	-28,859481	16	3,11E-15	4,44E-14
P18484	AP-2 complex subunit alpha-2 (100 kDa coated vesicle protein C) (Adaptor protein complex AP-2 subunit alpha-2) (Adaptor-related protein complex 2 subunit alpha-2) {Alpha-adaptin C) (Alpha2-adaptin) {Clathrin assembly protein complex 2 alpha-C large chain) (Plasma membrane adaptor HA2/AP2 adaptin alpha Csubunit)	Ap2a2Adtab	AP2A2_RA T	-0,7083282	0,085977237	-8,2385547	327	4,22E-15	5,97E-14
Q8R491	EH domain-containing protein 3	Ehd3Ehd2	EHD3_RAT	-1,8549946	0,176972901	-10,4818	59	4,44E-15	6,22E-14
P37805	Transgelin-3 (Neuronal protein 22) (NP22) (Neuronal protein NP25)	Tagln3Np25	TAGL3_RA T	-1,6S38184	0,182636999	-9,0552208	112	4,88E-15	6,78E-14
Q62915	Peripheral plasma membrane protein CASK (EC 2.7.11.1) (Calcium/calmodulin-dependent serine protein kinase)	Cask	CSKP_RA T	-3,3948217	0,123993685	-27,378989	16	7,11E-15	9,76E-14
P97700	Mitochondrial 2-oxoglutarate/malate carrier protein (OGCP) (Solute carrier family 25 member 11)	Slc25a11 Slc20a4	M20M_ RA T	-8,8174964	0,065313001	-135,00369		1,02E-14	1,39E-13
Q9ER34	Aconitate hydratase, mitochondrial (Aconitase) (EC 4.2.1.3) (Citrate hydro-lyase)	Aco2	ACON_RA T	-O,S293227	0,067711069	-7,817373	815	1,67E-14	2,25E-13
P10719	ATP synthase subunit beta, mitochondrial (EC 3.6.3.14)	Atp5b	ATPB_RAT	-0,7034667	0,090755803	-7,7512038	824	2,66E-14	3,56E-13
P15205	Microtubule-associated protein IB (MAP-1B) (Neuraxin) (Cleaved into: MAP1B heavy chain; MAPI light chain LC1]	Map1b	MAP1B_RAT	-0,2S61448	0,033348287	-7,6808991	1309	3,06E-14	4,05E-13
P62832	60S ribosomal protein L23	Rpl23	RL23_RAT	-1,2206837	0,048920532	-24,95238	16	3,09E-14	4,05E-13
P85125	Polymerase I and transcript release factor (Cavin-1) (cav-p60)	Ptrf	PTRF_RA T	-1,0026427	0,040282974	-24,889987	16	3,20E-14	4,16E-13
CON_007044	Succinate dehydrogenase [ubiquinone] flavoprotein subunit, mitochondrial (EC 1.3.5.1) (Flavoprotein subunit of complex II) (Fp)	Sdha	SDHA_RAT	O,S9921808	0,047399765	12,6417943	32	5,48E-14	7,07E-13
Q920L2	2',3'-cyclic-nucleotide 3'-phosphodiesterase (CNP) (CNPase) (EC 3.1.4.37)	CnpCnp1	CN37_RA T	-1,2033831	0,14609419	-8,2370363	154	7,11E-14	9,07E-13
P13233	NADH dehydrogenase [ubiquinone] flavoprotein 2, mitochondrial (EC 1.6.5.3) (EC 1.6.99.3) (NADH-ubiquinone oxidoreductase 24 kDa subunit)	Ndufv2	NDUV2_RAT	-O,S003202	0,065472735	-7,6416567	559	9,37E-14	1,19E-12
P19234	Branched-chain-amino-acid aminotransferase, mitochondrial (BCAT(m)) (EC 2.6.1.42)	Bcat2 Bcatm Eca 40	BCAT2_RAT	-0,4S69204	0,047213383	-9,6777725	56	1,48E-13	1,86E-12
035854	4- aminobutyrate aminotransferase, mitochondrial (EC 2.6.1.19) ((S)-3-amino-2-methylpropionate transaminase) (EC 2.6.1.22) (GABA aminotransferase) (GABA-AT) (Gamma-amino-N-butyrate transaminase) (GABA transaminase) (GABA-T) (L-AIBAT) (Cleaved into : 4-aminobutyrate aminotransferase, brain isoform; 4-aminobutyrate aminotransferase, liver isoform]	AbatGabat	GABT_RAT	-6,2204674	0,146889141	-42,348041	11	1,55E-13	1,93E-12
P50554	Syntaxin-binding protein 1 (N-Sec1) (Protein unc-18 homolog 1) (Unc18-1) (Protein unc-18 homolog A) (Unc-18A) (p67) (rbSec1)	Stxbp1 Unc18a	STXB1_RAT	-0,977S331	0,126406652	-7,7332412	286	1,80E-13	2,22E-12
P61765-2	Macrophage-capping protein (Actin regulatory protein CAP-G)	Capg	CAPG_RAT	-1,90S1981	0,208272802	-9,1476089	66	2,38E-13	2,91E-12
Q6AYC4	Monoglyceride lipase (MGL) (EC 3.1.1.23) (Monoacylglycerol lipase) (MAGL)	Mg11Mgl2	MG LL_RAT	-O,S879888	0,069674521	-8,4390796	101	2,40E-13	2,91E-12
Q8R431	60S ribosomal protein L18	Rpl18	RL18_RAT	-6,4422294	0,074843897	-86,075547		3,70E-13	4,45E-12
P12001	ATP synthase subunit f, mitochondrial	Atp5j2	ATPK_RA T	-0,48S1S17	0,056624127	-8,5679321	82	5,21E-13	6,21E-12
D3ZAF6	Serine/threonine-protein phosphatase 28 catalytic subunit alpha isoform (EC 3.1.3.16) (CAM-PRP catalytic subunit) (Calmodulin-dependent calcineurin A subunit alpha isoform)	Ppp3ca Calna	PP2BA_RA T	-1,6630871	0,074740911	-22,251363	15	6,69E-13	7,92E-12
P63329	40S ribosomal protein S30	[illegible]	RS30_RAT	7,73927007	0,065281009	118,553163		8,01E-13	9,40E-12
P62864	Sodium/potassium-transporting ATPase subunit alpha-2 (Na(+)/K(+) ATPase alpha-2 subunit) {EC 3.6.3.9) (Na{+)/K{+) ATPase alpha(+) subunit) (Sodium pumpsubunitalpha-2)	Atp1a2	AT1A2_RAT	-0,8143989	0,043304189	-18,80647	16	2,47E-12	2,87E-11
P06686	Tetraspanin-2 (Tspan-2)	Tspan2	TSN2_RAT	-0,54SSS9	0,076635751	-7,1188572	771	2,49E-12	2,88E-11
Q9JJW1	2,4-dienoyl-CoA reductase, mitochondrial (EC 1.3.1.34) (2,4-dienoyl-CoA reductase [NADPH]) (4-enoyl-CoA reductase (NADPH])	Decr1 Deer	DECR_RAT	-3,S009969	0,128781438	-27,185571	12	3,78E-12	4,33E-11
Q64591	Cytochrome b-c1 complex subunit 8 (Complex III subunit 8) (Complex III subunit VIII) (Low molecular mass ubiquinone-binding protein) (Ubiquinol-cytochrome c reductase complex 9.5 kDa protein) (Ubiquinol-cytochrome c reductase complex ubiquinone-binding protein QP-C)	Uqcrq Qpc	QCR8_RAT	-6,148706S	0,096157582	-63,944063		3,98E-12	4,52E-11
Q7TQ16	Alpha-1-antiproteinase (Alpha-1-antitrypsin) (Alpha-1-proteinase inhibitor) (SerpinA1)	Serpina1	Al AT_RAT	-7,674969S	0,163640945	-46,901278		4,56E-12	5,14E-11
P17475	Cytochrome c oxidase subunit 4 isoform 1, mitochondrial (Cytochrome c		COX41_RAT	0,54130201	0,070327327	7,69689447	109	6,73E-12	7,53E-11
			QCR6_RAT	-O,S7S26	0,076214726	-7,5478856	122	8,86E-12	9,84E-11

o x i d a	se polypeptide IV) (Cytochrome c oxidase subunit IV isoform 1) (COX IV-1)	Cox4i1 Cox4 Cox4a
P10888	Cytochrome b-cl complex subunit 6, mitochondrial (Complex III subunit 6) (Complex III subunit VIII) (Cytochrome cl non-heme 11 kDa protein) (Mitochondrial hinge protein) (Ubiquinol-cytochrome c reductase complex 11 kDa protein)	Uqcrh
Q5M9I5	Hippocalcin-like protein 1 (Neural visinin-like protein 3) (NVL-3) (NVP-3) (Visinin-like protein 3) (VILIP-3)	Hpcal1
P62749	Mitochondrial-processing peptidase subunit beta (EC 3.4.24.64) (Beta-MPP) (P-52)	PmpcbMppb

Q03346	Seel family domain-containing protein 1 (SLYI homolog) (Slylp) (Syntaxin-binding protein 1-like 2) (Vesicle transport-related protein Ra410)	Scfdl Ra410 Slyl Stxbpll2	SCFDl_RAT	-6,0308487	0,232954419	-25,888535	11	3,30E-11	3,58E-10
Q62991	Pyridoxal phosphate phosphatase (PLP phosphatase) (EC 3.1.3.3) (EC 3.1.3.74) (Chronophin) (Reg I-binding protein 1)	PdxpCin Pip Plpp Rbpl	PLPP_RAT	-3,3747946	0,133301782	-25,316951	11	4,21E-11	4,53E-10
Q8VD52	Legumain (EC 3.4.22.34) (Asparaginyl endopeptidase) (Protease, cysteine 1)	Lgmn Prscl	LGMN_RAT	0,68954923	0,098049601	7,03265718	173	4,51E-11	4,82E-10
Q9ROJ8	Maleylacetoacetate isomerase (MAAI) (EC 5. 2.1.2) (GSTZl-1) (Glutathione s-transferase zeta I)(EC2.5.1.18)	Gstzl	MAAI_RAT	0,98961054	0,061181241	16,1750648	15	6,66E-11	7,07E-10
P57113	Carbonic anhydrase 1 (EC 4.2.1.1) (Carbonate dehydratase I) (Carbonic anhydrase I)(CA-I)	Cal Carl	CAHI _RAT	-0,9748932	0,056031499	-17,39902	14	7,04E-11	7,42E-10
BOBNN3	Diphosphomevalonate decarboxylase (EC 4.1.1.33) (Mevalonate (diphospho)decarboxylase) (MDDase) (Mevalonate pyrophosphate decarboxylase)	MvdMpd	MVDI_RAT	0,6252686	0,084712849	7,38103616	89	7,93E-11	8,30E-10
Q62967	Trifunctional enzyme subunit alpha, mitochondrial {TP-alpha) (Includes: Long-chain enoyl-CoA hydratase (EC 4.2.1.17); Long chain 3-hydroxyacyl-CoA dehydrogenase {EC 1.1.1.211)]	Hadha	ECHA_RAT	0,39284255	0,042414915	9,26189626	34	8,00E-11	8,31E-10
Q64428	Redox-regulatory protein FAM213A (Peroxiredoxin-like 2 activated in M-CSF stimulated monocytes) (Protein PAMMI (Sperm head protein 1)	213a PammShp I	F213A RA T	-0,6102212	0,087897281	-6,9424357	163	8,68E-11	8,95E-10
Q6AXX6	Heat shock protein HSP 90-beta (Heat shock 84 kOa) (HSP 84) (HSP84)	Hsp90abl Hsp84 Hspcb	HS90B_RA T	-0,2853735	0,032507804	-8,7786156	39	8,94E-11	9,15E-10
P34058	Branched-chain-amino-acid aminotransferase, cytosolic (BCAT(c)) (EC 2.6.1.42)	Bcatl	BCATl_RAT	-0,3904599	0,059108236	-6,6058457	558	9,23E-11	9,38E-10
P54690	Annexin A3 (35-alpha calcimedin) (Annexin III) (Annexin-3) (Upocortin III) (Placental anticoagulant protein III) (PAPI-II)	Anxa3Anx3	ANXA3_RA T	-0,5800469	0,086657659	-6,6935445	295	1,IOE-10	1,IIE-09
P14669	Aspartate aminotransferase, mitochondrial (mAspAT) (EC 2.6.1.1) (EC 2.6.1.7) (Fatty acid-binding protein) (FABP-1) (Glutamate oxaloacetate transaminase 2) (Kynurenine aminotransferase 4) (Kynurenine aminotransferase IV) (Kynurenine- o xoglutarate transaminase 4) (Kynurenine--oxoglutarate transaminase IV) (Plasma membrane-associated fatty acid-binding protein) (FABPpm) (Transaminase A)	Got2Maat	AATM_RAT	0,447792	0,067821955	6,60246372	446	1,15E-10	1,15E-09
P00507	Prohibitin	Phb	PHB_RAT	-0,4586467	0,069620077	-6,5878505	402	1,40E-10	1,39E-09
P67779	Tropomodulin-1{Erythrocyte tropomodulin) (E-Tmod)	Tmodl Tmod	TMODI_RAT	-0,4637257	0,059938418	-7,7367026	57	1,88E-10	1,86E-09
P70567	Synapsin-2(Synapsin II)	Syn2	SYN2_RAT	-7,3010674	0,187728988	-38,891529		2,IOE-10	2,06E-09
Q63537	60S ribosomal protein L4 (60S ribosomal protein LI)	Rpl4 Rpll	Rl4_RA T	-7,4958207	0,194381212	-38,562475		2,25E-10	2,19E-09
P50878				-0,6342171	0,082002017	-7,734165	55	2,37E-10	2,29E-09
Q9WVA1	Mitochondrial import inner membrane translocase subunit Tim8 A (Deafness dystonia protein 1 homolog) / Ubiquitin carboxyl-terminal hydrolase 7 (EC 3.4.19.12) (Deubiquitinating enzyme 7) (Herpesvirus-associated ubiquitin-specific protease) (rHAUSP) (Ubiquitin thioesterase 7) (Ubiquitin-specific-processing protease 7)	Timm8a Ddp1 Tim8a / Usp7 Hausp	TIM8A_RAT / UBP7_RAT	-0,8442467	0,070345932	-12,001357	19	2,59E-10	2,49E-09
Q4VSl4	NAO-dependent protein deacylase sirtuin-5, mitochondrial (EC 3.5.1.-) (Regulatory protein SIR2 homolog 5) (SIR2-like protein 5)	Sirt5	SIR5_RAT	1,96309335	0,248780096	7,89087783	49	2,81E-10	2,68E-09
Q68FX9	Proteasome activator complex subunit 1 (llS regulator complex subunit alpha) (REG-alpha) (Activator of multicatalytic protease subunit 1) (Proteasome activator 28 subunit alpha) (PA28a) (PA28alpha)	Psmel	PSMEI _RA T	-2,3676545	0,275511702	-8,5936622	36	3,02E-10	2,87E-09
Q63797	Annexin A6 {Annexin VI) (Annexin-6) (Calcium-binding protein 65/67) (CBP 65/67)	Anxa6Anx6	ANXA6_RA T	0,556117	0,083599722	6,65213935	168	3,89E-10	3,67E-09
P48037	Coatomer subunitgamma-1(Gamma-1-coat protein) (Gamma-1-COP)	Copgl Copg	COPGl_RAT	-0,3179898	0,050321967	-6,3191056	680	4,76E-10	4,46E-09
Q4AEF8	Calretinin (CR)	Calb2	CALB2_RAT	-1,05198	0,133615262	-7,8732027	41	9,92E-10	9,24E-09
P47728	V-type proton ATPase subunit E 1 (V-ATPase subunit E 1) (Vacuolar proton pumpsubunitE 1)	Atp6vlel	VATEI _RAT	-0,4939495	0,079531528	-6,2107387	480	1,14E-09	1,06E-08
Q6PCU2	ATP synthase F(O) complex subunit Bl, mitochondrial (ATP synthase subunit b) (ATPase subunit b)	Atp5f1Atp5f	AT5FI _RAT	-0,4407879	0,070470076	-6,254966	305	1,34E-09	1,23E-08
P19511	Calcyclin-binding protein (CacyBP)	Cacybp	CYBP_RAT	-0,8087168	0,104707005	-7,723617	42	1,37E-09	1,25E-08
Q6AYK6	40S ribosomal protein S9	Rps9	RS9_RAT	0,36931402	0,05707646	6,47051384	123	2,09E-09	1,89E-08
P29314	Neurotrimin {GP65)	Ntm HntNt	NTRI_RAT	-0,6142256	0,092234613	-6,6593826	86	2,47E-09	2,23E-08
Q62718	Protein disulfide-isomerase A3 (EC 5.3.4 .1) (58 kDa glucose-regulated protein) (58 kDa microsomal protein) (p58) (Disulfide isomerase ER-60) (Endoplasmic reticulum resident protein 57) (ER protein 57) (ERp57) (Endoplasmic reticulum resident protein 60) (ER protein 60) (ERp60) (HIP-70) (Q-2)	Pdia3 Erp60 Grp58	PDIA3_RA T	-0,4237018	0,035994185	-11,771396	16	2,72E-09	2,44E-08
P11598	Glycine cleavage system H protein, mitochondrial (Upoic add-containing protein)	Gcsh	GCSH_RAT	0,35858871	0,059979634	5,97850791	378	5,22E-09	4,65E-08
Q5I0P2	60S ribosomal protein L12	Rpl12	Rl 12_RA T	-3,1529802	0,424784518	-7,4225402	39	5,67E-09	5,02E-08
P23358	Tubulin beta-3 chain (Neuron-specific class III beta-tubulin)	Tubb3	TBB3_RAT	-0,4972352	0,080128809	-6,2054487	125	7,36E-09	6,48E-08
Q4QRB4	Tubulin alpha-4A chain (Alpha-tubulin 4) (Tubulin alpha-4 chain)	Tuba4a Tuba4	TBA4A_RAT	-0,9484418	0,157494931	-6,0220464	214	7,41E-09	6,48E-08
Q5XIF6	Gamma-enolase {EC 4.2.1.11) (2-phospho-0-glycerate hydro-lyase) (Enolase 2) (Neural enolase) (Neuron-specific enolase) (NSE)	Eno2 Eno-2	ENOG_RAT	-0,5336046	0,083897805	-6,3601736	88	8,75E-09	7,61E-08
P07323	Proteasome subunit beta type-10 (EC 3.4 .25.1) (Low molecular mass protein 10)(Macropain subunit MECl-1) (Multicatalytic endopeptidase complex subunit MECl-1) (Proteasome MECl-1) (Proteasome subunit beta-2i)	PsmblO LmplO Mecll	PSBl O_RA T	-0,5637037	0,096320266	-5,852389	487	8,91E-09	7,70E-08
Q4KM35	ATP-binding cassette sub-family A member 2 (ATP-binding cassette transporter 2) (ATP-binding cassette 2)	Abca2Abc2	ABCA2_RAT	0,82256912	0,060926764	13,5009489	12	1,29E-08	1,IIE-07
Q9ESR9	Thymosin beta-10	ThyblO	TYBIO_RAT	0,70925608	0,023997958	29,5548509		1,31E-08	1,12E-07
P63312	Cytochrome b-cl complex subunit Rieske, mitochondrial (EC 1.10.2.2) (Complex III subunit 5) (Cytochrome b-cl complex subunit 5) (Liver regeneration-related protein LRRGT00195) (Rieske iron-sulfur protein) (RISP) (Ubiquinol-cytochrome c reductase iron-sulfur subunit) (Cleaved into: Cytochrome b-cl complex subunit 11 (Complex III subunit IX) (Ubiquinol-cytochrome c reductase 8 kDa protein)]	Uqcrfsl	UCRI_RA T	0,76983535	0,110539687	6,96433444	43	1,45E-08	1,23E-07
CON_002203	Sideroflexin-5 (Tricarboxylate carrier BBG-TCC)	Sfxn5	SFXN5_RAT	-1,0104532	0,07572222	-13,34421	12	1,47E-08	1,24E-07
P20788	Cysteine and glycine-rich protein 1 (Cysteine-rich protein 1) (CRP) (CRPI)	CsrplCsrp	CSRPl_RAT	-0,6602071	0,085426847	-7,7283329	29	1,60E-08	1,34E-07
Q8CFDO	40S ribosomal protein S19	Rps19	RS19_RAT	-0,5918491	0,076602254	-7,7262625	28	2,04E-08	1,70E-07
P47875	Neurocan core protein (245 kDa early postnatal core glycoprotein) (Chondroitin sulfate proteoglycan 3) [Cleaved into : 150 kDa adult core glycoprotein]	Ncan Cspg3	NCAN_RAT	-1,2409175	0,208619161	-5,9482434	117	2,88E-08	2,39E-07
P17074	NADH-ubiquinone oxidoreductase 75 kDa subunit, mitochondrial (EC 1.6.5.3) (EC l. 6.99.3)	Ndufsl	NDUSI _RAT	-0,4646868	0,076891039	-6,0434449	88	3,55E-08	2,93E-07
P55067	40S ribosomal protein SA (37 kDa laminin receptor precursor) (37LRP) (37/67 kDa laminin receptor) (LRP/LR) (67 kDa laminin receptor) (67LR) (Laminin receptor 1) (LamR) (laminin-binding protein precursor p40) (LBP/p40)	Rpsa Lamrl	RSSA_RAT	-0,2851664	0,049049275	-5,8138762	144	3,78E-08	3,IOE-07
Q66HF1	3'(2'),5'-bisphosphate nucleotidase 1 (EC 3.1.3.7) (Bisphosphate 3'-nucleotidase 1) (PAP-inositol 1,4-phosphatase) (PIP) (scHAL2 analogous 3)	Bpnt1Sal3	BPNTI _RAT	-0,3815751	0,066943012	-5,6999988	182	4,76E-08	3,88E-07
P38983	Alpha-2-HS-glycoprotein (59 kDa bone sialic acid-containing protein) (BSP) (Fetuin-A) (Glycoprotein PP63)	AhsgFetua	FETUA_RAT	-0,2614391	0,043628078	-5,9924511	85	4,83E-08	3,92E-07
Q9ZIN4	NADH dehydrogenase [ubiquinone] 1 alpha subcomplex subunit 10, mitochondrial (Complex I-42kD) (Cl-42kD) (NADH-ubiquinone oxidoreductase 42 kDa subunit)	NdufalO	NDUAA_RAT	-0,5554462	0,095085188	-5,8415637	113	5,07E-08	4,09E-07
P24090	Proenkephalin-A [Cleaved into: Synenkephalin; Met-enkephalin (Opioid growth factor) (OGF); PENK(l14-133); PENK(143-185); Met-enkephalin-Arg-Gly-Leu; Leu-enkephalin; PENK(239-260); Met-enkephalin-Arg-Phe]	Penk Penk-rs Penkl	PENK_RAT	0,5542585	0,091644161	6,04794119	75	5,31E-08	4,25E-07
Q561SO	V-type proton ATPase subunit B, brain isoform (V-ATPase subunit B 2) (Endomembrane proton pump 58 kDa subunit) (Vacuolar proton pump subunit B 2)	Atp6vlb2 Atp6b2 Vat2	VATB2_RAT	-0,8127996	0,137794748	-5,8986252	91	6,18E-08	4,92E-07
P04094	Pantothenate kinase 4 (rPanK4) (EC 2.7.1.33) (Pantothenic acid kinase 4)	Pank4Fangl	PANK4_RA T	-1,393301	0,110712139	-12,584898	11	7,12E-08	5,65E-07
P62815	Bleomycin hydrolase (BH) (BLM hydrolase) {BMH) (EC 3.4 .22.40)	Blmh	BLMH_RAT	-0,4343572	0,079947103	-5,4330568	639	7,88E-08	6,18E-07
Q923S8	Beta-2-microglobulin	B2m	B2MG_RAT	0,76885104	0,099582958	7,72070903	23	7,85E-08	6,18E-07
P70645	Annexin AB (Annexin VIII) (Annexin-8)	Anxa8	ANXA8_RA T	-4,0064875	0,517621989	-7,7401803	22	1,02E-07	7,93E-07
	Allograft inflammatory factor 1 (AIF-1) (Ionized calcium-binding adapter								

P09495	T-kininogen 2 (Alpha-1-MAP) (Major acute phase protein) (T-kininogen II) (Thiostatin) [Cleaved into: T-kininogen 2 heavy chain (T-kininogen II heavy chain); T-kinin; T-kininogen 2 light chain (T-kininogen II light chain)]		KNT2_RA T	O,S611712S	0,099528589	5,63829202	81	2,43E-07	1,85E-06
Q8CFN2	Protein-arginine deiminase type-2 (EC 3.5.3.15) (Peptidylarginine deiminase II) (Protein-arginine deiminase type II)	Padi2 Pdi Pdi2	PADI2_RAT	0,48067433	0,056603691	8,49192555	16	2,54E-07	1,92E-06
P08932	Hemoglobin subunit alpha-1/2 (Alpha-1/2-globin) (Hemoglobin alpha-1/2 chain)	Hba1Hba-al	HBA_RAT	0,4913849	0,090210126	5,44711466	94	4,09E-07	3,07E-06
P20717	ATP synthase subunit d, mitochondrial (ATPase subunit d)	Atp5h Atp5jd	ATPSH_RAT	-O,S323S16	0,103060787	-5,1654139	326	4,19E-07	3,13E-06
P01946	Macrophage migration inhibitory factor (MIF) (EC 5.3.2.1) (Glutathione-binding 13 kDa protein) (L-dopachrome isomerase) (L-dopachrome tautomerase) (EC 5.3.3.12) {Phenylpyruvate tautomerase)	Mif	MIF_RAT	0,61093119	0,116291372	5,25345237	176	4,28E-07	3,18E-06
P31399	Ceruloplasmin (EC 1.16.3.1) (Ferroxidase)	Cp	CERU_RAT	-0,912219	0,165692227	-5,5055028	75	4,93E-07	3,65E-06
P30904	ATP synthase subunit O, mitochondrial (Oligomycin sensitivity conferral protein) (OSCP) (Sperm flagella protein 4)	Atp5o	ATPO_RAT	-O,S737877	0,107500982	-5,3375112	108	5,25E-07	3,87E-06
P F P13635	Peptidyl-prolyl cis-trans isomerase FKBP4 (PPlase FKBP4) (EC 5.2.1.8) (52 kDa K506-binding protein) (52 kDa FKBP) (FKBP-52) (59 kDa immunophilin) (p59) (FK506-binding protein 4) (FKBP-4) (FKBP59) (HSP-binding immunophilin) (HBI) (Immunophilin FKBP52) (Rotamase) [Cleaved into: Peptidyl-prolyl cis-trans isomerase FKBP4, N-terminally processed]	Fkbp4Fkbp52	FKBP4_RAT	0,42218167	0,081492803	5,18060072	193	5,54E-07	4,06E-06
Q06647	ER membrane protein complex subunit 2 (Tetratricopeptide repeat protein 35) (TPR repeat protein 35)	Emc2Ttc35	EMC2_RA T	-0,772761S	0,131021886	-5,8979571	42	5,57E-07	4,06E-06
Q9QVC8	SUMO-activating enzyme subunit 1 (Ubiquitin-like 1-activating enzyme EIA) [Cleaved into: SUMO-activating enzyme subunit 1, N-terminally processed]	Sae1 Ublela	SAEI _RA T	0,8876891	0,167821728	5,28947657	117	5,79E-07	4,20E-06
BOBNGO	Sideroflexin-3	Sfxn3	SFXN3_RA T	-0,3S82416	0,045157345	-7,9331851	16	6,18E-07	4,46E-06
Q6AXQO	Hyaluronan and proteoglycan link protein 2 (Brain link protein 1)	Hapln2 Bral1	HPLN2_RAT	0,47S63311	0,06781282	7,01391148	21	6,34E-07	4,55E-06
Q9JHY2	Unconventional myosin-Id (Myosin heavy chain myr 4)	Myo1d Myr4	MYOID_RAT	-O,S669C03	0,066568766	-8,5160109	14	6,57E-07	4,69E-06
Q9ESM2	40S ribosomal protein S25	Rps25	RS2S_RAT	-0,4668923	0,068190283	-6,8469029	22	7,06E-07	5,02E-06
Q63357	ATP-dependent RNA helicase DDXl (EC 3.6.4.13) (DEAD box protein 1)	Ddxl	DDXI _RAT	-0,6438024	0,05439549	-11,835584		8,66E-07	6,13E-06
P62853	Proteasome activator complex subunit 2 (11S regulator complex subunit beta) (REG-beta) {Activator of multicatalytic protease subunit 2) (Proteasome activator 28 subunit beta) {PA28b) {PA28beta)	Psme2	PSME2_RA T	-0,973409S	0,092320023	-10,543861	10	9,76E-07	6,87E-06
Q641Y8	Complement Clq subcomponent subunit C	C1qcC1qg	Cl QC_RA T	-O,S223346	0,103946361	-5,0250395	215	1,06E-06	7,41E-06
Q63798	Heat shock protein 105 kDa (Heat shock 110 kDa protein)	Hsph1 Hsp105 Hsp110	HS l OS_RA T	-1,7302079	0,167558808	-10,325974	10	1,18E-06	8,25E-06
P31722	Clathrin light chain A(Lca)	Clta	CLCA_RAT	0,362012S7	0,040844085	8,86328007	12	1,30E-06	9,00E-06
Q66HA8	Microtubule-associated protein RP/EB family member 3 (EB1 protein family member 3) (EBF3) (End-binding protein 3) (EB3) (RP3)	Mapre3	MARE3_RAT	0,3S39601S	0,072396709	4,88917456	426	1,44E-06	9,92E-06
P08081-2	0-amino-acid oxidase (DAAO) (DAMOX) (DAO) (EC 1.4.3.3)	Dao Dael	OXDA_RAT	O,S33317S9	0,072411375	7,36510787	16	1,59E-06	1,10E-05
Q5XIT1	60S ribosomal protein L7	Rpl7	RL7_RAT	-0,3498382	0,069119627	-5,0613443	113	1,63E-06	1,11E-05
035078	Quinone oxidoreductase (EC 1.6.5.5) (NADPH:quinone reductase) (Zeta-crystallin)	Cryz	QOR_RAT	-0,7300489	0,102713457	-7,1076262	17	1,76E-06	1,20E-05
P05426	Myelin proteolipid protein (PLP) (Upophilin)	PlpIPlp	MYPR_RAT	-0,4473279	0,089853095	-4,9784361	146	1,78E-06	1,21E-05
Q6AYTO	Isoaspartyl peptidase/L-asparaginase (EC 3.4.19.5) (EC 3.5.1.1) (Asparaginase-like protein 1) (Asparaginase-like sperm autoantigen) (Beta-aspartyl-peptidase) (Glial asparaginase) (Isoaspartyl dipeptidase) (L-asparagine amidohydrolase) (Cleaved into : Isoaspartyl peptidase/L-asparaginase alpha chain; Isoaspartyl peptidase/L-asparaginase beta chain]	Asrgll Alp Gliap Hiob	ASGLI_RAT	-1,10S8721	0,121886548	-9,0729628	11	1,94E-06	1,31E-05
P60203	Mitochondrial inner membrane protein (Mitofilin) (Fragment)	Immt	IMMT_RAT	-0,7708176	0,156904472	-4,9126554	172	2,08E-06	1,40E-05
Q8VI04	Peptidyl-prolyl cis-trans isomerase FKBPIA (PPlase FKBPIA) (EC 5.2.1.8) (12 kDa FK506-binding protein) (12 kDa FKBP) (FKBP-12) (FK506-binding protein 1A) (FKBP-1A) (Immunophilin FKBP12) (Rotamase)	Fkbp1a Fkbp1	FKBIA_RAT	-0,3082932	0,062871169	-4,9035698	180	2,09E-06	1,40E-05
Q3KR86	Ferritin light chain 1 (Ferritin Lsubunit 1)	FtlI Ftl	FRILI _RAT	-0,4938614	0,090286856	-5,4699147	43	2,15E-06	1,43E-05
Q62658	Serine/threonine-protein phosphatase 2A 65 kDa regulatory subunit A beta isoform (PP2A subunit A isoform PR65-beta) (PP2A subunit A isoform R1-beta)	Ppp2r1b	2AAB_RAT	0,63473S61	0,085690439	7,40730959	15	2,19E-06	1,45E-05
P02793	Cytochrome c oxidase subunit 2 (Cytochrome c oxidase polypeptide II)	Mtco2 Coii mt-Co2	COX2_RA T	0,89022732	0,180482255	4,93249222	140	2,27E-06	1,49E-05
Q4QQT4	Twinfilin-1	Twf1Ptk9	TWF1_RAT	-0,6323546	0,120767799	-5,2361192	56	2,56E-06	1,68E-05
P00406	Pyruvate kinase PKM (EC 2.7.1.40) (Pyruvate kinase muscle isozyme)	Pkm Pkm2 Pykm	KPYM_RAT	-0,6203994	0,125435864	-4,945949	113	2,66E-06	1,74E-05
Q5RJR2	Platelet-activating factor acetylhydrolase IB subunit alpha (Ussencephaly-1 protein) (LIS-1) {PAF acetylhydrolase 45 kDa subunit) (PAF-AH 45 kDa subunit) (PAF-AH alpha) (PAFAH alpha)	Pafah1bl li s-1 Us l Pafaha	LISI_RAT	0,33830288	0,068670501	4,92646595	119	2,73E-06	1,78E-05
P11980-2	Serine/threonine-protein phosphatase 2A catalytic subunit alpha isoform (PP2A-alpha) {EC 3.1.3.16)	Ppp2ca	PP2AA_RAT	0,33SS3338	0,058693456	5,7167085	31	2,75E-06	1,78E-05
P63004	Protein LZIC (Leucine zipper and CTNNBIPl domain-containing protein) (Leucine zipper and ICAT homologous domain-containing protein)	Lzic	LZIC_RAT	0,32792967	0,069140275	4,74296162	416	2,90E-06	1,87E-05
P63331	Peroxisomal multifunctional enzyme type 2 (MFE-2) (17-beta-hydroxysteroid dehydrogenase 4) (17-beta-HSD 4) (D-bifunctional protein) (DBP) (Multifunctional protein 2) (MPF-2) [Cleaved into: (3R)-hydroxyacyl-CoA dehydrogenase (EC 1.1.1.n12); Enoyl-CoA hydratase 2 (EC 4.2.1.107) (EC 4.2.1.119) (3-alpha,7-alpha,12-alpha-trihydroxy-5-beta-cholest-24-enoyl-CoA hydratase)]	Hsd17b4 Edh17b4	DHB4_RAT	-0,3482837	0,050081854	-6,9542891	16	3,25E-06	2,08E-05
Q5PQN7	60S ribosomal protein L30	Rpl30	RL30_RAT	0,2S1471SS	0,048731912	5,16030543	54	3,62E-06	2,31E-05
P97852	Ubiquitin-like modifier-activating enzyme 1 (Ubiquitin-activating enzyme El)	Ubal Ubel	UBAI _RAT	-0,394S003	0,08129992	-4,8524076	118	3,77E-06	2,40E-05
P62890	Coronin-1A (Coronin-like protein A) {Clipin-A) {Tryptophan aspartate-containing coat protein) (TACO)	Carola Corol	COR1A_RAT	-0,31278S8	0,058638455	-5,3341406	40	4,06E-06	2,57E-05
Q5U300	Misshapen-like kinase 1 (EC 2.7.11.1) (GCK family kinase MiNK) (MAPK/ERK kinase kinase kinase 6) (MEK kinase kinase 6) (MEKKK 6) (Misshapen/NIK-related kinase) (Mitogen-activated protein kinase kinase kinase kinase 6)	Minkl Map4k6 Mink	MINKI_RAT	-0,2707701	0,058526512	-4,6264523	659	4,48E-06	2,82E-05
Q91ZN1	265 protease regulatory subunit 6A (26S proteasome AAA-ATPase subunit RPT5) (Proteasome 26S subunit ATPase 3) (Spermatogenic cell/sperm-associated Tat-binding protein homolog SATA) (Tat-binding protein 1) (TBP-1)	Psmc3Tbp1	PRS6A_RAT	0,4671007S	0,095775692	4,87702818	92	4,49E-06	2,82E-05
F1LP90	Elongation factor 1-alpha2 [illegible] elongation factor1 A-2) 1	Eef1a2 Eef1al Stn Stnl	EFl A_ RAT 2	-0,4132909	0,061645801	-6,7042839	16	5,06E-06	3,16E-05
Q63569	Protein rogdi homolog	Rogdi	ROGDI_RA T	0,27400368	0,058331921	4,69731961	175	5,31E-06	3,30E-05
P62632	Glutamate dehydrogenase 1, mitochondrial (GOH 1) (EC 1.4.1.3) (Memory-related gene 2 protein) (MRG-2)	GludlGlud	DHE3_RAT	-0,9163489	0,191214392	-4,7922592	98	5,87E-06	3,64E-05
Q4V702	Serine/threonine-protein kinase PAK 2 (EC 2.7.11.1) (Gamma-PAK) (p21-activated kinase 2) (PAK-2) (Cleaved into : PAK-2p27; PAK-2p34]	Pak2	PAK2_RA T	-0,4486S8	0,056893093	-7,8859844	11	7,49E-06	4,62E-05
P10860	Guanine nucleotide-binding protein G(I)/G(S)/G(T) subunit beta-2 (G protein subunit beta-2) (Transducin beta chain 2)	Gnb2	GBB2_RAT	-0,2638404	0,058463617	-4,5128989	623	7,65E-06	4,69E-05
Q64303	Guanine nucleotide-binding protein G(I)/G(S)/G(T) subunit beta-1 (Transducin beta chain 1)	Gnbl	GBBI_RAT	0,54235444	0,106973742	5,06997722	43	8,05E-06	4,92E-05
P 54313 ; P 543	Isocitrate dehydrogenase [NAO] subunit beta, mitochondrial (EC 1.1.1.41) (Isocitric dehydrogenase subunit beta) (NAD(+)-specific ICDH subunit beta)	Idh3B	IDH3B_RAT	-0,4718S99	0,041257082	-11,437065		8,77E-06	5,34E-05
Q68FXO	Nicotinamide phosphoribosyltransferase (NAmPRTase) (Nampt) (EC 2.4.2.12) (Pre-B-cell colony-enhancing factor 1 homolog) (PBEF) (Visfatin)	NamptPbefl	Fahdl	NAMPT_RA T	-0,34SS747		0,076394735	-4,523541	
	Acylpyruvase FAHDl, mitochondrial (EC 3.7.1.5) (Fumarylacetoacetate								

Q80Z29 287	5,40E-05	hydrolase domain-containing protein 1)		FAHDI_RAT	0,4274487S	0,063295364	6,75323959	14	9,27E-06	5,60E-05
Q6AYQ8 8,91E-06		Elongation factor Tu, mitochondrial	Tufm	EFTU_RAT	-0,618S034	0,07569899	-8,1705633	10	9,78E-06	5,88E-05
P85834		Gelsolin (Actin-depolymerizing factor) (ADF) (Brevin)	Gso	GELS_RAT	-0,3196176	0,071028918	-4,4998232	235	1,07E-05	6,41E-05
Q68FP1-2		ATP synthase subunit alpha, mitochondrial	Atp5a1	ATPA_RAT	0,92314997	0,121829593	7,57738695	11	1,09E-05	6,50E-05
P15999		Mitochondrial import inner membrane translocase subunit Tim13	Timm13 Tim13a Timm13a	TIM13_ RAT	-0,3233094	0,072877909	-4,436316	516	1,12E-05	6,65E-05
P62076		Complement Clq subcomponent subunit B	Clqb	ClQB_RAT	-1,19C0681	0,109208474	-10,897213		1,21E-05	7,13E-05
P31721		Ubiquitin-like modifier-activating enzyme ATG7 (ATG12-activating enzyme El ATG7) (Autophagy-related protein 7) (APG7-like)	Atg7 Apg7l	ATG7_RAT	0,49814774	0,07792964	6,39227568	15	1,21E-05	7,13E-05
Q641Y5		Up-regulated during skeletal muscle growth protein 5 (Diabetes-associated protein in insulin-sensitive tissues)	Usmg5Dapit	USMGS_RAT	-0,3363382	0,062032002	-5,4220116	25	1,26E-05	7,37E-05
Q9JJW3		Sodium/potassium-transporting ATPase subunit beta-2 (Sodium/potassium-dependent ATPase subunit beta-2)	Atp1b2	AT1B2_RAT	-1,2138S11	0,096031945	-12,640076		1,50E-05	8,78E-05

P13638	Kinesin-1 heavy chain (Conventional kinesin heavy chain) (Ubiquitous kinesin heavy chain) (UKHC)	Kif5bKhc	KINH_R AT	-0,2528902	0,056198268	-4,4999651	122	1,56E-05	9,09E-05
Q2PQA9	Mannose-6-phosphate isomerase (EC 5.3.1.8) (Phosphohexomutase) (Phosphomannose isomerase) (PMI)	Mpi	MPI_RAT	0,26394453	0,059487165	4,4369996	174	1,61E-05	9,35E-05
Q68FX1	Endoplasmic reticulum resident protein 29 (ERp29) (Endoplasmic reticulum resident protein 31) (ERp31)	Erp29	ERP29_RAT	-0,6502673	0,118969736	-5,4658211	22	1,72E-05	9,92E-05
P52555	tRNA-splicing ligase RtcB homolog (EC 6.5.1.3) (p55)	Rtcb	RTCB_RAT	0,24153298	0,052110599	4,63500669	66	1,73E-05	9,97E-05
Q6AYT3	Long-chain specific acyl-CoA dehydrogenase, mitochondrial (LCAD) (EC 1.3.8.8)	Acadl	ACADL_RAT	-0,2587381	0,056136931	-4,609054	66	1,91E-05	0,00010912
P15650	Aldehyde dehydrogenase, mitochondrial (EC 1.2.1.3) (ALDH class 2) (ALDH-E2)(ALDH1)	Aldh2	ALDH2_RAT	-0,4742377	0,109545393	-4,3291429	214	2,30E-05	0,00013112
P11884	Septin-2 (Vascular endothelial cell specific protein 11)	Sept2 Vespll	SEPT2_RA T	-0,4095897	0,096154474	-4,2597051	328	2,68E-05	0,0001521
Q91Y81	Thymosin beta-4 (T beta 4) [Cleaved into: Hematopoietic system regulatory peptide(Seraspenide)]	Tmsb4x Thyb4 Tmsb4	TYB4_RAT	-0,3825182	0,085600428	-4,4686479	75	2,74E-05	0,00015513
P62329	Serine/threonine-protein phosphatase 6 catalytic subunit (PP6C) (EC 3.1.3 .16) (Protein phosphatase V) (PP-V) (Cleaved into: Serine/threonine-protein phosphatase 6 catalytic subunit. N-terminally processed]	Ppp6c Ppv	PPP6_RAT	0,83886	0,193734417	4,32994826	143	2,79E-05	0,00015721
Q64620	Uncharacterized protein C12orf43 homolog		Cl043_RAT	0,32612537	0,067218868	4,85169392	33	2,85E-05	0,00016023
Q5I034	Aspartate aminotransferase. cytoplasmic (cAspAT) (EC 2.6 .1.1) (EC 2.6.1.3) (Cysteine aminotransferase, cytoplasmic) (Cysteine transaminase, cytoplasmic) (cCAT) (Glutamate oxaloacetate transaminase 1) (Transaminase A)	Gotl	AATC_RAT	0,42254128	0,065282944	6,47246047	12	3,06E-05	0,00017101
P13221	Unconventional myosin-Va (Dilute myosin heavy chain, non-muscle)	Myo5a	MY05A_RAT	-0,3709406	0,088406875	-4,1958338	596	3,13E-05	0,00017454
Q9QYF3	Cyclin-dependent kinase 5 (EC 2.7.11.22) (Cell division protein kinase 5) (Serine/threonine-protein kinase PSSALRE) (Tau protein kinase II catalytic subunit)(TPKII catalytic subunit)	Cdk5 Cdkn5	CDK5_RAT	-0,320939	0,075300619	-4,2621035	172	3,33E-05	0,00018485
CON_007152	Fatty acid-binding protein. brain (Brain lipid-binding protein) (BLBP) (Brain-type fatty acid-binding protein) (B-FABP) (Fatty acid-binding protein 7)	Fabp7 Blbp	FABP7_RAT	0,47931201	0,07170741	6,68427453	11	3,45E-05	0,00019064
Q03114	Sodium- and chloride-dependent GABA transporter 3 (GAT-3) (Solute carrier family 6 member 11)	Slc6all Gabt3 Gat-3 Gat-b	S6AII _RAT	-0,4387573	0,101487035	-4,3232842	99	3,67E-05	0,00020199
P55051	Alpha-2-macroglobulin (Alpha-2-M)	A2mA2ml	A2MG_RAT	0,45742732	0,106865877	4,28038714	119	3,80E-05	0,0002083
P31647	Galectin-3-binding protein (Cyp-C-associated protein) (CyCAP) (Lectin galactoside-binding soluble 3-binding protein) (Mac-2-binding protein) (MAC2BP) (Mac-2 BP) (Protein 90K)	Igals3bp	LG3BP_RAT	-0,4861173	0,114829632	-4,2333785	152	3,97E-05	0,00021694
P06238	Sodium/potassium-transporting ATPase subunit alpha-3 (Na(+)/K(+) ATPase alpha-3 subunit) (EC 3.6.3.9) (Na(+)/K(+) ATPase alpha(III) subunit) (Sodium pumpsubunitalpha-3)	Atpla3	AT1A3_RAT	-0,276051	0,045838394	-6,0222664	13	4,29E-05	0,00023348
070513	Serotransferrin (Transferrin) (Beta-1 metal-binding globulin) (Liver regeneration-related protein LRRG03) (Siderophilin)	Tf	TRFE_RAT	0,92212516	0,148017059	6,22985733	12	4,39E-05	0,00023788
P06687	Protein kinase C and casein kinase substrate in neurons protein 1 (Dynamin praline-rich domain-interacting protein) (Dynamin PRO-interacting protein) (Synaptic, dynamin-associated protein I) (Syndapin-1) (Syndapin-1) (Sdpl)	Pacsinl	PACNI _RAT	-0,25708	0,062631459	-4,1046468	815	4,46E-05	0,00023998
P12346	Adenylate kinase isoenzyme 1 (AK 1) (EC 2.7 .4.3) (EC 2.7.4.6) (ATP-AMP transphosphorylase 1) (ATP:AMP phosphotransferase) (Adenylate monophosphate kinase) (Myokinase)	Akl	KADI _RAT	0,75814143	0,18017439	4,20782018	148	4,45E-05	0,00023998
Q9ZOW5	T-kininogen 1 (Alpha-1-MAP) (Major acute phase protein) (T-kininogen I) (Thiostatin) (Cleaved into: T-kininogen 1 heavy chain (T-kininogen I heavy chain); T-kinin; T-kininogen 1 light chain (T-kininogen I light chain)]	Mapl	KNTI _RAT	-0,3665903	0,088494422	-4,1425248	282	4,54E-05	0,00024378
P39069	N-acetylaspartate synthetase (NAA synthetase) (N-acetyltransferase 8-like protein) (EC2.3.1.17)	Nat8l	NAT8L_RAT	-0,4561675	0,110235846	-4,1381053	281	4,63E-05	0,00024713
P54311	Dolichyl-diphosphooligosaccharide-protein glycosyltransferase subunit 1 (EC 2.4.99.18) (Dolichyl-diphosphooligosaccharide--protein glycosyltransferase 67 kOa subunit) (Ribophorin I) (RPN-I) (Ribophorin-1)	Rpnl	RPN I _RAT	-0,4055431	0,09715675	-4,1741114	181	4,64E-05	0,00024713
P01048	14-3-3 protein theta (14-3-3 protein tau)	Ywhaq	1433T_RAT	1,21335073	0,25493285	4,75949148	29	4,95E-05	0,00026186
D3ZVU9	CDGSH iron-sulfur domain-containing protein 1 (MitoNEET)	Cisdl	CISDI _RAT	-0,4201157	0,074860959	-5,6119468	15	4,95E-05	0,00026186
P07153	Brevican core protein (Brain-enriched hyaluronan-binding protein) (BEHAB)	Bean Behab	PGCB_RAT	-0,2716141	0,063873991	-4,2523428	88	5,26E-05	0,00027677
P68255	40S ribosomal protein S4, X isoform	Rps4xRps4	RS4X_RAT	0,55504297	0,130384319	4,25697636	85	5,31E-05	0,00027889
BOK020	Electrogenic sodium bicarbonate cotransporter 1 (Sodium bicarbonate cotransporter) (NBC-like protein) (Na(+)/HC03(-) cotransporter) (Solute carrier family 4 member 4)	Slc4a4 Nbc Nbcl Nbcel Rnbcl	S4A4_RAT	-0,4378242	0,09802014	-4,4666757	42	5,89E-05	0,00030797
P55068	Atlastin-1 (EC 3.6.5.-) (Spastic paraplegia 3A homolog)	Atl1Spg3a	ATLAI _RAT	-0,3444747	0,083471261	-4,1268655	136	6,38E-05	0,00033212
P62703	V-type proton ATPase 16 kDa proteolipid subunit (V-ATPase 16 kDa proteolipid subunit) (Vacuolar proton pump 16 kDa proteolipid subunit)	Atp6v0c Atp6c Atp6l Atpl	VATL_RAT	-0,3660936	0,089371246	-4,096324	158	6,69E-05	0,00034711
Q9JI66-3	Large neutral amino acids transporter small subunit 1 (4F2 light chain) (4F2 LC) (4F2LC) (Integral membrane protein E16) (Protein TAI) (L-type amino acid transporter 1) (Solute carrier family 7 member 5)	Slc7a5 Mpe16Tal	LATI_RAT	-0,243383	0,046058424	-5,2842234	16	7,41E-05	0,00038334
Q6PST4	Copine-9 (Copine IX)	Cpne9	CPNE9_RAT	-0,7438464	0,182034192	-4,0863007	122	7,88E-05	0,00040619
P63081	ZWIO interactor (SNAP25-interacting protein 30) (Scoilin) (ZWIO-interacting protein 1) (Zwint-1)	ZwintSip30	ZWINT_RA T	-0,6174351	0,136961102	-4,5081053	31	8,75E-05	0,00044925
Q63016	26S proteasome non-ATPase regulatory subunit 2	Psmd2	PSMD2_RAT	0,3937554	0,058713557	6,70637951		8,79E-05	0,00044977
Q5BJS7	Ras GTPase-activating protein 1 (GAP) (GTPase-activating protein) (RasGAP) (Ras p21 protein activator) (p120GAP)	Rasal Rasa	RASAI_RAT	-0,2671535	0,051586636	-5,1787339	16	9,14E-05	0,00046618
Q8VII3	Arfaptin-2 (ADP-ribosylation factor-interacting protein 2)	Arfip2	ARFP2_RAT	0,46167121	0,108849067	4,24138874	51	9,36E-05	0,00047549
Q4FZT9	Regulating synaptic membrane exocytosis protein 3 (Nim3) (RIM3 gamma) (Rab-3-interacting molecule 3) (RIM 3)	Rims3Nim3	RIMS3_RAT	0,27906456	0,070417119	3,9630215	269	9,49E-05	0,00048039
P50904	Rab GTPase-binding effector protein 1 (Rabaptin-5) (Rabaptin-5alpha)	Rabepl Rabpt5 Rabpt5a	RABEI _RA T	0,5082693	0,10800426	4,70601161	23	9,69E-05	0,00048868
Q6AY65	Lanosterol 14-alpha demethylase (LDM) (EC 1.14.13.70) (CYPLI) (Cytochrome P450 51AI) (Cytochrome P450-14DM) (Cytochrome P45014DM) (Cytochrome P450LI) (Sterol 14-alpha demethylase)	Cyp51a1Cyp51	CP51A_RA T	-0,6914293	0,126251814	-5,4765889	13	0,0001063	0,00053444
Q9JIR3	60S ribosomal protein L32	Rpl32	RL32_RAT	1,1453551	0,103953173	11,0179907	5	0,0001072	0,00053713
035550	Adenosine kinase (AK) (EC 2.7.1.20) (Adenosine 5'-phosphotransferase)	Adk	ADK_RA T	0,25762716	0,061701376	4,17538758	54	0,00010909	0,00054467
Q64654	5-oxoprolinase (EC 3.5.2.9) (5-oxo-L-prolinase) (5-0Pase) (Pyroglutamase)	Oplah	OPLA_RAT	-0,7802747	0,127272051	-6,1307623	10	0,0001111	0,0005528
P62912	Protein SIOO-AI (S-100 protein alpha chain) (S-100 protein subunit alpha) (SIOO calcium-binding protein AI)	SIOOaI	SI OAI _RAT	-0,4715861	0,092881297	-5,0772984	16	0,00011201	0,00055545
Q64640	Alpha-enolase (EC4.2.1.11) (2-phospho-0-glycerate hydro-lyase) (Enolase 1) (Non-neural enolase) (NNE)	Enol Eno-1	ENOA_RAT	-0,3007396	0,075203291	-3,9990221	106	0,00011792	0,00058247
P97608	Myelin protein PO (Myelin peripheral protein) (MPP) (Myelin protein zero)	MpzPO	MYPO_RAT	0,30034064	0,064350714	4,66724644	22	0,00011827	0,00058247
P35467	Plastin-3 (T-plastin)	Pls3	PLST_RAT	-0,9023133	0,219765815	-4,1057946	62	0,0001199	0,00058851
P04764	Diphosphoinositol polyphosphate phosphohydrolase 1 (DIPP-1) (EC 3.6.1.52) (Diadenosine 5',5"'-PI,P6-hexaphosphate hydrolase 1) (EC 3.6.1.-) (Nucleoside diphosphate-linked moiety X motif 3) (Nudix motif 3)	Nudt3 Dipp Dippl	NUDT3_RA T	0,28351728	0,073267973	3,86959366	425	0,00012613	0,00061697
P06907	DNA-(apurinic or apyrimidinic site) lyase (EC 3.1.-.-) (EC 4.2.99.18) (APEX nuclease) (APEN) (Apurinic-apyrimidinic endonuclease 1) (AP endonuclease 1)(REF-1) (Redox factor-1) [Cleaved into: DNA-(apurinic or apyrimidinic site) lyase, mitochondrial)	Apexl Ape Apex Ref	APEXI _RAT	-0,6243591	0,15437611	-4,0444025	74	0,00012737	0,00062091
Q63598	Methylmalonate-semialdehyde dehydrogenase (acylating], mitochondrial (MMSDH) (Malonate-semialdehyde dehydrogenase (acylating]) (EC 1.2.1.18) (EC 1.2.1.27) (Aldehyde dehydrogenase family 6 member AI)	Aldh6a1Mmsdh	MM SA_RA T	-0,2917243	0,075480281	-3,8649072	327	0,00013404	0,00065123
Q566C7	Glutathione S-transferase kappa 1 (EC 2.5.1.18) (GST 13-13) (GST class-kapp a) (GSTKI-1) (rGSTKI) (Glutathione S-transferase subunit 13)	Gstkl	GSTKI_RAT	0,25026124	0,061713773	4,05519266	64	0,00013837	0,00067004
P43138	CTP synthase 2 (EC 6.3.4.2) (CTP synthetase 2) (UTP--ammonia ligase 2)	Ctps2	PYRG2_RAT	0,44950903	0,071575897	6,28017326	9	0,00014435	0,00069663
Q02253	Protein arginine N-methyltransferase 1 (EC 2.1.1.-) (Histone-arginine N-methyltransferase PRMTI) (EC 2.1.1.125)	Prmt1Hrmtll2	ANMI_RAT	-0,3269657	0,084454115	-3,8715192	179	0,00015144	0,00072843
P24473	ATP synthase subunit delta, mitochondrial (F-ATPase delta subunit)	Atp5d	ATPD_RAT	-0,3676332	0,082068063	-4,4796139	23	0,00017031	0,00081649
Q5U2NO	Dihydrolipoyl dehydrogenase, mitochondrial (EC 1.8.1.4) (Dihydrolipoamide dehydrogenase)	Dld	DLDH_RAT	-0,3892254	0,053890005	-7,2225901	7	0,00017401	0,00083145
Q63009	60S acidic ribosomal protein Pl	Rplpl	RLAI_RAT	0,26362632	0,06781824	3,88724809	101	0,00018155	0,00086459
P35434	Sorbitol dehydrogenase (EC 1.1.1.14) (L-iditol 2-dehydrogenase)	Sord Sdhl	DHSO_RA T	-0,3342786	0,082376667	-4,0579286	42	0,00021069	0,00100006
Q6P6R2	Peptidyl-prolyl cis-trans isomerase D (PPlase D) (EC 5.2.1.8) (40 kDa peptidyl-prolyl cis-trans isomerase) (Cyclophilin-40) (CYP-40) (Rotamase D)	Ppid	PPID_RAT	-0,2684027	0,071275372	-3,7657139	223	0,00021248	0,00100529
P19944	60S ribosomal protein L23a	Rpl23a	RL23A_RAT	-0,5336663	0,129386932	-4,1245768	35	0,0002174	0,00102518
P27867	Four and a half LIM domains protein 1 (FHL-1)	Fhll	FHLI _RAT	0,22093118	0,057437388	3,84646981	88	0,00022631	0,00106371

Q6DGGO	Myosin-10 (Cellular myosin heavy chain, type B) (Myosin heavy chain 10) (Myosin heavy chain, non-muscle llb) (Non-muscle myosin heavy chain B) (NMMHC-B) (Non-muscle myosin heavy chain llb) {NMMHC 11-b) (NMMHC-IIB)	MyhlO	MYHl O_RA T	0,2558769	0,068852488	3,71630583	347	0,00023557	0,00110364
P62752	Myotrophin (Granule cell differentiation protein) (Protein V-1)	MtpnGcdp	MTPN_RAT	-0,4717416	0,119629102	-3,9433678	53	0,00023727	0,00110801
Q9WUH4	60S ribosomal protein L18a	Rpl18a	RL18A_RAT	0,46832403	0,050499386	9,27385583	5	0,00024511	0,00114096
Q9JLTO	Potassium voltage-gated channel subfamily C member 3 (KSHIIID) (Voltage-gated potassium channel subunit Kv3.3)	Kcnc3	KCNC3_RA T	-0,2018991	0,055005482	-3,6705263	561	0,00026517	0,00123034
P62775	Vacuolar protein sorting-associated protein 45 (rVps45)	Vps45Vps45a	VPS45_RAT	0,28679322	0,061672657	4,65024912	16	0,00026673	0,00123362
P62718	UPF0587 protein Clorf123 homolog		CA123_RAT	-0,3001662	0,078656515	-3,8161645	67	0,00029823	0,00137489
Q01956	Histone-binding protein RBBP7 (Nucleosome-remodeling factor subunit RBAP46) (Retinoblastoma-binding protein 7) (RBBP-7)	Rbbp7	RBBP7_RAT	-0,3682005	0,064808412	-5,6813682	9	0,00030134	0,00138483
008700	60S ribosomal protein L9	Rpl9	RL9_RAT	-0,398315	0,100598392	-3,9594565	37	0,00032847	0,00150471
Q498R7	8-oxo-dGDP phosphatase NUDT18 (EC 3.6.1.58) (2-hydroxy-dADP phosphatase) (7,8-dihydro-8-oxoguanine phosphatase) {MutT homolog 3) (Nucleoside diphosphate-linked moiety X motif 18) (Nudix motif 18)	Nudt18Mth3	NUD18_RA T	0,33667605	0,083132659	4,04986507	30	0,00033288	0,0015201
Q71UF4	Tubulin beta-2A chain	Tubb2a	TBB2A_RA T	0,30796567	0,080055352	3,84690912	51	0,00033399	0,00152034
P17077	Trafficking protein particle complex subunit 3	Trappc3	TPPC3_RAT	-0,4434852	0,118506445	-3,7422879	81	0,00033943	0,00154022
Q641Y7	Transcription elongation factor B polypeptide 2 (Elongin 18 kDa subunit) (Elongin-B) (EloB) (RNA polymerase II transcription factor SIii subunit B) (SIii p18)	Tceb2	ELOB_RAT	0,3489863	0,076040503	4,58947904	15	0,0003543	0,00160268
P85108	Succinyl-CoA ligase [ADP/GDP-forming] subunit alpha, mitochondrial (EC 6.2.1.4) (EC 6.2.1.5) (Succinyl-CoA synthetase subunit alpha) (SCS-alpha)	Suclgl	SUCA_RAT	-0,2974669	0,064981186	-4,5777386	15	0,00036265	0,00163533
QSU122	Cell adhesion molecule 3 (Immunoglobulin superfamily member 4B) (lgSF48) (Nectin-like protein 1) (NECL-1)	Cadm3 lgsf4b Necll	CADM3_RA T	0,25465867	0,064880355	3,92505049	36	0,00037498	0,00168565
P62870	Peptidyl-prolyl cis-trans isomerase A (PPlase A) (EC 5.2.1.8) (Cyclophilin A) (Cyclosporin A-binding protein) (Rotamase A) (p1B15) (p31) (Cleaved into: Peptidyl-prolyl cis-trans isomerase A, N-terminally processed]	Ppia	PPIA_RAT	0,29034002	0,076329967	3,80374884	51	0,00038261	0,00170403
P13086	Sortilin (Glycoprotein 110) (GpllO) (Neurotensin receptor 3) {NTR3)	Sortl	SORT_RA T	-0,5645863	0,154000482	-3,6661336	109	0,00038229	0,00170403
Q1WIM3	Nucleosome assembly protein 1-like 4	Napll4	NP1L4_RAT	-0,258496	0,06706918	-3,854169	43	0,00038252	0,00170403
P10111	Vitamin D-binding protein (DBP) (VDB) (Ge-globulin) (Group-specific component)	GcDbp	VTDB_RAT	0,42082447	0,117692778	3,57561846	327	0,00040211	0,00178539
054861	Guanine nucleotide-binding protein subunit beta-5 (GbetaS) (Transducin beta chains)	GnbS	GBBS_RAT	0,47817804	0,115838074	4,12798681	23	0,00040922	0,00181135
QSU223	Thiosulfate sulfurtransferase (EC 2.8.1.1) (Rhodanese)	Tst	THTR_RAT	-0,4275949	0,119390632	-3,5814777	152	0,00045925	0,00202662
P04276	Clusterin (Dimeric acid glycoprotein) (DAG) (Sulfated glycoprotein 2) (SGP-2) (Testosterone repressed prostate message 2) (TRPM-2) (Cleaved into: Clusterin beta chain; Clusterinalphachain]	Clo	CLUS_RAT	0,79473625	0,129246469	6,14899778		0,000468	0,00205891
P62882	Oxidation resistance protein 1 (Protein C7)	Oxrl C7	OXR1_RAT	-0,3144408	0,083146801	-3,7817545	42	0,00048663	0,00213436
P24329	Spermatogenesis-defective protein 39 homolog (hSPE-39) (VPS33B-interacting protein in apical-basolateral polarity regulator) (VPS338-interacting protein in polarity and apical restriction)	Vipas39Spe39Vipar	SPE39_RA T	-0,2192506	0,061605674	-3,5589353	153	0,00049629	0,00217014
P05371	Bridging integrator 2	Bin2	BIN2_RAT	0,58467419	0,162215343	3,6043088	87	0,00052087	0,00227074
Q4V8BO	60S ribosomal protein LIOa	RpllOa	Rl l OA_RAT	0,23669272	0,067155278	3,52455866	200	0,00052575	0,00227825
QSPQN6	Aflatoxin BI aldehyde reductase member 2 (rAFAR2) (EC 1.1.1.nll) (Succinic semialdehyde reductase) (SSA reductase)	Akr7a2Afar2Aiar	ARK72_RAT	-0,7762232	0,160622887	-4,8325813	11	0,00052519	0,00227825
Q68FR2	Aldose 1-epimerase (EC 5.1.3.3) (Galactose mutarotase)	Galm	GALM_RAT	0,54231347	0,144088607	3,76374982	40	0,00053774	0,00232323
P62907	Prostaglandin reductase 2 (PRG-2) (EC 1.3.1.48) (15-oxoprostaglandin 13-reductase) (Zinc-binding alcohol dehydrogenase domain-containing protein 1)	Ptgr2Zadhl	PTGR2_RAT	-0,4704481	0,127586597	-3,687285	52	0,00054214	0,00233526
Q8CG4S	DnaJ homolog subfamily A member 2 (RDJ2)	Dnaja2	DNJA2_RA T	-0,3269618	0,091703135	-3,565437	107	0,00054453	0,00233854
Q66HG4	Rho guanine nucleotide exchange factor 7 (Beta-Pix) (PAK-interacting exchange factor beta)	Arhgef7 Pak3bpPixb	ARH G7_RA T	0,26421884	0,072609221	3,63891573	62	0,00055936	0,00238802
QSBK81	Aldose reductase (AR) (EC 1.1.1.21) (Aldehyde reductase)	Akrlbl Akrlb4 Aldrl	ALDR_RAT	0,31869675	0,074242603	4,29263971	16	0,00055917	0,00238802
035824	265 protease regulatory subunit 7 (26S proteasome AAA-ATPase subunit RPTI) (Proteasome 26S subunit ATPase 2) (Protein MSSI)	Psmc2Mssl	PRS7_RAT	-0,6026862	0,16871146	-3,5722894	80	0,00060279	0,00256587
055043	Clathrin light chain B(Lcb)	Cltb	CLCB_RAT	-0,4457654	0,094103749	-4,7369573	11	0,00061244	0,00259927
P07943	3-hydroxyacyl-CoA dehydrogenase type-2 (EC 1.1.1.35) (17-beta-hydroxysteroid dehydrogenase 10) (17-beta-HSD 10) (EC 1.1.1.51) (3-hydroxy-2-methylbutyryl-CoA dehydrogenase) (EC 1.1.1.178) (3-hydroxyacyl-CoA dehydrogenase type II) (Endoplasmic reticulum-associated amyloid beta-peptide-binding protein) (Mitochondrial ribonuclease P protein 2) (Mitochondrial RNase P protein 2) (Type II HADH)	Hsd17b10 Erab Hadh2	HCD2_RAT	-0,2793495	0,080940438	-3,4512968	341	0,00062802	0,00265759
Q63347	Tubulin beta-2B chain (T beta-15)	Tubb2b Tubb2a	TBB2B_RAT	0,25073547	0,073052421	3,43226773	245	0,00070249	0,00296403
P08082	T-complex protein 1 subunit gamma (TCP-1-gamma) (CCT-gamma)	Cct3	TCPG_RAT	0,25421841	0,060833522	4,1789198	16	0,00070908	0,0029831
070351	Sortingnexin-5	Snx5	SNXS_RA T	-0,2993141	0,084823909	-3,5286528	75	0,00071723	0,00300573
Q3KRE8	Serine--tRNA ligase. cytoplasmic (EC 6.1.1.11) (Seryl-tRNA synthetase) (SerRS) (Seryl-tRNA(Ser/Sec)synthetase)	SarsSarsl	SYSC_RAT	-0,3208946	0,076906477	-4,1725302	16	0,00071863	0,00300573
Q6P502	lg kappa chain C region, B allele		KACB_RAT	-0,254553	0,075018684	-3,3931943	350	0,00076995	0,00321109
B1H267	Solute carrier family 15 member 2 (Kidney H(+)/peptide cotransporter) (Oligopeptide transporter, kidney isoform) (Peptide transporter 2)	Slc15a2 Pept2	S15A2_RA T	0,24896948	0,066339357	3,752968	27	0,00084759	0,0035247
Q6P799	LETMI and EF-hand domain-containing protein 1, mitochondrial (Leucine zipper-EF-hand-containing transmembrane protein 1)	Letml	LETM l _RA T	0,26758832	0,079482235	3,36664315	284	0,00086585	0,00359031
P01835	Ectonucleotide pyrophosphatase/phosphodiesterase family member 6 {E-NPP 6) (NPP-6) (EC 3.1.4.-) (EC 3.1.4.38) (Choline-specific glycerophosphodiester phosphodiesterase) (Glycerophosphocholine cholinephosphodiesterase) (GPC-Cpde)	Enpp6	ENPP6_RAT	-0,3441193	0,084333983	-4,0804344	16	0,0008716	0,0036038
Q63424	Cell cycle exit and neuronal differentiation protein 1	Cendl	CEND_RAT	-0,6353679	0,123405784	-5,1486072	8	0,00087582	0,00360637
QSXIN6	Sentrin-specific protease 8 (EC 3.4.22.68) (Deneddylase-1) (NEDD8-specific protease 1) {Sentrin/SUMO-specific protease SENP8)	Senp8	SENP8_RA T	-0,3057321	0,083041482	-3,6816797	31	0,00087722	0,00360637
BOBNDO	Secretory carrier-associated membrane protein 1 (Secretory carrier membrane protein 1) {SCAMP 37)	ScamplScamp	SCAM1_RAT	-0,5549819	0,163608309	-3,3921373	136	0,00090847	0,00371367
QSFVl4	60S ribosomal protein L13	Rpl13	RL13_RAT	-0,437912	0,125298569	-3,494948	59	0,00090655	0,00371367
QSFVJ8	Reticulocalbin-2 {Calcium-binding protein ERC-55) (Taipoxin-associated calcium-binding protein 49) {TCBP-49)	Rcn2Erc55	RCN2_RA T	1,39025245	0,159003812	8,74351645	4	0,00094287	0,0038434
P56603	ATP synthase protein 8 (AGL) (Chargerin II) (F-ATPase subunit 8)	Mt-atp8 Atp8 Atpase8 Mtatp8	ATP8_RAT	-0,7668975	0,215494138	-3,558786	40	0,00097771	0,00397417
P41123	Beta-glucuronidase {EC 3.2.1.31)	GusbGus	BGLR_RAT	-0,2971212	0,087855053	-3,3819483	96	0,00104261	0,00422608
Q62703	Mitochondrial import receptor subunit TOM70 (Mitochondrial precursor proteins import receptor) (Translocase of outer membrane 70 kDa subunit)	Tomm70a	TOM70_ RA T	-0,4764916	0,141690252	-3,3629101	103	0,0010837	0,00438035
P11608	NADH dehydrogenase [ubiquinone] 1 alpha subcomplex subunit 5 (Complex I subunit B13) {Complex l-13kD-B) {Cl-13kD-B) (NADH-ubiquinone oxidoreductase 13 kDa-B subunit)	NdufaS	NDUA5_RAT	-0,9119703	0,184005062	-4,9562241	8	0,00111216	0,0044828
P06760	40S ribosomal protein S18	Rps18	RS18_RAT	-0,4204698	0,085254847	-4,9319168	8	0,00114667	0,00460903
Q75Q39	Serine/threonine-protein phosphatase 2A catalytic subunit beta isoform (PP2A-beta) {EC 3.1.3.16)	Ppp2cb	PP2AB_RA T	-0,5965894	0,17167854	-3,4750379	44	0,00116025	0,00465065
Q63362	Serine/threonine-protein phosphatase CPPEDl (EC 3.1.3.16) (Calcineurin-like phosphoesterase domain-containing protein 1)	Cppedl Cstpl	CPPED_RAT	-0,4882408	0,120359836	-4,0565096	14	0,00117815	0,00470934
P62271	Charged multivesicular body protein 5 (Chromatin-modifying protein 5)	ChmpS	CHMPS_RA T	-0,3053613	0,08996534	-3,3942103	56	0,00127048	0,00506438
P62716	265 protease regulatory subunit 4 (P26s4) (26S proteasome AAA-ATPase subunit RPT2) {Proteasome 26S subunit ATPase 1)	Psmcl	PRS4_RAT	-0,2041188	0,052815249	-3,86477	16	0,00137193	0,00545372
Q66H71	Bis(S'-nucleosyl)-tetraphosphatase [asymmetrical] (EC 3.6.1.17) (Diadenosine 5',5'''-Pl,P4-tetraphosphate asymmetrical hydrolase) (Ap4A hydrolase) (Ap4Aase) (Diadenosine tetraphosphatase) (Nucleoside diphosphate-linked moiety X motif 2) (Nudix motif 2)	Nudt2	AP4A_RAT	0,27803647	0,080073341	3,47227261	35	0,00139161	0,00551674
CON_000213	40S ribosomal protein S12	Rps12	RS12_RAT	0,61464013	0,173676778	3,53898858	28	0,00142425	0,00561527
Q4QQV8	Stress-70 protein, mitochondrial (75 kDa glucose-regulated protein) (GRP-75) (Heat shock 70 kDa protein 9) (Mortalin) (Peptide-binding protein 74) (PBP74) {mtHSP70)	Hspa9 Grp75 Hspa9a	GRP75_RAT	0,3760194	0,112126171	3,35353824	57	0,001423	0,00561527
P62193	26S protease regulatory subunit GB (26S proteasome AAA-ATPase subunit RPT3) (Proteasome 26S subunit ATPase 4) (S6 ATPase) (Tat-binding protein 7) (TBP-7)	Psmc4 Tbp7	PRS6B_RAT	0,29759467	0,09160468	3,24868411	147	0,00143685	0,00564953
Q6PECO	Glycerophosphodiester phosphodiesterase domain-containing protein 1 {EC 3.1.-.-)	Gdpdl	GDPD1_RAT	-0,7411204	0,225465772	-3,2870639	88	0,00145556	0,00570755

P63324	Sterol -4-alpha-carboxylate 3-dehydrogenase, decarboxylating (EC 1.1.1.170)	Nsdhl	NSDHL_RAT	-0,2564212	0,078603423	-3,2622147	111	0,00146908	0,00574493
P48721	Prelamin-A/C [Cleaved into: Lamin-A/C]	Lmnalmnl	LMNA_RAT	-0,1250884	0,039278295	-3,1846705	391	0,00156536	0,0060558
Q63570	Nischarin (Imidazoline receptor 1) (1-1) (IRI) (Imidazoline-1receptor) (IIR)	Nisch	NISCH_RAT	0,30242708	0,093322051	3,24068186	115	0,00155986	0,0060558
QOVGK4	Myelin-oligodendrocyte glycoprotein	Mog	MOG_RAT	-0,3841951	0,061687083	-6,2281287	5	0,00156122	0,0060558
Q5PPL3	Acidic leucine-rich nuclear phosphoprotein 32 family member E	Anp32e	AN32E_RA T	-0,353238	0,086822787	-4,0684938	12	0,00155781	0,0060558
P48679	Aromatic-L-amino-acid decarboxylase (AADC) (EC4.1.1.28) (DOPA decarboxylase) (DOC)	Ddc	DDC_RAT	0,52572402	0,086613449	6,06977358	5	0,00175294	0,00676334
Q4G017	Glutathione S-transferase Yb-3 (EC 2.5.1.18) (Chain 4) (GST Yb3) (GST class-mu3)	Gstm3	GSTM4_RAT	-0,7829C02	0,23381089	-3,3484335	40	0,00178005	0,00684964
Q63345	Calnexin	Canx	CALX_RAT	-0,316049	0,097336295	-3,2469801	66	0,00183462	0,00704083
Q5XIEO	Tyrosine-protein phosphatase non-receptor type 23 (EC 3.1.3.48) (His domain-containing protein tyrosine phosphatase) (HD-PTP) (Protein tyrosine phosphatase TD14) {PTP-TD14) (Fragment)	Ptpn23	PTN23_RA T	-0,4733819	0,089546706	-5,2864246	6	0,00185377	0,00709545
P14173	Enoyl-CoA delta isomerase 1, mitochondrial (EC 5.3.3 .8) (3,2-trans-enoyl-CoA isomerase) (Delta(3),Delta(2)-enoyl-CoA isomerase) (D3,D2-enoyl-CoA isomerase) {Dodecenoyl-CoA isomerase)	Ecil Dci	ECIl _RAT	0,20384272	0,061918886	3,29209276	48	0,00187043	0,00714028
P08009	Protein phosphatase 1 regulatory subunit 7 (Protein phosphatase 1 regulatorysubunit22)	Ppplr7Sds22	PP1R7_RAT	0,34789563	0,11023257	3,15601488	155	0,00192203	0,0073179
P35565	Neurosecretory protein VGF (VGF8a protein) (Cleaved into: VGF(24-63); VGF(180-194); VGF(375-407); Neuroendocrine regulatory peptide-I {NERP-1); Neuroendocrine regulatory peptide-2 (NERP-2); TLQP-11; TLQP-21; TLQP-30; TLQP-62; HFHH-10; AQEE-30; LQEQ-19]	Vgf	VGF_RAT	-0,3224253	0,103001739	-3,1302897	277	0,00193303	0,00734042
088902	Nucleosome assembly protein 1-like 1 (NAP-I-related protein)	Naplll Nrp	NPll l _RAT	0,34582502	0,106170558	3,25725912	54	0,00194721	0,00735555
P23965	60S ribosomal protein L8	Rpl8	RL8_RAT	-0,3299907	0,097163815	-3,39623	30	0,00194277	0,00735555
Q5HZV9	Myeloid-associated differentiation marker (Myeloid up-regulated protein)	Myadm	MYADM_RAT	0,17452245	0,055946525	3,11945108	270	0,00200813	0,00750708
P20156	Small ubiquitin-related modifier 1 (SUM0-1)	Sumol	SUMOl _RAT	0,48144804	0,142718128	3,37341894	31	0,00200786	0,00750708
Q9Z2G8	Isocitrate dehydrogenase [NAD] subunit alpha, mitochondrial (EC 1.1.1.41) (Isocitric dehydrogenase subunit alpha) (NAD(+)-specific ICDH subunit alpha)	Idh3a	IDH3A_RAT	-0,5193559	0,153391989	-3,3858084	30	0,00199673	0,00750708
P62919	Dihydropyrimidinase-related protein 4 (DRP-4) (Collapsin response mediator protein 3) {CRMP-3) (UNC33-like phosphoprotein4) (ULIP-4) (Fragment)	Dpysl4 Crmp3 Ulip4	DPYL4_RAT	-0,6535821	0,203244485	-3,2157434	67	0,00200387	0,00750708
Q6VBQ5	Myoglobin	Mb	MYG_RAT	-0,553137	0,158970715	-3,4794897	23	0,00202684	0,00755744
Q5I0H3	Saccharopine dehydrogenase-like oxidoreductase (EC 1. .-.-)	Sccpdh	SCPDL_RAT	0,42865422	0,106896351	4,00999859	11	0,00205085	0,00760764
Q99NA5	Thioredoxin-dependent peroxide reductase, mitochondrial (EC 1.11.1.15) (PRx III) (Peroxiredoxin-3) {PRX-3)	Prdx3	PRDX3_RAT	-0,4238368	0,135681736	-3,1237572	202	0,00204797	0,00760764
Q62951	RWD domain-containing protein 1 (Small androgen receptor-interacting protein)	Rwddl Sarip	RWDDI_RAT	0,22613583	0,072784202	3,10693565	295	0,00207448	0,00767559
Q9QZ76	ATP synthase subunit e, mitochondrial (ATPase subunit e)	Atp5i	ATP5I_RAT	0,29295797	0,076507085	3,82916136	13	0,00208846	0,00770753
Q6AY30	Rho GDP-dissociation inhibitor 1 (Rho GDI 1) (Rho-GDI alpha)	Arhgdia	GDIRI_RAT	-0,9601438	0,28630286	-3,3535948	31	0,00211596	0,00778912
Q9ZOV6	Dolichyl-diphosphooligosaccharide-protein glycosyltransferase subunit DADI (Oligosaccharyl transferase subunit DADI) (EC 2.4.99.18) (Defender against cell death 1) (DAD-1)	Dadl	DADI _RAT	-0,4208137	0,13428501	-3,1337354	124	0,00215449	0,00791076
P54313	Neurocalcin-delta	Ncald	NCALD_RAT	-0,3850077	0,122070511	-3,1539779	84	0,0022347	0,00818445
Q99ND9	Secretogranin-3 {1B1075) (Secretogranin III) (SgIII)	Scg3	SCG3_RAT	0,64569465	0,138132944	4,67444353	7	0,0022756	0,00831315
P29419	Guanine nucleotide-binding protein G(o) subunit alpha	Gnaol GnaO Gnao	GNAO_RAT	-0,4771591	0,139108442	-3,4301235	23	0,0022853	0,00832749
Q5XI73	ATP-dependent 6-phosphofructokinase, platelet type (ATP-PFK) (PFK-P) {EC 2.7.1.11) (6-phosphofructokinase type C) {Phosphofructo-1-kinase isozyme C) (PFK-C) (Phosphohexokinase)	Pfkp Pfkc	PFKAP_RAT	0,3040762	0,098972843	3,07231954	331	0,00230025	0,00836086
P61805	Vesicle-associated membrane protein-associated protein A (VAMP-A) (VAMP-associated protein A) (VAP-A) (33 kDa VAMP-associated protein) (VAP-33)	VapaVap33	VAPA_RAT	-0,2983691	0,087228368	-3,4205509	23	0,00233901	0,0084804
Q5PQNO	Elongator complex protein 1 {ELPl) (lkappaB kinase complex-associated protein) {IKK complex-associated protein)	Ikbkap Elpl Ikap	ELPI_RAT	-0,9866562	0,273425897	-3,6084956	16	0,00235654	0,00852251
P47868	Serine/arginine-rich splicing factor 2 (Splicing component, 35 kDa) (Splicing factor SC35) (SC-35) (Splicing factor, arginine/serine-rich 2)	Srsf2Sfrs2	SRSF2_RAT	-1,2935613	0,359580099	-3,5974219	16	0,00241232	0,00870243
P59215	60S ribosomal protein L13a	Rpl13a	RL13A_RAT	-0,661208	0,17303812	-3,8211694	12	0,0024343	0,00875985
P47860	Cellular nucleic acid-binding protein (CNBP) (Zinc finger protein 9)	CnbpZnf9	CNBP_RAT	-0,1629952	0,053685655	-3,036104	728	0,00248206	0,00890947
Q9Z270	Rap guanine nucleotide exchange factor 2 (Cyclic nucleotide ras GEF) (CNrasGEF) (Neural RAP guanine nucleotide exchange protein) (nRap GEP) (PDZ domain-containing guanine nucleotide exchange factor 1) (PDZ-GEFI) (RA-GEF-1) (Ras/Rapl-associatingGEF-1)	Rapgef2 Pdzgefl	RPGF2_RAT	-0,4102194	0,128480884	-3,1928437	47	0,00251403	0,009C0187
Q8VHU4	Elongation factor 1-alpha 1 (EF-1-alpha-1) (Elongation factor Tu) (EF-Tu) (Eukaryotic elongation factor 1 A-1) (eEFIA-1)	EeflalEefla	EF1 Al _RAT	0,32470731	0,099562379	3,26134543	32	0,00263533	0,00941282
Q6POU1	Stress-induced-phosphoprotein 1 (STIl) (Hsc70/Hsp90-organizing protein) (Hop)	Stipl Hop	STIPI _RAT	-0,2735938	0,083601887	-3,2725789	30	0,0026841	0,00956335
P35427	Ras-related protein Ral-A	Raia Ral Ral-a	RALA_RAT	-0,3079693	0,095706443	-3,2178532	36	0,00273251	0,00971186
P62634	40S ribosomal protein S29	Rps29	RS29_RAT	-0,3993425	0,088435096	-4,5156564	7	0,00274638	0,00973715
F1M386	Calcium/calmodulin-dependent protein kinase type 1 (EC 2.7.11.17) {CaM kinase I) (CaM-KI) {CaM kinase I alpha) {CaMKI-alpha)	Camkl	KCCl A_RAT	0,29227591	0,080699558	3,62177825	14	0,00277593	0,00981779
P62630	Heat shock protein 75 kDa, mitochondrial (HSP 75) (TNFR-associated protein 1) (Tumor necrosis factor type 1 receptor-associated protein) (TRAP-1)	Trapl Hsp75	TRAPI_RAT	-0,5051709	0,16654077	-3,0333166	166	0,00280793	0,00990672
035814	Alpha-ketoglutarate-dependent dioxygenase FTO (EC 1.14.11.-) (Fat mass and obesity-associated protein)	Fto	FTO_RAT	-0,2143288	0,071613753	-2,9928437	547	0,00288871	0,01014211
P63322	E3 ubiquitin-protein ligase NEDD4 (EC 6.3.2.-)	Nedd4Nedd4a	NEDD4_RAT	-0,1359232	0,043008924	-3,1603477	43	0,00288462	0,01014211
P6227S	E3 ubiquitin-protein ligase RNF181 (EC 6.3.2.-) (RING finger protein 181)	Rnf181	RN181_RAT	-0,3746728	0,117457336	-3,1898629	37	0,00289737	0,01014782
Q634SO	Potassium voltage-gated channel subfamily A member 2 (RAK) (RBK2) (RCK5) (Voltage-gated potassium channel subunit Kvl.2)	Kcna2	KCNA2_RAT	-0,3103107	0,100467995	-3,088652	67	0,00292555	0,01022172
Q5XHZO	Inosine triphosphate pyrophosphatase (ITPase) (Inosine triphosphatase) (EC 3.6.1.19) (Non-canonical purine NTP pyrophosphatase) (Non-standard purine NTP pyrophosphatase) (Nucleoside-triphosphate diphosphatase) (Nucleoside-triphosphate pyrophosphatase) (NTPase)	Itpa	ITPA_RAT	-0,221018	0,063274517	-3,493002	16	0,00300767	0,01048327
Q2A121	NEDD8-activating enzyme El regulatory subunit (Amyloid beta precursor protein-binding protein 1, 59 kDa) (APP-BPI) (Amyloid protein-binding protein 1)	Nael Appbpl	ULAI_RAT	0,43721088	0,135386285	3,22935868	29	0,00307831	0,01070363
Q62940	40S ribosomal protein SlI	RpslI	RSII_RAT	0,18889222	0,062091834	3,04214279	83	0,00314418	0,01088624
Q6AXU4	Ubiquitin thioesterase OTUBI (EC 3.4.19.12) (Deubiquitinating enzyme OTUBI) (OTU domain-containing ubiquitin aldehyde-binding protein 1) (Otubain-1) (Ubiquitin-specific-processing protease OTUBI)	Otubl	OTUBI _RAT	0,12659955	0,036465921	3,47172235	16	0,00314592	0,01088624
P63142	Microtubule-associated protein IA {MAP-I A) [Cleaved into: MAPIA heavy chain; MAPI light chain LC2]	MaplaMtapla	MAP1A_RA T	-0,3127486	0,089623618	-3,4895779	15	0,00329321	0,01136867
D3ZW55	Glycogenin-1 (GN-1) (GNI) (EC 2.4.1.186)	GyglGyg	GLYG_RAT	0,15847798	0,052261798	3,03238674	66	0,00346578	0,01192523
Q9ZIA5	Hematological and neurological expressed 1 protein [Cleaved into: Hematological and neurological expressed 1 protein, N-terminally processed]	Hol	HNI _RAT	0,43483057	0,140169041	3,10218696	41	0,00347096	0,01192523
P62282	40S ribosomal protein S21	Rps21	RS21_RAT	-0,2266994	0,07386114	-3,0692652	49	0,003493	0,01197245
B2RYG6	Guanine nucleotide-binding protein subunit beta-4 (Transducin beta chain 4)	Gob4	GBB4_RAT	-0,2947516	0,100356841	-2,937035	259	0,00361187	0,01235055
P34926	Homeodomain-only protein (Global ischemia-induced gene 15B protein) (Gllg15b) (Odd homeobox protein 1)	Hopx Giig15b Hod Hop Obl	HOP_RAT	-0,119057	0,040951271	-2,9072843	1746	0,00369183	0,0125941
008730	Proteasome subunit beta type-2 (EC 3.4 .25.1) (Macropain subunit C7-I) (Multicatalytic endopeptidase complex subunit C7-I) (Proteasome componentC7-I)	Psmb2	PSB2_RAT	0,26394314	0,086505463	3,05117307	44	0,00385486	0,01311926
Q6AXU6	Fascin	Fscnl	FSCNI_RAT	0,27290601	0,076578487	3,56374257	12	0,00389694	0,01323127
P05765	Long-chain fatty acid transport protein 1 (FATP-1) (Fatty acid transport protein 1) (EC 6.2.1.-) {Solute carrier family 27 member 1)	Slc27a1 Fatp Fatp l	S27A I _RAT	-0,3075919	0,095619735	-3,216824	22	0,00397141	0,01345245
035353	Tropomodulin-2 {Neuronal tropomodulin) (N-Tmod)	Tmod2Ntmod	TMOD2_RAT	-0,6806557	0,187962832	-3,6212251	11	0,00401784	0,01357786

P97526	Pyruvate dehydrogenase El component subunit beta, mitochondrial (PDHEl-B)(EC 1.2.4.1)	Pdhb	ODPB_RAT	-0,3731662	0,117678432	-3,1710674	22	0,00442323	0,01474069
Q5XIH7	Low molecular weight phosphotyrosine protein phosphatase (LMW-PTP) (LMW-PTPase) (EC 3.1.3.48) (Low molecular weight cytosolic acid phosphatase) (EC3.1.3.2)	Acpl	PPAC_RAT	-0,5473899	0,181623833	-3,0138662	38	0,00457516	0,01521188
Q5XIM5	Phosphatidate cytidylyltransferase 2 (EC 2.7 .7.41) (CDP-DAG synthase 2) (CDP-DG synthase 2) (CDP-diacylglycerol synthase 2) (CDS 2) (CDP-diglyceride pyrophosphorylase 2) (CDP-diglyceride synthase 2) (CTP:phosphatidate cytidylyltransferase 2)	Cds2	CD52_RAT	0,1469181	0,048605651	3,02265477	36	0,00459582	0,01524544
P13596	Septin-8	sep-08	SEPT8_RA T	-0,2855775	0,0998384	-2,8603971	227	0,00462575	0,01530954
P48508	MOB-like protein phocein (Class II mMOBl) (Mobl homolog 3) (Mob3) (Mps one binder kinase activator-like 3) (Phocein) (Preimplantation protein 3)	Mob4 Mob3 Mobkll Phocn Prei3	PHOCN_RAT	0,19808608	0,053006885	3,73698777	9	0,00464705	0,01534484
P49432	Heat shock-related 70 kDa protein 2 (Heat shock protein 70.2) (Testis-specific heat shock protein-related) (HST)	Hspa2 Hst70	HSP72_RAT	-0,2654106	0,093346066	-2,8432968	364	0,00471688	0,01548372
P41498	Neuronal calcium sensor 1 (NCS-1) (Frequenin homolog) (Frequenin-like protein) (Frequenin-like ubiquitous protein)	Ncsl Flup Freq	NCSl _RAT	-0,2749301	0,091581312	-3,0020323	38	0,00472087	0,01548372
Q91XU8	Complement C4 [Cleaved into : Complement C4 beta chain; Complement C4 alpha chain; C4a anaphylatoxin; Complement C4 gamma chain]	C4C4a	C04_RAT	-0,2311338	0,070483969	-3,2792388	16	0,0047213	0,01548372
BOBNFI	Neurofilament medium polypeptide (NF-M) (160 kDa neurofilament protein) (Neurofilament 3) (Neurofilament triplet M protein)	Nefm Nef3 Nfm	NFM_RAT	-0,2416473	0,080511441	-3,0014035	37	0,00479199	0,01567993
Q9QYW3	Ragulator complex protein LAMTOR3 (late endosomal/lysosomal adaptor and MAPK and MTOR activator 3) (Mitogen-activated protein kinase kinase 1-interacting protein 1)	Lamtor3Map2kl ipl	LTOR3_RAT	0,29608913	0,089717843	3,30022567	15	0,00485666	0,01585556
P14659	L-lactate dehydrogenase A chain (LOH-A) (EC 1.1.1.27) (LOH muscle subunit) (LDH-M)	Ldha Ldh-1 Ldhl	LDHA_RAT	0,35863765	0,125175699	2,86507403	125	0,00489237	0,0159361
P62168	Glutathione S-transferase alpha-4 (EC 2.5.1.18) (GST 8-8) (GST A4-4) (GST K) (Glutathione S-transferase Yk) (GST Yk)	Gsta4	GSTA4_RAT	-0,642756	0,134483693	-4,7794342	5	0,00497312	0,01616265
P08649	Hsc70-interacting protein (Hip) (Protein FAMlOAl) (Protein ST13 homolog)	St13 FamlOal Hip	Fl OAl _RAT	0,18703809	0,065519996	2,85467181	126	0,0050391	0,01634026
P12839	Protein FAM98A	Fam98a	FA98A_RAT	-0,2424886	0,086201011	-2,81306	523	0,0050918	0,01645759
Q5U204	Dolichyl-diphosphooligosaccharide-protein glycosyltransferase subunit 2 (EC 2.4.99.18) (Dolichyl-diphosphooligosaccharide--protein glycosyltransferase 63 kDa subunit) (Ribophorin II) (RPN-11) (Ribophorin-2)	Rpn2	RPN2_RA T	0,22030757	0,067938296	3,24275974	16	0,00509809	0,01645759
P04642	Tumor protein p63-regulated gene 1-like protein (Mossy fiber terminal-associated vertebrate-specific presynaptic protein)	Tprgll Mover	TPRGL_RAT	0,31054538	0,109861046	2,82671054	221	0,0051342	0,01653717
P14942	Protein RUFY3 (Rap2-interacting protein x) (RIPx) (Single axon-regulated protein) (Singar)	Rufy3 Ripx	RUFY3_RAT	-0,2670248	0,092651601	-2,8820314	72	0,00520435	0,0167258
P50503	1-phosphatidylinositol 4,5-bisphosphate phosphodiesterase gamma-1 (EC 3.1.4.11) (Phosphoinositide phospholipase C-gamma-1) (Phospholipase c-gamma-1) (PLC-gamma-1)	Plcgl	PLCGl _RAT	0,17684964	0,062679902	2,82147286	209	0,00524133	0,01680653
Q5FWT1	Sortingnexin-3	Snx3	SNX3_RA T	-0,2213087	0,030206448	-7,3265396	3	0,00525277	0,01680653
P25235	STE20/SPS1-related proline-alanine-rich protein kinase (Ste-20-related kinase) (EC 2.7.11.1) (Pancreatic serine/threonine-protein kinase) (PS/TK) (PSTKl) (Serine/threonine-protein kinase 39)	Stk39PaskSpak	STK39_RA T	-0,4782051	0,164495442	-2,9071024	51	0,00538743	0,01719925
A8WCF8	Neuroendocrine protein 7B2 (Secretogranin V) (Secretogranin-5) (Secretory granule endocrine protein I) (Cleaved into : N-terminal peptide; C-terminal peptide]	Scg5Sgnel	7B2_RAT	-1,0496345	0,355193848	-2,9551032	37	0,00541086	0,01723591
Q5FVJO	Cytochrome c, somatic	Cycs	CYC_RAT	0,24483566	0,075648063	3,23650926	15	0,00553418	0,01758992
P10686	Renin receptor (ATPase H(+)-transporting lysosomal accessory protein 2) (ATPase H(+)-transporting lysosomal-interacting protein 2) (Renin/prorenin receptor)	Atp6ap2 Atp6ip2	RENR_RAT	0,24898914	0,081849598	3,0420326	24	0,00561472	0,01780669
Q5U211	Translation initiation factor eIF-2B subunit beta (eIF-2B GDP-GTP exchange factor subunit beta)	Eif2b2 Eif2bb	EI2BB_RAT	-0,172995	0,061006436	-2,835684	83	0,00574272	0,01817269
088506	Microsomal glutathione 5-transferase 1 (Microsomal GST-1) (EC 2.5.1.18) (Microsomal GST-I)	Mgst1Gst12	MGSTl _RA T	-0,4116384	0,145062158	-2,8376689	74	0,00586076	0,01850565
P27682	Cytosolic non-specific dipeptidase (EC 3.4.13.18) (CNDP dipeptidase 2)	Cndp2	CNDP2_RA T	-0,3841559	0,129610671	-2,9639216	30	0,00590073	0,01859116
P62898	Estradiol 17-beta-dehydrogenase 12 (EC 1.1.1.62) (17-beta-hydroxysteroid dehydrogenase 12) (17-beta-HSD 12) (3-ketoacyl-CoA reductase) (KAR) (EC 1.3.1.-)	Hsd17b12	DHB12_RAT	-0,4319113	0,154281844	-2,7994953	119	0,00597361	0,01877977
Q6AXS4	Gap junction alpha-1 protein (Connexin-43) (Cx43) (Gap junction 43 kDa heart protein)	GjalCxn-43	CXAl _RAT	-0,2154179	0,06660104	-3,234453	14	0,00599529	0,01880696
Q62818	Isocitrate dehydrogenase [NADP] cytoplasmic (IDH) (EC 1.1.1.42) (Cytosolic NADP-isocitrate dehydrogenase) (IDP) (NADP(+)-specific ICDH) (Oxalosuccinate decarboxylase)	Idhl	IDHC_RAT	0,25745545	0,087095021	2,95602949	30	0,00601841	0,01883853
P08011	ATP-dependent (S)-NAD(P)H-hydrate dehydratase (EC 4.2.1.93) (ATP-dependent NAD(P)HX dehydratase) (Carbohydrate kinase domain-containing protein)	Carkd	NNRD_RAT	-0,5352834	0,150096609	-3,5662589	9	0,00605962	0,01892646
Q6QON1	Bifunctional protein NCOAT (Meningioma-expressed antigen 5) (Nuclear cytoplasmic 0-GlcNAcase and acetyltransferase) [Includes: Beta-hexosaminidase (EC 3.2.1.52) (Beta-N-acetylhexosaminidase) (Hexosaminidase C) (N-acetyl-beta-D-glucosaminidase) (N-acetyl-beta-glucosaminidase) (0-GlcNAcase); Histone acetyltransferase (HAT) (EC 2.3.1.48)]	Mgea5HexcMea5	NCOAT_RAT	0,26435506	0,095650822	2,763751	290	0,00607979	0,01894847
Q6P7R8	NADH dehydrogenase [ubiquinone] iron-sulfur protein 4, mitochondrial (Complex 1-18 kDa) (Cl-18 kDa) (NADH-ubiquinone oxidoreductase 18 kDa subunit)	Ndufs4	NDUS4_RAT	-0,3306618	0,062941989	-5,2534374	4	0,00628248	0,01953797
P08050	Parvalbuminalpha	PvalbPva	PRVA_RAT	-0,338931	0,122259181	-2,7722337	140	0,00632534	0,01962897
P41562	Propionyl-CoA carboxylase beta chain, mitochondrial (PCCase subunit beta) (EC 6.4.1.3) (Propanoyl-CoA:carbon dioxide ligase subunit beta)	Pccb	PCCB_RAT	-0,2136784	0,077912982	-2,7425266	368	0,00639486	0,01980212
D4AAT7	405 ribosomal protein 517	Rps17	RS17_RAT	-0,5464413	0,149600485	-3,6526707	8	0,00646935	0,01998986
Q8VIJ5	Matrin-3 (Nuclear scaffold protein p130/MAT3)	Matr3	MATR3_RA T	0,30862709	0,084615474	3,64740725	8	0,00651875	0,02009949
Q5XIF3	Leucine-rich PPR motif-containing protein, mitochondrial (130 kDa leucine-rich protein) (LRP 130) (Leucine rich protein 157) (rLRP157)	Lrpprclrp157	LPPRC_RAT	-0,4517239	0,110893962	-4,0734765	6	0,00655008	0,02015303
P02625	Glutathione S-transferase P (EC 2.5.1.18) (Chain 7) (GST 7-7) (GST class-pi)	Gstpl	GSTPl_RAT	-0,308276	0,112454106	-2,7413497	164	0,00679785	0,02087086
P07633	Integrin alpha-1 (CD49 antigen-like family member A) (Laminin and collagen receptor) (VLA-1) (CD antigen CD49a)	Itgal	ITAl _RAT	-0,2973322	0,106970097	-2,779582	70	0,00698436	0,02139795
P04644	Tubulin-specific chaperone E (Tubulin-foldingcofactor E)	Tbce	TBCE_RAT	-0,2239736	0,079010578	-2,8347292	41	0,00708842	0,02167073
P43244	Acyl-coenzyme A thioesterase 1 (Acyl-CoA thioesterase 1) (EC 3.1.2 .2) (CTE-1) (Inducible cytosolic acyl-coenzyme A thioester hydrolase) (LACH2) (ACH2) (Long chain acyl-CoA thioester hydrolase) (Long chain acyl-CoA hydrolase)	AcotlCtel	ACOTl _RAT	-0,4140835	0,130143499	-3,1817453	13	0,00721681	0,02201661
Q5SGEO	Glutathione S-transferase A6 (EC 2.5.1.18) (GST class-alpha member 6)	Gsta6	GSTA6_RAT	-0,4660597	0,168164063	-2,7714581	65	0,00727048	0,02213355
P04906	Peroxiredoxin-6 (EC 1.11.1.15) (1-Cys peroxiredoxin) (1-Cys PRX) (Acidic [illegible] specific antioxidant protein)	[illegible] Prdx6 Aipla2 Aop2 Tsa	PRDX6_RAT	0,4256345	0,157406429	2,70404775	237	0,00734659	0,02231818
P18614	Stromal interaction molecule 1	StimlSim	STIMl _RAT	-0,4294674	0,066382293	-6,4696081	3	0,00749359	0,02271691
Q5FVQ9	40S ribosomal protein Sl5 (RIG protein)	Rps15Rig	RS15_RAT	0,18472315	0,060418205	3,05740869	16	0,00752134	0,02275322
088267	Alpha-soluble NSF attachment protein (SNAP-alpha) (N-ethylmaleimide-sensitivefactorattachmentproteinalpha)	NapaSnapSnapa	SNAA_RAT	0,85082525	0,229604147	3,70561793	7	0,00759854	0,02293869
Q6AXYO	28 kDa heat- and acid-stable phosphoprotein (PDGF-associated protein) (PAP) (PDGFA-associated protein 1) (PAPl)	Pdapl Haspp28	HAP28_RA T	0,50098999	0,182430241	2,74620037	63	0,0078494	0,02364653
035244	Tropomyosin alpha-1chain (Alpha-tropomyosin) (Tropomyosin-1)	Tpml Alpha- tm Tpma	TPMl _RAT	0,13899693	0,052268793	2,65927184	427	0,00812547	0,02442614
P84903	ADP-sugar pyrophosphatase (EC 3.6.1.13) (8-oxo-dGDP phosphatase) (EC 3.6.1.58) (Nucleoside diphosphate-linked moiety X motif 5) (Nudix motif 5)	Nudt5	NUDT5_RA T	-0,3627281	0,107353089	-3,3788324	9	0,00814205	0,02442614
P62845	Enoyl-CoA hydratase domain-containing protein 3, mitochondrial	Echdc3	ECHD3_RAT	0,34958282	0,090262992	3,87293633	6	0,0082378	0,02466214
P54921	Trifunctional enzyme subunit beta, mitochondrial (TP-beta) [Includes: 3-ketoacyl-CoA thiolase (EC 2.3.1.16) (Acetyl-CoA acyltransferase) (Beta-ketothiolase)]	Hadhb	ECHB_RAT	-0,1783394	0,066624595	-2,6767809	148	0,00827205	0,02466905
Q62785	Galectin-3 (Gal-3) (35 kDa lectin) (Carbohydrate-binding protein 35) (CBP 35) (Galactose-specific lectin 3) (IgE-binding protein) (Lami nin-b inding protein) (Lectin L-29)(Mac-2antigen)	Lgals3	LEG3_RAT	0,2359186	0,086223896	2,73611621	57	0,0082743	0,02466905
P04692	COP9 signalosome complex subunit 3 (SGN3) (Signalosome subunit 3)	Cops3Csn3	CSN3_RA T	0,29260045	0,087118818	3,35863656	9	0,00840735	0,02496257
Q6AY63	FACT complex subunit SSRPl (Facilitates chromatin transcription complex subunit SSRPl) (Recombination signal sequence recognition protein 1) (Structure-specific recognition protein 1) (T160)	SsrplCiidbp	SSRPl _RAT	0,19993794	0,064431977	3,10308567	13	0,00839675	0,02496257
Q3MIEO	Serum albumin	Alb	ALBU_RAT	0,20891419	0,069574307	3,0027492	16	0,00843101	0,0249814

Q60587	Isocitrate dehydrogenase [NAO] subunit gamma 1, mitochondrial (EC 1.1.1.41) (Isocitric dehydrogenase subunit gamma) (NAD(+)-specific ICDH subunit gamma)	Idh3g	IDHGI _RAT	-0,3755165	0,138358142	-2,7140907	61	0,00862855	0,02551435
P08699	Cysteine sulfinic acid decarboxylase (EC 4.1.1.29) (Cysteine-sulfinate decarboxylase) (Sulfinoalanine decarboxylase)	CsadCsd	CSAD_RAT	0,263S7134	0,097375227	2,70675965	63	0,00873296	0,02571769
Q68FW9	Receptor-type tyrosine-protein phosphatase-like N (R-PTP-N) (105 kDa islet cell antigen) (BEM-3) (Brain-enriched membrane-associated protein tyrosine phosphatase) (ICA105) (PTP IA-2) (PTPLP)	Ptprn	PTPRN_RAT	-0,41422	0,153937129	-2,6908385	78	0,00871616	0,02571769
Q04931	Fatty aldehyde dehydrogenase (EC 1.2.1.3) (Aldehyde dehydrogenase 4) (Aldehyde dehydrogenase family 3 member A2) (Microsomal aldehyde dehydrogenase) {msALDH)	Aldh3a2Aldh4	AL3A2_RAT	-0,1947872	0,065283487	-2,9837133	16	0,00877242	0,02578127
P02770	Ras-related protein Rab-GA (Rab-6)	Rab6a Rab6	RAB6A_RAT	0,16137293	0,061399732	2,62823515	548	0,00882357	0,0258789
P41565	NAO kinase 2, mitochondrial (EC 2.7.1.23) {Mitochondrial NAO kinase) (NAO kinase domain-containing protein 1, mitochondrial)	Nadk2 Mnadk Nadkdl	NAKD2_RAT	-0,2167161	0,081832142	-2,6483004	138	0,00903218	0,026437
Q64611	ATP-dependent 6-phosphofructokinase, liver type (ATP-PFK) (PFK-L) (EC 2.7.1.11) (6-phospho fructokinase type B) {Phosphofructo-1-kinase isozyme 8) (PFK-B) (Phosphohe xokinase)	Pfkl Pfk-l	PFKAL_RAT	0,2119S147	0,077145324	2,74743118	37	0,00922582	0,0269491
Q63259	Cytochrome c oxidase subunit 7A2, mitochondrial (Cytochrome c oxidase subunit VIia-iiver/heart) (Cytochrome c oxidase subunit VIla-L)	Cox7a2 Cox7a3 Cox7a I	CX7A2_RAT	0,27S19278	0,067084059	4,10220825	5	0,00933465	0,02721192
P30839	Glycylpeptide N-tetradecanoyltransferase 1 (EC 2.3.1.97) (Myristoyl-CoA:protein N-myristoyltransferase 1) (NMT 1) (Type IN-myristoyltransferase) (Peptide N-myristoyltransferase 1)	NmtI	NMTI_RAT	-0,2233296	0,084356584	-2,6474472	104	0,00937191	0,02726546
Q9WV81	Omega-amidase NIT2 (EC 3.5.1.3) (Nitrilase homolog 2)	Nit2	NIT2_RAT	-0,2089S61	0,080114857	-2,6082067	160	0,00996365	0,02892867
Q1HCL7	Dihydropyrimidinase-related protein 2 (DRP-2) (Collapsin response mediator protein 2) {CRMP-2) (Turned on after division 64 kDa protein) (TOAD-64)	Dpysl2	DPYL2_RAT	-0,2119071	0,077618846	-2,730098	33	0,01007867	0,02920385
P30835	265 proteasome non-ATPase regulatory subunit 1 (26S proteasome regulatory subunit RPN2) (26S proteasome regulatory subunit SI) (26S proteasome subunit p112)	Psmdl	PSMDI_RAT	-0,306S233	0,117215307	-2,6150447	107	0,0102089	0,02952194
P35171	cAMP-dependent protein kinase catalytic subunit beta (PKA C-beta) (EC 2.7.11.11)	PrkacbPkacb	KAPCB_RAT	-0,412S822	0,149752568	-2,7550927	26	0,01057275	0,03051295
Q8K1QO	Sulfotransferase 1 family member 01 (ST1DI) (EC 2.8.2.-) (Dopamine sulfotransferase SultIdl) (Tyrosine-ester sulfotransferase)	SultIdl	ST1DI _RAT	0,46073286	0,170951346	2,69511103	36	0,01062662	0,0306072
Q49780	Prefoldinsubunit2	Pfdn2	PFD2_RAT	-0,2348954	0,090285998	-2,6016809	93	0,01079395	0,03102722
P47942	Electron transfer flavoprotein subunit alpha, mitochondrial (Alpha-ETF)	Etfa	ETFA_RAT	-0,2706SS9	0,106002859	-2,5532883	712	0,01087847	0,03111981
088761	Alpha-mannosidase 2Cl (EC 3.2.1.24) (Alpha-0-mannoside mannohydrolase) (Mannosidase alpha class 2C member 1)	Man2cl	MA2C1_RAT	0,1189S62	0,046342902	2,56687	232	0,01089085	0,03111981
P68182	Dipeptidyl peptidase 2 (EC 3.4.14.2) (Dipeptidyl aminopeptidase II) (Dipeptidyl peptidase 7) (Dipeptidyl peptidase II) (OPP II) (Quiescent cell proline dipeptidase)	Dpp7 Dpp2	DPP2_RAT	-O,S06S948	0,188828584	-2,6828289	37	0,0108491	0,03111981
G3V9R3	Tissue alpha-L-fucosidase (EC 3.2.1.51) (Alpha-L-fucosidase I) (Alpha-L-fucoside fucohydrolase 1) (Alpha-L-fucosidase 1)	FucalFuca	FUCO_RAT	-0,1544S38	0,048634105	-3,1758329	9	0,01125992	0,0321108
808N18	40S ribosomal protein S2	Rps2	RS2_RAT	-0,298S376	0,111657968	-2,6736796	35	0,01132226	0,0322249
P13803	Serine protease inhibitor A3M (Serpin A3M) (Serine protease inhibitor 2.4) (SPI-2.4)(Fragment)	Serpina3m	SPA3M_RAT	-0,2928386	0,114984253	-2,5467715	255	0,01146093	0,03255537
P21139	Insulin-degrading enzyme (EC 3.4.24.56) (Insulin protease) (lnsulinase) (Insulysin)	Ide	IDE_RAT	-1,11766	0,330139284	-3,3854197	7	0,01167209	0,03309003
Q9EP81	Tubulin polymerization-promoting protein family member 3	Tppp3	TPPP3_RAT	0,20334878	0,079435512	2,55992284	115	0,01176614	0,03329126
P17164	Myosin-9 (Cellular myosin heavy chain. type A) (Myosin heavy chain 9) (Myosin heavy chain, non-muscle IIa) (Non-muscle myosin heavy chain A) (NMMHC-A) {Non-muscle myosin heavy chain IIa) (NMMHC II-a) (NMMHC-IIA)	Myh9	MYH9_RAT	0,30SSS074	0,105699788	2,89074122	14	0,01185698	0,03348263
P27952	Proteasome subunit beta type-8 (EC 3.4.25.1) (Macropain subunit C13) (Multicatalytic endopeptidase complex subunit C13) (Proteasome component C13) (Proteasome subunit beta-Si)	Psmb8	PSB8_RAT	-0,2766204	0,107852655	-2,564799	84	0,01210125	0,03410567
Q63556	Leukocyte elastase inhibitor A (Serine protease inhibitor EIA) (Serpin Bla)	Serpinbla	ILEUA_RAT	0,79317117	0,207018012	3,83141137	5	0,01223009	0,03440161
P35559	NADH-ubiquinone oxidoreductase chain 4 (EC 1.6.5.3) (NADH dehydrogenase subunit4)	Mtnd4 mt-Nd4 Nd4	NU4M_RAT	-0,1862764	0,070409965	-2,6455976	33	0,01239338	0,03479309
Q5PPN5	Delta-1-pyrroline-5-carboxylate dehydrogenase, mitochondrial (PSC dehydrogenase) (EC 1.2.1.88) (Aldehyde dehydrogenase family 4 member AI) (L-glutamate gamma-semialdehyde dehydrogenase)	Aldh4al	AL4AI _RAT	-0,2716874	0,107653224	-2,5237278	149	0,01266001	0,03547261
Q62812	Heme oxygenase 2 (H0-2) (EC 1.14 .99 .3)	Hmox2	HMOX2_RAT	0,2172621S	0,085581975	2,53864378	98	0,01270261	0,035523
P28064	Monocarboxylate transporter 1 (MCT 1) (Solute carrier family 16 member 1)	Slc16a1Mctl	MOTI _RAT	0,2881S02	0,100933709	2,85484608	14	0,01272767	0,03552425
Q4G075	cGMP-dependent 3',5'-cyclic phosphodiesterase (EC 3.1.4.17) (Cyclic GMP-stimulated phosphodiesterase) (CGS-PDE) (cGSPDE)	Pde2a	PDE2A_RAT	0,28380917	0,112428309	2,52435682	121	0,01288561	0,03589562
P05508	Lambda-crystallin homolog {EC 1.1.1.45) (L-gulonate 3-dehydrogenase) (Gul3DH)	Cryll Cry	CRYLI_RAT	-0,7S343S3	0,176684385	-4,2643006	4	0,01300898	0,03616949
POC2X9	CD59 glycoprotein (MAC-inhibitory protein) (MAC-IP) (Membrane attack complex inhibition factor) (MACIF) (Protectin) (CD antigen CD59)	Cd59	CDS9_RAT	-0,416S90S	0,161765681	-2,5752711	49	0,01308543	0,03622078
P23711	Small glutamine-rich tetratricopeptide repeat-containing protein beta (Beta-SGT) (Small glutamine-rich protein with tetratricopeptide repeats 2)	Sgtb Sgt2	SGTB_RAT	-0,22734	0,086145752	-2,6390158	30	0,0130593	0,03622078
P53987	Rho-associated protein kinase 2 (EC 2.7.11.1) (Rho-associated, coiled-coil-containing protein kinase 2) (Rho-associated, coiled-coil-containing protein kinase II) (ROCK-II) (RhoA-binding kinase 2) (pISO ROK-alpha) (ROKalpha) (p164 ROCK-2)	Rock2	ROCK2_RAT	-0,346S974	0,10917686	-3,1746419	8	0,01310274	0,03622078
Q01062	Complex III assembly factor LYRM7 (LYR motif-containing protein 7)	Lyrm7MZM1L	LYRM7_R AT	-1,1S77226	0,351701489	-3,2917763	7	0,01327004	0,03661314
Q811X6	Alpha-actinin-1(Alpha-actinin cytoskeletal isoform) (F-actin cross-linking protein) (Non-musclealpha-actinin-1)	ActnI	ACTNI_RAT	0,212402S9	0,082430677	2,576742	45	0,01332421	0,03669243
P27274	C-1-tetrahydrofolate synthase. cytoplasmic (Cl-THF synthase) (Cleaved into: C-1-tetrahydrofolate synthase. cytoplasmic, N-terminally processed] (Includes: Methylenetetrahydrofolate dehydrogenase (EC 1.5.1.5); Methenyltetrahydrofolate cyclohydrolase (EC 3.5.4.9); Formyltetrahydrofolate synthetase (EC 6.3.4.3)]	MthfdlMthfd	CITC_RAT	-0,3548811	0,13617563	-2,6060541	35	0,01336332	0,03673003
Q80W98	Alpha-crystallin Bchain (Alpha(B)-crystallin)	Cryab	CRYAB_RAT	-0,4476662	0,173038678	-2,5870876	39	0,01352323	0,03709891
Q62868	Synaptotagmin-1 {Synaptotagmin I) (Sytl) {p65)	Sytl	SYTI_RAT	0,311S8338	0,104510736	2,98135288	10	0,01377579	0,03772004
84F7A1	Protein bassoon	Bsa	BSN_RAT	-0,4913226	0,058448345	-8,4060987	2	0,01385828	0,03787406
Q9Z1P2	Delta(3,5)-Delta(2,4)-dienoyl-CoA isomerase, mitochondrial (EC 5.3.3.-)	Echl	ECHI_RAT	0,3329S63	0,124055763	2,68392449	21	0,01389599	0,03790531
P27653	Splicing factor U2AF 26 kDa subunit (Liver regeneration-related protein LRRG157/LRRG158) (U2 auxiliary factor 26) (U2 small nuclear RNA auxiliary factor 1-like protein 4)	U2afll4 Cb2-806 Cb2-807	U2AF4_RAT	-0,24SS32	0,09855167	-2,4914037	130	0,01398309	0,03799925
P23928	Enoyl-CoA hydratase. mitochondrial (EC 4.2.1.17) (Enoyl-CoA hydratase 1) (Short-chain enoyl-CoA hydratase) (SCEH)	Echsl	ECHM_RAT	0,2690378	0,107714511	2,49769316	111	0,01396698	0,03799925
P21707	LanC-like protein 1 (40 kDa erythrocyte membrane protein) (p40)	Lancl1Gpr69a	LANCl _RAT	-0,203220S	0,08151156	-2,4931489	120	0,01402441	0,03803989
088778	40S ribosomal protein S26	Rps26	RS26_RAT	O,SS1S2871	0,161126582	3,42295297	6	0,01409254	0,03815297
Q62651	Pyruvate carboxylase, mitochondrial (EC 6.4.1.1) (Pyruvic carboxylase) (PCB)	Pc	PYC_RAT	-0,2080982	0,079969298	-2,6022265	29	0,01443864	0,03901678
Q7TP17	Prostamide/prostaglandin F synthase (Prostamide/PGF synthase) (Prostamide/PGF synthase) (EC 1.11.1.20)	Fam213b	PGFS_RAT	-0,392S277	0,107475232	-3,6522619	5	0,01471342	0,03968499
P14604	Inactive phospholipase C-like protein 1 (PLC-LI) (PRIPI) (Phospholipase c-related but catalytically inactive protein) (p130)	Plcll	PLCLI_RAT	-0,8S91377	0,344383466	-2,4947125	67	0,01507775	0,04059179
Q9QX69	40S ribosomal protein S3	Rps3	RS3_RAT	-0,2854728	0,114205974	-2,499631	56	0,01538934	0,04135348
P62856	Isovaleryl-CoA dehydrogenase, mitochondrial (IVD) (EC 1.3.8.4)	Ied	IVD_RAT	-0,2336S8	0,091965137	-2,5407236	36	0,01551691	0,04161877
P52873	Eukaryotic translation initiation factor 5 (eIF-5)	EifS	IFS_RAT	-0,09S707	0,039462539	-2,4252609	435	0,01570349	0,04204107
03ZVR7	N(G),N(G)-dimethylarginine dimethylaminohydrolase 2 (DDAH-2) (Dimethylarginine dimethylaminohydrolase 2) (EC 3.5.3.18) (DDAHII) (Dimethylargininase-2)	Ddah2	DDAH2_RAT	0,26466454	0,086681954	3,05328298	8	0,01574234	0,04206703
Q62688	40S ribosomal protein S16	Rps16	RS16_RAT	0,18993896	0,077055936	2,46494915	77	0,01593032	0,04249068
P62909	60S ribosomal protein LIS	RplIS	Rl I S_RAT	-0,1140143	0,046961489	-2,427826	226	0,01597201	0,04252326
	Creatine kinase LI-type, mitochondrial (EC 2.7.3 .2) (Acidic-type mitochondrial								

P12007	creatine kinase) (Mia-CK) (Ubiquitous mitochondrial creatine kinase) (U-MtCK)	CkmtlCkmt	KCRU_RAT	-0,6S061S9	0,258051256	-2,5212663	38	0,01600802	0,04254066
Q07205	Triosephosphate isomerase (TIM) (EC 5.3.1.1) (Triose-phosphate isomerase)	Tpil	TPIS_RAT	-0,26104S3	0,106376059	-2,4539857	77	0,01638698	0,04346766
Q6MG60	Elongator complex protein 2 (ELP2) (SHINC-2) (STAB-interacting protein 1) (StIPI)	Elp2Statipl	ELP2_RAT	0,191248S7	0,078602525	2,43310978	118	0,01647005	0,04360786

P62250	Unconventional myosin-le (Myosin heavy chain myr 3) (Unconventional myosinlE)	MyoleMyr3	MYOl E_RA T	-0,1930592	0,078556582	-2,457582	68	0,0165423	0,04371894
P61314	Multifunctional protein ADE2 [Includes : Phosphoribosylaminoimidazole-succinocarboxamide synthase (EC 6.3.2.6) (SAICAR synthetase); Phosphoribosylaminoimidazole carboxylase (EC 4.1.1.21) (AIR carboxylase) (AIRC)]	PaicsAde2Airc	PUR6_RA T	-0,295456	0,119726197	-2,4677643	56	0,01667751	0,04399569
P25809	Calcium -dependent secret ion activator 1 (Calcium-dependent activator protein for secretion 1) (CAPS-1) (rCAPS)	CadpsCapsCaps 1	CAPSl_RAT	-0,2808628	0,116546779	-2,409872	164	0,01706478	0,04488742
P48500	Ubiquitin-fold modifier 1	Ufml	UFMl_RAT	-0,261204	0,108574613	-2,4057561	194	0,01707775	0,04488742
Q496ZO	Proteasome subunit alpha type-1 (EC 3.4.25.1) (Macropain subunit C2) (Multicatalytic endopeptidase complex subunit C2) (Proteasome component C2) (Proteasome nu chain)	Psmal	PSAl_RAT	0,2742433	0,091627495	2,99302411	8	0,01725419	0,04526873
Q63356	4F2 cell-surface antigen heavy chain (4F2hc) (Solute carrier family 3 member 2) (CD antigen CD98)	Slc3a2	4F2_RAT	-0,3593958	0,123488905	-2,9103492	9	0,01730141	0,04531023
P51583	Excitatory amino add transporter 2 (GLT-1) (Sodium-dependent glutamate/aspartate transporter 2) (GLUT-RI (Solute carrier family 1 member 2)	Slcl a2 Eaat2 Gltl	EAA2_RAT	0,20275183	0,084163973	2,40900975	135	0,017345	0,0453421
Q62717	Fetuin-B (Fetuin-like protein IRL685)	Fetub	FETUB_RAT	-0,1715035	0,071909501	-2,3849904	530	0,01743009	0,04548214
Q5BJP3	AP-2 complex subunit sigma (Adaptor protein complex AP-2 subunit sigma) (Adaptor-related protein complex 2 subunit sigma) (Clathrin assembly protein 2 sigma small chain) (Clathrin coat assembly protein AP17) (Clathrin coat-associated protein AP17) (Plasma membrane adaptor AP-217 kDa protein) (Sigma-adaptin 3b) (Sigma2-adaptin)	Ap2sl Ap17 Claps2	AP2S1_RAT	-0,2908844	0,118962019	-2,4451875	55	0,01770778	0,04612334
P18420	Obg-like ATPase 1	Olal	OLAl _RAT	0,22991856	0,096120181	2,39199046	175	0,01781699	0,04632418
Q794F9	DnaJ homolog subfamily A member 1 (DnaJ-like protein 1) (Heat shock proteinJ2) (HSJ-2)	Dnajal Hsj2Rdjl	DNJAl _RA T	-0,4478929	0,186265337	-2,4045959	108	0,01789182	0,04643507
P31596	Cyclin-G-associated kinase (EC 2.7.11.1)	Gak	GAK_RA T	-0,4040241	0,163827075	-2,4661619	41	0,01792881	0,04644754
Q9QX79	Prenylcysteine oxidase (EC 1.8.3.5) (Chlo ri de ion pump-associated 55 kDa protein)	PcyoxlClp55	PCYOX_RAT	0,39782188	0,134528643	2,95715375	8	0,01822531	0,04713104
P62744	CBl cannabinoid receptor-interacting protein 1 (CRIP-1)	Cnripl	CNRPl_RAT	-0,2481328	0,103460028	-2,3983444	88	0,01858153	0,04796628
AOJPJ7	Paraspeckle component 1	Pspcl	PSPCl _RAT	-0,2884174	0,12164861	-2,3709062	214	0,01863213	0,04801102
P63036	Protein kinase C alpha type (PKC-A) (PKC-alpha) (EC 2.7.11.13)	Prkca Pkca	KPCA_RAT	-0,4735792	0,185784204	-2,549082	21	0,01868093	0,04805095
P97874				-0,320701	0,126873553	-2,5277212	23	0,01880578	0,04811478
Q99MLS				-0,2588049	0,106711057	-2,4252862	52	0,01880423	0,04811478
Q5M7A7				-0,1333702	0,050185159	-2,6575623	14	0,01875015	0,04811478
Q4KLH4				-0,4328384	0,127142916	-3,4043452	5	0,01916048	0,04893554
P05696				-0,19C0497	0,076722683	-2,4770995	28	0,01955016	0,04984256

Supplementary Table S2: Altered proteins by Ad-Bgal infection after RA

Protein	Description	Gene	Entry Name	Estimate Fold Change	Std. Error	t value	DF	p value	adj. p val
B0BN93	265 proteasome non-ATPase regulatory subunit 13 (26S proteasome regulatory subunit RPN9) (26S proteasome regulatory subunit 511) (265 proteasome regulatory subunit p40.5)	Psmd13	PSD13_RAT	-1,155993263	0,10892362	-10,61288	125		
035550	Rab GTPase-binding effector protein 1 (Rabapt in-5) (Rabaptin-Salpha) ll	RabptS Ral	RABEl_RAT	-2,27050162	0,05875012	-38,64676	54		
P04041	Glutathione peroxidase 1 (GPx-1) (GSHPx-1) (EC 1.11.1.9) (Cellular glutathione peroxidase)	Gpxl	GPXl_RAT	-1,866954523	0,09452584	-19,750732	79		
P05545	Serine protease inhibitor A3K (Serpin A3K) (CPI-21) (Contraps in-like protease inhibitor 1) (GHR-P63) (Growth hormone-regulated proteinase inhibitor) (Kallikrein-binding protein) (KBP) (SPI-2.3) (Serine protease inhibitor 2) (SPI-2) (Thyroid hormone-regulated protein)	[illegible] Spin;	SPA3K_RAT	2,937846306	0,10974679	26,7693132	116		
P07633	Propionyl-CoA carboxylase beta chain, mitochondrial (PCCase subunit beta) (EC 6.4.1.3) (Propanoyl-CoA:carbon dioxide ligase subunit beta)	Pccb	PCCB_RAT	-1,797240535	0,10526829	-17,072952	70		
P09812	Glycogen phosphorylase, muscle form (EC 2.4.1.1) (Myophosphorylase)	Pygm	PYGM_RAT	-0,929480115	0,07284617	-12,759492	239		
P11442	Clathrin heavy chain 1	Cite	CLHl_RAT	-0,74546929	0,04031118	-18,492866	1787		
P13264	Glutaminase kidney isoform, mitochondrial (GLS) (EC 3.5.1.2) (K-glutaminase) (L-glutamine amidohydrolase) [Cleaved into: Glutaminase kidney isoform 68 kDa chain; Glutaminase kidney isoform 65 kDa chain]	Gls	GLSK_RAT	-4,293237217	0,23892245	-17,969166	27		
P13437	3-ketoacyl-CoA thiolase, mitochondrial (EC 2.3.1.16) (Acetyl-CoA acyltransferase) (Beta-ketothiolase) (Mitochondrial 3-oxoacyl-CoA thiolase)	Acaa2	THIM_RAT	-0,882236527	0,07146997	-12,344157	168		
P18422	Proteasome subunit alpha type-3 (EC 3.4.25.1) (Macropain subunit CB) (Multicatalytic endopeptidase complex subunit CB) (Proteasome component CB) (Proteasome subunit K)	Psma3	PSA3_RAT	-4,304068385	0,09205354	-46,75614	104		
P24368	Peptidyl-prolyl cis-trans isomerase B (PPlase B) (EC 5.2.1.8) (CYP-51) (Cyclophilin B) (Rotamase B) (5-cyclophilin) (SCYLP)	Ppib	PPIB_RAT	-1,431301824	0,08693548	-16,463955	99		
P26284	Pyruvate dehydrogenase El component subunit alpha, somatic form, mitochondrial (EC 1.2.4.1) (PDHEl-A type I)	Pdhal	ODPA_RAT	-0,813234733	0,08563672	-9,4963323	419		
P27605	Hypoxanthine-guanine phosphoribosyltransferase (HGPRT) (HGPRTase) (EC 2.4.2.8)	Hprtl Hprt	HPRT_RAT	-1,969194585	0,07336624	-26,840609	179		
P28023	Dynactin subunit 1 (150 kDa dynein-associated polypeptide) (DAP-150) (DP-150) (p150-glued)	Dctnl	DCTNl_RAT	-0,870695974	0,05969286	-14,586267	444		
P29266	3-hydroxyisobutyrate dehydrogenase, mitochondrial (HIBADH) (EC 1.1.1.31)	Hibadh	3HIDH_RAT	-1,821803001	0,12235181	-14,889873	142		
P30349	Leukotriene A-4 hydrolase (LTA-4 hydrolase) (EC 3.3.2.6) (Leukotriene A(4) hydrolase)	Lta4h	LKHA4_RAT	-0,751880219	0,05300106	-14,186136	251		
P35281	Ras-related protein Rab-10	RablO	RABlO_RAT	-1,897923999	0,10398805	-18,251367	58		
P38650	Cytoplasmic dynein 1 heavy chain 1 (Cytoplasmic dynein heavy chain 1) chl (Dynein heavy chain, cytosolic) (MAP lC)	Dnchcl	DYHCl_RAT	-0,401498078	0,0297316	-13,504084	2339		
P41562	Isocitrate dehydrogenase [NADP] cytoplasmic (IDH) (EC 1.1.1.42) (Cytosolic NADP-isocitrate dehydrogenase) (IDP) (NADP(+)-specific ICDH) (Oxalosuccinate decarboxylase)	ldhl	IDHC_RAT	-0,887248369	0,09088786	-9,7620118	368		
P47858	ATP-dependent 6-phosphofructokinase, muscle type (ATP-PFK) (PFK-M) (EC 2.7.1.11) (6-phosphofructokinase type A) (Phosphofructo-1-kinase isozyme A) (PFK-A) (Phosphohexokinase)	Pfkm Pfk-m	PFKAM_RAT	-0,964282021	0,08735507	-11,038649	423		
P48037	Annexin A6 (Annexin VI) (Annexin-6) (Calcium-binding protein 65/67) (CBP 65/67)	Anxa6 Anx6	ANXA6_RAT	-0,871593341	0,05142861	-16,947636	680		
P50878	605 ribosomal protein L4 (605 ribosomal protein Ll)	Rpl4 Rpll	RL4_RAT	-2,158034132	0,07953625	-27,13271	55		
P51146	Ras-related protein Rab-4B	Rab4b	RAB4B_RAT	-1,867074322	0,1068527	-17,473347	73		
P61107	Ras-related protein Rab-14	Rab14	RAB14_RAT	-0,908661026	0,0973764	-9,3314295	250		
P62193	265 protease regulatory subunit 4 (P 26s4) (265 proteasome AAA-ATPase subunit RPT2) (Proteasome 265 subunit ATPase 1)	Psmcl	PRS4_RAT	-1,122195028	0,09087523	-12,348744	147		
Q08602	Geranylgeranyl transferase type-2 subunit alpha (EC 2.5.1.60) (Geranylgeranyl transferase type II subunit alpha) (Rab geranyl-geranyltransferase subunit alpha) (Rab GG transferase alpha) (Rab GGTase alpha) (Rab geranylgeranyltransferase subunit alpha)	Rabggta Ggta	PGTA_RAT	-0,676874953	0,06077722	-11,136985	191		
Q498R7	UPF0587 protein Clorf123 homolog		CA123_RAT	-2,221915385	0,09304396	-23,880274	30		
Q4KMA2	UV excision repair protein RAD23 homolog B	Rad23b	RD23B_RAT	1,962226805	0,11497222	17,0669642	63		
Q5HZV9	Protein phosphatase 1 regulatory subunit 7 (Protein phosphatase 1 regulatory subunit 22)	Ppplr7 Sds22	PP1R7_RAT	-0,803745626	0,05917421	-13,582702	270		
Q5RJR2	Twinfilin-1	Twfl Ptk9	TWFl_RAT	-1,170523566	0,07656887	-15,287199	119		
Q5U300	Ubiquitin-like modifier-activating enzyme 1 (Ubiquitin-activating enzyme El)	Ubal Ubel	UBAl_RAT	-0,643963036	0,05858279	-10,992359	659		
Q62667	Major vault protein (MVP)	Mvp	MVP_RAT	-0,890462113	0,06497203	-13,705314	152		
Q63525	Nuclear migration protein nude (Nuclear distribution protein C homolog) (c15)	Nude	NUDC_RAT	-0,970424587	0,08849056	-10,966419	201		
Q63598	Plastin-3 (T-plastin)	Pls3	PLST_RAT	-1,797176968	0,07403173	-24,275767	327		
Q641W2	UPF0160 protein MYGl, mitochondrial l	11ygl C12orfl	MYGl_RAT	-2,292223596	0,08842302	-25,923381	42		
Q64537	Calpain small subunit 1 (CSSl) (Calcium-activated neutral proteinase small subunit) (CANP small subunit) (Calcium-dependent protease small subunit) (COPS) (Calcium-dependent protease small subunit 1) (Calpain regulatory subunit)	:rnsl Capn4 C	CPNSl_RAT	-1,008948593	0,10565995	-9,5490165	135		
Q75Q39	Mitochondrial import receptor subunit TOM70 (Mitochondrial precursor proteins import receptor) (Translocase of outer membrane 70 kDa subunit)	Tomm70a	TOM70_RAT	-2,622044683	0,1588691	-16,504435	44		
Q80Z30	Protein phosphatase lE (EC 3.1.3.16) (Ca(l+)/calmodulin-dependent protein kinase phosphatase N) (CaMKP-N) (CaMKP-nucleus) (CaMKN) (Partner of PIX 1) (Partner of PIX-alpha) (Partner of PIXA)	)pmle Camkr	PPMlE_RAT	3,033463252	0,12027047	25,2220114	31		
Q8VHF5	Citrate synthase, mitochondrial (EC 2.3.3.1) (Citrate (Si)-synthase)	Cs	CISY_RAT	-1,184299439	0,08651213	-13,689403	162		
Q8VHU4	Elongator complex protein 1 (ELPl) (IkappaB kinase complex-associated protein) (IKK complex-associated protein)	:bkap Elpl lka	ELPl_RAT	-2,273872952	0,09120203	-24,932262	32		
Q8Vl04	Isoaspartyl peptidase/L-asparaginase (EC 3.4.19.5) (EC 3.5.1.1) (Asparaginase-like protein 1) (Asparaginase-like sperm autoantigen) (Beta-aspartyl-peptidase) (Glial asparaginase) (Isoaspartyl dipeptidase) (L-asparagine amidohydrolase) [Cleaved into: Isoaspartyl peptidase/L-asparaginase alpha chain; Isoaspartyl peptidase/L-asparaginase beta chain]	:ll Alp Gliap	ASGLl_RAT	-0,706488669	0,06361015	-11,10654	180		
Q9ESB5	N-terminal EF-hand calcium-binding protein 1 (EF-hand calcium-binding protein 1) (Neuronal calcium-binding protein 1) (Synaptotagmin-interacting protein 1) (Stip-1)	cabl Efcbpl	NECAl_RAT	5,742156428	0,05986842	95,9129505	11		
Q9QXQ0	Alpha-actinin-4 (F-actin cross-linking protein) (Non-muscle alpha-actinin 4)	Actn4	ACTN4_RAT	-0,963209148	0,05736175	-16,791836	374		
Q9WU06	Advillin (Peripheral nervous system villin-like protein) (Pervin)	Avil Advil	AVIL_RAT	1,712477336	0,04285966	39,9554606	86		
035832	Cyclin-dependent kinase 18 (EC 2.7.11.22) (Cell division protein kinase 18) (PCTAIRE-motif protein kinase 3) (Serine/threonine-protein kinase PCTAIRE-3)	Pctaire3 Pc	CKD18_RAT	-3,035012572	0,202468	-14,990086	40		
054975	Xaa-Pro aminopeptidase 1 (EC 3.4.11.9) (Aminoacylproline aminopeptidase) (Cytosolic aminopeptidase P) (Soluble aminopeptidase P) (sAmp) (X-Pro aminopeptidase 1) (X-prolyl aminopeptidase 1, soluble) [Cleaved into: Xaa-Pro aminopeptidase 1, N-terminally processed]	Xpnpepl App	XPPl_RAT	-0,960815694	0,08423602	-11,406233			

P02770	Serum albumin	Alb	ALBU_RAT	0,738372087	0,060207	12,2638916	548
P05708	Hexo kinase -1 (EC 2.7.1.1) (Bra in form hexok inase) (Hexokinase type I) {HK I)	Hkl	HXKl_RAT	-0,809562732	0,05859809	-13,815513	372
P08649	Com ple ment C4 [Cle aved in to: Complement C4 beta chain; Comp le ment C4 alpha chain; C4a anaphy latox in; Complement C4 gamma chain]	C4C4a	C04_RAT	-0,76523277	0,07763929	-9,856257	126
P19139	Casein kinase II subunit alpha (CK II alpha) (EC 2.7.11.1)	Csnk2al	CSK21_RAT	-2,259309202	0,10057301	-22,46437	134
P23514	Coatomer subunit beta (Beta-coat protein) (Beta-COP)	C opbl Copb	COPB_RAT	-3,758 15657	0,12848663	-29,249399	65
P25093	Fum ar ylacetoacetase {FAA) (EC 3.7.1.2) (Be ta-d ike to nase) {Fum arylacetoacetate hydro lase)	Fah	FAAA_RAT	2,943395967	0,07453284	39,491265	38
P27952	405 ribosomal protein 52	Rps2	RS2_RAT	-1,431678329	0,10145487	-14,111479	84
P30713	Glu tat hione 5-transferase theta -2 (EC 2.5.1.18) (GST 12 -12) (GST class-theta -2) {Glutathione 5-transferase 12) (G1utath ione 5-transferase Yrs-Yrs)	Gstt2	GSTT2_RAT	-2,052793962	0,13208336	-15,541655	44
P63029	Trans lat ion ally-contro lle d tumor protein (TCTP) (Lens epithelial protein)	Tptl Trt	TCTP_RAT	-1,869054341	0,11354997	-16,460192	92
P63047	Sulfot rans fe rase 4Al {ST4Al) (EC 2.8.2.-) (Brai n sulfotransferase-like protein) (rBR-STL) (Nervous system sulfot rans fe rase) (NST)	Sult4al Sultx3	ST4Al_RAT	-1,818396947	0,11699982	-15,541879	97
P80299	Bifu nctiona l epoxide hydrolase 2 [Includes: Cytosolic epoxide hydrolase 2 {CEH) (EC 3.3.2.10) (Epoxide hydratase) (Soluble epoxide hydrolase) {SEH); li pid-p hosphate phosphatase (EC 3.1.3.76))	Ephx2	HYE S_RAT	-4,339620517	0,15236915	-28,480966	23
Q00981	Ubiquitin carboxy l-te rmina l hydrolase isozyme Ll (UC H-L1) (EC 3.4.19.12) (EC 6.- . -. -) (Ne uron cytoplasmic protein 9.5) (PGP 9.5) (PGP9.5) (Ub iqu itin th ioeste rase Ll)	Uchll	UCHLl_RAT	-1,243175877	0,10637935	-11,686252	353
Q02253	Met hylmalon ate-sem ialdehyde dehydrogenase (acylat ing], mitochondrial {MMSDH) {Malonate-semialdehyde dehydrogenase [acylat in g]l (EC 1. 2.1.18) (EC 1.2.1.27) (Aldeh yde dehydrogenase family 6 member Al)	ldh6al Mmsd	MMSA_RAT	-1,694679321	0,08972105	-18,888315	179
Q4QQT4	Serine/ threon ine- protein phosphatase 2A 65 kDa regulatory subunit A beta isoform {PP2A subunit A isoform PR65-beta) (PP2A subunit A isoform Rl-beta)	Ppp2rlb	2AAB_RAT	-3,361818517	0,10473686	-32,097759	56
QSXI72	Euka ryotic translation in itiat ion factor 4H {elF-4H) (W illiams -Beuren syndrome chromosomal region 1 protein homo1og)	Eif4hWbscrl	I F4H_RAT	1,806000744	0,08835017	20,4413961	92
Q63570	265 protease regulator y subunit 6B (265 proteasome AAA-ATPase subunit RPT3) {Proteasome 265 subunit ATPase 4) (56 ATPase) (Tat-bin din g prote in 7) {TBP-7)	Psmc4 Tbp7	PRS6B_RAT	-1,376584101	0,10541375	-13,058867	115
Q7TP36	Protein Shroom2 {liver regene ration -re lated protein LRRG167) (Protein Apxl)	*oml* Apxl Ab	SHRMl RAT -	2,580532516	0,10061334	25,6480165	56
Q8K4K5	Lethal(2) giant larvae protein homolog 1 {LLGL) (Rgl-1)	Llgll Rgll	L2Gll_RAT	-1,674884845	0,07461708	-22,446401	55
Q8R491	EH doma in-cont ain ing prote in 3	Ehd3 Ehd2	EHD3_RAT	-1,896633875	0,15980057	-11,868755	59
Q9Z1B2	Glu tath ione 5-transferase Mu 5 (EC 2.5.1.18) (GST class-mu 5)	GstmS	GSTMS_RAT	-1,309279271	0,13403977	-9,7678419	180
008618	Phos phoribosy l pyrophosphate synthase-associated protein 2 (PRPP synthase-associated protein 2) (41 kDa phosphoribosypyrophosphate synthetase-assoc iated protein) (PAP 41)	Prpsap2	KPRB_RAT	-1,110105689	0,09646208	-11,508208	111
P05370	Glu cose-6- phosphate 1-deh ydrogenase (G6PD) (EC 1.1.1.49)	G6pdx G6pd	G6PD_RAT	-0,616559064	0,05941486	-10,377185	291
P07943	Aldose reductase (AR) (EC 1.1.1.21) (Aldeh yde reductase)	lbl Akrlb4 A	ALDR_RAT	-1,046278383	0,07953282	-13,155303	341
P13697	NADP -de pendent ma lic enzyme (N: P-ME) (EC 1.1.1.40) (Malic enzyme el	Mod-l Mo	MAOX_RAT	-1,612975649	0,06218644	-25,937738	271
P39069	Adeny late kinase isoe nzyme 1 (AK 1) (EC 2.7.4.3) (EC 2.7.4.6) (ATP-AMP transphosphorylase 1) (ATP:AMP phosphotransferase) (Adenylate monophosphate kinase) {Myokinase)	Akl	KADl_RAT	-1,41198902	0,11229544	-12,573877	281
P47709	Rabph ilin-3 A (Exophi lin -1)	Rph3a	RP3A_RAT	-0,777375672	0,06525064	-11,913686	183
P53534	Glycogen phosphorylase, brain form (EC 2.4.1.1) (Fragment)	Pygb	PYGB_RAT	-0,748946083	0,07181354	-10,429037	658
P63039	60 kDa heat shock protein, mitochondria l (60 kDa chaperon in) {Chaperonin 60) {CPN60) {HSP-65) (He at shock protein 60) (HSP-60) {Hsp60) {Mit ochon dr ial mat rix prote in Pl)	Hspdl Hsp60	CH60_RAT	-0,995881845	0,07377091	-13,499654	645
P70619	Glutat hione reductase {GR) (GRase) (EC 1.8 .1. 7) (Fragment)	Gs,	GSHR_RAT	-2,724204813	0,09701889	-28,079116	42
P85973	Purine nucleoside phosphor ylase {PNP) (EC 2.4.2.1) (1nos ine phosphorylase){l nosine-guanosine phosphorylase)	Pnp Np	PNPH_RAT	-1,581606045	0,08106042	-19,511446	190
Q3KRD5	Mitochondrial im port recepto r subun it TOM34 {Translocase of outer membrane 34 kDa subunit)	Tomm34	TOM34_RAT	-1,328849333	0,07799808	-17,03695	116
QSXll O	Mamma lian ependymin-re lated protein 1 (MERP-1)	Epdr2 Merpl	EPDRl_RAT	-3,904997455	0,10763486	-36,280046	48
Q63450	Calcium/c almodu lin -dependent protein kinase type 1 (EC 2.7.11.17) {CaM kinase I) {Ca M- KI) {CaM kinase I alpha) (Ca M Kl- al pha)	Camkl	KCClA_RAT	-1,893110052	0,09070432	-20,871223	67
Q63560	Microtubu le- assoc iated protein 6 {MAP-6) (145-kDa STOP) (STOP145) {Stable tubule -o nly polypeptide) (STOP)	Map6 Mtap6	MAP6_RAT	0,796222264	0,05851883	13,6062567	280
Q64640	Adenosine kinase (AK) (EC 2.7.1.20) (Ade nos ine 5'-phosphotransferase)	Adk	ADK_RAT	-1,592044315	0,07436983	-21,407124	106
Q9JLZ1	Glu taredox in-3 {PKC-inte racting cous in of th io redoxin) (P ICOT) (PKC-the ta-in teract in g protein) {PKCq-interacting protein) (Th ioredoxin-li ke protein 2)	lrx3 PicotTxn	GLRX3_RAT	-2,730736099	0,2082629	-13,111966	54
P14669	Annexin A3 (35-alpha calcimedin) (Annexin Ill) (Annex i n-3) (lipocortin Ill) {Placenta l ant icoagu lant protein Ill) (PAP- Il l)	Anxa3 Anx3	ANXA3_RAT	-0,766928983	0,0729536	-10,512558	446
P20717	Protein-arginine deiminase type-2 (EC 3.5.3. 15) (Pept idylargi ni ne deiminase II) {Protein-arginine deiminase type II)	ladi2 Pdi Pdi2	PADl2_RAT	-1,162935017	0,12172523	-9,5537712	326
P22062	Protein -L- iso aspartate(D-asparta te) 0-methyltransferase (P I MT) (EC 2.1.1.77) (L- iso aspa rtyl protein carboxy l methy ltransferase) (Protein l-isoaspa rtyl/0 -a spa rtyl meth ylt ransferase) (Protein-beta-aspa rtate methyltransferase)	Pcmtl	PIMT_RAT	-1,18925548	0,08402155	-14,154172	241
P27139	Carbonic anhyd rase 2 (EC 4.2.1.1) (Carbo nate deh yd ratase II) (Carbonic anhyd rase II) {CA-II)	Ca2	CAH2_RAT	-1,739342176	0,10123354	-17,181482	260
P47860	ATP-dependent 6-phosphofructokinase, plate le t type (ATP-PFK) (PFK-P) (EC 2.7.1.11) (6-phosphofructokinase type C) (Ph osphofructo-1-ki nase isozyme C) {PFK-C) {Phosphohexokinase)	Pfkp Pfkc	PFKAP_RAT	-0,523227542	0,05643593	-9,2711782	728
P50399	Rab GDP dissociation inhib it or beta (Rab GDI beta) (GD l-3) (Gu anos i ne diphosphate dissociation inhibitor 2) {GDl-2)	Gdi2 Gdi3	GDI B_RAT	-1,452589745	0,07944798	-18,283533	455
P53042	Serine/threonine -prote in phosphatase 5 (PPS) (EC 3.1.3.16) (Protein phosphatase T) {PPT)	PppSc	PPPS_RAT	-1,647186071	0,10050081	-16,389779	120
Q01205	Dihy dro lipoyllysine-residue succiny ltrans fe rase component of 2-oxoglu tarate dehyd rogenase complex, mitochondria l (EC 2.3.1.61) (2-oxoglutarate dehydrogenase complex component El) (OGDC-E2) (Dih ydrolipoamide succ iny lt rans fe rase component of 2-oxoglutarate de hydrogenase comp le x) (E2K)	Dist	0002_RAT	1,556025115	0,06149978	25,3013105	133
Q05175	Brain acid so lubl e protein 1 (22 kDa neurona l tissue-enriched acidic protein) (Ne urona l axona l membrane protein NAP-22)	Baspl Nap22	BASPl_RAT	0,886550806	0,07739979	11,4541766	212
Q2TL32	E3 ubiquitin -p rotein ligase UBR4 (EC 6.3.2.-) (N- reco gnin- 4) (Zinc finger UBRl -t ype prote in 1)	·4 Rbaf600 Zu	UBR4_RAT	-1,221924852	0,09614133	-12,709673	133
QSM9G3	Caprin-1 (Cytop lasm ic activation- and prolife rat io n-assoc iated protein 1) {GPl- anchored protein p137) (GP l- pl3 7) (p 137 GP I) (RNA gran ule protein nl 105)	Gpiapl Rn	CAPRl_RAT	-1,989308809	0,15187522	-13,098311	52
QSRJQ4	NAO -de pendent protein deacetylase sirtuin-2 (EC 3.5.1.-) (Regulatory protein SIR2 homolo g 2) {SIR2-like protein 2)	Sirt2 Sir212	SIR2_RAT	-0,9656565	0,08107365	-11,910854	189
Q63270	Cytop lasm ic aconitate hydratase (Aconitase) (EC 4.2.1.3) (Cit rate hydro-lyase) {Iron regulator y protein 1) (IRPl) (Iron-respons ive element-bin din g prote in 1) {IRE-BP 1)	col lrebl lret	ACOC_RAT	-1,22318507	0,09592009	-12,752126	191
Q6AY84	Secernin-1	Scrnl	SCRNl_RAT	-0,845257181	0,09002042	-9,389616	342

Q9ER24	Ataxin-10 (Ne urona l bet a-caten in- like protein) (Spinoce rebe llar ataxia type 10 protein homolog)	AtxnlO ScalO	ATXlO_RAT	-3,173992751	0,20981817	-15,127349	47		
P47728	Calreti n in {CR)	Calb2	CALB2_RAT	-1,019814238	0,07525422	-13,551588	480		
Q68FR6	Elong at ion factor 1-gamma (EF-1-gamma) (e EF-1B gamma)	Eeflg	EFlG_RAT	-0,790054942	0,08148691	-9,6954826	314		
Q6AYC4	Macrophage-capping protein (Actin regu latory protein CAP-G)	Capg	CAPG_RAT	-0,936082129	0,07667609	-12,208266	101		
Q99N27	Sorting nexin-1	Snx1	SNXl_RAT	-1,532844996	0,06271155	-24,442785	88		
A0JPM9	Euka ryot ic trans lat ion in itiat ion facto r 3 subunit J {elF3j) (Eukaryotic tr ans lat ion initiation factor 3 subunit 1) {el F-3- alpha) (elF3 p35)	Eif3j Eif3sl	EIF3J_RAT	-3,109745972	0,07891836	-39,404596	42		
P04631	Protein SlOO-B {S-100 protein beta chain) (S-100 protein subunit beta) {Sl00 calcium-bin ding protein B)	Sl00b	Sl00B_RAT	-4,578924542	0,3645395	-12,560846	57		
P10760	Adenosy lhomocyste inase (AdoHcyase) (EC 3.3.1.1) (S-adenosyl-L-homocysteine hydro lase)	Ahcy	SAHH_RAT	-1,273912679	0,10614673	-12,001431	180		
P85968	6-phosphogluconate dehydrogenase, decarboxy lat in g{EC 1.1.1.44)	Pgd	6PGD_RAT	-1,007379197	0,10205309	-9,8711289	194		
Q5XHY5	Threonine--tRNA ligase, cytop lasm ic (EC 6.1.1.3) (Thre onyl-tRN A s ynthetase) {ThrRS)	Tars	SYTC_RAT	-1,678514378	0,07321089	-22,927113	105		
Q6BBl8	Ubi qu itin- fo ld modi fie r-conjugating enzyme 1 (Ufml-conjugating enzyme 1)	Ufcl	UFCl_RAT	-3,662171432	0,13062113	-28,036593	33		
Q9JIX3	Bis (5'-adenos yl)-tri phosphatase (EC 3.6.1.29) (AP3A hydrolase) (AP3Aase) (Diadenosine 5',5'''-Pl,P3-tr iphosphate hydrolase) (Dinu cleosidetri phosp hatase) {Fragile histidine triad protein)	Fhit	FHIT_RAT	-4,330556377	0,09043208	-47,88739	32		
008697	ADP-ribosylation factor- like protein 2	Arl2 Arl184	ARL2_RAT	-1,396935684	0,09861091	-14,166137	74		
Q04631	Protein farnes ylt rans fe rase /ge rany lge ra nylt ransferase type-1 subunit alpha (EC 2.5.1.58) (EC 2.5.1.59) (CAAX fa rnesy lt ransferase subunit alpha) {Frase-a lpha) (Ras proteins pre nylt ransferase subunit alpha) {Type I protein geranyl-ge rany lt rans fe rase subunit alpha) (GGT ase-1-alpha)	Fnta	FNTA_RAT	-1,955422271	0,08580939	-22,787976	118		
Q32PX2	Amin oacy l tRNA synthase comple x-inte racting mu lt ifunct io nal protein 2 {Mu ltisynth ase comp le x auxiliary component p38) (P rote in JTV-1)	Aimp2 Jtvl	AIMP2_RAT	-3,525463241	0,12827725	-27,483154	30		
Q63081	Protein disulfide-isomerase AG (EC 5.3.4.1) (Calci um-b ind ing protein 1) {CaBPl) {Protein disulfide isomerase PS) (Thioredox in domain-containinga6 protei n 7)	Cabpl Txn	PDIA6_RAT	-1,207218906	0,09965419	-12,114081	152		
Q6PECO	Bis (S'-nu cleosy l)- tetraphosphatase [asymmetrical] (EC 3.6.1.17) (Diadenosine 5',5'''-Pl,P4-tet raphosphate asymmet rical hydrolase) (Ap4A hydro lase) (Ap4Aase) (Diadenos ine tetraphosphatase) (Nucleoside diphosphate- linke d mo iety X motif 2) {Nu dix motif 2)	Nudt2	AP4A RAT	-2.383471099	0,21873393	-10,896669	88		
Q920J4	Thio redoxin- like protein 1 {Thiore dox in- related protein)	Txnll Trp	TXNLl_RAT	-0,857289401	0,09330608	-9,1879269	213		
B0BN18	Prefo ldin subunit 2	Pfdn2	PFD2_RAT	-4,880011032	0,10909077	-44,73349	35		
P62959	Histi dine triad nucleotide-binding protein 1 (EC 3.-.-.-) (17 kDa inhibitor of prote in kinase C) (Adenosine 5'-mo nop hosphoram idase) (Protein kinase C in hibi tor 1) {Protein kinase (-interacting protein 1) (P KCl-1)	intl Hint Pkci	HINTl_RAT	-2,386449782	0,15745197	-15,156685	114		
Q03114	Cyclin-dependent kinase 5 (EC 2.7.11.22) (Ce ll division protein kinase 5) {Serine/threonine-protein kinase PSSALRE) (Tau protein kinase II catalytic subu nit) {TPKII c ata lytic subunit)	CdkS CdknS	CDKS_RAT	-1,326557551	0,08069225	-16,439715	99		
Q9EPH8	Polyadeny late-b in ding protein 1 {PABP-1) (Po ly(A)-binding protein 1)	Jabpcl Pabpl	PABPl_RAT	-0,92823097	0,05345299	-17,36537	206		
Q9QZA2	Prog rammed cell death 6-inte racting prote in (ALG-2-interacting protein 1)	Pdcd6ip Aipl	PDC6I_RAT	-0,637056686	0,06213086	-10,253467	332		
Q6RUV5	Ras-re lated C3 botulinum toxin subst rate 1 (p21-Racl)	Rael	RACl_RAT	-2,477784895	0,16676642	-14,857817	67		
Q63468	Phosp horibosy l pyrophosphate synthase-associated protein 1 (PRPP synthase-associated protein 1) (39 kDa phosphoribosypyrophosphate syn thase-associated protein) (PAP39)	Prpsapl	KPRA_RAT	-1,659728354	0,1163728	-14,262168	79		
P04764	Alpha-eno lase (EC 4.2.1.11) (2- phosp ho-D-glycerate hydro-lyase) (Eno lase 1) (No n-neu ral eno lase) (NNE)	Enol Eno-1	ENOA_RAT	-0,907142297	0,07437394	-12,197045	425		
Q7TP48	Adipocyte plasma memb ra ne- assoc iated protein	pmap Ab2-3C	APMAP_RAT	-5,999531534	0,13421239	-44,701772	15		
035509	Ras-re lated protein Rab-llB	Rabllb	RBllB_RAT	-1,247571919	0,05645329	-22,099189	43		
P12368	cAMP-dependent protein kinase type II - alpha regu latory subunit	Prkar2a	KAP2_RAT	-0,97586403	0,10763736	-9,0662206	182	2,22E-16	2,52E-15
055043	Rho guanine nucleotide exchange factor 7 (Be ta-Pix) (PAK- interact ing exchange facto r beta)	gef7 Pak3bp	IARHG7_RAT	-6,297080183	0,08149624	-77,268355	11	2,22E-16	2,52E-15
Q497B0	Omega-amidase NIT2 (EC 3.5.1.3) (Nit rilase homolog 2)	Nit2	NIT2_RAT	-0,889474981	0,08905756	-9,9876415	93	2,22E-16	2,52E-15
Q07009	Calpain-2 catalytic subunit (EC 3.4.22.53) {Calcium-activated neutral proteinase 2) {CANP 2) {Calpain M-type) (Cal pa in-2 large subunit) {Millimo lar-ca lpain) {M-c alpain)	Capn2	CAN2_RAT	-0,763567719	0,08990884	-8,4926881	362	4,44E-16	4,89E-15
Q6P7B0	Tryptop han--t RNA ligase, cytop lasm ic (EC 6.1.1.2) (Tryptophany l-tR NA synthetase) {TrpRS) [Cle aved into: Tl-Trp RS; T2-TrpRS]	Wars	SYWC_RAT	-0,797890821	0,09079534	-8,7877944	230	4,44E-16	4,89E-15
089049	Thioredoxin reductase 1, cytop lasm ic {TR) (EC 1.8.1.9) (NADPH-dependent thioredoxin reductase) (Thiore dox in reductase TRl)	Txnrdl Trxrl	TRXRl_RAT	-0,841447229	0,09117388	-9,2290383	143	4,44E-16	4,89E-15
P13086	Succiny l-CoA ligase [ADP/GDP- fo rming] subunit alpha, mitochondrial (EC 6.2.1.4) (EC 6.2.1.5) {Succiny l-CoA synthetase subunit alpha) (SCS-alpha)	Suclgl	SUCA_RAT	-1,33128011	0,13928523	-9,5579415	109	4,44E-16	4,89E-15
P34067	Proteasome sub unit beta type-4 (EC 3.4.25.1) (Macropain beta chain) {Mu ltic ata lytic endopeptidase complex beta chain) (P roteasome beta chain) {Proteasome chain 3) (RN3)	Psmb4	PSB4_RAT	1,163488715	0,1280272	9,08782447	137	8,88E-16	9,71E-15
Q568Z9	Phytanoy l-CoA hydroxy lase- in teract ing protein (Ph yta noy l- CoA hyd roxylase-asso ciated protein 1) (PA HX-A Pl) (PAHXAPl)	Phyhip	PHYIP_RAT	-2,179931766	0,17076418	-12,765744	40	1,ll E-15	1 ,20E-14
Q8K1P7	Transcription activator BRGl (EC 3.6.4.-) (ATP-dependent helicase SMARCA4) (BRGl-associated factor 190A) (BA F190A) (Prote in brahma homo log 1) (SNF2-beta) {SWI/ SNF- re lated matrix-associated actin-dependent regu lato r of ch romatin sub fam ily A member 4)	1f190a Brgl S1	SMCA4_RAT	-9,876761293	0,06077476	-162,51419		2,22E-15	2,39E-14
Q62940	E3 ubiquitin- protein ligase NEDD4 (EC 6.3.2.-)	edd4 Nedd4;	NEDD4_RAT	-0,701895982	0,07235714	-9,7004388	83	2,66E-15	2,85E-14
054715	V-type proton ATPase subunit Sl (V-ATPase subunit S1) (C7-1 protein) (V-ATPase Ac45 subunit) (V-ATPase Sl accessory protein) (Vacuolar proton 1 pump subunit Sl)	pl Atp6ipl A	VASl_RAT	-3,519475208	0,13382319	-26,299442	17	3,llE-15	3,30E-14
QSUlZ0	Rab3 GTPase-activating protein non-cata lytic subunit (Rab3 GTPase-activat in g prote in 150 kDa subun it) (Rab3 -GAP plS0) (Rab3 -GAP150) (Rab3-GAP regu lato ry subun it)	Rab3gap2	RBGPR_RAT	-1,529373144	0,119785	-12,767652	36	6,22E-15	6,55E-14
P23928	Alpha-crysta llin B chain {Alpha(B)-crystallin)	Cryab	CRYAB_RAT	-1,137573993	0,12802283	-8,885712	111	1,29E-14	1,35E-13
QSU2P0	Dl S3-like exonuclease 1 (EC 3.1.13.-)	Dis31	Dl3Ll_RAT	-1,19143243	0,04541783	-26,2327	16	1,42E-14	1,48E-13
P06760	Beta-glucuronidase (EC 3.2.1.31)	GusbGus	BGLR_RAT	-7,566850599	0,06255995	-120,95358		2,44E-14	2,52E-13
Q63356	Unconventiona l myosin- le {Myosin hea vy chain myr 3) (Unconvent ional myosin lE)	Myole Myr3	MYOlE_RAT	-6,44631016	0,07750731	-83,170354		2,66E-14	2,73E-13
P36972	Adenine phospho ribosy ltrans fe rase (AP RT) (EC 2.4.2.7)	Aprt	APT_RAT	-0,789246307	0,09091021	-8,6816028	107	4,84E-14	4,92E-13
P50554	4- am inobutyrate am inotransfe rase, mitochondr ial (EC 2.6.1.19) ((S)-3-am ino- 2-methylpropionate transam inase) (EC 2.6.1.22) (GABA am inot rans fe rase) (GA BA-AT) (G amma -a mi no- N- butyrate transaminase) (GABA transaminase) (GA BA-Tl (L-AIBAT) (Cle aved into: 4-aminobutyrate am in otransfe rase, brain isofo rm; 4-ami nobutyrate aminotransferase, live r isoform]	AbatGabat	GABT_RAT	-0,986316056	0,12471619	-7,9084848	286	5,73E-14	5,78E-13
P29826	Rana class II histocompatibility antigen, B-1 beta chain {RTl class II his tocompat ibility antigen, B-1 beta chain) {RTl.B-beta{l))	RTl-Bb	HB2B_RAT	-7,151949199	0,07197182	-99,371515		1,17E-13	1,18E- 1 2
P13635	Ce rulo plasm in {EC 1.16 .3.1) {Ferroxidase)	Cp	CERU_RAT	0,631375185	0,07911378	7,98059648	193	1,27E-13	1,26E-1 2
P62198	26S protease regu la ry subunit 8 {26S proteasome AAA-ATPase subunit RPT6) {Proteasome 26S subunit ATPase 5) (P roteasome subunit p45) (Thyroid hormone receptor-inte racting protein 1) (TRIP!) (p45/SUG)	Psmc5 Sugl	PRS8_RAT	-1,412786124	0,16535533	-8,5439407	100	1,51E-13	1,50E-1 2
P17425	Hydroxymethy lg lutary l-CoA synthase, cytop lasm ic (HM G-CoA synthase) (EC 2.3.3.10) {3-hydrox y-3-methy lg lutary l coenzyme A synthase)	mgcsl Hmgc	HMCSl RAT	-0,777877291	0,0985199	-7,8956359	212	1,54E-13	1,51E-12

Q6AY30	Saccharopine dehydrogenase-like oxidoreductase (EC 1.-.-.-)	Sccpdh	SCPDL_RAT	-3,077332377	0,24919465	-12,349111	31	1,66 E-13	1,6 2E-1 2
Q3MIEO	Enoyl-CoA hydratase domain-containing protein 3, mitochondrial	Echdc3	ECHD3_RAT	1,527663838	0,06957431	21,9572988	16	2,26E-13	2,19E-12
P85515	Alpha-centractin (Centractin)	Actrla Ctrnl	ACTZ_RAT	-0,922650968	0,11768982	-7,8396835	203	2,50E-13	2,41E-12
P30835	ATP-dependent 6-phosphofructokinase, liver type (ATP-PFK) (PFK-L) (EC 2.7.1.11) (6-phosphofructokinase type B) (Phospho fructo-1-kinase isozyme B) (PFK-B) (Phosphohexokinase)	Pfkl Pfk-1	PFKAL_RAT	-1,008991658	0,12140212	-8,3111536	107	3,24E-13	3,lOE-12
P16086	Spectrin alpha chain, non -erythroc:: (Alpha-II spectrin) (Fodrin alpha anl Sp na 2 Sp		SPTNl_RAT	0,226764238	0,03113741	7,28269492	1298	5,66E-13	5,37E-12
P47942	Dihydropyrimidinase-related protein 2 (DRP-2) (Collapsin response mediator protein 2) (CRMP-2) (Turned on after division 64 kDa protein) {TOAD-64)	Dpysl2	DPYL2_RAT	-0,810287357	0,11043395	-7,3373032	712	5,96E-13	5,62E-12
Q9QUL6	Vesicle-fusing ATPase (EC 3.6.4.6) (N-e thylma1ei mide -sens iti ve fusion protein) (NEM-sensitive fusion protein) (Ves icular-fus ion protein NSF)	Nsf Ergl	NSF_RAT	-0,419265922	0,05734809	-7,3108965	821	6,30E-13	5,91E-12
Q63692	Hsp90 co-chaperone Cdc37 (Hsp90 chaperone protein ki nase -ta rget i ng subunit) (p50Cdc37) [Cle aved into: Hsp90 co-chaperone Cdc37, N-terminally processed]	Cdc37	CDC37_RAT	0,561641404	0,07397452	7,59236244	246	6,51E-13	6,07E-12
P63329	Serine/threonine -protein phosphatase 2B catalytic subunit alpha isoform (EC 3.1.3.16) (CAM-PRP catalytic subunit) (Calmodu lin-dependent calcineurin A subunit alpha isoform)	Ppp3ca Ca lna	PP2BA_RAT	7,784460359	0,06528101	119,245406		7,69E-13	7,llE-12
P85125	Polymerase I and transcript release factor (Cavin-1) (cav-p60)	Ptrf	PTRF_RAT	-0,795057448	0,04028297	-19,736811	16	1,17 E-1 2	1,08E-11
P00564	Creatine kinase M-type (EC 2.7.3.2) (Creat ine kinase M chain) (M-CK)	Ckm Ckmm	KCRM_RAT	0,674577865	0,06066506	11,1197098	32	1,59E-1 2	1,45E -11
P10687	1-phosphatidylinositol 4,5-bisphosphate phosphodiesterase beta-1 (EC 3.1.4.11) (PLC-154) (Phosphoinositide phospholipase C-be ta-1) (Phospholipase C-1) (PLC-I) (Phos pholipase C-beta- 1) (PLC-beta-1)	Plcbl	PLCBl_RAT	-1,735846585	0,17254952	-10,059991	40	1,62E-12	1,47E-11
035331	Pyridoxal kinase (EC 2.7.1.35) (Pyridox ine kinase)	Pdxk Pkh	PDXK_RAT	-0,71192295	0,09644173	-7,3818971	289	1,67E-1 2	1,51E-11
Q63798	Proteasome activator complex subunit 2 (115 regulator complex subunit beta) (REG-beta) (Activator of mu lticatalytic protease subunit 2) (Proteasome activator 28 subunit beta) (PA28b) (PA28beta)	Psme2	PSME2_RAT	-6,444387388	0,15739409	-40,944277	10	1,81E-12	1,62E-11
P06687	Sodium/potassium-transporting ATPase subunit alpha-3 (Na(+)/K(+) ATPase alpha-3 subunit) (EC 3.6.3.9) (Na(+)/K(+) ATPase alpha(III) subunit) (Sodium pump subunit alpha-3)	Atpla3	AT1A3_RAT	0,422779867	0,05934403	7,12421925	815	2,30E-12	2,05E-11
P46844	Biliverdin reductase A (BVR A) (EC 1.3.1.24) (Biliverdin -lX alpha-reductase)	Blvra Blvr	BIEA_RAT	1,049075489	0,13592829	7,71785982	130	2,76E-12	2,44E-11
P36506	Dual specificity mito gen- activated protein kinase kinase 2 (MAP kinase kinase 2) (MAPKK 2) (EC 2.7.12.2) (ERK activator kinase 2) (MAPK/ERK kinase 2) (MEK 2)	l Mek2 Mkk2	MP2K2_RAT	-7,554068593	0,11761757	-64,225682		3,84E-12	3,38E-11
Q5XIC3	Hsp90 co-chaperone Cdc37-like 1	Cdc3711	CD37L_RAT	-5,66034223	0,11985624	-47,226094		4,29E-12	3,75E-11
Q1WIMl	Cell adhesion molecule 4 (lmmunoglobulin superfamily member 4C) (lgSF4C) (Nectin-like protein 4) (NECL-4)	m4 lgsf4 c Ne	CADM4_RAT	0,499736621	0,06477156	7,71537059	116	4,61E-12	4,00E-11
Q5PQN6	Spermatogenesis-defective protein 39 homo lo g (hSPE-39) (VPS33B-in teract in g protein in apical-basolateral polarity re gulator) (VPS33B-in teract in g protein in polarity and apical restriction)	as39 Spe39 Vi	SPE39_RAT	-4,63460023	0,15087893	-30,717436	11	5,16E-12	4,45E-11
P18420	Proteasome subunit alpha type-1 (EC 3. 4.25.1) (M acropa i n subunit C2) (Multicatalytic endopeptidase complex subunit C2) (Proteasome component C2) (Proteasome nu chain)	Psmal	PSAl_RAT	-0,771424206	0,10455136	-7,3784234	175	6,19E-12	5,32E-11
Q1WIM3	Cell adhesion molecule 3 (lmmunoglobulin superfamily member 4B) (lgSF4B) (Nectin-like protein 1) (NECL-1)	lm3 lgsf4 b Ne	CADM3_RAT	0,624061598	0,06706918	9,3047447	43	7,34E-12	6,27E-11
Q5XIE6	3- hydroxyisobutyryl-CoA hyd ro lase, mitochondrial (EC 3.1.2.4) (3-hydroxyisobutyryl -coe nzyme A hydrolase) (HtB -CoA hydrolase) (HIBYL-CoA-H)	Hibch	HIBCH_RAT	3,85959473	0,2641771	14,6098762	19	8,74E-12	7,42E-11
Q99MS0	SEC14-like protein 2 (Alpha-tocopherol-associated protein) (TAP) (Squalene transfer protein) (Supernatant protein factor) (SPF)	Sec1412	S14L2_RAT	-1,187376538	0,15543282	-7,6391625	103	1,17 E-11	9,88E-11
P24329	Thiosulfate sulfurtransferase (EC 2.8.1.1) (Rhodanese)	Tst	THTR_RAT	-0,419510934	0,05742557	-7,3052985	153	1,4 2E-11	1,20E-10
055096	Dipeptidyl peptidase 3 (EC 3. 4.14.4) (Di peptidyl aminopeptidase Ill) (Dipeptidyl arylamidase Ill) (D ipeptidyl peptidase Ill) (OPP HI) (Enkephalinase B)	Dpp3	DPP3_RAT	-0,475833565	0,06748366	-7,0510926	271	1,48E-11	1,23E-10
Q80U96	Exportin-1 (Expl) (Chromosome regio n maintenance 1 protein homolog)	Xpol Crml	XPOl_RAT	-1,573862549	0,21390354	-7,3578143	128	1,99E-11	1,65E-10
B2RYI0	WO repeat -containin g protein 91	Wdr91	WDR91_RAT	-5,250587981	0,10098292	-51,994811		2,07E-11	1,71E-10
Q641Y5	Ubi qu itin -like modi fier -act ivat in g enzyme ATG7 (ATG12-activating enzyme El ATG7) (Autophagy-related protein 7) (APG7-like)	Atg7 Apg71	ATG7_RAT	-0,831697503	0,07318074	-11,364978	25	2,28E-11	1,87E-10
P12839	Neurofilament medium polypeptide (NF-M) (160 kDa neurofilament protein) (Neurofilament 3) (Neurofilament triplet M protein)	lefm Nef3 Nfr	NFM_RAT	0,558474907	0,08176685	6,83008921	523	2,36E-11	1,93 E-10
Q5U318	Ast roc ytic phosphoprotein PEA-15 (15 kDa phosphoprotein enriched in astrocytes)	Pea15	PEA15_RAT	-1,691106455	0,23875332	-7,08307	162	4,08E-11	3,31E-10
P31647	Sodium- and chloride-dependent GABA transporter 3 (GAT-3) (Solute carrier family 6 member 11)	1 Ga bt 3 Gat-.= 56	A ll_RAT	-0,683411132	0,09634046	-7,0937086	152	4,63E-11	3,73E-10
P37805	Trans ge lin -3 (Neuronal protein 22) (NP22) (Neu rona l protein NP25)	Tagln3 Np25	TAGL3_RAT	-1,362220167	0,1883496	-7,2324027	112	6,26E-11	4,99E-10
P35559	Insulin -deg radin g enzyme (EC 3.4.24.56) (Insu li n protease) (lnsulinase) {lnsulysin)	Ide	IDE_RAT	-0,794977673	0,08395853	-9,4686943	33	6,24E-11	4,99E-10
Q6P7A9	Lysoso ma l alpha-glucosidase (EC 3.2.1.20) (Acid maltase)	Gaa	LYAG_RAT	1,432238751	0,17176819	8,3382071	45	1,12E-10	8,84E-10
Q63532	Cornifin-A (Small praline-rich protein lA) (SPRlA) (SPRRl)	:mla Spr Sprr	SPRlA_RAT	1,44673956	0,0711711	20,3276271	12	1,15 E-10	9,lOE-10
Q64057	Alpha-aminoadipic semialdehyde dehydrogenase (Al pha -AASA dehydrogenase) (EC 1.2.1.31) (Aldehyde deh ydrogenase family 7 member Al) (EC 1.2.1.3) (An tiquitin -1) (Be ta ine aldehyde dehydrogenase) (EC 1.2.1.8) (De ltal -pi per ide ine-6-carboxy late dehydrogenase) (P6c dehydrogenase)	ldh7al Ald7a	AL7Al_RAT	-0,353973395	0,05237275	-6,7587327	191	1,64E-10	1,29E-09
P11884	Aldehyde dehydrogenase, mitochondrial (EC 1.2.1.3) (ALDH class 2) {ALDH-E2) {ALDHI)	Aldh2	ALDH2_RAT	-0,603869989	0,09151123	-6,5988622	328	1,66 E-10	1 ,30E-09
P81799	N-ace tyl- 0 -g lucos am ine kinase (N-acety l glucosamine kinase) (EC 2.7.1.59) (GlcNAc kinase)	Nagk	NAGK_RAT	-6,985472004	0,13029933	-53,610959		2,06E-10	1,60E-09
Q68FQ0	T-complex protein 1 subunit epsilon (TCP-1-epsilon) (CCT-eps il on)	Cct5	TCPE_RAT	-0,558584411	0,08519978	-6,5561719	312	2,29E-10	1,76E-09
Q5FVl4	Cell cycle exit and neuronal differentiation protein 1	Cendl	CEND_RAT	0,909400701	0,11934733	7,61978282	59	2,41E-10	1,85E-09
A2RRU1	Glycogen [starch] synthase, muscle (EC 2.4.1.11)	Gysl	GYSl_RAT	-2,305086026	0,27258298	-8,4564561	38	2,90E-10	2,20E-09
Q6P6V0	Glucose-6-phosphate isomerase (GP I) (EC 5.3.1.9) (Autocrine motility factor) (AMF) (Neuroleukin) (NLK) (Phos phoglucose isomerase) (PGt) (Phosphohexose isomerase) (PHI)	Gpi	G6PI_RAT	-0,844678689	0,12979974	-6,5075533	325	2,89E-10	2,20E-09
Q5U2Q3	Ester hydrolase Cllorf54 homolo g (EC 3.1.-.-)		CK054_RAT	-1,804338988	0,24200289	-7,4558571	64	2,94E-10	2,22E-09
P69735	Rab3 GTPase-activating protein catalytic subunit (RAB3 GTPase-activating protein 130 kDa subunit) (Rab3-GAP p130) (Rab3-GAP) (Fragments)	33 gap l Ra b	3 3 RB GP _RAT	-3,965693619	0,16223952	-24,44345	10	3,00E-10	2,25E-09
Q9QXU9	ProSAAS (Proprotein convertase subtilisin/kexin type 1 inhibitor) (Proprotein convertase 1 inh ib itor) (pro-SAAS) [Cle aved into: KEP; Big SAAS {b-SAAS); Little SAAS (I-SAAS); Big PEN-LEN (b-PEN-LEN) (SAAS CT(l-49)); PEN; PEN-20; Little LEN (I-LEN); Big LEN (b-LEN) (SAAS CT{25-40))]	Pcskln	PCSKl_RAT	0,314849995	0,04536577	6,94025485	105	3,34E-10	2,50E-09
P24090	Alpha-2-HS-glyco protein (59 kDa bone sialic acid-containing protein) (BSP) (Fetuin-A) (Glycoprotein PP63)	Ahsg Fetua	FETUA_RAT	0,706660424	0,09825493	7,19211153	75	4,05E-10	3,0lE-09
CON_002203	Phosphoglycerate kinase 1 (EC 2.7.2.3)	Pgkl Pgk-1	PGKl_RAT	-1,366340027	0,07572222	-18,044109	12	4,62E-10	3,42E-09
P16617	Phospholysine phosphoh isti dine ino rganic pyrophosphate phosphatase {EC 3.1.3. -) (EC 3.6.1.1)	Lhpp	LHPP_RAT	-0,44517297	0,07084163	-6,2840589	789	5,45E-10	4,0lE-09
Q51005	Spermatogenic leucine zipper protein 1	Spzl	SPZl_RAT	-1,693633388	0,24924728	-6,7949924	109	5,99E-10	4,38E-09
Q6AXY9	Bleomycin hydrolase (BH) (BLM hydrolase) (BMH) (EC 3.4.22.40)	Blmh	BLMH_RAT	-7,469676088	0,10937313	-68,295348		6,63E-10	4,83E-09
P70645	Sulfotransferase 1 family member 01 (STl Dl) (EC 2.8.2.-) (Dopamine sulfotransferase Sultldl) (Tyrosine-ester sulfotransferase)	Sultldl	STlDl_RAT	-3,714841139	0,36601402	-10,14945	22	9,21E-10	6,68E-09

G3V9R3									
P01946	Hemoglobin subunit alpha-1/2 (Alpha-1/2-globin) (Hemoglobin alpha-1/2 chain)	Hbal Hba-a1	HBA_RAT	1,191633525	0,04698501	25,361994		1,llE-09	7,99E-09
P09456	cAMP-dependent protein kinase type I-alpha regulatory subunit (Cleaved into: cAMP-dependent protein kinase type I-alpha regulatory subunit, N-terminally processed]	Prkarl a	KAPO_RAT	0,752250143	0,11716906	6,42021172	176	1,22E-09	8,79E-09
D3ZXK7	E3 ubiquitin-protein ligase RNF123 (EC 6.3. 2.-) (Kipl ubiquitination-promoting complex protein 1) (RING finger protein 123)	Rnf123 Kpcl	RN123_RAT	-1,451030345	0,20487629	-7,082471	63	1,44E-09	1,03E-08
P49432	Pyruvate dehydrogenase El component subunit beta, mitochondrial (PDHEl-B) (EC 1.2.4.1)	Pdhb	ODPB_RAT	-6,604336833	0,16395958	-40,280274		1,SlE-09	1,08E-08
088506	STE20/SPS1-related praline-alanine-rich protein kinase (Ste-20-related kinase) (EC 2.7.11.1) (Pancreatic serine/threonine-protein kinase) (PS/TK) (PSTKl) (Serine/threonine-protein kinase 39)	tk39 Pask Spa	STK39_RAT	-0,597283251	0,09642777	-6,1941001	364	1,58E-09	1,12E-08
P31722	Complement Clq subcomponent subunit C	Clqc Clqg	ClQC_RAT	-1,006734672	0,14641383	-6,8759536	74	1,67E-09	1,lS E-08
P04094	Proenkephalin-A [Cleaved into: Synenkephalin; Met-enkephalin (Opioid growth factor) (OGF); PENK(114-133); PENK(143-185); Met-enkephalin-Arg-Gly-Leu; Leu-enkephalin; PENK (239-260); Met-enkephalin-Arg-Phe]	1k Penk-rs Per	PENK_RAT	0,670629975	0,04178315	16,0502484	12	1,79E-09	1,25E-08
P40112	Proteasome subunit beta type-3 (EC 3.4.25.1) (Proteasome chain 13) (Proteasome component Cl0-11) (Proteasome theta chain)	Psmb3	PSB3_RAT	-2,223428027	0,12553577	-17,711511	11	1,96E-09	1,37E-08
Q51001	Glyoxalase domain-containing protein 4	Glod4	GLOD4_RAT	-1,428847224	0,21240366	-6,7270367	84	1,98E-09	1,37E-08
P82471	Guanine nucleotide-binding protein G(q) subunit alpha (Guanine nucleotide-binding protein alpha-q)	Gnaq	GNAQ_RAT	-0,693894672	0,11012115	-6,3011935	185	2,lOE-09	1,45E-08
P62864	40S ribosomal protein S30	Fau	RS30_RAT	0,536594382	0,07072032	7,58755621	42	2,12E-09	1,46E-08
P62762	Visinin-like protein 1 (VILIP) (21 kDa CABP) (Neural visinin-like protein 1) (NVL-1) (NVP-1)	Vsnll Visll	VISLl_RAT	-0,516916095	0,04330419	-11,936861	16	2,22E-09	1,52E-08
Q6XQN1	Nicotinate phosphoribosyltransferase (NAPRTase) (EC 6.3.4.21) (Nicotinate phosphoribosyltransferase domain-containing protein 1)	Naprt Naprtl	PNCB_RAT	0,592888789	0,09521589	6,22678401	228	2,27E-09	1,54E-08
Q9WVR7	Protein phosphatase lF (EC 3.1.3.16) (Ca(2+)/calmodulin-dependent protein kinase phosphatase) (CaM-kinase phosphatase) (CaMKPase) (Partner of PIX 2)	Ppmlf Popx2	PPMlF_RAT	-5,682021416	0,19918292	-28,52665		2,47E-09	1,67E-08
P07335	Creatine kinase B-type (EC 2.7.3.2) (B-CK) (Creatine kinase B chain)	Ckb Ckbb	KCRB_RAT	-0,799103618	0,12747079	-6,2689156	184	2,52E-09	1,70E-08
Q9E5M2	Hyaluronan and proteoglycan link protein 2 (Brain link protein 1)	Hapln2 Brall	HPLN2_RAT	-0,483791099	0,08028105	-6,0262179	645	2,82E-09	1,89E-08
P07323	Gamma-enolase (EC 4.2.1.11) (2-phospho-D-glycerate hydro-lyase) (Enolase 2) (Neural enolase) (Neuron-specific enolase) (NSE)	Eno2 Eno-2	ENOG_RAT	-0,679285058	0,07176434	-9,4654956	22	3,2SE-09	2,17E-08
Q66H80	Coatomer subunit delta (Archain) (Delta-coat protein) (Delta-COP)	Arcnl Copd	COPD_RAT	-0,655759558	0,10946094	-5,9908087	487	4,06E-09	2,70E-08
035244	Peroxiredoxin-6 (EC 1.11.1.15) (1-Cys peroxiredoxin) (1-Cys PRX) (Acidic calcium-independent phospholipase A2) (aiPLA2) (EC 3.1.1.-) (Antioxidant protein 2) (Non-selenium glutathione peroxidase) (NSGPx) (EC 1.11.1.9) (Thiol-specific antioxidant protein)	Aop2 Prdx6	PRDX6_RAT	-1,050367465	0,15664195	-6,7055309	69	4,53E-09	3,00E-08
P35434	ATP synthase subunit delta, mitochondrial (F-ATPase delta subunit)	AtpSd	ATPD_RAT	-0,316793795	0,05304857	-5,9717687	427	4,94E-09	3,26E-08
Q9Z2F5	C-terminal-binding protein 1 (CtBPl) (EC 1.1.1.-) (50 kDa BFA-dependent ADP-ribosylation substrate) (BARS-50) (C-terminal-binding protein 3) (CtBP3)	bpl Bars Ctb1	CTBPl_RAT	0,582228197	0,07991711	7,28540144	42	S,69E-09	3,74E-08
Q9JU3	4-trimethylaminobutyraldehyde dehydrogenase (TMABADH) (EC 1.2.1.47) (Aldehyde dehydrogenase family 9 member Al) (EC 1.2.1.3)	Aldh9al	AL9Al_RAT	-0,812098379	0,12582699	-6,4540872	88	S,75E-09	3,76E-08
QSBJP3	Ubiquitin-fold modifier 1	Ufml	UFMl_RAT	-0,343083105	0,05683538	-6,0364357	231	6,23E-09	4,0SE-08
Q6AXU4	E3 ubiquitin-protein ligase RNF181 (EC 6.3.2.-) (RING finger protein 181)	Rnf181	RN181_RAT	-0,777177283	0,11419083	-6,8059519	55	7,84E-09	S,07E-08
P04904	Glutathione S-transferase alpha-3 (EC 2.5.1.18) (GST 2-2) (GST A3-3) (GST AA) (Glutathione S-transferase Yc-1) (GST Ycl)	Gsta3 Gst Ycl	GSTA3_RAT	0,397135743	0,03646592	10,8905995	16	8,28E-09	S,33E-08
Q9E5WO	DNA damage-binding protein 1 (Damage-specific DNA-binding protein 1)	Ddbl	DDBl_RAT	-2,837297143	0,38757416	-7,3206562	38	9,09E-09	5,83E-08
035179	Endophilin-Al (Endophilin-1) (SH3 domain protein 2A) (SH3 domain-containing GRB2-like protein 2) (SH3 p4)	12 Sh3d2a Sh	SH3G2_RAT	-0,830312942	0,13576061	-6,1160078	131	1,03E-08	6,58E-08
Q5RJZ6	Short coiled-coil protein	Scoc Scoco	SCOC_RAT	-0,36467135	0,06265273	-5,8205183	323	1,41E-08	8,99E-08
Q8CG45	Aflatoxin Bl aldehyde reductase member 2 (rAFAR2) (EC 1.1.1.nll) (Succinic semialdehyde reductase) (SSA reductase)	:r7a2 Afar2 Ai	ARK72_RAT	-1,272003278	0,11595894	-10,969429	15	1,46E-08	9,24E-08
035458	Vesicular inhibitory amino acid transporter (GABA and glycine transporter) (Solute carrier family 32 member 1) (Vesicular GABA transporter) (rGVAT)	:32al Vgat Vic	VIAAT_RAT	-0,734550983	0,11992091	-6,1252952	107	1,53E-08	9,63E-08
Q9JJW1	Tetraspanin-2 (Tspan-2)	Tspan2	TSN2_RAT	-0,755990714	0,12668269	-5,9675927	156	1,56E-08	9,7SE-08
P68511	14-3-3 protein eta	Ywhah	1433F_RAT	1,709504318	0,12878144	13,2744621	12	1,56E-08	9,7SE-08
P35467	Protein SlOO-Al (S-100 protein alpha chain) (S-100 protein subunit alpha) (SlOO calcium-binding protein Al)	SlOOal	SlOAl_RAT	0,720764267	0,12366821	5,82820972	254	1,69E-08	1,0SE-07
D3ZAF6	ATP synthase subunit f, mitochondrial	Atp5j2	ATPK_RAT	-1,947754831	0,30235632	-6,4419186	62	1,97E-08	1,22E-07
P54311	Guanine nucleotide-binding protein G(l)/G(S)/G(T) subunit beta-1 (Transducin beta chain 1)	Gnbl	GBBl_RAT	-0,738997218	0,06919664	-10,67967	15	2,09E-08	1,29E-07
Q4V8B0	Oxidation resistance protein 1 (Protein C7)	Oxrl C7	OXRl_RAT	0,530121139	0,09065446	5,8477118	181	2,28E-08	1,40E-07
Q9ZOW7	Chloride intracellular channel protein 4 (Intracellular chloride ion channel protein p64Hl)	Clic4	CLIC4_RAT	0,390072441	0,0677161	5,76040952	200	3,13E-08	1,91E-07
P23565	Alpha-internexin (Alpha-lnx)	Ina lnexa	AINX_RAT	-0,749744615	0,13105991	-5,7206253	219	3,46E-08	2,ll E-07
P30009	Myristoylated alanine-rich C-kinase substrate (MARCKS) (Protein kinase C substrate 80 kDa protein)	MarcksMacs	MARCS_RAT	0,402295569	0,07007041	5,74130456	189	3,69E-08	2,24E-07
P85970	Actin-related protein 2/3 complex subunit 2 (Arp2/3 complex 34 kDa subunit) (p34-ARC)	Arpc2	ARPC2_RAT	0,991542875	0,16788639	5,90603481	111	3,90E-08	2,35E-07
P55068	Brevican core protein (Brain-enriched hyaluronan-binding protein) (BEHAB)	Bean Behab	PGCB_RAT	-0,701900704	0,12095486	-5,8029972	138	4,26E-08	2,56E-07
Q9QZ76	Myoglobin	Mb	MYG_RAT	0,505676534	0,08778311	5,76052176	136	S,3SE-08	3,20E-07
P61459	Pterin-4-alpha-carbinolamine dehydratase (PHS) (EC 4.2.1.96) (4-alpha-hydroxy-tetrahydropterin dehydratase) (Dimerization cofactor of hepatocyte nuclear factor 1-alpha) (DCoH) (Dimerization cofactor of HNFl) (Phenylalanine hydroxylase-stimulating protein) (Pterin carbinolamine dehydratase) (PCD)	:bdl Dech Pd	PHS_RAT	0,782256778	0,0708318	11,0438647	13	S,62E-08	3,3SE-07
P05369	Farnesyl pyrophosphate synthase (FPP synthase) (FPS) (EC 2.5.1.10) ((2E,6E)-farnesyl diphosphate synthase) (Cholesterol-regulated 39 kDa protein) (CR 39) (Dimethylallyltranstransferase) (EC 2.5.1.1) (Farnesyl diphosphate synthase) (Geranyltranstransferase)	Fdps	FPPS_RAT	-1,750123861	0,2142618	-8,1681563	21	S,89E-08	3,SOE-07
Q6AYR6	Haloacid dehalogenase-like hydrolase domain-containing protein 2	Hdhd2	HDH02_RAT	-1,616509859	0,27897646	-5,7944311	115	6,09E-08	3,60E-07
Q5U211	Sorting nexin-3	Snx3	SNX3_RAT	-0,869995774	0,1466216	-5,9336126	85	6,23E-08	3,67E-07
Q6AYTO	Quinone oxidoreductase (EC 1.6.5.5) (NADPH:quinone reductase) (Zeta-crystallin)	Cryz	QOR_RAT	-0,355782737	0,0600978	-5,9200622	83	7,00E-08	4,lOE-07
P63322	Ras-related protein Ral-A	Raia Ral Ral-a	RALA_RAT	-1,949356875	0,15537547	-12,546104	11	7,35E-08	4,30E-07
P14942	Glutathione S-transferase alpha-4 (EC 2.5.1.18) (GST 8-8) (GST A4-4) (GST K) (Glutathione S-transferase Yk) (GST Yk)	Gsta4 Gsta	GSTA4_RAT						
Q5FVC7	Arf-GAP with coiled-coil, ANK repeat and PH domain-containing protein 2 (Centaurin-beta-2) (Cnt-b2)	Acap2 Centb2	ACAP2_RAT						
P02401	60S acidic ribosomal protein P2	Rplp2 Rp2	RLA2_RAT	42,654048422	0,22018839	-12,053535	11	1,llE-07	6,40E-07
P57113	Maleylacetoacetate isomerase (MAAI) (EC 5.2.1.2) (GSTZl-1) (Glutathione S-transferase zeta 1) (EC 2.5.1.18)	Gstzl	MAAI_RAT	0,22714436	0,03826419	5,93621266	65	1,2SE-07	7,16E-07
Q8CGV7	Thiamine-triphosphatase (ThTPase) (EC 3.6.1.28)	Thtpa	THTPA_RAT	-0,611819798	0,06264512	-9,7664399	14	1,2SE-07	7,16E-07
P62755	40S ribosomal protein S6	Rps6	RS6_RAT	0,671761409	0,10762791	6,24151708	46	1,26E-07	7,17E-07
P11240	Cytochrome c oxidase subunit SA, mitochondrial (Cytochrome c oxidase polypeptide Va)	CoxSa	COXSA_RAT	0,770367867	0,11523583	6,68514205	32	1,52E-07	8,62E-07
		Ube2n	UBE2N_RAT	-1,328456877	0,23608224	-5,6270937	104	1,56E-07	8,8[illegible]E-07

Ubiquitin-conjugating enzyme E2 N {EC 6.3.2.19) (Bendless-like ubiquitin-conjugating enzyme) (Ubiquitin carrier protein N	) (Ubiquitin-protein ligase N)	0,278272983	0,04300892	6,47012201	43	7,57E-08	4,40E-07
		-0,625202694	0,10530348	-5,9371514	72	9,37E-08	S,43E-07

Q9EQX9	Neuromodulin {Axonal membrane protein GAP-43) (G rowth -assoc iated protein 43) {Protein Fl)	Gap43	NEUM_RAT	0,643389648	0,07311639	8,79952758	16	1,SBE-07	8,89E-07
P07936	Glu tath ione 5-transferase Mu 2 (EC 2.5.1.18) (GST 4-4) (GSTM2-2) {Glutathione 5-trans fe ra se Yb-2) (GST Yb2)	Gst m2	GSTM2_RAT	0,904283757	0,1592347	5,67893672	91	1,61E-07	9,0lE-07
P08010	Protein LZIC {Leucine zipper and CTNNBIPl doma in-co n ta i nin g protein) {Leucine zipper and ICAT homolo go us doma in -co ntaining prot ein)	Lzic	LZIC_RAT	0,994808275	0,11406346	8,72153351	16	1,78E-07	9,94E-07
QSPQN7	l nte grin beta -1 {Beta olig o dendro glia) (Be ta OL) (Fi bronect i n receptor subunit beta) {VLA-4 subunit beta) (CD antigen CD29)	lt gbl	ITBl _RAT	-0,331237899	0,05543174	-5,9755997	54	1,87E-07	1,04E -06
P49134	Tyrosine --tRNA ligase, cytoplasmic {EC 6.1.1.1) (Tyrosyl-tR NA synthetase) {TyrRS) [Cle aved into: Tyro sine --tRN A lig ase, cytoplasmic, N- term i na lly processed]	Vars	SYYC_RAT	-0,936749283	0,13400094	-6,9906175	26	2,0lE-07	1,12E-06
Q4KM49	Enoyl-CoA delta isomerase 2, mitochondrial (EC 5.3.3.8) (Delta{3),delta{2)-enoyl-CoA isomerase) (D3, D2-e no yl- CoA is omeras e) (Dodecenoyl-CoA isomerase) (Peroxi so ma l 3, 2-tra ns -e no yl- CoA isomerase) {pECI)	Eci2 Peci	ECl2_RAT	-0,36768861	0,0691463	-5,3175458	292	2,09E-07	1,16 E-06
QSXlCO	14 -3-3 protein epsilon {14-3-3E) {Mitochondr ial import stimu lat io n factor L subunit) {MSF L)	Ywhae	1433E_RAT	-1,229312789	0,13168587	-9,3351915	14	2,lBE-07	1,19 E-06
P62260	Elongation factor I-alpha 2 {EF-1-alpha-2) (Eukaryot ic elongation factor_1a A-2) {eEFlA-2) {Statin -51) 2	Eeflal Stn EFl	A 2 RAT -	0,423374396	0,08084478	5,23687959	688	2,17E-07	1,19 E-06
P62632	605 ribosomal protein L23	Rpl23	RL23_RAT	-1,201127926	0,21547401	-5,5743517	98	2,19E-07	1,20 E-06
P62832	ATP synthase subunit beta, mitochondrial (EC 3.6.3. 14)	Atp Sb	ATPB_RAT	-0,418444562	0,04892053	-8,553557	**16**	2,30E-07	1,25 E-06
P10719	Hemopexin	Hp,	HEM O_RAT	-0,486294605	0,09344175	-5,2042542	**824**	2,46E-07	1,33 E-06
P20059	Ketimine reductase mu -crys tallin {EC 1.5 .1. 25) (C DK108) (NADP -regulated th yroid -hormone -bindin g protein)	Crym	CRYM_RAT	0,44453121	0,08295014	5,35901679	180	2,54E-07	1,37 E-06
Q9QYU4	Heme oxyge nase 2 {H0 -2) (EC 1.1 4.99.3)	H mo x2	HM OX2_RAT	-0,828842031	0,1174614	-7,0562927	24	2,70E-07	1,45 E-06
P23711	Proteasome subunit beta type-10 (EC 3. 4.25.1) (Low mo le cular ma ss protein 10) {Macropain subunit MECl-1) {Multicatalytic endopeptidase co mp lex subunit MECl-1) {Proteasome MECl-1) (P rot easome su bun it beta -2i)	blO LmplO M	PSBlO_RAT	-0,801218113	0,12182849	-6,5766071	30	2,82E-07	1,51E-06
Q4KM35	Tryptophan 5-hydroxylase 2 (EC 1.1 4.16.4) (Ne u rona l tryptophan hydro xylase) {Tryptopha n 5-monooxygenase 2)	Tph2 Ntph	TPH2_RAT	-0,667938223	0,06580843	-10,149737	12	3,05E-07	1,63 E-06
Q8CGU9	Neurotrimin {GP65)	Ntm HntNt	NTRl_RAT	-0,751560045	0,08653709	-8,6848319	15	3,09E-07	1,64 E-06
Q62718	Protein S100-A4 {Metastasin) {Nerve growth factor-induced protein 42A) {P9K) (Placental calcium-binding protein) {5100 calcium-binding protein A4)	S100a4	S10A4_RAT	0,298284485	0,03599419	8,28701863	16	3,50E-07	1,86 E-06
P05942	Bifunctional ATP-dependent dihy drox yacetone kinase/FAD-AMP lyase (cyclizing) [Includes : ATP-dependent dihy dro xyace to ne kinase (DHA kinase) (EC 2.7.1.28) {EC 2.7.1.29) (G lyce rone kinase) (Trioki na se) (Triose kinase); FAD-A MP lyase {cyclizing) {EC 4.6.1.15) (FAD -AMP lyase (cy clic FMN forming)) {FMN cyclase)]	Dak	DHAK_RAT	-4,288059271	0,64361468	-6,6624634	27	3,77E-07	1,99 E-06
Q4KLZ6	Missh apen -like kinase 1 {EC 2.7.11.1) {GCK family kinase MiNK) {MAPK/ERK kinase kinase kinase 6) {MEK kin ase kinase 6) (ME KKK 6) {Misshapen/NIK-related kinase) (M itogen-ac tivated protein kinase kinase kinase kinase 6)	kl Map4k6	MINKl_RAT	-0,50510461	0,08811695	-5,732207	52	5,0BE-07	2,68E-06
F1LP90	Arfapt in -2 {ADP-ribosylation factor-interacting protein 2)	Arf i p2	ARFP 2_RAT	-0,493761315	0,0616458	-8,0096504	16	5,46E-07	2,86E-06
Q6AY65	Palmito yl- prote in thioesterase 1 {PPT-1) (EC 3.1.2 .22) (Pa lmit oyl- prote in hydro lase 1)	Pptl Ppt	PPTl_RAT	1,137138638	0,12625181	9,00690934	13	5,97E-07	3,12E-06
P45479	Trans ge lin {Smooth mu scle protein 22-alpha) (SM22-alpha)	Tagln Sm22	TAGL_RAT	-0,576508186	0,10402743	-5,5418862	63	6,27E-07	3,27E-06
P31232	Excitatory amino acid transporter 2 (G LT-1) (Sodium -dep end ent glu tamate/aspartate transporter 2) (G LU T-R) (So l ute carrier family 1 member 2)	cla2 Eaat2 GI	EAA2_RAT	-0,566096094	0,10194588	-5,5529078	59	7,04E-07	3,66E-06
P31596	MARCKS-re lated protein {Bra in protein F5 2) (MA RCKS-li ke protein 1) {Macrophage myristo ylated alanine-rich C kinase substrate) (Mac-MARCKS) {MacMARCKS)	MarcksllMlp	MRP_RAT	0,984911927	0,16886908	5,83239931	41	7,49E-07	3,87E-06
Q9EPH2	Estradiol 17-beta -deh ydro ge na se 11 (EC 1.1.1. -) (17 - beta -hydro xyste ro id deh ydro ge nase 11) {17-beta-HSD 11) (17bH SD11) (17b eta HSD11) (17 - s dl be ta-h ydro xystero id deh ydro ge nase XI) (17- beta -HSD XI) (17bet a HSDXI) (Dehydrogenase/reductase SDR fam ily member 8)	7b ll Dhr!	DHBll RAT -	0,548928658	0,06597608	8,32011661	14	8,65E-07	4,46E- 06
Q6AYS8	Reti noid -in du cib le serine carboxypeptidase (EC 3. 4.16.-) (Se ri ne ca rbox ypeptidase 1)	Scpepl Rise	RISC_RAT	-6,169637718	0,38992248	-15,822729		9,76E-07	5,0lE-06
Q920A6	Nischarin (l mida zoline receptor 1) {l- 1) (I Rl) (lmidazo li ne-1 rece ptor) {ll R)	Nisch	NISCH_RAT	-0,804700689	0,15098226	-5,3297698	65	1,32E- 06	6,76E-06
Q4G017	Guanine nu cleotide -bin ding protein G{z) subunit alpha (G(x) alpha chain) {Gz-alpha)	Gnaz	GNAZ_RAT	-1,237359864	0,21791334	-5,6782198	40	1,34E-06	6,83E-06
P19627	ADP-ribosylation factor-like protein 3 {ARD3)	Arl3	ARL3_RAT	0,519027137	0,06975008	7,44124034	16	1,40E-06	7,1lE-06
P37996	Guanine nu cleotide -bin ding protein subunit alpha-11 (G alpha-11) (G-protein subunit alpha-11)	Gnall	GNAll _RAT	-0,254596113	0,04900488	-5,1953216	83	1,44E-06	7,27E-06
Q9JlD2	Annexin AB {Annexin VIII) {Annexin -8)	Anx a8	ANXAB_RAT	-2,002022571	0,29717835	-6,7367713	20	1,49E- 06	7,51E-06
Q4FZU6	405 ribosom al protein 518	Rps18	RS18_RAT	-0,505850102	0,06374164	-7,9359441	14	1,50E-06	7,56E-06
P62271	Ciliar y neurotrophic factor receptor subunit alpha (C NTF receptor subunit alpha) {CNTFR-alpha)	Cntfr	CNTFR_RAT	0,466214621	0,08691751	5,36387484	56	1,60E- 06	8,04E- 06
Q08406	Serine -threon ine kinase receptor -assoc iated protein (UNR - interact ing protein)	Strap Un rip	STRAP_RAT	0,768997763	0,06222674	12,3579958		1,71E-06	8,55 E-06
QSXlGB	Atlastin-1{EC 3.6.5.-) {Spastic parap le g ia 3A homolog)	Atll Spg3a	ATLAl_RAT	-0,435137391	0,08854867	-4,9141042	197	1,87E- 06	9,32E-06
Q6PST4	Ras-re lated protein Rab -6A (Rab-6)	Rab6a Rab6	RAB6A_RAT	-0,823933353	0,16523003	-4,9865837	122	2,06E-06	1,02E- 05
Q9WVB1	Vimentin	Vim	VIME_RAT	-0,405845194	0,08242916	-4,9235634	160	2,lOE-06	1,04 E- 05
P31000	Cytochrome bS type B {Cytochrome bS outer mitochondrial membran e isoform)	Sb CybSm Or	CYBSB_RAT	1,508014831	0,25825189	5,83931779	29	2,48E-06	1,22E- 05
P04166	AP-2 complex subunit alpha-2 {100 kDa coated vesicle protein C) (Adaptor protein complex AP-2 subunit alpha-2) (Adaptor -re lat ed protein complex 2 subunit alpha-2) {Alpha-adapt in C) (Al pha2 -a dapt in) {Clathrin assemb ly protein complex 2 alpha-C lar ge chain) (P lasma membrane adaptor HA2/AP2 adaptin alpha C s ubun it)	Ap2a2 Adtab	AP2A2_RAT	-0,670989707	0,08836244	-7,5936074	14	2,50E-06	1,23E- 05
P18484	CD151 antigen {Platelet-endothelial tetraspan antigen 3) (PETA-3) (CD antigen CD151)	CdlSl Peta3	CDlSl_RAT	-0,443644031	0,09271328	-4,7851183	327	2,59E-06	1,27 E- 0 5
Q9QZA6	Allograft in flammator y factor 1 {AlF-1) (lo nize d calcium-binding adapter mo lecule 1) {MRF-1) {Microglia response factor)	ifl lbal Mrfl	AlFl _RAT	1,396908773	0,15895624	8,78800817	11	2,65E-06	1,29 E- 05
P41498	GMP synthase [glut am ine -hydrol yzin g] (EC 6.3.5.2) (GMP sy nthetas e) {Glutamine amidotransferase)	Gmps	GUAA_RAT	-0,474128499	0,08598317	-5,5142012	38	2,64E-06	1,29 E- 0 5
P55009	Protein NDR G2 {Antidepressant-related protein ADRG123) (N- myc downstream -regulated ge ne 2 prote in) (NDRGl -re lated prote i n)	Ndr g2	NDR G2_RAT	-0,37466149	0,04289822	-8,7337308	11	2,BlE-06	1,36 E- 05
Q4V7C6	Protein 5100-All (Calgizzarin) {5100 calcium-binding protein All)	SlOOall	Sl OAB_RAT	-0,369129019	0,0759636	-4,859288	152	2,91E-06	1,40 E- 05
Q8VBU2-2	LIM and SH3 domain protein 1 {LASP-1)	Laspl	LASPl_RAT	-1,454820512	0,1951053	-7,4565913	14	3,0BE-06	1,47 E- 05
Q6B345	Pirin {EC 1.13 .11. 24) {Probab le quercetin 2,3-dioxygenase PIR) (P robab le quercetinase)	Pir	PIR_RAT	-1,942915032	0,27810953	-6,9861504	16	3,07E-06	1,47 E- 05
Q99MZ8	Guanine nu cleotide -bind ing protein subunit beta-4 (Tra nsduc i n beta chain 4)	Gnb4	GBB4_RAT	0,320782503	0,06599065	4,86102939	139	3,llE-06	1,49E- 0 5
QSM827	M yelin protein PO {Mye lin peripheral protein) (MPP) (Myelin protein zero)	Mpz PO	MYPO_RAT	-0,654751779	0,12753333	-5,1339661	61	3,13E-06	1,49 E- 05
035353	Chloride in trace llul a r channel protein 1	Clicl	CLICl_RAT	1,461765153	0,17057349	8,56970884	11	3,38E-06	1,60 E- 05
P06907	NADH -c ytochrome bS reductase 2 (bSR. 2) (EC 1.6 .2.2)	Cyb5r2	NBSR2_RAT	0,70362844	0,1403725	5,01258043	74	3,56E-06	1,68 E- 05

Q6MG61	Ubiquitin carboxyl-terminal hydrolase 7 (EC 3. 4.19.12) (Deu biqu iti nat ing enzyme 7) (Herpesvirus-associated ubiquitin-specific protease) (r HAUSP) (Ubiquitin thioesterase 7) (Ubiquitin-specific-processing protease 7)	Usp7 Hausp	UBP7_RAT	-0,453143335	0,08348676	-5,4277272	37	3,74E-06	1,76 E-05
Q6AY12	Guanine nu cleotide -bin ding protein G(i) subunit alpha-1 (Adeny late cyclase-inhibiting G alpha protein)	Gnail Gnai-1	GNAll _RAT	-1,072397645	0,10782974	-9,9452866		3,75E-06	1,76E-05
Q4VSl4	Protein disulfide-isomerase A3 (EC 5.3.4.1) (58 kDa g lucose -regulated protein) (58 kDa microsomal protein) (p58) (Disul fide isomerase ER-60) (Endoplasmic reticulum resident protein 57) (ER protein 57) (ERp57) (Endoplasmic reticulum resident protein 60) (ER protein 60) (ERp60) (HIP-70) (Q-2)	ia3 Erp60 Grp	PDIA3_RAT	1,247107419	0,24004429	5,19532222	49	3,95E-06	1,85 E-05
P10824	Protein bassoon	Bsn	BSN_RAT	0,301429466	0,05605761	5,37713702	38	4,07E-06	1,90 E-05
CON_007152	Prosaposin receptor GPR37 (G-protein coupled receptor 37) (G- prote in coupled re cepto r CNSl)	Gpr37	GPR37_RAT	0,601473277	0,07170741	8,38788175	11	4,15E-06	1,93E-05
P11598	Prohibitin	Phb	PHB_RAT	0,302764528	0,06496832	4,66018738	378	4,39E-06	2,03E-05
088778	Thioredoxin, mitochondrial (MTRX) (Mt-Trx) (Thio redox in-2)	Txn2 Trx2	THIOM_RAT	2,120481345	0,13617682	15,5715298		4,44E-06	2,05E-05
Q9QYC6	Spectrin beta chain, non -e rythroc ytic 2 (Beta Spill sigma 1) (Be ta- Ill spectrin) (Glutamate transporter EAAT4-associated protein 41) (SPNB-3) (Spectrin-like protein GTRAP41)	Sptbn2 Spnb3	SPTN2_RAT	0,453555382	0,0630009	7,19918949	14	4,57E-06	2,llE-05
P67779	CBl cannabinoid receptor -in teract in g protein 1 (C R IP-1)	Cnripl	CNRPl_RAT	0,292003373	0,05775252	5,05611454	57	4,74E-06	2,18E-05
P97615	Transitional endoplasmic reticulum ATPase (TER ATPase) (EC 3.6.4.6) (155 Mg(2+)-ATPase p97 subunit) (Valosin -conta ining protein) (VCP)	Vcp	TERA_RAT	-0,393211817	0,0505446	-7,7795019	12	5,00E-06	2,29E-05
Q9QWN8	Enoyl-CoA delta isomerase 1, mitochondrial (EC 5.3.3.8) (3,2-tr ans-enoy l-CoA isomerase) (Delta(3),Delta(2)-enoyl-CoA isomerase) (03,02-enoyl-CoA isomerase) (Dodecenoyl-CoA isomerase)	Ecil Dci	ECIl_RAT	0,358652739	0,07407854	4,84151989	92	5,18E-06	2,36E-05
Q5M7A7	Coiled-coil domain-containing protein 91 (GGA-binding partner)	:cdc91 Ggab	CCD91_RAT	0,330788712	0,04646243	7,11948814	14	5,18E-06	2,36E-05
P46462	Acetyl-CoA acetyltransferase, mitochondrial (EC 2.3.1.9) (Acetoacetyl-CoA thiolase)	Acatl	THIL_RAT	-0,294875323	0,06437358	-4,5806884	785	5,39E-06	2,45E-05
P23965	Pantothenate kinase 4 (rPanK4) (EC 2.7.1.33) (Pantot hen ic acid kinase 4)	Pank4 Fangl	PANK4_RAT	-0,43174223	0,07833597	-5,5114173	30	5,50E-06	2,49E-05
Q6AY97	Jouberin (Abelson helper inte gration site 1 protein homolog) (AHl-1)	Ahil	AHIl_RAT	0,499425133	0,06133145	8,14305099	11	5,52E-06	2,49E-05
	Peptidyl-prolyl cis-trans isomerase FKBPlA (PPlase FKBPlA) (EC 5.2.1.8) (12 kDa FK506-binding protein) (12 kDa FKBP) (FKBP-12) (FK506-binding	Fkbpla Fkbpl	FKBlA_RAT	0,351152909	0,07572158	4,63742175	265	5,55E-06	2,49E-05
P17764	protein lA) (FKBP-lA) (lmmunophilin FKBP12) (Rotamase)	Vdacl	VDACl_RAT	0,58268755	0,09958296	5,85127778	23	5,80E-06	2,60E-05
Q92358	Voltage-dependent anion-selective channel protein 1 (VDAC-1) (rVDACl) (Outer mitochondrial membrane protein porin 1)	10 PtmblOTt	TYBlO_RAT	0,984198378	0,13516192	7,28162493	13	6,17E-06	2,75E-05
Q6DTM3	Thymosin beta-10	Rtnl Nsp	RTNl_RAT	0,533120578	0,07933393	6,71995672	15	6,87E-06	3,06E-05
Q62658	Reti c ulon -1 (Neuroendocrine-specific protein) (5- re x)	Dpp7 Dpp2	DPP2_RAT	0,178900032	0,03899046	4,58830276	191	8,08E-06	3,58E-05
Q9Z2L0	Dipeptid yl peptidase 2 (EC 3.4.14.2) (Dipept idyl aminopeptidase II) (Dipeptidyl peptidase 7) (Dipeptidyl peptidase II) (DPP II) (Quies cent cell praline dipeptidase)	Mdrl MdrH	MDRl_RAT	0,560240134	0,11053969	5,06822618	43	8,09E-06	3,58E-05
P63312	Multidru g resistance protein 1 (EC 3.6.3.44) (ATP-bind i ng cassette sub-. family B member 1) (P-glycoprotein 1) (CD antigen CD243)	1dh5al Ssadl	SSDH_RAT	-1,057023751	0,20375261	-5,1877802	34	9,80E-06	4,33E-05
Q64548-2	Succinate-semialdehyde dehydrogenase, mitochondrial (EC 1.2 .1.24) (Aldehyde dehydro ge nase family 5 member Al) (NAD(+)-dependent succinic semialdehyde dehydrogenase)								
Q9EPB1	Mevalonate kinase (MK) (EC 2.7.1.36)	**Mvk**	KIME_RAT	-0,385670388	0,08386797	-4,5985419	115	1,lOE-05	4,84E-05
P43245	Neural cell adhesion molecule 1 (N-CAM-1) (NCAM- 1) (CD antigen CD56)	Ncaml Ncam	NCAMl_RAT	-1,673599321	0,32397438	-5,1658384	33	1,13 E-05	4,96E-05
P51650	N-ace tylaspartate synthetase (NAA synthetase) (N-acetyltransferase 8-like protein) {EC 2.3.1.17)	Nat81	NAT8L_RAT	0,864574	0,13557471	6,37710366	15	1,24 E-05	5,44E-05
P17256	Cysteine-rich protein 2 (CRP-2) (Prote in ESPl)	Crip2 Crp2	CRIP2_RAT	-0,562364527	0,11814247	-4,7600538	59	1 ,30E-05	5,65E-05
P13596	Thymosin beta -4 (T beta 4) [Cle aved in to: Hematopoietic system regu lator y peptide (Seraspenide)]	b4x Thyb4 Tn	TYB4_RAT	-0,418330741	0,09415279	-4,4431052	227	1,39E-05	6,02E-05
D3ZVU9	Kinesin heavy chain isoform SA (Kinesin heavy chain neuron-specific 1) (Neuronal kinesin heavy chain) (N KHC)	Kif5a	KIFSA_RAT	-0,43611819	0,06930778	-6,2924853	15	1,44 E-05	6,25E-05
P36201	Tubu lin beta -2A chain	Tubb2a	TBB2A_RAT	-0,368901852	0,06030438	-6,1173311	16	1,49E -05	6,42E-05
P62329	Microtubule -asso cia ted protein lB (MAP-lB) (Ne ura xin) [Cle aved into: MAPlB heav y chain; MAPl light chain LCl]	Maplb	MAPlB_RAT	0,885632994	0,19754625	4,48316795	143	1 ,50E-05	6,43E-05
Q6QLM7	605 ribosomal protein Lll	Rplll	RLll_RAT	-0,761335152	0,15455133	-4,9260989	40	1 ,50E-05	6,43E-05
P85108	405 ribosom al protein 525	Rps25	R525_RAT	0,376612355	0,06016089	6,2600864	15	1 ,53E-05	6,54E-05
P15205	Ubiquitin conjugation factor E4 A	Ube4a	UBE4A_RAT	-0,148866413	0,03430623	-4,3393407	1309	1 ,54E-05	6,57E-05
P62914	NAO -de pendent protein deacylase sirtuin-5, mitochondrial (EC 3.5. 1. -) (Regulatory protein SIR2 homolog 5) (S|R2-like protein 5)	Sirt5	SIRS_RAT	0,869539931	0,18379276	4,73108923	55	1,60E -05	6,81E-05
P62853	UPF0364 protein C6orf211 homolog		CF211_RAT	-0,660178347	0,08635747	-7,6447158	10	1,75E -05	7,42E-05
Q6P7A2	Serine/arginine-rich splicing factor 2 (Splicing component, 35 kDa) {Splicing factor SC35) {SC-35) (Splicing factor, arginine/serine-rich 2)	Srsf2 Sfrs2	SRSF2_RAT	-0,448533073	0,07051559	-6,3607651	14	1,77 E-05	7,47E-05
Q68FX9	GTP-binding nuclear protein Ran (GTPase Ran) (Ras-li ke protein TC4) (Ras-related nuclear protein)	Rao	RAN_RAT	-1,317795985	0,26662188	-4,9425651	36	1,79E -05	7,55E-05
Q6AYT5	Dynactin subunit 2	Dctn2	DCTN2_RAT	-0,461897195	0,09881172	-4,6745184	55	1 ,95E-05	8,20E-05
Q6PDU1	Potassium voltage-gated channel subfamily C member 3 (KSHII ID) (Voltage-gated potassium channel subunit Kv3.3)	Kcnc3	KCNC3_RAT	0,3912338	0,07740031	5,05468015	30	2,00E-05	8,37E-05
P62828	605 ribosomal protein L27a	Rpl27a	RL27A_RAT	0,500174079	0,10657603	4,69311994	51	2,06E-05	8,61E-05
Q6AYH5	Lo w molecular weig ht phosphotyrosine protein phosphatase (LMW-PTP) {LMW-PTPase) (EC 3.1.3.48) (Lo w molecular weight cytosolic acid phosphatase) (EC 3.1.3.2)	Acpl	PPAC_RAT	-0,448661088	0,1034217	-4,3381718	222	2,18E-05	9,lOE-05
Q01956	Kinesin heavy chain isoform SC (Kinesin heavy chain neuron-specific 2) {Fragment)	Kif5c Nkhc2	KIFSC_RAT	0,623331027	0,07789002	8,00270746		2,21E-05	9,18E-05
P18445	T-comp lex protein 1 subunit delta {TCP-1-delta) (CCT-de lt a)	Cct4	TCPD_RAT	-0,506498979	0,10100558	-5,0145644	30	2,24E-05	9,22E-05
P41498-2	Inositol monophosphatase 1 (IMP 1) (IMPase 1) (EC 3.1.3.25) (lnositol-1{or 4)-monophosphatase 1) (Lithium -se nsit ive myo-inositol monophosphatase Al)	Im pal Imp	IMPAl _RAT	-0,720933522	0,08217364	-8,7732947		2,23E-05	9,22E-05
P56536	ER membrane protein complex subunit 2 (Te tratr icopept ide repeat protein 35) {TPR repeat protein 35)	Emc2 Ttc35	EMC2_RAT	-0,513667742	0,08492556	-6,0484472	15	2,23E-05	9,22E-05
Q7TPB1	405 ribosom al protein 520	Rps20	R520_RAT	0,320662618	0,0743908	4,31051421	280	2,26E-05	9,28E-05
P97697	Alpha-1-macroglobulin (Alpha-1-M) (Alpha-1-macro glob uli n 165 kDa subunit) [Cleaved into: Alpha-1-macroglobulin 45 kDa subunit]	Alm Pzp	AlM_RAT	-0,568150257	0,1285325	-4,4202848	104	2,43E-05	9,95E-05
B0BNG0	Beta-synuclein {Phosphoneuroprotein 14) (PNP 14)	Sncb	SYUB_RAT	0,26385276	0,04515734	5,84296445	16	2,50E-05	0,00010205
P60868	Eukaryotic translation initi at io n factor 5 (elF-5)	Eif5	IFS_RAT	0,414586641	0,09296531	4,45958433	84	2,53E-05	0,00010293
Q63041	lnosine triphosphate pyro phosphatase (ITPase) (lnosine triphosphatase) {EC 3.6.1.19) (Non-canonical purine NTP pyrophosphatase) (Non-standard purine NTP pyro phosphatase) (N ucle oside -t ri phosphate diphosphatase) (Nucleoside-triphosphate pyrophosphatase) (NTPase)	ltpa	ITPA_RAT	0,227738752	0,05350437	4,25645124	429	2,55E-05	0,00010377
Q63754	Elongation factor G, mitochondrial (EF-Gmt) (Elon gat io n factor G 1 , mitochondrial) {mEF-G 1) {Elongation factor Gl)	111 Efg Efgl G	EFGM_RAT	-0,504354729	0,11391687	-4,4273928	94	2,57E-05	0,00010422
Q07205	Secreto granin -1 {Chromogranin-B) (CgB) (Gluc agonoma peptide) {Secretogranin-1) {Sgl) [Cle aved into: CCB peptide short form; CCB peptide lon g form]	Chgb	SCGl_RAT	-0,463296218	0,1035846	-4,4726363	77	2,63E-05	0,00010588
D3ZW55	605 ribosomal protein L18a	Rpl18a	RL18A_RAT	-0,232001057	0,05132007	-4,5206694	66	2,62E-05	0,00010588
Q07803	NADH - ubiquinone oxidoreductase chain 3 (EC 1.6.5.3) (NAD H dehydrogenase subunit 3)	nd3 mt-Nd3 II	NU3M_RAT	-0,442661042	0,05170223	-8,5617391		2,67E-05	0,00010735
035314	CD81 antigen {26 kDa cell surface protein TAPA-1) (Target of the antiproliferative antibody 1) (CD antigen CD81)	Cd81 Tapal	CD81_RAT	0,75820881	0,15486698	4,89587147	32	2,69E-05	0,00010778
P62718	Sulfated glyco prote in 1 {SGP-1) (Prosaposin)	Psap Sgpl	SAP_RAT	0,354207721	0,07865652	4,50322164	67	2,74E-05	0,0001097

P05506	Cellular nucleic acid-binding protein (CNBP) (Zinc finger protein 9)	Cnbp Znf9	CNBP_RAT	0,394997208	0,05771609	6,843797	11	2,79E-05	0,00011079
Q62745	14-3-3 protein gamma [Cleaved into: 14-3-3 protein gamma, N-te rmina lly processed]	Ywhag	1433G_RAT	0,758642746	0,15610538	4,85981175	33	2,79E-05	0,00011079
P10960	NADH dehydrogenase [ubiquinone] 1 alpha subcomplex subunit 5 (Complex I subunit B13) (Complex l-13kD-B) (C l-13 kD- B) (NADH-ubiquinone oxidoreductase 13 kDa-B subunit)	Ndufa5	NDUA5_RAT	-0,487016389	0,11248043	-4,3297879	128	2,98E-05	0,00011832
P62634	Tissue alpha-L-fucosidase (EC 3.2.1.51) (Alpha-L-fucos idase I) (Alpha-l-fucoside fucohydrolase 1) (Alpha-L- fu cos idase 1)	Fucal Fuca	FUCO_RAT	0,787058497	0,08337741	9,43970934		3,12E-05	0,00012353
P61983	Tropomodulin-2 (Neuronal tropomodulin) (N-Tmod)	rmod2 Ntmoc	TMOD2_RAT	-0,561792814	0,13281778	-4,2298014	292	3,13E-05	0,00012355
Q63362	Serine/threonine-protein phosphatase 2A catalytic subunit beta isoform (PP2A-beta) (EC 3.1.3.16)	Ppp2cb	PP2AB_RAT	-0,720590467	0,12035984	-5,9869678	14	3,33E-05	0,00013088
P17164	Golgi reassembly-stacking protein 2 (GRS2) (Golgi reassemb ly-s tac ki ng protein of 55 kDa) (GRASP55)	Gorasp2	GORS2_RAT	-0,680968177	0,11416882	-5,9645723	14	3,46E-05	0,00013565
CON_007006	Lanosterol 14-alpha demethylase (LDM) (EC 1.1 4.13.70) (CYPU) (Cytochrome P450 51Al) (Cytochrome P450-14DM) (Cytochrome P45014DM) (Cytochrome P450U) (Sterol 14-alpha demethylase)	:yp51al Cyp5	CP51A_RAT	-1,908785438	0,30147595	-6,3314683	12	3,77E-05	0,0001473
P70566	Mitochondrial inner membrane protein (M itofilin) (Fragment)	lmmt	IMMT_RAT	0,372946152	0,08640914	4,31604985	95	3,89E-05	0,00015187
P62716	WO repeat-containing protein 61 [Cleaved into: WO repeat -conta ining protein 61, N-te rmina lly processed]	Wdr61	WDR61_RAT	0,294024108	0,05281525	5,56703066	16	4,25E-05	0,00016535
Q9R064	Amyloid beta A4 precursor protein-binding family A member I (Adapter protein Xllalpha) (Neuron-specific Xll protein) (Ne uronal Munc18-1- interacting protein 1) (Mint-1)	:>bal Mintl x:	APBAl_RAT	0,473639005	0,0834166	5,67799494		4,38E-05	0,00017001
Q64654	Large neutral amino acids transporter small subunit 1 (4F2 light chain) (4F2 LC) (4F2LC) {Integral membrane protein E16) (P rote i n TAl) (L-type amino acid transporter 1) (Solute carrier family 7 member 5)	:7a5 Mpe16 T	LATl_RAT	-0,948352992	0,13909644	-6,8179529	10	4,64E-05	0,00017939
Q3KR86	Par valbu min alpha	Pvalb Pva	PRVA_RAT	0,309968772	0,06856851	4,52057006	43	4,78E-05	0,00018452
Q4V7A0	Nucleobindin-2 {DNA-binding protein NEFA) (Prepronesfatin) [Cleaved into: Nesfatin-1]	Nucb2 Nefa	NUCB2_RAT	0,493235116	0,10978482	4,49274428	40	5,87E-05	0,00022571
035430	Calnexin	Canx	CALX_RAT	0,552674427	0,0839037	6,58700886	10	6,lSE-05	0,0002371
Q63016	Aldose 1-epimerase (EC 5.1.3.3) (Galactose mutarotase)	Galm	GALM_RAT	0,342562488	0,04885263	7,01216023		6,24E-05	0,00023886
P02625	Dystrobrevin beta (DTN-B) (Be ta-dystrobrev in)	Dtnb	DTNB_RAT	-0,439495881	0,1071589	-4,1013474	164	6,45E-05	0,00024634
Q9Jl85	Alanine--tRNA ligase, cytoplasmic (EC 6.1.1.7) (Ala nyl-tRNA synthetase) {Ala RS)	Aars	SYAC_RAT	-0,554059875	0,07324261	-7,564721		6,52E-05	0,00024814
P35565	Acylpyruvase FAHDl, mitochondrial (EC 3.7.1.5) (Fum arylacetoacetate hydrolase domain-containing protein 1)	Fahdl	FAHDl_RAT	-0,414003445	0,10221944	-4,0501439	277	6,65E-05	0,00025255
Q66HG4	Syntaxin-7	Stx7	STX7_RAT	-0,299739238	0,07021221	-4,269047	62	6,85E-05	0,00025946
P84060	Xaa-Pro dipeptidase (X-Pro dipeptidase) (EC 3.4.13.9) (lmidodipeptidase) {Peptidase D) (Praline dipeptidase) (P ro li dase)	Pepd	PEPD_RAT						
P50475	PDZ domain-containing protein GIPCl (GAIP (-terminus-interacting protein) (GLUTl C-terminal-binding protein) (GLUTlCBP) (RGS-GAIP-interacting protein) (RGS19-interacting protein 1) (Synectin)	cl Gipe Rgsl	GIPCl_RAT	-1,09744444	0,18501954	-5,9315055	12	6,91E-05	0,00026095
Q6AYQ8	Protein IMPACT {Imprinted and ancient gene protein homolog)	Impact	I MPCT_RAT	-0,19333088	0,04872361	-3,9679096	686	8,0lE-05	0,00030185
				-0,571473888	0,08956825	-6,3803175	10	8,03E-05	0,00030185
070257	Very-long-chain enoyl-CoA reductase (EC 1.3 .1.93) (Synaptic glycoprotein SC2) {Trans-2,3-enoyl-CoA reductase) (TER)	Teer Gpsn2	TECR_RAT	0,481249048	0,11389641	4,22532243	59	8,39E-05	0,00031441
Q51007	Malectin	Mlec	MLEC_RAT	-0,271269288	0,06689258	-4,0552969	132	8,51E-05	0,00031824
Q9Z254	Glyceraldehyde-3-phosphate dehydrogenase (GAPDH) (EC 1.2.1.12) (38 kDa BFA-dependent ADP-ribosylation substrate) (BARS-38) (Peptidyl-cysteine 5-nitrosylase GAPDH) (EC 2.6.99.-)	Gapdh Gapd	G3P_RAT	-0,929573752	0,17145223	-5,4217653	14	9,00E-05	0,00033564
Q5GFD9	AP-2 complex subunit mu (AP-2 mu chain) (Adaptor protein complex AP-2 subunit mu) (Adaptor-related protein complex 2 subunit mu) (Clathrin assembly protein complex 2 mu medium chain) (Clat hr in coat assembly protein AP50) (Clathrin coat-associated protein AP50) (M u2 -a dapt in) {Plasma membrane adaptor AP-2 50 kDa protein)	Ap2ml	AP2Ml_RAT	0,49193129	0,12312648	3,99533303	174	9,52E-05	0,00035419
Q64232	Kinesin-like protein KIF27	Kif27	KIF27_RAT	0,85499593	0,19286594	4,43310991	33	9,70E-05	0,00035967
Q5FVQ4	Four and a half LIM domains protein 1 (FHL-1)	Fhll	FHLl_RAT	-0,68980744	0,14534993	-4,7458394	22	9,77E-05	0,00036135
P04797	Hippocalcin-like protein 4 {Neural visinin-like protein 2) (NVL-2) {NVP-2) {Visinin-like protein 2) {VILIP-2)	Hpcal4	HPCL4_RAT	-0,643222625	0,16332955	-3,938189	305	0,00010179	0,00037566
P84092	Oligoribonuclease, mitochondrial (EC 3.1.-.-) (RN A exonuclease 2 homolog) {Small fragment nuclease)	Rexo2 Smfn	ORN_RAT	0,263144246	0,06667581	3,94662251	235	0,00010473	0,00038552
CON_007119	Hydroxyacyl-coenzyme A dehydrogenase, mitochondrial (HCDH) (EC 1.1.1.35) {Medium and short-chain L-3- hydroxyacyl -coe nzyme A dehydrogenase) (Short-cha in 3-hydroxyacyl-CoA dehydrogenase)	Had Had hsc '	HCDH_RAT	-0,606543955	0,15130656	-4,0087089	119	0,00010689	0,00039247
Q7M6Z5	Cofilin-1 {Cofilin, non-muscle isoform)	Cfll	COFl_RAT	-0,609599413	0,11450662	-5,3237046	14	0,00010745	0,00039353
Q9WUH4	Paralemmin-1 (Paralemmin)	Palm	PALM_RAT	0,607506463	0,05531931	10,9818165		0,00010892	0,0003979
P35332	Nuclear protein localization protein 4 homolog (Protein NPL4)	Nploc4 Npl4	NPL4_RAT	0,574115787	0,12764559	4,49773291	28	0,00010933	0,00039839
Q5U1Xl	Valine--tRNA ligase (EC 6.1.1.9) (Valyl-tRNA synthetase) (Val RS)	Vars Vars2	SYVC_RAT	-2,137521902	0,41358791	-5,1682407	15	0,00011451	0,00041622
Q9WVK7	V-type proton ATPase subunit F (V-ATPase subunit F) (V-ATPase 14 kDa subunit) (Vacuolar proton pump subunit F)	v lfAtp s 5 6 14	VATF_RAT	-2,605457482	0,55162423	-4,723247	21	0,0001155	0,00041771
P45592	Aconitate hydratase, mitochondrial (Aconitase) (EC 4.2.1.3) (Citrate hydro-lyase)	Aco2	ACON_RAT	-1,158054073	0,28344609	-4,0856237	70	0,00011541	0,00041771
Q920Q0	Golgi resident protein GCP60 (Acyl-CoA-binding domain-containing protein 3) (DMTl-associated protein) (OAP) (Go lgi complex-associated protein 1) (GOCAPl) (Golgi phosphoprotein 1) (GOLPHl)	Acbd3 Gcp60	GCP60_RAT	0,416281349	0,09560977	4,35396257	34	0,00011614	0,00041898
				-0,509827215	0,11926912	-4,2745952	39	0,00011946	0,00042989
Q9ES54	405 ribosomal protein 528	Rps28	RS28_RAT	-0,215315942	0,05549253	-3,8800884	328	0,00012622	0,00045306
Q04462	Electron transfer flavoprotein subunit alpha, mitochondrial (A1pha-H F)	Etfa	ETFA_RAT	-0,359776295	0,08972073	-4,0099571	85	0,00012999	0,00046547
				-0,257100744	0,06699587	-3,8375613	815	0,00013387	0,00047816
P50408	Heat shock 70 kDa protein 4 (lschemia responsive 94 kDa protein)	Hspa4 lrp94	HSP74_RAT	-0,951813888	0,1276164	-7,4583978		0,00014221	0,00050667
Q9ER34	KATS regulatory NSL complex subunit 3 (NSL complex protein NSL3) {Non- specific lethal 3 homolog)	Kans l 3 Ns l 3	KANL 3 RAT						
Q7TNY6	Protein phosphatase methylesterase 1 (PM E-1) (EC 3.1.1.89)	Ppmel Pmel	PPMEl_RAT						
P62859	605 ribosomal protein L30	Rpl30	RL30_RAT	0,271053368	0,05528028	4,90325613	16	0,00015915	0,00056564
P13803	Ribosyldihydronicotinamide dehydrogenase [quinone] (EC 1.10.99.2) {NRH dehydrogenase [quinone] 2) (NRH: qu inone oxidoreductase 2) {Quinone reductase 2) (QR2)	Nqo2	NQO2_RAT	-0,451073355	0,11778931	-3,8294932	255	0,00016168	0,00057144
088600	265 proteasome non-ATPase regulatory subunit 1 (265 proteasome regulatory subunit RPN2) (265 proteasome regulatory subunit 51) (265 proteasome subunit p112)	Psmdl	PSMDl_RAT	-0,187734162	0,0495256	-3,7906486	772	0,00016197	0,00057144
Q3KR73	Fatty aldehyde dehydrogenase (EC 1. 2.1.3) (Aldehyde dehydrogenase 4) {Aldehyde dehydrogenase family 3 member A2) (Microsomal aldehyde dehydrogenase) (msALDH)	ldh3a2 Aid hi	AL3A2_RAT	-1,168662732	0,23873264	-4,8952783	16	0,00016174	0,00057144
Q4FZT2	Copine-9 {Copine IX)	Cpne9	CPNE9_RAT						

P62890	Homeodomain-only protein (Global ischemia-induced gene 15B protein) {Gllg15b) {Odd homeobox protein 1)	i g l b 5 HodH	HOP RAT -	0,303822401	0,07729613	3,93062909	83	0,00017475	0,00061505
Q6AY80	Alpha/beta hydrolase doma in-containing protein 14B (Abhydro lase domain-containing protein 14B) (EC 3.-.-.-)	Abhd14b	ABHEB_RAT	0,242000587	0,05863846	4,12699458	40	0,00018103	0,0006356
088761	ATP-dependent (S)-NAD (P)H- hydrate dehydratase (EC 4.2.1.93) (ATP-dependent NAD(P)HX dehydratase) (Ca rbohydrate kinase domain-containing protein)	Carkd	NNRD_RAT	-1,396292558	0,06125461	-22,794899		0,00018491	0,00064763
	Phosphatidylethanolamine-binding protein 1 (PEB P-1) (23 kDa morphine-			-0,178512706	0,04707832	-3,7918243	232	0,00019075	0,00066646
P30839	binding protein) (HCNPpp) (P23K) [Cleaved into: Hippocampal cholinergic neurostimulating peptide (HCNP)]	ebpl Pbp Peb	PEBPl_RAT	-0,317677347	0,08242363	-3,8542024	104	0,00020124	0,00070144
Q5BJS7	Sideroflexin-3	Sfxn3	SFXN3_RAT	-0,244800207	0,05158664	-4,7454191	16	0,00021947	0,00076312
Q78ZRS	Vesicle-associated membrane protein 7 (VAMP-7) (Synaptobrev in-li ke protein 1)	Vamp7 Sybil	VAMP7_RAT	-0,281114117	0,04745041	-5,9243766		0,00022226	0,00077097
Q6DGG1	Endophilin-B1 {SH3 domain-containing GRB2-like protein Bl)	Sh3glbl	SHLBl_RAT	-0,449377356	0,11162546	-4,0257605	41	0,00023905	0,00082721

04AATT	COP9 signalosome complex subunit 1 (SGNl) (Signalosome sub un it 1) (G protein pathway suppressor 1) (GPS.-1) (JABl -co ntaining signaloso me subunit 1) (MFH protein)	:>s l Copsl Cs1	CSNl_RAT	-0,853152659	0,13603967	-6,2713522		0,0002401	0,00082885
P31044	Macrophage migration inhibitory factor (M IF) (EC 5.3.2. 1) (Glutath io ne-binding 13 kDa protein) (L-dopachrome isomerase) (L-dopachrome tautomerase) (EC 5.3.3.12) (Phe nyl pyruvate tautomerase)	Mif	MIF_RAT	-0,417647548	0,11227745	-3,7197812	228	0,0002511	0,00086478
Q9JHY2	Phospholemman (FXYD doma in -co ntainin g ion transport regu lator 1)	Fxydl Pim	PLM_RAT	0,298749075	0,0616307	4,84740666	14	0,00025851	0,00088818
Q9JHW5	Nuclear transport factor 2 (NTF-2)	Nutf2 Ntf2	NTF2_RAT	0,269161645	0,05099005	5,27870925	11	0,0002608	0,00089391
Q6AYE2	Ephrin type-B receptor 6 (Tyrosine -pro te in kina se-defect ive receptor EPH-6)	Ephb6	EPHB6_RAT	-0, 282352436	0,07155021	-3,9462142	46	0,00026954	0,00091949
P97834	Alpha-1-inhibi tor 3 (Alpha-1-inhibitor 3 variant II) (Alpha-I-inhibitor Ill)	Ali3 Mugl	A113_RAT	0,574121129	0,15250421	3,76462487	110	0,00026953	0,00091949
CON_000213	Beta,beta-carotene 15,15'-monooxy genase (EC 1.14 .99.36) (Beta-carotene dioxygenase 1)	1101 Bcdo Bed	BCDOl_RAT	-0,73594847	0,17683373	-4,1618104	28	0,0002719	0,00092325
P30904	Ribonuclease UK114 {EC 3.1.-.-) (14.5 kDa tran slat io nal inhibitor prote in) (Perchloric acid so ub : protein)	Hsp l 2 Pspl UK ll_ 4	RAT	-0,488534709	0,12977602	-3,764445	108	0,00027192	0,00092325
008589	Gap junction alpha-1 protein (Connex in-43) (Cx43) (Gap junction 43 kDa heart protein)	Gjal Cxn-43	CXAl_RAT	1,080953202	0,12070434	8,95537956		0,00028943	0,0009804
P61972	Seri ne protease inhibitor A3N (Serpin A3N) (CPl-2 6) (Co nt ra psin-like protease inhibitor 6) (SPl-2.2) (Seri ne protease inhibitor 3) (SPl-3)	!rp ina3n Spin	SPA3N_RAT	-0,364614318	0,09631541	-3,7856282	75	0,00030676	0,00103666
POCOK7	Sodium - and chloride-dependent GABA transporter 1 (GAT-1) (Solute carrier family 6 member 1)	l Gabtl Gat-1	SC6Al _RAT	0,693991833	0,16969236	4,08970588	29	0,00031341	0,00105666
P14046	Actin- re lated protein 2/3 comp lex subunit 1B (Ar p2/3 comp lex 41 kDa subunit) (p41-ARC)	Arpclb	ARClB_RAT	-0,680443732	0,17611731	-3,8635823	49	0,0003284	0,00110463
Q91XT5	Protein tweety homol og 1	Ttyhl	TTYHl_RAT	1,146928743	0,20627844	5,56009985		0,00035183	0,00118067
P52759	Heat shock-related 70 kDa protein 2 (Heat shock protein 70.2) (Testis-specific heat shock protein-related) (HST)	Hspa2 Hst70	H5P72_RAT	-0,311060892	0,08358129	-3,7216569	84	0,00035687	0,0011948
P08050	Eukaryotic initiation factor 4A- II (elF-4A-II) (e l F4A- II) (EC 3.6.4.13) (ATP-dependent RNA he licase elF4A-2)	Eif4a2	IF4A2_RAT	0,420482863	0,11513909	3,65195583	140	0,00036681	0,00122523
P09006	CD59 glycoprotein {MAC-inhibitory protein) {MAC- I P) (Membrane attack complex inhibition factor) (MACIF) {Protectin) (C D antigen CD59)	Cd59	CD59_RAT	-0,655304805	0,16643412	-3,9373226	35	0,00037412	0,00124678
P23978	Gelsolin {Actin-depolymerizing factor) (ADF) (Brevin)	Gsn	GELS_RAT	-0,605 20316	0,15954824	-3,7932299	54	0,00037743	0,00125491
088656	14-3-3 protein theta (14 -3-3 protein tau)	Ywhaq	1433T_RAT	-0,595432762	0,10161876	-5,8594766	8	0,00037869	0,00125621
P0C5X8	Ool ic hyt- di phosphoo li gosaccha ride--prote in glycosy lt rans ferase subunit 2 (EC 2.4.99.18) (Do lic hyl- di pho sp hoo li gosacchar ide--pro te in glycosy lt ra nsferase 63 kDa subunit) (Ribophorin II) (RPN-11) (Ribophorin-2)	Rpn2	RPN2_RAT	0,501440201	0,11778999	4,25706969	20	0,00038568	0,00127353
P14659	Multifunctional protein ADE2 (Inclu des: Phosphoribosylaminoimidazole-succinocarboxam ide synthase (EC 6.3. 2.6) (SAICAR synthetase); Phosphoribosylaminoimidazole carboxylase (EC 4.1.1.21) (AIR carboxylase) (AIRC)]	'aicsAde2 Air	PUR6_RAT	-0,48 0511161	0,13168515	-3,6489396	125	0,00038529	0,00127353
QSRKll	DnaJ homolog subfamily A member 1 (Dn aJ-like protein 1) (Heat shock protein J2) (HSJ-2)	na jal Hsj2 Rd	DNJAl_RAT	-0,407123622	0,11295084	-3,6044321	216	0,0003884	0,0012796
P27274	UDP -g lu cose 4-epimerase {EC 5.1.3.2) (G alactowa ldenase) (UDP-galactose 4-epimerase)	Ga le	GALE_RAT	0,493284287	0,12667881	3,89397628	35	0,00042378	0,00139298
Q68FP1-2	(-reactive protein	Crp Ptxl	CRP_RAT	0,643789907	0,13024131	4,94305446	11	0,00044045	0,00144448
P68255	Tumor protein p63-regulated gene I-like protein (Mossy fiber terminal-associated vertebrate-spec ific presynaptic protein)	T rgll Mover	TPRGL_RAT	0,49060336	0,13452809	3,64684703	85	0,0004567	0,00149436
P25235	Endoplasmic reticulum resident protein 29 (ERp29) (Endoplasmic reticulum resident protein 31) (ERp31)	Erp29	ER P29_RAT	-0,53744567	0,14372114	-3,7395032	51	0,00046779	0,00152719
P51583	Thioredoxin-dependent peroxide reductase, mitochondria l (EC 1.11.1.15) (PRx Ill) (Peroxiredoxin-3) (PRX-3)	Prdx3	PRDX3_RAT	-0,39092919	0,10903974	-3,5851991	135	0,00046953	0,00152941
P63036	Le ucine -r ic h repeat -conta ining protein 56	Lrrc56	LRC56_RAT	-0,836 726313	0,20254332	-4,1310981	21	0,00047492	0,00154349
P18645	Hi ppoca lci n-li ke protein 1 (Neural visinin-like protein 3) (NVL-3) (NVP-3) (Visi nin-li ke protein 3) (VIUP-3)	Hpcall	HPCLl _RAT	-0,890808221	0,22076796	-4,035043	24	0,00048224	0,00156375
P48199	Melanoma-associated antigen 01 (MAGE - 01 antigen) (Neuro trophin receptor-i ntera cting MAGE hom o lo g) (Sertoli ce ll necdin-related gene protein 1) (SNERG-1)	v1agedl Nrag1	MAGDl_RAT	0,356827456	0,09043431	3,94570872	28	0,00048596	0,0015723
A8WCF8	40S ribosomal protein S21	Rps21	RS21_RAT	-1,47491 1335	0,38604394	-3,8205789	37	0,00049259	0,00158661
PS2555	Glutamine synthetase (GS) (EC 6.3.1.2) (G lu tamat e decarboxylase) (EC 4.1.1.15) (Glu tamate--ammon ia lig ase)	Glul Gin s	GLNA_RAT	0,194703351	0,05310327	3,66650389	66	0,00049227	0,00158661
Q9ZOV6	lntegrin alpha-I (CD49 antigen-like fam ily member A) (Lam inin and co llagen receptor) (VLA-1) (CD antigen CD49a)	Itga1	ITAl_RAT	-0,43474088	0,12156454	-3,5762144	124	0,00049796	0,00160034
Q4V8C9	Proteasome sub unit beta type-7 (EC 3.4.25.1) (Macropain chain Z) (Multicatalytic endopeptidase complex chain Z) (Proteasome subunit Z)	Psmb7	PSB7_RAT	0,58132305	0,08636341	6,73112686		0,00052333	0,00167815
P62749	40S ribosomal protein S7 (S8)	Rps7	RS7_RAT	-0,3867 109 2	0,10606799	-3,6458777	64	0,00053627	0,00171583
Q9E573	Protein disulfide-isomerase A4 (EC 5.3.4.1) (Calcium-binding protein 2) (CaBP2) {Endoplasmic reticulum resident protein 70) (ER protein 70) (ERp70) {Endop lasm ic reticulum resident protein 72) (ER protein 72) (ERp 72) (ERp72)	4 Ca bp 2 Eri:PD l A_ 4	RAT	0,450911793	0,09381696	4,80629281	11	0,00054779	0,0017488
POS765	Protea so ma l ubiquitin receptor ADRMl (110 kDa cell membrane glycopro te in) (Gp ll O) (Adhes io n-regu la ti ng molecule 1) (ARM- 1) (Rpn13 homolog)	Adrml Gpll	CADRMl_RAT	0,34204678	0,0848745	4,03002988	22	0,00056056	0,00178564
P09606	Transtin-associated protein *X* (Translin -asso ciated factor *X)*	Tsna x Trax	TSNAX_RAT	0,271834358	0,07824846	3,47398999	408	0,0005677	0,00180437
P18614	ATP-binding cassette sub-family 8 member 6, mitochondrial (Ubiquitously-expressed mammalian ABC half transporter)	Abcb6 Umat	ABCB6_RAT	1,02595344	0,06638229	15,4552275		0,00058849	0,00186635
Q9JHWO	Coiled-coil doma in -containing prote in 43	Ccdc43	CCD43_RAT	-0,704341374	0,19311678	-3,6472303	55	0,00058986	0,0018666
CON_007078	Mitogen-activated protein kinase 1 (MAP kinase 1) (MAPK 1) (EC 2.7.11.24) (ERTl) (Extrace llul ar signa l- regu lated kinase 2) (ERK-2) (MAP kinase isoform p42) (p42-MAPK) (Mitogen-activated protein kinase 2) (MAP kinase 2) (MAPK 2)	l Erk2 Mapk I	MKOl_RAT	-0,644469112	0,13967799	-4,6139634	12	0,00059637	0,00188308
P62083	DNA-(apur in ic or apyrimidinic site) lyase (EC 3.1.-.-) (EC 4.2.99.18) (APEX nuclease) (APEN) (Apurinic -apyr imi din ic endonuclease 1) (AP endonuclease 1) (REF-1) (Re dox factor-I) (Cle aved into: ONA-(apurinic or xl apyrimidinic site) lyase, mitochondrial]	I Ape Apex	APEXl_RAT	-0,5936296	0,16772582	-3,5392858	93	0,00062857	0,0019804
P38659	UDP -N-acetylg lucos a m ine--peptide N-acety lglucosam inylt ransferase 110 kDa s ubun it {EC 2.4.1.255) (O-G lc NAc transferase subu nit pllO) (0-linked N-acetylglucosamine transferase 110 kDa s ubun it) (OG T)	Ogt	OGTl_RAT	-0,296573435	0,08167079	-3,6313279	52	0,00064474	0,00202252
Q9JMB 5	Gua nine nu cleot ide-bind ing protein G(I)/G(S)/G(T) subun it beta-2 {G protein subunit beta-2) (Trans ducin beta chain 2)	Gnb2	GBB2_RAT	0,624438678	0,17325592	3,60414053	59	0,0006445	0,00202252
Q9JHB5	Ne urofi la ment hea vy polypeptide (NF- H) (200 kDa neurofilament protein) (Neurofilament triple t H protein)	Nefh Nfh	NFH_RAT	-1, 2761881	0,334135	-3,8193787	29	0,00065183	0,00204032
070595	Oihydrolipoyllysine-residue acetyltransferase component of pyruvate dehydrogenase complex, mitochondrial (EC 2.3.1.12) (70 kDa mitochondrial autoantigen of primary biliary cirrhosis) (PBC) (Dihydrolipoamide ace tyltrans fe rase com ponent of pyruvate dehydrogenase comple x) (Pyruvate dehydrogenase comp le x component E2) (PDC-E2) (PDCE2)	Dlat	ODP2_RAT	0,521777165	0,09681695	5,3893164		0,00065431	0,00204366
Q5BK07	ATP-binding cassette sub-family A member 2 (ATP-bind ing cassette transporter 2) (ATP-bin din g cassette 2)	Abca2 Abc2	ABCA2_RAT	-0,800775295	0,14906676	-5,3719238		0,00066806	0,0020821
P63086	Aromatic-L-amino-acid decarboxy lase (AADC) (EC 4.1.1.28) (DOPA decarboxy lase) {DOC)	Ddc	DDC_RAT	-0,15661359	0,04555418	-3,437963	275	0,00067688	0,00210503
P43138	NADH dehydrogenase [ub iqu inone] flavoprotein 3, mitochondria l (Complex l-9kD) (Cl-9k D) (NAD H-u biq ui none ox ido re ductase 9 kDa subun it)	Ndufv3	NDUV3_RAT	0,361993717	0,0715759	5,05748072		0,00068337	0,00212066

P56558	V-type proton ATPase subunit B, brain isoform (V-ATPase subunit B 2) (Endomembrane proton pump 58 kDa subunit) (Vacuo lar proton pump vlb2 subunit B 2)	Atp6b2	VATB2_RAT	-0,714169018	0,20022563	-3,5668211	63	0,00069678	0,00215299
P54313;P543	Staph ylococc al nuclease domain-containing protein 1 {100 kDa coactivator) {SND p102) (plO0 co-activator) (p105 coactivator)	Sndl	SNDl_RAT	-0,257263957	0,04472161	-5,7525646		0,00069658	0,00215299
P16884	Lysosome-associated membrane glycoprotein 1 (LA MP-1) (Lysosome-associated membrane protein 1) (1 20 kDa lysosomal membrane glycoprotein) (LGP-120) (CD107 ant igen- like family member A) (CD antigen CD107a)	.ampl Lamp-:	LAMPl_RAT	0,467689556	0,1362678	3,43213557	251	0,00070021	0,00215566
P08461	AP-2 complex subunit sigma (Adaptor protein complex AP-2 subunit sigma) (Adaptor-related protein complex 2 subunit sigma) (Clathrin assemb ly protein 2 sigma small chain) (Clathrin coat assembly protein AP17) (Clathrin coat-associated protein AP17) (Plasma membrane adaptor AP-2 17 kDa protein) (Sigma-adaptin 3b) (Sigma2-a dapt in)	2sl Ap17 Clat	AP2Sl_RAT	-0,243565771	0,07098713	-3,4311257	256	0,00070063	0,00215566
O9ESR9	Ezrin {Cytovillin) (Villin- 2) (p81)	Ezr Vil2	EZRI_RAT	0,23017517	0,04069052	5,65672747		0,00076908	0,00236125
P14173	Cellular retinoic acid-binding protein 1 (Cellular retinoic acid-binding protein I) (CRABP-1)	Crabpl	RABPl_RAT	0,198469455	0,0556555	3,56603462	48	0,00083302	0,00255211
O6PCU8	Sentrin-specific protease 8 (EC 3.4.22.68) (Dene ddy lase -1) (NEDD8-specific protease 1) (Sentrin/SUMO-specific protease SENP8)	Senp8	SENP8_RAT	0,528204269	0,10194714	5,18115815		0,00084156	0,00257282
P62815	Alpha-mannosidase 2Cl (EC 3.2.1.24) (A1pha-D-m annos ide mannohydrolase) (Mannos idase alpha class 2C member 1)	Man2cl	MA2Cl_RAT	-0,257445311	0,07690384	-3,3476264	639	0,00086291	0,00263251
O66X93	Connector enhancer of kinase suppressor of ras 2 (Connector enhancer of KSR 2) (CNK homolog protein 2) (CNK2) (Me mbrane -assoc iated guanylate kinase-interacting protein) (M aguin)	Cnksr2	CNKR2_RAT	0,292809089	0,08648209	3,38577738	148	0,00090906	0,00276116
P14562	Unconventional myosin-ld (Myosin heavy chain myr 4)	Myold Myr4	MYO1D_RAT	0,412950659	0,11626342	3,5518536	45	0,00091082	0,00276116
P62744	Liprin- alpha-3 (Protein tyrosine phosphatase receptor type f polypeptide-interacting protein alpha-3) (PTPRF-interacting protein alpha-3)	Ppfia3	LIPA3_RAT	-0,340247231	0,09909625	-3,4335026	88	0,00091044	0,00276116
P31977	Major prion protein (PrP) (CD antigen CD230)	Prnp Prn Prp	PRIO_RAT	-1,036471968	0,20289258	-5,1084765		0,00092017	0,00278366
P62966	Guanine deaminase (Guanase) (G uanine aminase) (EC 3.5.4.3) (Guanine aminohy dro lase) (GAH)	Gda	GUAD_RAT	0,302340831	0,08621172	3,50695743	53	0,00093197	0,00281347
O5FVJ8	Aspartate aminotransferase, cytoplasm ic (cAs pAT) (EC 2.6.1.1) (EC 2.6.1.3) {Cysteine aminotransferase, cytoplasmic) (Cysteine transaminase, cytoplasmic) (cCAT) (Glutamate oxaloacetate transaminase 1) (Transaminase A)	Gotl	AATC_RAT	1,390040283	0,15900381	8,74218211		0,00094342	0,00284209
P21139	Fructose-bisphosphate aldo lase C (EC 4.1.2.13) (Br ai n-type aldolase)	Aldoc	ALDOC_RAT	-2,542069295	0,46688745	-5,4447154		0,0009614	0,0028902
O9Z1T4	cAMP-dependent protein kinase catalytic subunit beta (PKA C-beta) (EC 2.7.11.11)	Prkacb Pkacb	KAPCB_RAT	-0,715487793	0,08283431	-8,6375773		0,00098796	0,00296388
063357	Stromal interaction molecule 1	StimlSim	STIMl_RAT	-0,253052225	0,05286291	-4,7869518		0,0009916	0,00296864
091279	Leucine-rich glioma-inactivated protein 1	Lgil	LGIl_RAT	0,392945554	0,09642488	4,07514696	15	0,00099518	0,00297317
P13852	Kynurenine--o xoglut arate transaminase 3 (EC 2.6.1.7) (Cysteine-S-conjugate beta-lyase 2) (EC 4.4.1.13) (Kynuren i ne aminotransferase Ill) (KATIII) (Kynurenine--glyoxylate transam inase) (EC 2.6.1.63) (Kynurenine--oxog lutarate transaminase Ill)	Ccbl2 Kat3	KAT3_RAT	0,323563242	0,07976359	4,05652792	15	0,00103351	0,0030813
O9WTT6	Myelin-oligodendrocyte glycoprotein	Mog	MOG_RAT	-0,255178328	0,07590162	-3,3619615	119	0,00104121	0,00309786
P13221	FAS-associated factor 2 (UBX domain-containing protein 8)	Fafl Ubxd8	FAF2_RAT	-0,312725244	0,09505433	-3,2899633	596	0,00106112	0,0031506
P09117	Ubiquitin carboxy l-te rmina l hydro lase 19 (EC 3.4.19.12) (Deubiquitinating enzyme 19) (Ubi qu itin thioesterase 19) (Ub iq u iti n-specific-processing protease 19)	Usp19	UBP19_RAT	0,326009759	0,09915815	3,28777586	588	0,00107007	0,00316914
P68182	Charged multivesicular body protein 5 (Chromatin -mod ifying protein 5)	Chmp5	CHMP5_RAT	-0,634889225	0,17888316	-3,5491839	37	0,00107175	0,00316914
P84903	UBX domain-containing protein 4 (Erasin) (UBX doma i n-con ta i n i ng protein 2)	n4 Ubxd2 U b,	UBXN4_RAT	0,503842397	0,10735309	4,69331997		0,00113076	0,00333679
O8K4Y5	Cysteine sulfinic acid decarboxylase (EC 4.1.1.29) (Cystei ne -su lfinate decarboxylase) (Sulfinoalanine decarbox ylase)	Csad Csd	CSAD_RAT	0,488035881	0,1396826	3,49389185	42	0,00113577	0,00334472
O58FK9	Cu llin-3	Cul3	CUL3_RAT	0,062964584	0,01087678	5,78889787		0,00116298	0,00341787
063345	Acyl-protein thioesterase 2 (APT-2) (EC 3.1.2.-) (Lysophospholipase 2) {Lysophospholipase II) (LPL-II) (LysoPLA II)	Lypla2	LYPA2_RAT	0,280782839	0,08295687	3,38468461	66	0,00120343	0,00352957
O5BK32	Pre-B-cell leukemia transcription factor-interacting protein 1 {Hematopoietic PBX-interacting protein)	Pbxipl Hpip	PBIPl_RAT	-1,235425823	0,28681225	-4,3074374	11	0,00124044	0,00363073
O6J1Y9	Vacuolar protein sorting-associated protein 45 (rVps45)	Vps45 Vps45a	VPS45_RAT	0,873794493	0,13360435	6,54016522		0,00125111	0,00365456
Q4QQV8	Hepatoma-derived growth factor (HD GF)	Hdgf	HD GF_RAT	0,361630034	0,10662311	3,39166647	57	0,00126814	0,00369683
O5HZV0	Microtubule-associated protein R P/ EB family member 3 (EBl protein family member 3) (EBF3) (End -bin ding protein 3) (EB3) (RP3)	Mapre3	MARE3_RAT	0,42860525	0,06576056	6,51766457		0,00127088	0,00369733
064611	Phos phoglycerate mutase 2 (EC 3.1.3.13) (EC 5.4.2.11) (EC 5.4.2.4) (BPG-dependent PGAM 2) (Muscle-specific phosphoglycerate mutase) {Phosphoglycerate mutase isozyme M) (PGA M-M)	Pgam2	PGAM2_RAT	-0,301926838	0,08684321	-3,4766891	37	0,00131436	0,00381614
B5DF89	ADP- ribosy lat ion factor 4	Arf4	ARF4_RAT	-0,338365799	0,1042116	-3,246911	158	0,0014247	0,0041282
O9OYL8	Contactin-1 (Ne ura l ce ll surface protein F3)	Cntnl	CNTNl_RAT	0,839179886	0,23831773	3,52126496	29	0,00144151	0,00416853
A2VD12	Prohibitin-2 (B-cell receptor-associated protein BAP37) (BAP-37)	Phb2 Bcap37	PHB2_RAT	-0,911149205	0,25711821	-3,5436977	27	0,00145963	0,0042054
008700	Calumenin (CBP-50) (Crocalbin)	Calu	CALU_RAT	-0,320304399	0,09313601	-3,4391036	37	0,00146009	0,0042054
O8VHK7	Mitochondr ial import receptor subunit TOM22 homolog (rTOM22) {Translocase of outer membrane 22 kDa subunit homolog)	Jmm22 Tom2	TOM22_RAT	0,380726367	0,10598672	3,59220833	24	0,00146573	0,00421325
O5X\T1	Tubulin beta-3 chain (Neuron-specific class Ill beta-tubulin)	Tubb3	TBB3_RAT	0,20070516	0,06160822	3,25776591	**113**	0,00148321	0,00425102
P16290	Hematological and neurological expressed 1 protein [Cleaved into: He mato logic al and neurolog ic al expressed 1 protein, N-terminally processed]	Hal	HNl_RAT	1,185575088	0,15274012	7,76204129		0,00148476	0,00425102
P61751	ADP-ribosylation factor-like protein 8B	Arl8b	ARL8B_RAT	0,474271532	0,10573675	4,48539916		0,00152055	0,00434487
063198	Vesicular glutamate transporter 2 (VGluT2) (Different iation-assoc iated BNPI) (Differentiation-associated Na(+)- dependent inorganic phosphate cotransporter) (Solute carrier fam ily 17 member 6)	17a6 Dnpi Vgl	VGLU2_RAT	0,194266458	0,06088686	3,19061368	401	0,0015314	0,00436722
O5X\H7	Aminoacylase-lA (ACY-lA) (EC 3.5.1.14) (ACY IA) (N-acyl-L-amino-acid amidohydrolase)	Acyla Acyl	ACYlA_RAT	0,619477587	0,18162383	3,41077257	38	0,00154912	0,00440904
035783	Ga lectin-3 (Gal-3) (35 kDa lectin) (Carbohydrate -bi nd i ng protein 35) (CBP 35) (Galactose-spe cific lectin 3) {lgE-binding protein) (Lam inin- bin d ing protein) (Lectin L-29) (Mac-2 antigen)	Lgals3	LEG3_RAT	-1,39606218	0,22411848	-6,2291256		0,00156009	0,00442281
075041	Osteoclast-stimulating factor 1	Ostfl	OSTFl_RAT	0,49906346	0,09126045	5,46856218		0,00156001	0,00442281
O4ORB4	Histone Hl.4 {Hld)	-listlhle HlfL	H14_RAT	-0,477692013	0,14918828	-3,2019407	214	0,00157296	0,00445056
O6AXU6	D-dopachrome decarbo xylase (EC 4.1.1.84) (D-dopachrome tautomerase)	Ddt	DOPD_RAT	0,358917288	0,08842522	4,05899232	12	0,00158457	0,00447462
O66HA6	COP9 signalosome complex subunit 4 (SGN4) (Signalosome subunit 4) (JABl-containing signalosome subunit 4)	Cops4 Csn4	CSN4_RAT	0,431176458	0,12426841	3,46971906	30	0,00160027	0,00451013
O9Jl 2	Pe ptidyl- prolyl cis-trans isomerase FKBP4 (PPlase FKBP4) (EC 5.2.1.8) (52 kDa FK506-binding prote in) (52 kDa FKBP) (FKBP-52) (59 kDa immunophilin) {p59) (FK506-binding protein 4) (FKBP-4) (FKBP59) (HSP-binding immunophilin) {HBI) {lmmunophilin FKBP52) (Rotamase) [Cle aved into: Pe ptidyl- prolyl cis-trans isomerase FKBP4, N- te rm ina lly processed]	Fkbp4 Fkbp52	FKBP4_RAT	-0,571250419	0,16925811	-3,3750254	41	0,00162413	0,00456846
O6AYS7	ATP synthase F(0) com ple x subunit Bl, mitochondrial (ATP synthase subunit b) (ATPase subunit b)	Atp5fl Atp5f	AT5Fl_RAT	-0,167986019	0,05176208	-3,2453492	92	0,00163648	0,00459424
P08699	Paras peckle component 1	Pspcl	PSPCl_RAT	-0,345041955	0,10506841	-3,2839742	63	0,00167338	0,00467962
O6P686	Cytosolic acyl coenzyme A thioester hydrolase (EC 3.1.2.2) (ACHl) (ACT) (Acyl-CoA thioesterase 7) (Bra in acyl-CoA hydro lase) (BACH) (CTE-lla) {CTE-llb) (CTE-11) (LAC H!) (Lo ng chain acyl-CoA thioester hydrolase) (MTE-II)	Acot7 Bach	BACH_RAT	-0,785674967	0,23718796	-3,3124572	53	0,00167059	0,00467962
P15865	lg kappa chain C region, B allele		KACB_RAT	0,36198605	0,09001805	4,02126084	12	0,00169561	0,00473263

P80254	Ectonucleotide pyrophosphatase / phosphod ies terase family member 6 (E-NPP 6) (NPP-6) {EC 3.1.4.-) (EC 3. 1. 4.38) (Cho li ne -s pecific glycerophosphodiester phosphodies ter ase) (Glycerop hosphocholi ne cholinephosphodiesterase) {GPC-Cpde)	Enpp6	ENPP6_RAT	-0,272220911	0,08551932	-3,1831509	177	0,0017212	0,00479477
Q68FS2	Protein phosphatase lA (EC 3.1.3.16) (P rote in phosphatase 2C isoform la Pp alpha) {PP2C-alpha) (Protein phosphatase IA)	2cl Ppp	PPMlA_RAT	-0,287202696	0,09050577	-3,1733082	216	0,00172668	0,00480077
CON_007185	Fetuin-B {Fetuin-like protein IRL685)	Fetub	FETUB_RAT	0,520907753	0,13703896	3,80116532	15	0,00173908	0,00482595
Q9QVC8	Microtubule -assoc iated protein RP/EB family member 2	Mapre2	MARE2_RAT	0,513249643	0,16044484	3,19891639	117	0,00177526	0,00490746
P19511	Cytochrome c, somatic	Cycs	CYC_RAT	0,349567463	0,10470701	3,33852985	42	0,00177302	0,00490746
Q4KLH4	Ke lch-like protein 22	Klhl22	KLH 22_RAT	-0,825413318	0,13733004	-6,0104351		0,00183184	0,00504454
Q64559-1	605 ribosomal protein L3 {L4)	Rpl3	RL3_RAT	0,343085678	0,08623497	3,97849828	12	0,00183124	0,00504454
P01835	tRNA wybutosine-synthesizing protein 4 (tRNA yW-synthesizing protein 4) {EC 2.1.1.290) (EC 2.3.1.231) (Le ucine carboxyl methyltransferase 2) (tRNA(Phe) (7-(3-amino-3-(methoxycarbonyl)propyl)wyosine(37)-N)-methoxycarbonyltransferase) (tRN A(P he) (7-(3-amino-3-carboxypropyl)wyosine(37)-0)-methyltransferase)	Lcmt2 Tyw4	TYW4_RAT	0,31373735	0,08433398	3,72017708	16	0,00186135	0,00511604
BOBNDO	Vitamin 0-binding protein (DBP) (VDB) (Ge-g lob ulin) (G roup -spec if ic component)	Ge Dbp	VTDB_RAT	-0,482398182	0,15224426	-3,1685805	136	0,00189221	0,00518898
P20650	Serine/threonine-protein kinase 24 (EC 2.7.11.1) (M amma li an STE20-like protein kinase 3) (MST-3) (MST3b) (STE20-like kinase MST3) [Cleaved into: Serine/threonine-protein kinase 24 35 kDa subunit (M amma li an tk STE20-like protein kinase 3 N- te rmina l) (MST3/N); Serine/threonine-protein kinase 24 12 kDa subunit (Mammalian STE20-like protein kinase 3 (-terminal) {MST3/C)]	24Mst 3StkST K2	4 RAT	0,391147042	0,12320435	3,17478262	123	0,00189507	0,00518898
Q9QX79	Alpha-actinin-1 (Alpha-actinin cytoskeletal isoform) (F-act i n cross-linking protein) (Non-muscle alpha-actinin-1)	Actnl	ACTNl_RAT	0,622118988	0,13709132	4,5379895		0,00190423	0,00520416
Q3B8QO	Heterochromatin protein 1-bin ding protein 3	Hplbp3	H P1B3_RAT	0,309871937	0,0957872	3,23500358	62	0,00195251	0,00532604
P62898	405 ribosomal protein SA (37 kDa laminin receptor precursor) (37 LR P) (37/67 kDa laminin receptor) (LRP/LR) (67 kDa lam in in receptor) (67 LR) {Laminin receptor 1) (LamR) (Laminin-binding protein precursor p40) {LBP/p40)	Rpsa Lamrl	RSSA_RAT	-0,547818285	0,17315517	-3,163742	119	0,00197729	0,00538344
03ZZC3	Estradiol 17-beta -dehydrogenase 12 (EC 1.1.1.62) (17-beta-hydroxysteroid dehydrogenase 12) (17 - be ta-HSD 12) (3-ketoacyl-CoA reductase) (KAR) (EC 1.3 .1. -)	Hsd17b12	DHB12_RAT	0,473197878	0,11043905	4,28469722		0,00203586	0,00552208
P21531	405 ribosomal protein 511	Rps11	RS11_RAT	-0,768026021	0,23135488	-3,319688	37	0,00203299	0,00552208
Q5XIA3	Obg-like ATPase l	Dial	OLAl_RAT	0,691438825	0,20459287	3,37958414	28	0,00215286	0,00581755
P04276	Complexin-1 {Complexin I) (CPX I) (Synaphin-2)	Cplxl	CPLXl_RAT	0,630390437	0,13348518	4,72255002		0,00215114	0,00581755
BOLT89	Mannose -6- phosphate isomerase (EC 5.3.1.8) (Phosphohexomutase) {Phosphomannose isomerase) (PM I)	Mpi	MPl_RAT	-0,549044964	0,15933814	-3,4457849	23	0,0022	0,00593384
Q9Z1P2	Catenin beta -1 (Beta-eaten in)	Ctnnbl Catnb	CTNBl_RAT	0,517274817	0,14860535	3,48086259	21	0,00223075	0,00600555
Q6P747	WO repeat -containing protein 1	Wdrl	WDRl_RAT	1,20233396	0,32687791	3,67823561	15	0,00223662	0,00601012
P38983	605 acidic ribosomal protein Pl	Rplpl	RLAl_RAT	0,138931105	0,04413872	3,1476018	85	0,00227059	0,00609007
Q6P7R8	Dynein light chain 2, cytoplasmic {Dynein light chain LC8-type 2)	Dynll2 Dlc2	DYL2_RAT	-0,543470245	0,07850245	-6,9229715		0,00228488	0,00611703
P62282	Latexin (Endogenous carboxypeptidase inhibitor) (ECI) (Tissue carboxypeptidase inhibitor) (TCI)	urn	LXN_RAT	0,242250842	0,07534517	3,21521394	49	0,00230908	0,00617038
AOJPJ7	Protein quaking {Rqkl)	QkiQk	QKI_RAT	-0,365974488	0,11895822	-3,0764961	214	0,0023679	0,00631586
P63041	Neudesin {Neuron-derived neurotrophic factor) (SCIR PlO-re lated protein) {Spinal cord injury-related protein 10)	Nenf ScirplO	NENF_RAT	0,470910538	0,15325757	3,07267394	208	0,00240534	0,0064039
Q68FX1	Membr ane -ass ociated progesterone receptor component 1 (25-DX) (Acidic 25 kDa protein) {Ventral midline antigen) (VEMA)	:125dx Lewi I	PGRCl_RAT	-0,578990911	0,16905557	-3,4248556	22	0,00242312	0,00643935
Q9WU82	Beta-adducin {Adducin-63) (Erythrocyte adducin subunit beta)	Add2	ADDB_RAT	1,597452175	0,2833222	5,63828799		0,00243394	0,0064562
Q5RKIO	Synaptogyrin-1{p29)	Syngrl	SNGl_RAT	-0,228023057	0,07516144	-3,0337772	496	0,00254177	0,00671973
P19944	Heat shock protein HSP 90-beta (Heat shock 84 kDa) (HSP84) (HSP84)	Oabl Hsp84 f-	HS90B_RAT	-0,546927439	0,16821277	-3,2514026	35	0,0025426	0,00671973
Q78P75	F-actin-capping protein subunit alpha-1 (CapZ alpha-1)	Capzal	CAZAl_RAT	0,399964735	0,12685854	3,15284046	58	0,00256003	0,00675341
Q64361	605 ribosomal protein L19	Rpl19	RL19_RAT	-0,465165298	0,15087462	-3,0831249	112	0,00257943	0,00679219
Q91XU1	Nucleosome assembly protein 1-like 4	Napll4	NP1L4_RAT	-0,209108117	0,06666183	-3,1368494	62	0,002612	0,00685986
Q61UR5	Protein DPCD	Oped	DPCD_RAT	0,331441686	0,09710089	3,41337446	21	0,00261464	0,00685986
P70580	DnaJ homolo g subfamily B member 6 (He at shock protein J2) (HSJ-2) {Hsp40 homolo g) {MRJ) {MSJ-1)	Dnajb6	DNJB6_RAT	-0,32650515	0,10322672	-3,1629906	48	0,00270767	0,00709105
Q05764	Protein lin -7 homolog C (Lin-7C) (Mammalian lin -seven protein 3) (MALSa 3) (Vertebrate lin-7 homolog 3) (Veli-3)	c Ma s 7 Vel 3	LIN7C RAT -	0,648490647	0,11852583	5,47130233		0,00277811	0,00726234
Q62876	Stress-70 protein, mitochondrial (75 kDa glucose-regulated protein) (GRP-75) (Heat shock 70 kDa protein 9) (Mortalin) (Pe ptide -bi nd i ng protein 74) {PBP74) {mtHSP70)	a9 Grp75 Hsp	GRP75_RAT	0,312851086	0,09854453	3,1747179	42	0,00280714	0,00732496
P34058	Nucleobindin -1 (Bone 63 kDa calcium-binding protein) {CALNUC)	Nucbl Nuc	NUCBl_RAT	-0,173457097	0,0578719	-2,9972592	558	0,0028454	0,0074114
B2GUZ5	Uncharacterized protein C12orf43 homolog		CL043_RAT	-0,236488526	0,07740497	-3,055211	101	0,00287668	0,00747937
P84100	Prostaglandin reductase 2 (PRG-2) (EC 1.3 .1. 48) (15 - oxoprostag land i n 13 - reductase) (Zinc-binding alcohol dehydrogenase domain-containing protein 1)	Ptgr2 Zadhl	PTGR2_RAT	0,694247739	0,21795193	3,185325	38	0,0028866	0,00749166
Q5U2Z3	Secretogranin-3{1B1075) {Secretogranin Ill) (Sglll)	Scg3	SCG3_RAT	-0,337514874	0,11157602	-3,0249768	152	0,00292032	0,00756558
Q6AYM4	Trafficking protein particle complex subunit 3	Trappc3	TPPC3_RAT	-0,623562733	0,17802776	-3,5026152	16	0,00294722	0,00762158
Q6AYU3	Beta-2-m icroglobu lin	B2m	B2MG_RAT	0,973297826	0,27251969	3,57147711	14	0,0030673	0,0079179
079210	Arginine--tRNA ligase, cytoplasmic {EC 6.1.1.19) (Arginyl-tRNA synthetase) {ArgRS)	Rars	SYRC_RAT	0,529741307	0,14360617	3,68884781	12	0,00309829	0,00798363
P48721	0 -a mino -a cid oxidase (DAAO) (DAMOX) (DAO) (EC l.4.3.3)	Dao Daol	OXDA_RAT	0,109592702	0,03694969	2,96599751	391	0,00320217	0,0082366
Q63083	605 ribosomal protein L5	Rpl5	RLS_RAT	0,332305683	0,08054567	4,12568047		0,00331813	0,00851969
Q51034	Ubi qu itin -like modi fier -activat in g enzyme 5 (Ubiquitin-activating enzyme 5) (UFMl -activat in g enzyme) (Ubi qu itin -act ivat ing enzyme El doma i n- containing protein 1)	Jba5 Ubeldc:	UBA5_RAT	0,237968718	0,06528294	3,64518973	12	0,003356	0,00860161
Q5BK81	Clusterin {Dimeric acid glyco prote in) (DAG) (Sulfated glycoprotein 2) (SGP-2) (Testosterone repressed prostate message 2) (TRPM-2) {Cleaved into: Clusterin beta chain; Clusterin alpha chain]	Cl,	CLUS_RAT	0,255219403	0,0742426	3,43764082	16	0,00338064	0,0086494
P47868	GMP reductase 1 {EC 1.7 .1.7) (G uanosine 5'-monophosphate oxidoreductase 1) (Guanosine monophosphate reductase 1)	Gmpr Gmprl	GMPRl _RAT	-1,654127491	0,48194562	-3,432187	16	0,00341978	0,00873407
Q5U1Z2	Vesicle-associated membrane protein 3 (VAMP-3) (Ce ll ubrev in) (CEB) {Synaptobrevin-3)	Vamp3 Syb3	VAMP3_RAT	0,197918302	0,06317588	3,13281418	36	0,00343311	0,00875262
P07151	NADH dehydro ge nase [ubiquinone] 1 alpha subcomplex subunit 11 {Complex I- B1 4.7) (CI-B14.7) (NADH - ubiquinone oxidoreductase subunit B14.7)	Ndufa11	NDUAB _RAT	0,285434484	0,08129136	3,5112524	14	0,00345704	0,00879807
P40329	Ubiquitin thioesterase OTUBl (EC 3.4.19.12) (Deub iqu itin at i ng enzyme OTUBl) {OTU doma in -contain ing ubiquitin aldehyde-binding protein 1) {Otubain-1) {Ubiquitin-specific-processing protease OTUBl)	Otubl	OTUBl_RAT	-0,244419855	0,08224502	-2,9718499	128	0,00353703	0,00898579
035078	Myosin -3 {Myosin heavy chain 3) (Myos in heavy chain, fast skeletal muscle, embryonic)	Myh3	MYH3_RAT	-0,486952054	0,14399891	-3,3816372	17	0,00354591	0,00899252
P09895	Potassium voltage-gated channel subfamily A member 2 (RAK) (RBK2) {RCK5) {Voltage-gated potassium channel subunit Kvl.2)	Kcna2	KCNA2_RAT	-0,743456025	0,23317251	-3,1884377	27	0,00360234	0,00911961
Q5M7A4	405 ribosomal protein 54, X isoform	Rps4x Rps4	RS4X_RAT	-0,226915319	0,07452967	-3,0446309	52	0,00365097	0,00922654
P05371	Hs p70-bin din g protein 1 (HspBPl) (Heat shock protein-binding protein 1) {Hsp70-interacting protein 1)	spbpl HspbJ	HPBPl RAT -	0,492852051	0,16528598	2,98181405	87	0,00371621	0,00937499
Q9Z244	Prelamin -A/C [Cleaved into: Lam in -A/ C]	Lmna Lmnl	LMNA_RAT	-0,65703855	0,22205575	-2,95889	119	0,00372602	0,00937973
		Asahl	ASAHl_RAT	0,174704644	0,05811774	3,00604669	65	0,00375877	0,00943288
		Tf	TRFE_RAT	-0,284558035	0,09738602	-2,9219599	259	0,00378541	0,00948324

P63025	Trans form ing protein RhoA	10a Arha Arh; RHOA_RAT	0,27540142	0,06459726	4,26336078	0,00373109	0,00937973
Q80W89	Acid ceramidase {AC) (ACDase) (Ac id CDase) (EC 3.5.1.23) (Acylsphingosine deacylase) (N-acylsphingosine amidohydrolase)						
B2RYG6	Serotransferrin (Transferrin) (Be t a-1 meta l- bi nd i ng globulin) (Liver regeneration -re lated protein LRRG03) (Sideroph ilin)						

P12847	NSFLl cofactor p47 (XV body-associated protein XY40) (p97 cofactor p47)	Nsfllc	NSFlC_RAT	0,241318255	0,0474294	5,08794639		0,00380875	0,00952517
P63142	Nucleoside diphosphate kinase A (NOKA) (NOPkinase A) (EC 2.7.4.6) (Metastasis inhibition factor NM23) (Tumor metastatic process-associated protein)	Nmel	NDKA_RAT	0,28354422	0,08297535	3,41721043	15	0,00382045	0,00953791
P62703	NADH dehydrogenase [ubiquin one] 1 alph a subcomplex subunit 10, mitochondrial (Com pl ex I-42kD) (Cl-42kD) (NADH-ubi quin one oxidoreductase 42 kDa subunit)	NdufaIO	NDUAA_RAT	0,251325249	0,08577587	2,93002263	158	0,00389181	0,00969927
Q6 1M X7	Signal transducer and activator of transcription 3	Stat3	STAT3_RAT	-0,51495806	0,12191017	-4,2240781		0,00391676	0,00974462
P48679	Calcium -regulated heat stable protein 1 (Calcium-regulated heat -stable protein of 24 kDa) (CRHSP-24)	Carhspl	CHSPl_RAT	0,389308305	0,07746942	5,02531564		0,00401685	0,00997645
P61589	Coronin-lA (Coronin-like protein A) {Clipin-A) (Tryptophan aspartate -containing coat protein) {TACO)	:::o rol a Coro]	CORlA_RAT	0,475527829	0,1632369	2,91311484	162	0,00408407	0,01012596
Q6P751	405 ribosomal protein 523	Rps23	R523_RAT	0,236719435	0,06212071	3,8106361		0,00414907	0,01026949
P12346	ADP-ribosylation factor-like protein 1	Arll Arf7	ARll _RAT	0,493882451	0,17009798	2,90351742	148	0,00425531	0,0105144
035987	Aspartate aminotransferase , mitochondrial (mAs pAT) (EC 2.6.1.1) (EC 2.6.1.7) (Fatty acid-binding protein) (FABP-1) (Glutamate oxaloacetate transaminase 2) (Kynurenine aminotransferase 4) (Kynurenine aminotransferase IV) (Kynurenine--oxoglutarate transaminase 4) (Kynurenine--oxoglutarate transaminase IV) {Plasma membrane-associated fatty acid-binding protein) (FABPpm) (Transaminase A)	Got2 Maat	AATM_RAT	0,192883383	0,06692865	2,88192548	276	0,00426311	0,01051566
Q05982	T-complex protein 1 subunit alpha (TCP-1-alpha) (CCT-alpha)	fcpl Cctl Cct;:	TCPA_RAT	-0,5 42240843	0,16346197	-3,3172294	16	0,00435819	0,01073185
Q56150	Alpha-2-macroglobulin receptor-associated protein (Alpha-2-MRAP) (Gp330-binding 45 kDa protein) (Low density lipoprotein receptor-related protein-associated protein 1) (RAP)	Lrpapl	AMRP_RAT	-0,424450367	0,14585384	-2,9101075	91	0,00454162	0,0111645
P52631	Coatomer subunit gamma -1 (Gamma -1-coat protein) (Gamma -1-COP)	Copgl Copg	COPGl _RAT	-0,460710929	0,15710624	-2,9324801	69	0,0045595	0,01118938
Q9WU49	Retinal-binding protein 1 (Cellular retinal-binding protein) (CRBP) (Cellular retinol-binding protein I) (CRBP-1)	Rbpl Rbp-1	RETl_RAT	-0,488980665	0,16279022	-3,0037472	40	0,004584	0,0112304
Q91ZN1	Solute carrier family 2, facilitated glucose transporter member 3 {Glucose transporter type 3, brain) {GLUT-3)	2 3 Gu ł 3 G li GTR 3	RAT	-0, 281593781	0,09699581	-2,903154	92	0,00462401	0,01130924
P62268	405 ribosomal protein 515 (RIG protein)	Rps15 Rig	R515_RAT	0,426889238	0,14359764	2,97281504	46	0,00468363	0,01143565
P61212	Citrate lyase subunit beta-like protein, mitochondrial (Citrate lyase beta-like) (EC 4.1.-.-)	Clybl	CLYBL_RAT	-0,485790539	0,15836071	-3,0676203	**28**	0,00474882	0,01157524
P00507	V-type proton ATPase 16 kDa proteolipid subunit (V-ATPase 16 kDa proteolipid subunit) (Vacuolar proton pump 16 kDa proteolipid subunit)	0cAtp c Atp[6] f	VATL_ RAT	0,190663089	0,06744019	2,82714339	402	0,0049312	0,01199953
P28480	Swi5-dependent recombination DNA repair protein 1 homolog (Liver regeneration-related protein LRRGT00030) (Meiosis protein 5 homolog)	frl Mei 5 Meir	SFRl _RAT	0,148810063	0,05273945	2,82160839	488	0,00497322	0,01206937
Q99068	Coiled-coil domain-containing protein 134	Ccdc134	CC134_RAT	0,340056235	0,09208914	3,69268562		0,00497663	0,01206937
CON_007008	Delta-1-pyrrolin e-5-carboxylate dehydrogenase, mitochondrial {P5C dehydrogenase) (EC 1 . 2.1.88) (Aldehyde dehydrogenase family 4 member Al) (L-glutamate gamma-semialdehyde dehydrogenase)	Aldh4al	AL4Al_RAT	-0, 720285244	0,24644674	-2,9226811	55	0,00502809	0,01217372
Q4AEF8	Cytochrome b-cl complex subunit 2, mitochondria l (Complex Ill subunit 2) (Core protein II) (Ubiquinol-cytochrome -c reductase complex core protein 2)	Uqcrc2	QCR2_RAT	-0,455695703	0,15389327	-2,9611153	**41**	0,00507861	0,01227542
P02696	Tropomyosin alpha-4 chain (Tropomyosin -4) (TM- 4)	Tpm4	TPM4_RAT	-0,5675289 77	0,10241831	-5,5412841		0,00518616	0,01251444
Q07647	AP 2-associated protein kinase 1 (EC 2.7.11.1) (Adaptor-associated kinase 1)	Aakl	AAKl_RAT	-1,68543 26	0,35694448	-4,7218341		0,00523406	0,01255605
P62845	Eukaryotic translation initiation factor 2 subunit 3 (Eukaryotic translation initiation factor 2 subunit gamma) (eIF-2-gamma) (PP42)	Eif2s3 Eif2g	IF2G_RAT	0,341680943	0,07993825	4,27431106		0,00523821	0,01255 605
Q510K3	Guanine nucleotide-binding protein subunit beta-2-like 1 (Receptor for activated C kinase) (Receptor of activated protein kinase C 1) (RACKl) (Cleaved into : Guanine nucleotide-binding protein subunit beta-2- like 1, N-terminally processed]	Gnb2 l1	GBLP_RAT	-0,406695957	0,11441791	-3,5544781	10	0,0052291	0,01255605
P63081	Trifunctional enzyme subunit alpha , mitochondrial (TP-alpha) (Includes: Long-chain enoyl-CoA hydratase (EC 4.2.1.17); long chain 3-hydroxyacyl-CoA dehydrogenase (EC 1.1. 1 .211)]	Hadha	ECHA_RAT	0,391995245	0,13047127	3,00445652	31	0,00522933	0,01255605
Q6TXG9	Carbonic anhydrase 1 (EC 4.2.1.1) (Carbonate dehydratase I) (Carbonic anhydrase I) (CA-I)	Cal Carl	CAHl _RAT	0,757282862	0,13759971	5,50352094		0,00531584	0,01272098
Q5M862	Nudc domain-containing protein 2	Nud cd2	NUDC2_RAT	-0, 725273177	0,1991649	-3,6415713		0,0053877	0,0128716
POC2X9	405 ribosomal protein 527	Rps27 527-1	R527_RAT	-0,593511 201	0,20454483	-2,9016191	49	0,00554752	0,01323153
P32551	FACT complex subunit SSRPl (Facilitates chromatin transcription complex subunit SSRPl) (Recombination signal sequence recognition protein 1) (Structure-specific recognition protein 1) (T160)	Ssrpl Ciidbp	SSRPl_RAT	0,178611766	0,06353636	2,81117386	**151**	0,00559139	0,01331072
P09495	Copper transport protein ATOXl (ATXl homolog protein Rahl) (Metal transport protein ATXl)	Atoxl Rahl	ATOXl_RAT	0,291230259	0,10231402	2,84643553	81	0,00559917	0,01331072
POC1X8	Regulating synaptic membrane exocytosis protein 3 (Nim3) (RIM3 gamma) (Rab-3-interacting molecule 3) (RIM 3)	Rims3 Nim3	RIMS3_RAT	0,134423065	0,04848337	2,77256	384	0,00583272	0,01382038
P81795	COP9 signalosome complex subunit 3 (SGN3) (Signalosome subunit 3)	Cops3 Csn3	CSN3_RAT	0,129278224	0,04586219	2,81884106	98	0,00583188	0,01382038
P63245	Neurochondrin (Neurite outgrowth-related protein from the rat brain) (Norbin)	Ncdn	NCDN_RAT	0,383122461	0,1366075	2,80454934	110	0,00595867	0,01409568
Q64428	5-oxoprolinase (EC 3.5.2.9) (5-oxo -L- prolinase) {5-0Pase) (Pyroglutamase)	Oplah	OPLA_RAT	-0,24448 1042	0,08781693	-2,7839854	163	0,00600485	0,0141585
BOBNN3	Serine/ threonine- protein phosphatase 2A catalytic subunit alpha isoform (PP2A -alpha) (EC 3.1.3.16)	Ppp2ca	PP2AA_RAT	0,247770687	0,0880163	2,8150546	89	0,00600385	0,0141585
Q5M823	Fatty acid-binding protein, heart (Fatty acid-binding protein 3) (Heart-type fatty acid-binding protein) (H- FABP)	Fabp3	FABPH_RAT	0,374678204	0,12763401	2,9355672	32	0,00611892	0,01440393
Q71TY3	COP9 signalosome complex subunit 8 (SGN8) (Signalosome subunit 8) (COP9 homolog) (JABl-containing signalosome subunit 8)	Cops8 Csn8	CSN8_RAT	0,295037647	0,09642721	3,05969276	20	0,00618434	0,01453421
Q04931	CaM kinase-like vesicle-associated protein (1G5)	Camkv 1G5	CAM KV_RAT	-0,20553520 7	0,06528349	-3,1483491	16	0,0062167	0,0145865
Q9WUC4	Nicastrin	Ncstn	NlCA_RAT	-0,767926715	0,26338534	-2,9156016	34	0,006243	0,01462442
Q9JIR3	Secretogranin -2 (Chromogranin-C) (Secretogranin II) (Sg11) (Cleaved into: Secretoneurin (SN)]	cg2 Chge Scg-	SCG2_RAT	0,46666443	0,10395317	4,48917928		0,0064637	0,01511688
Q68FW9	Serine/threonine- protein kinase PAK 3 {EC 2.7.11.1) (Beta-PAK) (p21-activated kinase 3) (PAK-3) (p65-PAK)	Pak3	PAK3_RAT	-0,4860 10193	0,17401039	-2,7929953	78	0,00656672	0,01533298
035095	Translation initiation factor eIF-2B subunit beta (eIF-2B GDP-GTP exchange factor subunit beta)	Eif2b2 Eif2bb	El2BB_RAT	-0,3042486	0,11141597	-2,730745 1	326	0,00666284	0,01553227
P97608	Complement C3 [Cleaved into: Complement C3 beta chain ; Complement C3 alpha chain (EN CF-1) (Neutrophil chemotactic factor-I); Neutrophil chemotactic factor-2 (EN CF-2); C3a anaphylatoxin; Acylation stimulating protein (ASP) (C3 adesArg); Complement C3b alpha' chain; Complement C3c alpha ' chain fragment 1; Complement C3dg fragment ; Complement C3g fragment; Complement C3d fragment ; Complement C3f fragment; Complement C3c alpha' chain fragment 2]	C3	C0 3_RAT	-0,217871016	0,07291886	-2,9878554	22	0,00678276	0,01578632
P63331	General vesicular transport factor p115 (Protein USOl homolog) (Transcytosis-associated protein) (TAP) (Vesicle- docking protein)	Usol Vdp	USOl_RAT	0,155236883	0,05008185	3,09966329	16	0,00688473	0,01599786
P07483	Calcium/ calmodulin-dependent protein kinase type II subunit alpha (CaM kinase II subunit alpha) (CaMK-11 subunit alpha) (EC 2.7.11.17)	Camk2a	KCC2A_RAT	0,48463028	0,17214146	2,81530247	51	0,00690993	0,0160306
Q6P4Z9	Microtubule-associated protein RP/EB family member 1 (APC-binding protein EBl) (End-binding protein 1) (EB1)	Maprel	MAREl_RAT	-0,218 131759	0,07883443	-2,7669606	83	0,00697412	0,01614136
Q63092	Eukaryotic peptide chain release factor subunit 1 (Eukaryotic release factor 1) (eRFl)	Etfl	ERFl _RAT	0,197359769	0,07036016	2,80499332	54	0,00698005	0,01614136
Q8CGU6	405 ribosomal protein 516	Rps16	RS16_RAT	-0,459586639	0,14393858	-3,1929357	13	0,007063	0,01630704
P10362	605 ribosomal protein l34	Rpl34	Rl34_RAT	0,236022594	0,08698991	2,7132 1813	194	0,00726288	0,01674175

Q62829	Fibroblast growth factor 1 (FGF-1) (Acidic fibroblast growth factor) (aFGF) (Heparin-binding growth factor 1) (HBGF-1)	:gflFgf | Fgfc	FGFl_RAT -						
Q62818	Phenylalanine--tRNA ligase alpha subunit (EC 6.1.1.20) (Phenylalanyl-tRNA synthetase alpha subunit) (PheRS)	Farsa Farsla	SYFA_RAT	0,174180566	0,06311761	2,75961901	74	0,00728959	0,01677651
	8-oxo-dGDP phosphatase NUDT18 (EC 3.6.1.58) (2-hydroxy-dADP			0,264175693	0,09180621	2,8775361	30	0,00731596	0,0168104
P01026	phosphatase) (7,8-dihydro-8-oxoguanine phosphatase) (Muff homolog 3) (Nucleoside diphosphate-linked moiety X motif 18) (Nudix motif 18)	Nudt18 Mth3	NUD18_RAT	0,175050858	0,06494161	2,69551164	328	0,00739038	0,01695439
P41542	Acylamino-acid-releasing enzyme (AARE) (EC 3.4.19.1) (Acyl-peptide hydrolase) (APH) (Acylaminoacyl-peptidase)	Apeh	ACPH_RAT	0,232506896	0,08610914	2,70014203	195	0,00754041	0,01726712
P11275	Gastrin-releasing peptide (GRP) [Cleaved into: Neuromedin-C (GRP-10))	G,p	GRP_RAT	-0,528784507	0,18901502	-2,7975793	45	0,00755062	0,01726712
Q66HR2	ADP/ATP translocase 1 (ADP,ATP carrier protein 1) (Adenine nucleotide translocator 1) (ANT 1) (Solute carrier family 25 member 4)	S lc 25 a 4 Antl	ADTl_RAT	0,218412696	0,07552985	2,89174013	26	0,00764196	0,01744834
QSU2Q7	Ubiquitin-like-conjugating enzyme ATG3 (EC 6.3.2.-) (Autophagy-related protein 3) (APG3-like) (Preconditioning-inducible gene 1 protein)	Atg3 Apg31	ATG3_RAT	-0,911555643	0,28955028	-3,1481774	13	0,0076986	0,0175311
P62250	BAG family molecular chaperone regulator 5 (BAG-5) (Bcl-2-associated athanogene 5)	Bags	BAGS_RAT	0,21575346	0,07855658	2,74647208	68	0,00770251	0,0175311
P11250	Small ubiquitin-related modifier 1 (SUMO-1)	Sumol	SUMOl_RAT	0,177400305	0,05648197	3,14083062	13	0,00780826	0,01774382
P61149	6-phosphogluconolactonase (6PGL) (EC 3.1.1.31)	Pgls	6PGL_RAT	-0,319174737	0,11374619	-2,8060257	38	0,00786641	0,01784785
Q505J8	Zinc transporter 3 (ZnT-3) (Solute carrier family 30 member 3)	Slc30a3 Znt3	ZNT3_RAT	-0,263921561	0,0960585	-2,7475087	57	0,0080252	0,01817953
Q641Y7	Dynein light chain roadblock-type 1 (Bithoraxoid-like protein) (BLP) {robl/LC7-like protein} {Dynein light chain 2A, cytoplasmic} {Dynein-associated protein Km23)	rbl Dncl2a Dr	DLRBl_RAT	0,214016225	0,07039983	3,0400107		0,00827024	0,01870526
P13676	L [illegible] protein]-leucine -carboxy methyltransferase 1)	Lcmtl	LCMTl_RAT	-0,219453208	0,08223553	-2,6685935	171	0,00835015	0,01885644
P24393	cGMP-dependent 3',5'-cyclic phosphodiesterase (EC 3.1.4.17) (Cyclic GMP-stimulated phosphodiesterase) (CGS-PDE) (cGSPDE)	Pde2a	PDE2A_RAT	-0,416761758	0,10799607	-3,8590457		0,00837157	0,01887527
CON_007044	Isocitrate dehydrogenase [NAO] subunit alpha, mitochondrial (EC 1. .1.41) (Isocitric dehydrogenase subunit alpha) (NAD(+)-specific ICDH subunit alpha)	ldh3a	IDH3A_RAT	0,167379699	0,0596434	2,80634082	32	0,00845841	0,01904132
Q05962	C tochrome b-cl complex subunit 1, mitochondrial (Complex Ill subunit 1) (Core protein I) (Ubiquinol-cytochrome-c reductase complex core protein 1)	Uqcrcl	QCRl_RAT	0,14330937	0,05340459	2,68346545	87	0,00872105	0,019602
Q6AZ50	ATP-sensitive inward rectifier potassium channel 10 (ATP-sensitive inward rectifier potassium channel KAB-2) (BIRlO) (Brain-specific inwardly rectifying K(+) channel 1) (BIRK!) (Inward rectifier K(+) channel Kir4.1) (Potassium channel, inwardly rectifying subfamily J member 10)	KcnjlOKa b-2	KCJl0_RAT	-0,555043561	0,20092238	-2,7624775	36	0,00897757	0,02014718
QSQJC9	Neurofibromin (Neurofibromatosis-related protein NF-1)	Nfl	NFl_RAT	-0,17080815	0,06120394	-2,790803	30	0,00905434	0,0202879
QSI0H3	Nucleolar protein 3 (Apoptosis repressor with CARD)	Nol3 Arc	NOL3_RAT	0,450682765	0,14351318	3,14035804	11	0,0094024	0,02102974
P85971	Sortilin {Glycoprotein 110) (Gp110) (Neurotensin receptor 3) (NTR3)	Sortl	SORT_RAT	-0,195914265	0,0744833	-2,6303114	151	0,00941456	0,02102974
Q6QIX3	NEDD8-activating enzyme El catalytic subunit (EC 6.3.2.-) (NEDD8-activating enzyme ElC) (Ubiquitin-activating enzyme ElC) (Ubiquitin-like modifier-activating enzyme 3) (Ubiquitin-activating enzyme 3)	Uba3 Ubelc	UBA3_RAT	0,191161427	0,06289972	3,03914612	13	0,00949624	0,0211794
P62628	Acidic leucine -rich nuclear phosphoprotein 32 family member E	Anp32e	AN32E_RAT	-0,319965258	0,12124918	-2,6389066	81	0,00997267	0,02220766
Q6P4Z6	Endophilin-B2 (SH3 domain-containing GRB2-like protein B2)	Sh3glb2	SHLB2_RAT	-0,27095088	0,08589579	-3,1544141	10	0,01025571	0,02280276
Q01062	Mitogen-activated protein kinase 10 (MAP kinase 10) (MAPK 10) (EC 2.7.11.24) {SAPK-beta) (Stress-activated protein kinase JNK3) (c-Jun N-terminal kinase 3) (p54-beta)	ik10Jnk3 Prk1	MKlO_RAT	0,848853848	0,24404168	3,47831509		0,01028965	0,02281364
Q99NA5	Ubiquitin carboxyl-terminal hydrolase isozyme L3 (UCH-L3) (EC 3.4.19.12) (Ubiquitin thioesterase L3)	Uch13	UCHL3_RAT						
Q68FY0	Thioredoxin (Trx)	Txn Txnl	THIO_RAT	[illegible]	[illegible]	[illegible]	[illegible]	[illegible]	[illegible]
P49655	Serine--tRNA ligase, cytoplasmic (EC 6.1.1.11) (Seryl-tRNA synthetase) (SerRS) {Seryl-tRNA{Ser/Sec) synthetase)	Sars Sarsl	SYSC_RAT	-0,525409903	0,20002519	-2,6267187	59	0,01096739	0,02423574
P97526	605 ribosomal protein L7a	Rpl7a	RL7A_RAT	0,339740071	0,12256834	2,77184202	22	0,01112346	0,02454305
Q62881	Interferon-inducible double-stranded RNA-dependent protein kinase activator A {Protein activator of the interferon-induced protein kinase) (Protein kinase, interferon-inducible double-stranded RNA-dependent activator)	Prkra	PRKRA_RAT	-0,33335769	0,1280708	-2,6029171	70	0,01127559	0,02484072
				-0,26936759	0,09803325	-2,7477166	23	0,01146376	0,02521679
054861	Calcium-dependent secretion activator 1 (Calcium-dependent activator protein for secretion 1) (CAPS-1) {rCAPS)	dps Caps Cap	CAPSl_RAT	-0,233410156	0,08864761	-2,6330114	45	0,01155739	0,02538405
Q99Ml7	Methionine adenosyltransferase 2 subunit beta (Methionine adenosyltransferase II beta) (MAT II beta)	Mat2b	MAT2B_RAT						
QSXIEO	Nucleosome assembly protein 1-like 1 (NAP-1-related protein)	Naplll Nrp	NPlll_RAT	-0,292900215	0,08203601	-3,5703859		0,0117787	0,02575253
QSPPJ9	Calcium/calmodulin-dependent protein kinase kinase 2 (CaM-KK 2) (CaM-kinase kinase 2) {CaMKK 2) (EC 2.7.11.17) (Calcium/calmodulin-dependent protein kinase kinase beta) (CaM-KK beta) (CaM-kinase kinase beta) (CaMKK beta)	Camkk2	KKCC2_RAT	-0,236389565	0,09288056	-2,5450919	183	0,01175024	0,02575253
P49187	Rho-related GTP-binding protein RhoB	Rhob Arhb	RHOB_RAT	-0,684229472	0,25745613	-2,6576546	35	0,01177802	0,02575253
Q91Y78	Histidine triad nucleotide-binding protein 3 (HINT-3) (EC 3.-.-.-) (HINT-4)	Hint3 Hint4	HINT3_RAT	0,307763374	0,11956233	2,57408316	83	0,01182665	0,02581823
P11232	Beta-enolase (EC 4.2.1.11) (2-phospho-0-glycerate hydro-lyase) (Enolase 3) (Muscle-specific enolase) (MSE) (Skeletal muscle enolase)	Eno3 Eno-3	ENOB_RAT	-0,345198235	0,13335887	-2,5884911	53	0,01241421	0,02705997
Q6P799	Glycolipid transfer protein (GLTP)	GLTP	GLTP_RAT	-0,203348076	0,08083987	-2,5154429	284	0,01244033	0,02707601
P62425	3-mercaptopyruvate sulfurtransferase (MST) (EC 2.8.1.2)	Mpst	THTM_RAT	-0,259825192	0,1005285	-2,5845923	52	0,01259554	0,02737254
Q4V8C7	ZWl0 interactor (SNAP25-interacting protein 30) (Scoilin) (ZWlO-interacting protein 1) {Zwint-1)	Zwint Sip30	ZWINT_RAT	-0,897613472	0,33517545	-2,6780406	26	0,01266272	0,02747715
Q62717	605 ribosomal protein L14	Rpl14	RL14_RAT	-0,177864562	0,07130884	-2,4942848	530	0,01292463	0,02800336
QSU2R0	WO repeat -containing protein 7 (TGF-beta resistance-associated protein TRAG)	Wdr7Trag	WDR7_RAT	-0,437219229	0,16511992	-2,6478891	28	0,01315288	0,02845518
Q9Z2G8	Transgelin-2	Tagln2	TAGL2_RAT	-0,540591655	0,20558511	-2,6295272	30	0,01335771	0,02881193
088831	Heat shock protein beta-1 (HspBl) (Heat shock 27 kDa protein) (HSP 27)	Hspbl Hsp27	HSPBl_RAT	-0,502823592	0,19318161	-2,6028543	36	0,01334222	0,02881193
CON_007131	Ribonuclease inhibitor {Ribonuclease/angiogenin inhibitor 1)	Rnhl Rnh	RINI_RAT	-0,255755693	0,09975609	-2,5638103	49	0,01347134	0,02901366
P62747	Serine/threonine-protein phosphatase 2B catalytic subunit beta isoform (EC 3.1.3.16) {CAM-PRP catalytic subunit) (Calmodulin-dependent calcineurin A subunit beta isoform)	Ppp3cb	PP2BB_RAT	-0,636084548	0,22737897	-2,7974643		0,01352876	0,02909389
Q8K3P7	Eukaryotic translation initiation factor 2 subunit 1 (Eukaryotic translation initiation factor 2 subunit alpha) (eIF-2-alpha) (eIF-2A) (eIF-2alpha)	Eif2sl Eif2a	IF2A_RAT	0,568763771	0,21199007	2,68297369	22	0,01358743	0,02917658
P15429	Coatomer subunit beta' (Beta'-coat protein) (Beta'-COP) (p102)	Copb2	COPB2_RAT	0,576572523	0,19658647	2,93292069	11	0,01361909	0,02920111
B0BNM9	Importin subunit beta-1 (Karyopherin subunit beta-1) (Nuclear factor p97) {Pore targeting complex 97 kDa subunit) (PTAC97)	Kpnbl	IMBl_RAT	-0,338285198	0,13455165	-2,5141661	82	0,01388699	0,02973135
	van Willebrand factor A domain-containing protein SA (Loss of			-0,319863316	0,12732093	-2,5122603	79	0,01403333	0,03000015
P97532	heterozygosity 11 chromosomal region 2 gene A protein homolog) (Mast cell surface antigen 1) {Masa-1)	a Lohllcr2a	WWASA_RAT	0,263640754	0,10379756	2,53995146		0,01417603	0,03026037
QSXFX0	Alanyl-tRNA editing protein Aarsdl (Alanyl-tRNA synthetase domain-containing protein 1)								
Q8VIL3 P42930	F-actin-capping protein subunit alpha-2 (CapZ alpha-2)	Capza2	CAZA2_RAT	[illegible]	[illegible]	[illegible]		[illegible]	[illegible]
Q63507 P29315	ATP synthase subunit e, mitochondrial (ATPase subunit e)	Atp5i	ATPSI_RAT	0,864862305	[illegible]	[illegible]		[illegible]	0,03124934
Q9ERH3 P20651	Glycerol-3-phosphate dehydrogenase [NAO(+)], cytoplasmic (GPD-C) (GPDH-C) (EC 1.1.1.8)	Gpdl	GPDA_RAT	-1,420056822	0,49123821	[illegible]	11	0,0146856	0,03124934
	Syntaxin-12 {Syntaxin-13)	Stx12 Stx13	STX12_RAT	-0,254321956	0,10336292	-2,4604757	172	0,01486317	0,03154053
		Albg	AlBG_RAT	0,158116902	0,06445312	2,45320802	193	0,01504654	0,03188276

Alpha-<br>lB-<br>glycopr<br>otein<br>{Alpha-<br>1-B<br>glycopr<br>ote in)<br>(C44)<br>(Liver<br>regener<br>ation-<br>related<br>protein<br>1)<br>Sorbitol<br>dehydro<br>genase<br>(EC<br>1.1.1.14)<br>(L-<br>iditol 2-<br>dehydro<br>genase)	Aarsdl	AASDl_RAT							
P68101	Protein prune homolo g (EC 3.6.1.1)	Prune	PRUNE_RAT	-0,352736023	0,13814435	-2,5533873	35	0,01518226	0,03210206
035142	405 ribosomal protein 517	Rps17	RS17_RAT	0,179770028	0,0720853	2,49385139	64	0,01523113	0,03213235
P52296	Calcyclin-binding protein {CacyBP)	Cacybp	CYBP_RAT	-0,154331477	0,06315505	-2,4436917	220	0,01532535	0,03228392

Q75WE7	Thiopurine 5-methyltransferase (EC 2.1.1.67) (Thiopurine methyltransferase)	Tpmt	TPMT_RAT	-0,595174846	0,18714726	-3,1802487		0,01548529	0,03257328
Q5XI97	Microtubule-associated protein lA (MAP-lA) [Cleaved into: MAPlA heavy chain; MAPl light chain LC2]	,1apla Mtapl	MAPlA_RAT	-0,170914154	0,06825659	-2,5039948	50	0,01558832	0,03274227
Q3T1K5	Cytochrome b-cl complex subunit 6, mitochondrial (Complex Ill subunit 6) (Complex Ill subunit VIII) (Cytochrome cl non-heme 11 kDa protein) (Mitochondrial hinge protein) (Ubiquinol-cytochrome c reductase complex 11 kDa protein)	Uqcrh	QCR6_RAT	-0,295351525	0,12079166	-2,4451317	143	0,0156953	0,03291907
P29419	ADP-ribosylation factor 5	Arf5	ARF5_RAT	0,274074903	0,1051561	2,60636243	23	0,01578338	0,03305575
035077	Fatty acid synthase (EC 2.3.1.85) [Includes: [Acyl-carrier-protein] 5-acetyltransferase (EC 2.3.1.38); [Acyl-carrier-protein] 5-malonyltransferase (EC 2.3.1.39); 3-oxoacyl-(acyl-carrier-protein) synthase (EC 2.3.1.41); 3-oxoacyl-[acyl-carrier-protein] reductase (EC 1.1.1.100); 3-hydroxyacyl-[acyl-carrier-protein] dehydratase (EC 4.2.1.59); Enoyl-[acyl-carrier-protein] reductase (EC 1.3.1.39); Oleoyl-[acyl-carrier-protein] hydrolase (EC 3.1.2.14))	Fasn	FAS_RAT	-0,195253979	0,08053797	-2,4243717	366	0,01581854	0,03308137
G3V7Pl	Renin receptor (ATPase H(+)-transporting lysosomal accessory protein 2) (ATPase H(+)-transporting lysosomal-interacting protein 2) (Renin/prorenin receptor)	tp6ap2 Atp6i	RENR_RAT	-0,530510726	0,21202148	-2,5021556	47	0,0158816	0,03311812
CON_150456	Serine/threonine-protein phosphatase CPPEDl (EC 3.1.3.16) (Calcineurin-like phosphoesterase domain-containing protein 1)	::ppedl Cstpl	CPPED RAT -	0,313231747	0,11615777	2,69660599	16	0,01588201	0,03311812
Q9EPH1	Very long-chain specific acyl-CoA dehydrogenase, mitochondrial (VLCAD) {EC 1.3.8.9)	Acadvt Vlcad	ACADV RAT -	0,455616375	0,15414941	2,95568036		0,0160719	0,03346574
P27867	Aldehyde dehydrogenase X, mitochondrial (EC 1.2.1.3) (Aldehyde dehydrogenase family 1 member Bl)	ldhlbl Aldhl	ALlBl_RAT	-0,140878861	0,05743739	-2,4527379	88	0,01615045	0,03358083
Q6AYG3	Peptidyl-prolyl cis-trans isomerase A (PPlase A) (EC 5.2.1.8) (Cyclophilin A) (Cyclosporin A-binding protein) (Rotamase A) (p1B15) (p31) [Cleaved into: Peptidyl-prolyl cis-trans isomerase A, N-terminally processed]	Ppia	PPIA_RAT	-1,026549251	0,41426067	-2,4780273	53	0,01643122	0,03411547
P04644	lg gamma-2A chain C region	lgg-2a	lGG2A_RAT	0,191437003	0,07665152	2,49749783	41	0,01661383	0,03444504
Q6AYK6	Tropomodulin-1 (Erythrocyte tropomodulin) (E-Tmod)	Tmodl	TmodTMODl_RAT	0,138353678	0,05707646	2,42400596	123	0,01680543	0,03479231
Q9ZOTO	Protein NDRG3 (N-myc downstream-regulated gene 3 protein)	Ndrg3 Ndr3	NDRG3_RAT	-0,451226958	0,14506339	-3,1105503		0,01706805	0,03528538
P34926	Potassium/sodium hyperpolarization-activated cyclic nucleotide-gated channel 2	Hcn2	HCN2_RAT	-0,097981231	0,04105224	-2,3867448	1746	0,0171042	0,03530954
Q5M915	Band 4.1-like protein 1 (Neuronal protein 4.1) (4.1N)	ib4111 Epb4.1	E41Ll_RAT	0,138133642	0,0517206	2,67076641	15	0,01745141	0,03597483
P84083	605 ribosomal protein L12	Rpl12	RL12_RAT	-0,212628132	0,07128544	-2,982771		0,01752615	0,03607737
P12785	Up-regulated during skeletal muscle growth protein 5 (Diabetes-associated protein in insulin-sensitive tissues)	Usmg5 Dapit	USMG5_RAT	0,113316534	0,04792312	2,36454818	1042	0,01823462	0,03748227
Q6AXS4	Profilin-2 {Profilin II)	Pfn2	PROF2_RAT	0,177458663	0,06660104	2,66450287	14	0,01849759	0,03796875
Q66H71	Tumor susceptibility gene 101 protein (ESCRT-I complex subunit TSGlOl)	TsglOl	TSlOl_RAT	-0,213067076	0,08648909	-2,4635138	35	0,01881854	0,03857265
P45953	ATP-binding cassette sub-family D member 3 (70 kDa peroxisomal membrane protein) (PMP70)	d3 Pmp70 Pxr	ABCD3_RAT	-0,290351927	0,12050819	-2,4093957	60	0,01906418	0,03902072
Q66HF8	Glycerophosphodiester phosphodiesterase domain-containing protein 1 {EC 3.1.-.-)	Gdpdl	GDPD1_RAT	0,272301013	0,11302796	2,4091473	55	0,01936544	0,0395812
PlOlll	Tubulin beta-4B chain (Tubulin beta-2C chain)	ubb4b Tubb2	TBB4B_RAT	0,291264439	0,12418557	2,34539676	327	0,01960413	0,03995588
P20760	Prenylcysteine oxidase (EC 1.8.3.5) (Chloride ion pump-associated 55 kDa protein)	Pcyoxl Clp55	PCYOX_RAT	-0,253191612	0,09275805	-2,7295918	11	0,01959263	0,03995588
P70567	Ras-related protein Rab-18	Rab18	RAB18_RAT	0,544184155	0,18772899	2,8987753		0,0199291	0,04053139
Q6AYR2	Neuronal cell adhesion molecule (Nr-CAM) (Ankyrin-binding cell adhesion molecule NrCAM) (Neuronal surface protein Bravo) (rBravo) (NgCAM-related cell adhesion molecule) (Ng-CAM-related)	Nrcam	NRCAM_RAT	0,130241536	0,05455853	2,38718905	64	0,01994268	0,04053139
Q9JKA9	Ubiquitin-associated protein 1 (UBAP-1)	Ubapl	UBAPl_RAT	-0,322830367	0,12180823	-2,6503165	13	0,01999971	0,04059013
Q9WTP0-2	Syntaxin-binding protein 1 (N-Secl) (Protein unc-18 homolog 1) (Unc18-1) (Protein unc-18 homolog A) (Unc-18A) (p67) (rbSecl)	;txbplUnc l 8	STXBl RAT -	-0,405088514	0,1707883	-2,3718751	81	0,02007062	0,04061978
P23358	Tubulin beta-2B chain (T beta-15)	ubb2b Tubb2	TBB2B_RAT	0,202777398	0,08608114	2,3556541	125	0,02004618	0,04061978
CON_007041	Eukaryotic translation initiation factor 3 subunit A (elF3a) (Eukaryotic translation initiation factor 3 subunit 10) (elF-3-theta)	Eif3a Eif3s10	EIF3A_RAT	0,226262266	0,09448745	2,3946278	54	0,02013728	0,04066643
Q9JJW3	Tumor protein 054 (Tumor protein 052-like 2)	Tpd5212	TPD54_RAT	-0,333024088	0,10616731	-3,1367856		0,02015003	0,04066643
Q9EPC6	Serine/threonine-protein phosphatase 2A 55 kDa regulatory subunit B alpha isoform {PP2A subunit B isoform B55-alpha) (PP2A subunit B isoform BRA) {PP2A subunit B isoform PR55-alpha) (PP2A subunit B isoform R2-alpha) {PP2A subunit B isoform alpha)	Ppp2r2a	2ABA_RAT	-0,465369333	0,19741279	-2,3573414	107	0,02022446	0,04075964
Q6IRE4	405 ribosomal protein 512	Rps12	RS12_RAT	-0,291389598	0,07825379	-3,7236484		0,0204095	0,04107518
P16970	Myelin basic protein {MBP)	Mbp	MBP_RAT	-0,547870555	0,21514627	-2,5465027	16	0,02155511	0,04332036
QOVGK4	Isochorismatase domain-containing protein 1 (Down-regulated in nephrectomized rat kidney protein 1) (DR-NRl)	Isocl	lSOCl_RAT	-0,178275262	0,05440289	-3,2769445		0,02202877	0,04414932
Q6P9T8				-0,395887535	0,17007311	-2,3277491	96	0,02202552	0,04414932
Q99ML5				-0,243750764	0,10347303	-2,3556937	52	0,02229419	0,0446193
Q5EB77				-0,19616684	0,0840023	-2,3352556	64	0,02267754	0,04532367
P97686-3				0,22432532	0,08765851	2,55908201	14	0,02271365	0,04533305
Q5XIS7				0,30306513	0,08418058	3,60017881		0,02275452	0,04535189
P61765-2				-0,497404945	0,21385536	-2,3258942	66	0,02310613	0,04598917
Q3KRE8				0,192801638	0,07690648	2,50696231	16	0,02334481	0,04640023
Q1JU68				-0,414841694	0,17827435	-2,3269847	56	0,0236078	0,04685839
Q6PCT3				-0,272301233	0,11929886	-2,2825133	150	0,0238647	0,04730324
P36876				-0,210861846	0,08931768	-2,3608074	32	0,02449309	0,0484822
P63324				0,177700879	0,07797156	2,2790473	111	0,02457669	0,04858104
P02688-5				0,843074733	0,30535253	2,76098824		0,02463714	0,04863392
Q6I7R3				-0,334387421	0,1458994	-2,291904	69	0,02496726	0,04921825

Supplementary Table S3: Signatures for GRP78-promoted neuroprotection

Entry	Protein names	Gene names	Entry name
A8WCF8	Tumor protein p63-regulated gene 1-like protein (Mossy fiber terminal-associated vertebrate-specific presynaptic protein)	Tprgll Mover	TPRGL_RAT
BOBNFI	Septin-8	Sept8	SEPT8_RAT
BOK020	CDGSH iron-sulfur domain-containing protein 1 (MitoNEET)	Cisdl	CISDI_RAT
B1H267	Sorting nexin-5	Snx5	SNX5_RAT
B2RYG6	Ubiquitin thioesterase OTUBI (EC 3.4.19.12) (Deubiquitinating enzyme OTUBI) (OTU domain-containing ubiquitin aldehyde-binding protein 1) (Otubain-1) (Ubiquitin-specific-processing protease OTUBI)	Otubl	OTUBI_RAT
B2RYW9	Fumarylacetoacetate hydrolase domain-containing protein 2 (EC 3.-.-.-)	Fahd2 Fahd2a	FAHD2_RAT
B4F7AI	Complex III assembly factor LYRM7 (LYR motif-containing protein 7)	Lyrm7 MZMIL	LYRM7_RAT
D3ZVR7	Prostamide/prostaglandin F synthase (Prostamide/PG F synthase) (Prostamide/PGF synthase) (EC 1.11.1.20)	Fam213b	PGFS_RAT
F1M386	Rap guanine nucleotide exchange factor 2 (Cyclic nucleotide ras GEF) (CNrasGEF) (Neural RAP guanine nucleotide exchange protein) (nRap GEP) (PDZ domain-containing guanine nucleotide exchange factor 1) (PDZ-GEFI) (RA-GEF-1) (Ras/Rapl-associating GEF-1)	Rapgef2 Pdzgefl	RPGF2_RAT
008730	Glycogenin-1 (GN-1) (GNI) (EC 2.4.1.186)	Gygl Gyg	GLYG_RAT
035112	CD166 antigen (Activated leukocyte cell adhesion molecule) (HB2) (KG-CAM) (Protein MEMO) (SB-10 antigen) (CD antigen CD166)	Alcam	CD166_RAT
035814	Stress-induced-phosphoprotein 1 (STIl) (Hsc70/Hsp90-organizing protein) (Hop)	Stipl Hop	STIPI_RAT
035824	DnaJ homolog subfamily A member 2 (RDJ2)	Dnaja2	DNJA2_RAT
035854	Branched-chain-amino-acid aminotransferase, mitochondrial (BCAT(m)) (EC 2.6.1.42)	Bcat2 Bcatm Eca40	BCAT2_RAT
035964	Endophilin-A2 (Endophilin-2) (SH3 domain protein 2B) (SH3 domain-containing GRB2-like protein 1) (SH3p8)	Sh3gll Sh3p8	SH3GI_RAT
070351	3-hydroxyacyl-CoA dehydrogenase type-2 (EC 1.1.1.35) (17-beta-hydroxysteroid dehydrogenase 10) (17-beta-HSD 10) (EC 1.1.1.51) (3-hydroxy-2-methylbutyryl-CoA dehydrogenase) (EC 1.1.1.178) (3-hydroxyacyl-CoA dehydrogenase type II) (Endoplasmic reticulum-associated amyloid beta-peptide-binding protein) (Mitochondrial ribonuclease P protein 2) (Mitochondrial RNase P protein 2) (Type II HADH)	Hsd17b10 Erab Hadh2	HCD2_RAT
070513	Galectin-3-binding protein (Cyp-C-associated protein) (CyCAP) (Lectin galactoside-binding soluble 3-binding protein) (Mac-2-binding protein) (MAC2BP) (Mac-2 BP) (Protein 90K)	Lgals3bp	LG3BP_RAT
088267	Acyl-coenzyme A thioesterase 1 (Acyl-CoA thioesterase 1) (EC 3.1.2.2) (CTE-I) (Inducible cytosolic acyl-coenzyme A thioester hydrolase) (LACH2) (ACH2) (Long chain acyl-CoA thioester hydrolase) (Long chain acyl-CoA hydrolase)	Acotl Ctel	ACOTI_RAT
088902	Tyrosine-protein phosphatase non-receptor type 23 (EC 3.1.3.48) (His domain-containing protein tyrosine phosphatase) (HD-PTP) (Protein tyrosine phosphatase TD14) (PTP-TD14) (Fragment)	Ptpn23	PTN23_RAT
P00406	Cytochrome c oxidase subunit 2 (Cytochrome c oxidase polypeptide II)	Mtco2 Coii mt-Co2	COX2_RAT
P01048	T-kininogen 1 (Alpha-1-MAP) (Major acute phase protein) (T-kininogen I) (Thiostatin) [Cleaved into : T-kininogen 1 heavy chain (T-kininogen I heavy chain); T-kinin; T-kininogen 1 light chain (T-kininogen I light chain)]	Mapl	KNTI_RAT
P02793	Ferritin light chain 1 (Ferritin L subunit 1)	Ftll Ftl	FRILI_RAT
P04642	L-lactate dehydrogenase A chain (LDH-A) (EC 1.1.1.27) **(LOH** muscle subunit) (LDH-M)	Ldha Ldh-1 Ldhl	LDHA_RAT
P04692	Tropomyosin alpha-1 chain (Alpha-tropomyosin) (Tropomyosin-1)	Tpml Alpha-tm Tpma	TPMI_RAT
P04906	Glutathione S-transferase P (EC 2.5.1.18) (Chain 7) (GST 7-7) (GST class-pi)	Gstpl	GSTPI_RAT
P05426	60S ribosomal protein L7	Rpl7	RL7_RAT
P05696	Protein kinase C alpha type (PKC-A) (PKC-alpha) (EC 2.7.11.13)	Prkca Pkca	KPCA_RAT
P06238	Alpha-2-macroglobulin (Alpha-2-M)	A2m A2ml	A2MG_RAT

P06686	Sodium/potassium-transporting ATPase subunit alpha-2 (Na(+)/K(+) ATPase alpha-2 subunit) (EC 3.6.3.9) (Na(+)/K(+) ATPase alpha(+) subunit) (Sodium pump subunit alpha-2)	Atp1a2	AT1A2_RAT
P07153	Dolichyl-diphosphooligosaccharide--protein glycosyltransferase subunit 1 (EC 2.4.99.18) (Dolichyl-diphosphooligosaccharide--protein glycosyltransferase 67 kDa subunit) (Ribophorin I) (RPN-1) (Ribophorin-1)	Rpnl	RPN1_RAT
P07340	Sodium/potassium-transporting ATPase subunit beta-1 (Sodium/potassium-dependent ATPase subunit beta-1)	Atp1b1	AT1B1_RAT
P07895	Superoxide dismutase [Mn], mitochondrial (EC 1.1 5.1.1)	Sod2	SODM_RAT
P08009	Glutathione 5-transferase Yb-3 (EC 2.5.1.18) (Chain 4) (GST Yb3) (GST class-mu 3)	Gstm3	GSTM4_RAT
P08011	Microsomal glutathione 5-transferase 1 (Microsomal GST-1) (EC 2.5.1.18) (Microsomal GST-1)	Mgstl Gst12	MGST1_RAT
P08081-2	Clathrin light chain A (Lea)	Clta	CLCA_RAT
P08082	Clathrin light chain B (Lcb)	Cltb	CLCB_RAT
P08932	T-kininogen 2 (Alpha-1-MAP) (Major acute phase protein) (T-kininogen II) (Thiostatin) [Cleaved into: T-kininogen 2 heavy chain (T-kininogen II heavy chain); T-kinin; T-kininogen 2 light chain (T-kininogen II light chain)]		KNT2_RAT
P10686	1-phosphatidylinositol 4,5-bisphosphate phosphodiesterase gamma-1 (EC 3.1.4.11) (Phosphoinositide phospholipase C-gamma-1) (Phospholipase C-gamma-1) (PLC-gamma-1)	Plcgl	PLCG1_RAT
P10860	Glutamate dehydrogenase 1, mitochondrial (GDH 1) (EC 1.4.1.3) (Memory-related gene 2 protein) (MRG-2)	Gludl Glud	DHE3_RAT
P10888	Cytochrome c oxidase subunit 4 isoform 1, mitochondrial (Cytochrome c oxidase polypeptide IV) (Cytochrome c oxidase subunit IV isoform 1) (COX IV-1)	Cox4i1 Cox4 Cox4a	COX41_RAT
P11608	ATP synthase protein 8 (A6L) (Chargerin II) (F-ATPase subunit 8)	Mt-atp8 Atp8 Atpase8 Mtatp8	ATP8_RAT
P11980-2	Pyruvate kinase PKM (EC 2.7.1.40) (Pyruvate kinase muscle isozyme)	Pkm Pkm2 Pykm	KPYM_RAT
P12001	605 ribosomal protein L18	Rpl18	RL18_RAT
P12007	Isovaleryl-CoA dehydrogenase, mitochondrial (IVD) (EC 1.3.8.4)	Ivd	IVD_RAT
P13233	2',3'-cyclic-nucleotide 3'-phosphodiesterase (CNP) (CNPase) (EC 3.1.4.37)	Cnp Cnpl	CN37_RAT
P13383	Nucleolin (Protein C23)	Ncl Nuc	NUCL_RAT
P13638	Sodium/potassium-transporting ATPase subunit beta-2 (Sodium/potassium-dependent ATPase subunit beta-2)	Atp1b2	AT1B2_RAT
P14604	Enoyl-CoA hydratase, mitochondrial (EC 4.2.1.17) (Enoyl-CoA hydratase 1) (Short-chain enoyl-CoA hydratase) (SCEH)	Echsl	ECHM_RAT
P14841	Cystatin-C (Cystatin-3)	Cst3	CYTC_RAT
P15589	Steryl-sulfatase (EC 3.1.6.2) (Arylsulfatase C) (ASC) (Steroid sulfatase) (Steryl-sulfate sulfohydrolase)	Sts	STS_RAT
P15650	Long-chain specific acyl-CoA dehydrogenase, mitochondrial (LCAD) (EC 1.3.8.8)	Acadl	ACADL_RAT
P15999	ATP synthase subunit alpha, mitochondrial	Atp5a1	ATPA_RAT
P16036	Phosphate carrier protein , mitochondrial (Phosphate transport protein) (PTP) (Solute carrier family 25 member 3)	Slc25a3 Phc	MPCP_RAT
P17074	405 ribosomal protein 519	Rps19	RS19_RAT
P17077	605 ribosomal protein L9	Rpl9	RL9_RAT
P17475	Alpha-1-antiproteinase (Alpha-1-antitrypsin) (Alpha-1-proteinase inhibitor) (Serpin AI)	Serpinal	A1AT_RAT
P18088	Glutamate decarboxylase 1 (EC 4.1.1.15) (67 kDa glutamic acid decarboxylase) (GAD-67) (Glutamate decarboxylase 67 kDa isoform)	Gadl Gad67	DCE1_RAT
P19234	NADH dehydrogenase [ubiquinone] flavoprotein 2, mitochondrial (EC 1.6.5.3) (EC 1.6.99.3) (NADH-ubiquinone oxidoreductase 24 kDa subunit)	Ndufv2	NDUV2_RAT
P19527	Neurofilament light polypeptide (NF-L) (68 kDa neurofilament protein) (Neurofilament triplet L protein)	Nefl Nf68 Nfl	NFL_RAT
P19945	605 acidic ribosomal protein PO (605 ribosomal protein L10E)	Rplp0 Arbp	RLA0_RAT
P20156	Neurosecretory protein VGF (VGF8a protein) [Cleaved into : VGF(24-63); VGF(180-194) ; VGF(375-407); Neuroendocrine regulatory peptide-1 (N ERP -1); Neuroendocrine regulatory peptide-2 (N ERP -2); TLQP-11 ; TLQP-21 ; TLQP-30; TLQP-62; HFHH-10; AQ EE-30; LQEQ-19]	Vgf	VGF_RAT

P20788	Cytochrome b-cl complex subunit Rieske, mitochondrial (EC 1.10 .2.2) (Complex III subunit 5) (Cytochrome b-cl complex subunit 5) (Liver regeneration-related protein LRRGT00195) (Rieske iron-sulfur protein) (RISP) (Ubiquinol-cytochrome c reductase iron-sulfur subunit) [Cleaved into : Cytochrome b-cl complex subunit 11 (Complex III subunit IX) (Ubiquinol-cytochrome c reductase 8 kDa protein)]	Uqcrfsl	UCRI_RAT
P21396	Amine oxidase [flavin-containing] **A** (EC 1.4.3.4) (Monoamine oxidase type A) (MAO-A)	Maoa	AOFA_RAT
P21707	Synaptotagmin-1 (Synaptotagmin I) (Sytl) (p65)	Sytl	SYTI_RAT
P24268	Cathepsin D (EC 3.4.23.5) [Cleaved into: Cathepsin D 12 kDa light chain; Cathepsin D 9 kDa light chain ; Cathepsin D 34 kDa heavy chain ; Cathepsin D 30 kDa heavy chain]	Ctsd	CATD_RAT
P24473	Glutathione S-transferase kappa 1 (EC 2.5.1.18) (GST 13-13) (GST class-kappa) (GSTKl-1) (rGSTKl) (Glutathione S-transferase subunit 13)	Gstkl	GSTKI_RAT
P25809	Creatine kinase U-type , mitochondrial (EC 2.7.3.2) (Acidic-type mitochondrial creatine kinase) (Mia-CK) (Ubiquitous mitochondrial creatine kinase) (U-MtCK)	Ckmtl Ckmt	KCRU_RAT
P27653	C-1-tetrahydrofolate synthase, cytoplasmic (Cl-THF synthase) [Cleaved into: C-1-tetrahydrofolate synthase , cytoplasmic, N-terminally processed] [Includes : Methylenetetrahydrofolate dehydrogenase (EC 1. 5.1.5); Methenyltetrahydrofolate cyclohydrolase (EC 3.5.4.9); Formyltetrahydrofolate synthetase (EC 6.3.4.3)]	Mthfdl Mthfd	CITC_RAT
P27682	Neuroendocrine protein 7B2 (Secretogranin V) (Secretogranin-5) (Secretory granule endocrine protein I) [Cleaved into : N-terminal peptide; (-terminal peptide]	ScgS Sgnel	7B2_RAT
P27867	Sorbitol dehydrogenase (EC 1.1.1.14) (L-iditol 2-dehydrogenase)	Sord Sdhl	DHSO_RAT
P28064	Proteasome subunit beta type-8 (EC 3.4.25.1) (Macropain subunit C13) (Multicatalytic endopeptidase complex subunit C13) (Proteasome component C13) (Proteasome subunit beta-Si)	Psmb8	PSB8_RAT
P29314	40S ribosomal protein S9	Rps9	RS9_RAT
P31016	Disks large homolog 4 (Postsynaptic density protein 95) (PSD-95) (Synapse-associated protein 90) (SAP-90) (SAP90)	Dlg4 Dlgh4 Psd95	DLG4_RAT
P31721	Complement Clq subcomponent subunit B	Clqb	ClQB_RAT
P35171	Cytochrome c oxidase subunit 7A2, mitochondrial (Cytochrome c oxidase subunit VIIa-iiver/heart) (Cytochrome c oxidase subunit VIIa-L)	Co x7a2 Cox7a3 Cox7al	CX7A2_RAT
P35427	60S ribosomal protein L13a	Rpl13a	RL13A_RAT
P35435	ATP synthase subunit gamma, mitochondrial (F-ATPase gamma subunit)	AtpScl AtpSc	ATPG_RAT
P35565	Calnexin	Can x	CALX_RAT
P40307	Proteasome subunit beta type-2 (EC 3.4.25.1) (Macropain subunit C7-I) (Multicatalytic endopeptidase complex subunit C7-I) (Proteasome component C7-I)	Psmb2	PSB2_RAT
P41123	60S ribosomal protein L13	Rpl13	RL13_RAT
P41565	Isocitrate dehydrogenase **[NAD]** subunit gamma 1, mitochondrial (EC 1.1.1.41) (Isocitri c dehydrogenase subunit gamma) (NAD(+)-specific ICDH subunit gamma)	Idh3g	IDHGI_RAT
P43244	Matrin-3 (Nuclear scaffold protein p130/MAT3)	Matr3	MATR3_RAT
P47875	Cysteine and glycine-rich protein 1 (Cysteine-rich protein 1) (CRP) (CRP1)	Csrpl Csrp	CSRPI_RAT
P48500	Triosephosphate isomerase (TIM) (EC 5.3.1.1) (Triose-phosphate isomerase)	Tpil	TPIS_RAT
P48508	Glutamate--cysteine ligase regulatory subunit (GCS light chain) (Gamma-ECS regulatory subunit) (Gamma-glutamylcysteine synthetase regulatory subunit) (Glutamate--cysteine ligase modifier subunit)	Gclm Glclr	GSH0_RAT
P50503	Hsc70-interacting protein (Hip) (Protein FAM10A1) (Protein ST13 homolog)	St13 Fam10a1 Hip	F10A1_RAT
P50904	Ras GTPase-activating protein 1 (GAP) (GTPase-activating protein) (RasGAP) (Ras p21 protein activator) (p120GAP)	Rasal Rasa	RASAI_RAT
P52873	Pyruvate carboxylase, mitochondrial (EC 6.4.1.1) (Pyruvic carboxylase) (PCB)	Pc	PYC_RAT
P54690	Branched-chain-amino-acid aminotransferase , cytosolic (BCAT(c)) (EC 2.6.1.42)	Bcatl	BCATI_RAT
P54921	Alpha-soluble NSF attachment protein (SNAP-alpha) (N-ethylmaleimide-sensitive factor attachment protein alpha)	Napa Snap Snapa	**SNAA_RAT**

P55051	Fatty acid-binding protein, brain (Brain lipid-binding protein) (BLBP) (Brain-type fatty acid-binding protein) (B-FABP) (Fatty acid-binding protein 7)	Fabp7 Blbp	FABP7_RAT
P55067	Neurocan core protein (245 kDa early postnatal core glycoprotein) (Chondroitin sulfate proteoglycan 3) [Cleaved into : 150 kDa adult core glycoprotein]	Ncan Cspg3	NCAN_RAT
P56603	Secretory carrier-associated membrane protein 1 (Secretory carrier membrane protein 1) (SCAMP 37)	Scampl Scamp	SCAM1_RAT
P59215	Guanine nucleotide-binding protein G(o) subunit alpha	Gnaol Gna0 Gnao	GNAO_RAT
P60203	Myelin proteolipid protein (PLP) (Lipophilin)	Plpl Pip	MYPR_RAT
P60901	Proteasome subunit alpha type-6 (EC 3.4.25.1) (Macropain iota chain) (Multicatalytic endopeptida se complex iota chain) (Proteasome iota chain)	Psma6	PSA6_RAT
P61314	605 ribosomal protein L15	Rpl15	RL15_RAT
P61805	Dolichyl-diphosphooligosaccharide--protein glycosyltransferase subunit DAD1 (Oligosaccharyl transferase subunit DAD1) (EC 2.4.99.18) (Defender against cell death 1) (DAD-1)	Dadl	DAD1_RAT
P62076	Mitochondrial import inner membrane translocase subunit Tim13	Timm13 Tim13a Timm13a	TIM13_RAT
P62168	Neuronal calcium sensor 1 (NCS-1) (Frequenin homolog) (Frequenin-like protein) (Frequenin-like ubiquitous protein)	Ncsl Flup Freq	NCS1_RAT
P62243	405 ribosomal protein 58	Rps8	RS8_RAT
P62275	405 ribosomal protein 529	Rps29	RS29_RAT
P62630	Elongation factor 1-alpha 1 (EF-1-alpha-1) (Elongation factor Tu) (EF-Tu) (Eukaryotic elongation factor 1 A-1) (eEFIA-1)	Eef1a1 Eefla	EF1A1_RAT
P62752	605 ribosomal protein L23a	Rpl23a	RL23A_RAT
P62775	Myotrophin (Granule cell differentiation protein) (Protein V-1)	Mtpn Gcdp	MTPN_RAT
P62824	Ras-related protein Rab -3C	Rab3c	RAB3C_RAT
P62856	405 ribosomal protein 526	Rps26	RS26_RAT
P62870	Transcription elongation factor B polypeptide 2 (Elongin 18 kDa subunit) (Elongin-B) (EloB) (RNA polymerase II transcription factor SIii subunit B) (SIii p18)	Tceb2	ELOB_RAT
P62907	605 ribosomal protein L10a	Rpl10a	RL10A_RAT
P62909	405 ribosomal protein 53	Rps3	RS3_RAT
P62912	605 ribosomal protein L32	Rpl32	RL32_RAT
P62919	605 ribosomal protein L8	Rpl8	RL8_RAT
P63004	Platelet-a ctivating factor acet ylhydrolase IB subunit alpha (Lissencephaly-1 protein) (LIS-1) (PAF acetylhydrolase 45 kDa subunit) (PAF-AH 45 kDa subunit) (PAF-AH alpha) (PAFAH alpha)	Pafah1b1 Lis-1 Lisl Pafaha	LIS1_RAT
P67874	Casein kinase II subunit beta (CK II beta) (Phosvitin)	Csnk2b Ck2n	CSK2B_RAT
P81155	Voltage-dependent anion-selective channel protein 2 (VDAC-2) (B36-VDAC) (Outer mitochondrial membrane protein porin 2)	Vdac2	VDAC2_RAT
P85834	Elongation factor Tu, mitochondrial	Tufm	EFTU_RAT
P85845	Fascin	Fscnl	FSCN1_RAT
P85969	Beta-soluble NSF attachment protein (SNAP-beta) (N-ethylmaleimide-sensitive factor attachment protein beta)	Napb Snapb	SNAB_RAT
P97700	Mitochondrial 2-oxoglutarate/malate carrier protein (OGCP) (Solute carrier family 25 member 11)	Slc25a11 Slc20a4	M2OM_RAT
P97849	Long- chain fatty acid transport protein 1 (FATP-1) (Fatty acid transport protein 1) (EC 6.2.1.-) (Solute carrier family 27 member 1)	Slc27a1 Fatp Fatpl	S27A1_RAT
P97852	Peroxisomal multifunctional enzyme type 2 (MFE-2) (17-beta-hydroxysteroid dehydrogenase 4) (17-beta-HSD 4) (D-bifunctional protein) (DBP) (Multifunctional protein 2) (MPF-2) [Cleaved into: (3R)-hydroxyacyl-CoA dehydrogenase (EC 1.1.1.n1 2); Enoyl-CoA hydratase 2 (EC 4.2.1.107) (EC 4.2.1.119) (3-alpha ,7-alpha,1 2-alpha-trihydroxy-5-beta-cholest-24-enoyl-CoA hydratase)]	Hsd17b4 Edh17b4	DHB4_RAT
P97874	Cyclin-G-associated kinase (EC 2.7.11.1)	Gak	GAK_RAT
Q1HCL7	NAD kinase 2, mitochondrial (EC 2.7.1.23) (Mitochondrial NAD kinase) (NAD kinase domain-containing protein 1, mitochondrial)	Nadk2 Mnadk Nadkdl	NAKD2_RAT
Q2A121	Alpha-ketoglutarate-dependent dioxygenase FTO (EC 1.14.11.-) (Fat mass and obesity-associated protein)	Fto	FTO_RAT
Q2PQA9	Kinesin-1 heavy chain (Conventional kinesin heavy chain) (Ubiquitous kinesin heavy chain) (UKHC)	Kif5b Khc	KINH_RAT
Q3MIE4	Synaptic vesicle membrane protein VAT-1 homolog (EC 1. -.-.-) (Mitofusin-binding protein) (Protein MIB)	Vatl	VAT1_RAT

Q496Z0	Elongator complex protein 2 (ELP2) (SHINC-2) (STAT3-interacting protein 1) (StIPl)	Elp2 Statipl	ELP2_RAT
Q499N5	Acyl-CoA synthetase family member 2, mitochondrial (EC 6.2.1.-)	Acsf2	ACSF2_RAT
Q4FZT9	26S proteasome non-ATPase regulatory subunit 2	Psmd2	PSMD2_RAT
Q4G075	Leukocyte elastase inhibitor A (Serine protease inhibitor EIA) (Serpin Bla)	Serpinbla	ILEUA_RAT
Q4V7D2	Protein rogdi homolog	Rogdi	ROGDI_RAT
Q562C6	Leucine zipper transcription factor-like protein 1	Lztfll	LZTLI_RAT
Q566C7	Diphosphoinositol polyphosphate phosphohydrolase 1 (DIPP-1) (EC 3.6.1.52) (Diadenosine 5',5'"-P1,P6-hexaphosphate hydrolase 1) (EC 3.6.1.-) (Nucleoside diphosphate-linked moiety X motif 3) (Nudix motif 3)	Nudt3 Dipp Dippl	NUDT3_RAT
Q5BJT7	Coiled-coil domain-containing protein 93	Ccdc93	CCD93_RAT
Q5FVJ0	Protein RUFY3 (Rap2-interacting protein x) (RIPx) (Single axon-regulated protein) (Singar)	Rufy3 Ripx	RUFY3_RAT
Q5FVQ9	Tubulin-specific chaperone E (Tubulin-folding cofactor E)	Tbce	TBCE_RAT
Q5FWT1	Protein FAM98A	Fam98a	FA98A_RAT
Q510P2	Glycine cleavage system H protein, mitochondrial (Lipoic acid-containing protein)	Gcsh	GCSH_RAT
Q5PPL3	Sterol-4-alpha-carboxylate 3-dehydrogenase , decarboxylating (EC 1.1.1.170)	Nsdhl	NSDHL_RAT
Q5PPN5	Tubulin polymerization-promoting protein family member 3	Tppp3	TPPP3_RAT
Q5PQN0	Neurocalcin-delta	Ncald	NCALD_RAT
Q5SGEO	Leucine-rich PPR motif-containing protein, mitochondrial (130 kDa leucine-rich protein) (LRP 130) (Leucine rich protein 157) (rLRP157)	Lrpprc Lrp157	LPPRC_RAT
Q5U204	Ragulator complex protein LAMTOR3 (Late endosomal/lysosomal adaptor and MAPK and MTOR activator 3) (Mitogen-activated protein kinase kinase 1-interacting protein 1)	Lamtor3 Map2klip1	LTOR3_RAT
Q5U2N3	Membrane-associated phosphatidylinositol transfer protein 1 (Phosphatidylinositol transfer protein, membrane-associated 1) (PITPnm 1) (Pyk2 N-terminal domain-interacting receptor 2) (NIR-2)	Pitpnml Nir2 Pitpnm	PITMI_RAT
Q5XHZ0	Heat shock protein 75 kDa, mitochondrial (HSP 75) (TNFR-associated protein 1) (Tumor necrosis factor type 1 receptor-associated protein) (TRAP-1)	Trapl Hsp75	TRAPl_RAT
Q5Xl73	Rho GDP-dissociation inhibitor 1 (Rho GDI 1) (Rho-GDI alpha)	Arhgdia	GDIRl_RAT
Q5XIF3	NADH dehydrogenase [ubiquinone] iron-sulfur protein 4, mitochondrial (Complex 1-18 kDa) (Cl-18 kDa) (NADH-ubiquinone oxidoreductase 18 kDa subunit)	Ndufs4	NDUS4_RAT
Q5XIF6	Tubulin alpha-4A chain (Alpha-tubulin 4) (Tubulin alpha-4 chain)	Tuba4a Tuba4	TBA4A_RAT
Q5XIM5	Protein CDV3 homolog	Cdv3	CDV3_RAT
Q5XIN6	LETMI and EF-hand domain-containing protein 1, mitochondrial (Leucine zipper-EF-hand-containing transmembrane protein 1)	Letml	LETMI_RAT
Q5XIT1	Microtubule-associated protein RP/EB family member 3 (EBI protein family member 3) (EBF3) (End-binding protein 3) (EB3) (RP3)	Mapre3	MARE3_RAT
Q60587	Trifunctional enzyme subunit beta, mitochondrial (TP-beta) [Includes : 3-ketoacyl-CoA thiolase (EC 2.3.1.16) (Acetyl-CoA acyltransferase) (Beta-ketothiolase)]	Hadhb	ECHB_RAT
Q62651	Delta(3 ,5)-Delta(2,4)-dienoyl-CoA isomerase, mitochondrial (EC 5.3.3.-)	Echl	ECHl_RAT
Q62688	Inactive phospholipase C-like protein 1 (PLC-Ll) (PRIPl) (Phospholipase C-related but catalytically inactive protein) (p130)	Pleil	PLCLl_RAT
Q62703	Reticulocalbin-2 (Calcium-binding protein ERC-55) (Taipoxin-associated calcium-binding protein 49) (TCBP-49)	Rcn2 Erc55	RCN2_RAT
Q62785	28 kDa heat- and acid-stable phosphoprotein (PDGF-associated protein) (PAP) (PDGFA-associated protein 1) (PAPl)	Pdapl Haspp28	HAP28_RAT
Q62812	Myosin-9 (Cellular myosin heavy chain, type A) (Myosin heavy chain 9) (Myosin heavy chain, non-muscle IIa) (Non-muscle myosin heavy chain A) (NMMHC-A) (Non-muscle myosin heavy chain IIa) (NMMHC II-a) (NMMHC-IIA)	Myh9	MYH9_RAT

Q62868	Rho-associated protein kinase 2 (EC 2.7.11.1) (Rho-associated, coiled-coil-containing protein kinase 2) (Rho-associated, coiled-coil-containing protein kinase II) (ROCK-II) (RhoA-binding kinase 2) (pl50 ROK-alpha) (ROKalpha) (pl64 ROCK-2)	Rock2	ROCK2_RAT
Q62915	Peripheral plasma membrane protein CASK (EC 2.7.11.1) (Calcium/calmodulin-dependent se rine protein kinase)	Cask	CSKP_RAT
Q62951	Dihydropyrimidinase-related protein 4 (DRP-4) (Collapsin response mediator protein 3) (CRMP-3) (UNC33-like phosphoprotein 4) (ULIP-4) (Fragment)	Dpysl4 Crmp3 Ulip4	DPYL4_RAT
Q62967	Diphosphomevalonate decarboxylase (EC 4.1.1.33) (Mevalonate (diphospho)decarboxylase) (MDDase) (Mevalonate pyrophosphate decarboxylase)	Mvd Mpd	MVDl_RAT
Q62991	Seel family domain-containing protein 1 (SLYl homolog) (Slylp) (Syntaxin-binding protein 1-like 2) (Vesicle transport-related protein Ra410)	Scfdl Ra410 Slyl Stxbpll2	SCFDl_RAT
Q63009	Protein arginine N-methyltransferase 1 (EC 2.1.1.-) (Histone-arginine N-methyltransferase PRMTl) (EC 2.1.1.125)	Prmtl Hrmtll2	ANMl_RAT
Q63259	Receptor-type tyrosine-protein phosphatase-like N (R-PTP-N) (105 kDa islet cell antigen) (BEM-3) (Brain-enriched membrane-associated protein tyrosine phosphatase) (ICA105) (PTP **IA-2)** (PTPLP)	Ptprn	PTPRN_RAT
Q63347	26S protease regulatory subunit 7 (26S proteasome **AAA-**ATPase subunit RPTl) (Proteasome 26S subunit ATPase 2) (Protein MSSl)	Psmc2 Mssl	PRS7_RAT
Q63377	Sodium/potassium-transporting ATPase subunit beta-3 (Sodium/potassium-dependent ATPase subunit beta-3) (ATPB-3) (CD antigen CD298)	Atplb3	AT1B3_RAT
Q63424	Solute carrier family 15 member 2 (Kidney H(+)/peptide cotransporter) (Oligopeptide tr ansporter , kidney isoform) (Peptide transporter 2)	Slc15a2 Pept2	S15A2_RAT
Q63537	Synapsin-2 (Synapsin II)	Syn2	SYN2_RAT
Q63556	Serine protease inhibitor A3M (Serpin A3M) (Serine protease inhibitor 2.4) (SPl-2.4) (Fragment)	Serpina3m	SPA3M_RAT
Q63569	26S protease regulatory subunit 6A (26S proteasome AAA-ATPase subunit RPT5) (Proteasome 26S subunit ATPase 3) (Spermatogenic cell/sperm-associated Tat-binding protein homolog SATA) (Tat-binding protein 1) (TBP-1)	Psmc3 Tbpl	PRS6A_RAT
Q63797	Proteasome activator complex subunit 1 (11S regulator complex subunit alpha) (REG-alpha) (Activator of multicatalytic protease subunit 1) (Proteasome activator 28 subunit alpha) (PA28a) (PA28alpha)	Psmel	PSMEl_RAT
Q641Y2	NADH dehydrogenase [ubiquinone] iron-sulfur protein 2, mitochondrial (EC 1. 6.5.3) (EC 1.6.99.3) (Complex l-49kD) (Cl-49kD) (NADH-ubiquinone oxidoreductase 49 kDa subunit)	Ndufs2	NDUS2_RAT
Q641Y8	ATP-dependent RNA helicase DDXl (EC 3.6.4.13) (DEAD box protein 1)	Ddxl	DDXl_RAT
Q64303	Serine/threonine-protein kinase PAK 2 (EC 2.7.11.1) (Gamma-PAK) (p21-activated kinase 2) (PAK-2) [Cleaved into: PAK-2p27; PAK-2p34]	Pak2	PAK2_RAT
Q64591	2,4-dienoyl-CoA reductase, mitochondrial (EC 1.3.1.34) (2,4-dienoyl-CoA reductase [NADPH]) (4-enoyl-CoA reductase [NADPH])	Decrl Deer	DECR_RAT
Q64620	Serine/threonine-protein phosphatase 6 catalytic subunit (PP6C) (EC 3.1.3.16) (Protein phosphatase V) (PP-V) [Cleaved into: Serine/threonine-protein phosphatase 6 catalytic subunit, N-terminally processed]	Ppp6c Ppv	PPP6_RAT
Q66HA8	Heat shock protein 105 kDa (Heat shock 110 kDa protein)	Hsphl Hspl05 Hspll0	HS105_RAT
Q66HF1	NADH-ubiquinone oxidoreductase 75 kDa subunit, mitochondrial (EC 1. 6.5.3) (EC 1. 6.99.3)	Ndufsl	NDUSl_RAT
Q68FR2	Bridging integrator 2	Bin2	BIN2_RAT
Q68FX0	lsocitrate dehydrogenase [NAO] subunit beta, mitochondrial (EC 1.1.1.41) (lsocitric dehydrogenase subunit beta) (NAD(+)-specific ICDH subunit beta)	ldh3B	IDH3B_RAT
Q6AXQ0	SUMO-activating enzyme subunit 1 (Ubiquitin-like 1-activating enzyme ElA) [Cleaved into: SUMO-activating enzyme subunit 1, N-terminally processed]	Sael Ublela	SAEl_RAT
Q6AXX6	Redox-regulatory protein FAM213A (Peroxiredoxin-like 2 activated in M-CSF stimulated monocytes) (Protein PAMM) (Sperm head protein 1)	Fam213a Pamm Shpl	F213A_RAT
Q6AXY0	Glutathione S-transferase A6 (EC 2.5.1.18) (GST class-alpha member6)	Gsta6	GSTA6_RAT

Q6AY63	ADP-sugar pyrophosphatase (EC 3.6.1.13) (8-oxo-dGDP phosphatase) (EC 3.6.1.58) (Nucleoside diphosphate-linked moiety X motif 5) (Nudix motif 5)	NudtS	NUDTS_RAT
Q6AYT3	tRNA-splicing ligase RtcB homolog (EC 6.5.1.3) (pSS)	Rtcb	RTCB_RAT
Q6DGG0	Peptidyl-prolyl cis-trans isomerase D (PPlase D) (EC 5.2.1.8) (40 kDa peptidyl-prolyl cis-trans isomerase) (Cyclophilin-40) (CYP-40) (Rotamase D)	Ppid	PPID_RAT
Q6MG60	N(G),N(G)-dimethylarginine dimethylaminohydrolase 2 (DDAH-2) (Dimethylarginine dimethylaminohydrolase 2) (EC 3.5.3.18) (DDAHII) (Dimethylargininase-2)	Ddah2	DDAH2_RAT
Q6P502	T-complex protein 1 subunit gamma (TCP-1-gamma) (CCT-gamma)	Cct3	TCPG_RAT
Q6P6R2	Dihydrolipoyl dehydrogenase, mitochondrial (EC 1.8.1.4) (Dihydrolipoamide dehydrogenase)	Did	DLDH_RAT
Q6PCU2	V-type proton ATPase subunit E 1 (V-ATPase subunit E 1) (Vacuolar proton pump subunit E 1)	Atp6v1e1	VATEI_RAT
Q6Q0N1	Cytosolic non-specific dipeptidase (EC 3.4.13.18) (CNDP dipeptidase 2)	Cndp2	CNDP2_RAT
Q6VBQ5	Myeloid-associated differentiation marker (Myeloid up-regulated protein)	Myadm	MYADM_RAT
Q71UF4	Histone-binding protein RBBP7 (Nucleosome-remodeling factor subunit RBAP46) (Retinoblastoma-binding protein 7) (RBBP-7)	Rbbp7	RBBP7_RAT
Q7TP17	Splicing factor U2AF 26 kDa subunit (Liver regeneration-related protein LRRG157/LRRG158) (U2 auxiliary factor 26) (U2 small nuclear RNA auxiliary factor 1-like protein 4)	U2af1l4 Cb2-806 Cb2-807	U2AF4_RAT
Q7TQ16	Cytochrome b-cl complex subunit 8 (Complex III subunit 8) (Complex III subunit VIII) (Low molecular mass ubiquinone-binding protein) (Ubiquinol-cytochrome c reductase complex 9.5 kDa protein) (Ubiquinol-cytochrome c reductase complex ubiquinone-binding protein QP-C)	Uqcrq Qpc	QCR8_RAT
Q80W98	Small glutamine-rich tetratricopeptide repeat-containing protein beta (Beta-SGT) (Small glutamine-rich protein with tetratricopeptide repeats 2)	Sgtb Sgt2	SGTB_RAT
Q80Z29	Nicotinamide phosphoribosyltransferase (NAmPRTase) (Nampt) (EC 2.4.2.12) (Pre-B-cell colony-enhancing factor 1 homolog) (PBEF) (Visfatin)	Nampt Pbefl	NAMPT_RAT
Q811X6	Lambda-crystallin homolog (EC 1.1.1.45) (L-gulonate 3-dehydrogenase) (Gul3DH)	Cryll Cry	CRYLI_RAT
Q8CFD0	Sideroflexin-5 (Tricarboxylate carrier BBG-TCC)	SfxnS	SFXNS_RAT
Q8CFN2	Cell division control protein 42 homolog	Cdc42	CDC42_RAT
Q8K1Q0	Glycylpeptide N-tetradecanoyltransferase 1 (EC 2.3.1.97) (Myristoyl-CoA:protein N-myristoyltransferase 1) (NMT 1) (Type I N-myristoyltransferase) (Peptide N-myristoyltransferase 1)	Nmtl	NMTI_RAT
Q8R431	Monoglyceride lipase (MGL) (EC 3.1.1.23) (Monoacylglycerol lipase) (MAGL)	Mgll Mgl2	MGLL_RAT
Q8VD52	Pyridoxal phosphate phosphatase (PLP phosphatase) (EC 3.1.3.3) (EC 3.1.3.74) (Chronophin) (Reg I-binding protein 1)	Pdxp Cin Pip Plpp Rbpl	PLPP_RAT
Q8VIJS	Bifunctional protein NCOAT (Meningioma-expressed antigen 5) (Nuclear cytoplasmic O-GlcNAcase and acetyltransferase) [Includes: Beta-hexosaminidase (EC 3.2.1.52) (Beta-N-acetylhexosaminidase) (Hexosaminidase C) (N-acetyl-beta-D-glucosaminidase) (N-acetyl-beta-glucosaminidase) (O-GlcNAcase); Histone acetyltransferase (HAT) (EC 2.3.1.48)]	Mgeas Hexc Meas	NCOAT_RAT
Q91XU8	Phosphatidate cytidylyltransferase 2 (EC 2.7.7.41) (CDP-DAG synthase 2) (CDP-DG synthase 2) (CDP-diacylglycerol synthase 2) (CDS 2) (CDP-diglyceride pyrophosphorylase 2) (CDP-diglyceride synthase 2) (CTP:phosphatidate cytidylyltransferase 2)	Cds2	CDS2_RAT
Q91Y81	Septin-2 (Vascular endothelial cell specific protein 11)	Sept2 Vesp11	SEPT2_RAT
Q920L2	Succinate dehydrogenase [ubiquinone] flavoprotein subunit, mitochondrial (EC 1.3.5.1) (Flavoprotein subunit of complex II) (Fp)	Sdha	SDHA_RAT
Q99ND9	RWD domain-containing protein 1 (Small androgen receptor-interacting protein)	Rwddl Sarip	RWDDI_RAT
Q9Jl66-3	Electrogenic sodium bicarbonate cotransporter 1 (Sodium bicarbonate cotransporter) (NBC-like protein) (Na(+)/HCO3(-) cotransporter) (Solute carrier family 4 member4)	Slc4a4 Nbc Nbcl Nbcel Rnbcl	S4A4_RAT

Q9JLT0	Myosin-10 (Cellular myosin heavy chain , type B) (Myosin heavy chain 10) (Myosin heavy chain , non-muscle **lib)** (Non-muscle myosin heavy chain B) (NMMHC-B) (Non-muscle myosin heavy chain lib) (NMMHC 11-b) (NMMHC-11B)	Myhl0	MYHl0_RAT
Q9QUL6	Vesicle-fusing ATPase (EC 3.6.4.6) (N-ethylmaleimide-sensitive fusion protein) (NEM-sensitive fusion protein) (Vesicular-fusion protein NSF)	Nsf Ergl	NSF_RAT
Q9QX69	LanC-like protein 1 (40 kDa erythrocyte membrane protein) (p40)	Lanell Gpr69a	LANCl_RAT
Q9QYF3	Unconventional myosin-Va (Dilute myosin heavy chain, non-muscle)	MyoSa	MYOSA_RAT
Q9QYW3	MOB-like protein phocein (Class II mMOBl) (Mobl homolog 3) (Mob3) (Mps one binder kinase activator-like 3) (Phocein) (Preimplantation protein 3)	1ob4 Mob3 Mobkll Phocn Prei	PHOCN_RAT
Q9R0J8	Legumain (EC 3.4.22.34) (Asparaginyl endopeptidase) (Prot ease, cysteine 1)	Lgmn Prscl	LGMN_RAT
Q9WVA1	Mitochondrial import inner membrane translocase subunit Tim8 A (Deafness dystonia protein 1 homolog)	Timm8a Ddpl Tim8a	TIM8A_RAT
Q9Z0WS	Protein kinase C and casein kinase substrate in neurons protein 1 (Dynamin proline-rich domain-interacting protein) (Dynamin PRO-interacting protein) (Synaptic , dynamin-associated protein I) (Syndapin-1) (Syndapin-1) (Sdpl)	Pacsinl	PACNl_RAT
Q9Z1AS	NEDD8-activating enzyme El regulatory subunit (Amyloid beta precursor protein-binding protein 1, 59 kDa) (APP-BPI) (Amyloid protein-binding protein 1)	Nael Appbpl	ULAl_RAT
Q9Z1N4	3'(2'),5'-bisphosphate nucleotidase 1 (EC 3.1.3.7) (Bisphosphate 3'-nucleotidase 1) (PAP-inositol 1,4-phosphatase) (PIP) (scHAL2 analogous 3)	Bpntl Sal3	BPNTl_RAT
Q9Z270	Vesicle-associated membrane protein-associated protein A (VAMP-A) (VAMP-associated protein A) (VAP-A) (33 kDa VAMP-associated protein) (VAP-33)	Vapa Vap33	VAPA_RAT

Supplementary Table S4A: GO Analysis of the proteins altered by GRP78 overexpression (Terms from the Process Ontology of gene_association.rgd with p-value <= 0.05; PROCESS)

Gene Ontology term	Cluster frequency	Genome frequency	Corrected P-value	FDR	False Positives	Genes annotated to the term
oxidation-reduction process	33 of 220 genes, 15.0%	1899 of 36892 genes, 5.1%	3.52e-05	0.00%	0.00	LDHA _RAT, CRYLI_RAT, VATI_RAT, QCR8_RAT, F213A_RAT, NDUS4_RAT, DH E3_RAT, NDUSI _RAT, ACADL_RAT, DLDH_RAT, EC HM _RAT, COX2_RAT, AOFA_RAT, GLYG_RAT, FTO_RAT, IVD _RAT, DECR_RAT, SODM_RAT, TPIS_RAT, DHB4_RAT, NSDHL_RAT, NDUS2 _RAT, MGSTI _RAT , GSTKI_RAT, HCD2_RAT, ECHB_RAT, ECHI_RAT, TRAPI_RAT, DHSO_RAT, NDUV2 _RAT, SDHA_RAT, UCRI_RAT, CITC_RAT
small molecule metabolic process	57 of 220 genes, 25.9%	4674 of 36892 genes, 12.7 %	7.79e-05	0.00%	0.00	LTOR3_RAT, LDHA _RAT, CRYLI_RAT, BCATI_RAT, EFTU_RAT, DHE3_RAT, MGLL_RAT, RASAI _RAT, NUDT3_RAT, GSHO_RAT, NDUSI _RAT, ACADL_RAT, IDHGI _RAT, CN37_RAT, NAMPT_RAT, TPMI_RAT, ECHM _RAT, MVDI_RAT, RPGF2_RAT, GSTM4_RAT, GNAO_RAT, NUDTS_RAT, KI NH _RAT, IVD_RAT, MYH9_RAT, ATPA_RAT, DECR_RAT, KPYM _RAT, MYPR_RAT, SODM_RAT, DDXI_RAT, TPIS_RAT, DHB4_RAT, NSDHL_RAT, S27AI_RAT, MGSTI_RAT, SEPT2_RAT, CDC42_RAT, GSTKI _RAT, DPYL4_RAT, TBA4A_RAT, ECHB _RAT, ECHI_RAT, EFIAI_RAT, NSF_RAT, AT1A2_RAT, GSTA6_RAT, GDIRI _RAT, NDUV2_RAT, ATPG_RAT, SDHA_RAT, DDAH2_RAT, ACSF2_RAT, LISI _RAT, NAKD 2_RAT, ATP8_RAT, CITC_RAT
carboxylic acid metabolic process	27of220 genes, 12.3%	1452 of 36892 genes, 3.9%	0.00018	0.00%	0.00	MYPR_RAT, SODM_RAT, TPIS_RAT, LDH A_RAT, CRYLI_RAT, DHB4_RAT, BCATI_RAT, S27AI_RAT, MGSTI_RAT, GSTKI_RAT, DHE3_RAT, MGLL_RAT, ECHB_RAT, GSHO_RAT, ECHI _RAT, ACADL_RAT, IDHGI _RAT, ECHM_RAT, GSTM4_RAT, GSTA6_RAT, SDHA_RAT, DDAH2_RAT, ACSF2_RAT, IVD_RAT, DECR_RAT, KPYM _RAT, CITC_RAT
organism metabolic process	88of220 genes, 40.0%	9029 of 36892 genes, 24.5%	0.00024	0.00%	0.00	RPNI _RAT, LDHA _RAT, CDS2_RAT, BCATI_RAT, NDU54_RAT, MGLL_RAT, NUDT3 _RAT, GSHO_RAT, IDHGI _RAT, NAMPT _RAT, EC HM _RAT, COX2_RAT, MVDI_RAT, RPGF2_RAT, GSTM4_RAT, GLYG_RAT, PLPP_RAT, FTO_RAT, IVD_RAT, ATPA_RAT, KPYM_RAT, DDXI _RAT, TPIS_RAT, DLG4_RAT, BPNTI _RAT, DHB4_RAT, NDUS2_RAT, NSDHL_RAT, S27AI_RAT, SEPT2_RAT, TBA4A_RAT, HCD2_RAT, EFI AI _RAT, NSF_RAT, KPCA _RAT, RS3 _RAT, AT1A2_RAT, CIQB_RAT, GDIRI_RAT, GSTPI_RAT, NDUV2_RAT , ATPG_RAT, SDHA_RAT, DDAH2_RAT, ACSF2_RAT, ATP8_RAT, STS_RAT, LTOR3_RAT, CRYLI_RAT, CYTC_RAT, VATI_RAT, EFTU_RAT, NCOAT_RAT, QCR8_RAT, F213A_RAT, DHE3_RAT, RASAI _RAT, PLCLI_RAT, NDUSI _RAT, ACADL_RAT, ANMI_RAT, CN37_RAT, DLDH_RAT, TPMI _RAT, GNAO_RAT, AOFA_RAT, NUDT5 _RAT, KINH_RAT, PLCGI_RAT, MYH9_RAT, DECR_RAT, MYPR_RAT, SODM_RAT, DADI _RAT, MGSTI_RAT, CDC42_RAT, GSTKI_RAT, DPYL4_RAT, ECHB _RAT, ECHI _RAT, TRAPI_RAT, DHSO_RAT, GSTA6_RAT, LISI _RAT, NAKD2_RAT, UCRI_RAT, PYC_RAT, CITC_RAT
oxoacid metabolic process	27of220 genes, 12.3%	1535 of 36892 genes, 4.2%	0.00055	0.00%	0.00	MYPR_RAT, SODM_RAT, TPIS_RAT, LDHA _RAT, CRYLI_RAT, DHB4_RAT, BCATI_RAT, S27AI_RAT, MGSTI_RAT, GSTKI_RAT, DHE3_RAT, MGLL_RAT, ECHB _RAT, GSHO_RAT, ECHI _RAT, ACADL_RAT, IDHGI _RAT, ECHM_RAT, GSTM4_RAT, GSTA6_RAT, SDHA_RAT, DDAH2_RAT, ACSF2_RAT, IVD_RAT, DECR_RAT, KPYM _RAT, CITC_RAT
organic acid metabolic process	27of220 genes, 12.3%	1564 of 36892 genes, 4.2%	0.00079	0.00%	0.00	MYPR_RAT, SODM_RAT, TPIS_RAT, LDHA _RAT, CRYLI_RAT, DHB4_RAT, BCATI_RAT, S27AI_RAT, MGSTI _RAT, GSTKI_RAT, DHE3_RAT, MGLL_RAT, ECHB_RAT, GSHO_RAT, ECHI _RAT, ACADL_RAT, IDHGI _RAT, ECHM_RAT, GSTM4_RAT, GSTA6_RAT, SDHA_RAT, DDAH2_RAT, ACSF2_RAT, IVD_RAT, DECR_RAT, KPYM _RAT, CITC_RAT
hydrogen ion transmembrane transport	10of220 genes, 4.5%	227 of 36892 genes, 0.6%	0.00122	0.00%	0.00	COX41_RAT, COX2_RAT, ATPG_RAT, QCR8_RAT, UCRI_RAT, VATEI_RAT, ATPA_RAT, CX7A2_RAT, ATP8_RAT, MPCP_RAT
proton transport	10of220 genes, 4.5%	273 of 36892 genes, 0.7 %	0.00633	0.00%	0.00	COX41_RAT, COX2_RAT, ATPG_RAT, QCR8_RAT, UCRI_RAT, VATEI_RAT, ATPA_RAT, CX7A2_RAT, ATP8_RAT, MPCP_RAT
glutathione metabolic process	6of220 genes, 2.7%	76 of 36892 genes, 0.2%	0.00645	0.00%	0.00	SODM_RAT, MGSTI_RAT, GSTKI_RAT, GSTM4_RAT, GSHO_RAT, GSTA6_RAT
hydrogen transport	10of220 genes, 4.5%	275 of 36892 genes, 0.7%	0.00674	0.00%	0.00	COX41_RAT, COX2_RAT, ATPG_RAT, QCR8_RAT, UCRI_RAT, VATEI_RAT, ATPA_RAT, CX7A2_RAT, ATP8_RAT, MPCP_RAT

Supplementary Table S4B: GO Analysis of the proteins altered by GRP78 overexpression (Terms from the Process Ontology of gene_association .rgd with p-value <= 0.05; FUNCTION)

Gene Ontology term	Cluster frequency	Genome frequency	Corrected P-value	FDR	False Positives	Genes annotated to the term
oxidoreductase activity	24 of 220 genes, 10.9%	1489 of 36892 genes, 4.0%	0.00231	0.00%	0.00	COX41_RAT, SODM_RAT, CRYLI_RAT, DHB4_RAT, VATI_RAT, NDUS2_RAT, NSDHL_RAT, QCR8_RAT, MGSTI _RAT, GSTKI_RAT, DHE3 _RAT, HCD2_RAT, NDUSI _RAT, ACADL_RAT, IDHGI _RAT, COX2_RAT, DHSO_RAT, NDUV2 _RAT, SDHA_RAT, IVD_RAT, FTO_RAT, CX7A2_RAT, DECR_RAT, CITC_RAT
structural molecule activity	22of220 genes, 10.0%	1375 of 36892 genes, 3.7%	0.00614	1.00%	0.02	LTOR3_RAT, RS19_RAT, RS26_RAT, NFL_RAT, RL23A_RAT, CLCA_RAT, VAPA_RAT, RS29_RAT, RL18 _RAT, RL3 2_RAT, RLI S_RAT, SEPT2_RAT, TBA4A_RAT, RS3_RAT, RS8_RAT, RL9_RAT, CLCB_RAT, RLIOA_RAT, RL8_RAT, RS9_RAT, RL13_RAT, RLAO_RAT
hydrogen ion transmembrane transporter activity	8 of 220 genes, 3.6%	201 of 36892 genes, 0.5%	0.00648	0.67%	0.02	COX41_RAT, QCR8_RAT, VATEI_RAT, ATPA_RAT, COX2_RAT, CX7A2_RAT, ATP8_RAT, ATPG_RAT
3-hydroxyacyl-CoA dehydrogenase activity	3 of 220 genes, 1.4%	12 of 36892 genes, 0.0%	0.00950	1.00%	0.04	CRYLI_RAT, DHB4_RAT, HCD2_RAT

Supplementary Table 4C: GO Analysis of the proteins altered by GRP78 overexpression (Terms from the Process Ontology of gene_association .rgd with p-value <= **0.05;** COMPONENT)

Gene Ontology term	Cluster frequency	Genome frequency	Corrected P-value	FDR	False Positives	Genes annotated to the term
mitochondrial membrane	32 of 220 genes, 14.5%	916 of 36892 genes, 2.5%	2.03e-13	0.00%	0.00	COX41_RAT, KCRU _RAT, SODM_RAT, CDS2_RAT, VATI_RAT, NDUS2_RAT, M20M_RAT, QCR8_RAT, MGSTI_RAT, NDU54_RAT, GSTKI_RAT, DHE3 _RAT, ECHB _RAT, MPCP_RAT, NDUSI _RAT, ACAD L_RAT, LETMI _RAT, TRAPI_RAT, KPCA_RAT, COX2_RAT, DHSO_RAT, VDAC2_RAT, TIM13_RAT, NDUV2_RAT, ATPG_RAT, SDHA_RAT, PYC_RAT, IVD _RAT, ATPA_RAT, CX7A2_RAT, ATP8_RAT, SFXNS_RAT
mitochondrial envelope	32 of 220 genes, 14.5%	977 of 36892 genes, 2.6%	1.21e-12	0.00%	0.00	COX41_RAT, KCRU _RAT, SODM_RAT, CDS2_RAT, VATI_RAT, NDUS2_RA T, M20M_RAT, QCR8_RAT, MGSTI_RAT, NDU54_RAT, GSTKI_RAT, DHE3 _RAT, ECHB_RAT, MPCP_RAT, NDUSI _RAT, ACADL_RAT, LETMI _RAT, TRAPI _RAT, KPCA_RAT, COX2_RAT, DHSO_RAT, VDAC2_RAT, TIM13 _RAT, NDUV2_RAT, ATPG_RAT, SDHA_RAT, PYC_RAT, IVD _RAT, ATPA_RAT, CX7A2_RAT, ATP8_RAT, SFXNS_RAT
mitochondrial inner membrane	26 of 220 genes, 11.8%	632 of 36892 genes, 1.7%	2.86e-12	0.00%	0.00	COX41_RAT, KCRU _RAT, SODM_RAT, CDS2_RAT, NDUS2 _RAT, M20M_RAT, QCR8_RAT, MGSTI_RAT, NDU54_RAT, GSTKI_RAT, DHE3 _RAT, EC HB_RAT, MPCP_RAT, NDUSI _RAT, LETMI _RAT, TRAPI_RAT, COX2_RAT, TIM 13_RAT, NDUV2_RAT, ATPG_RAT, SDHA_RAT, PYC_RAT, ATPA_RAT, CX7A2_RAT, ATP8_RAT, SFXN5_RAT

mitochondria l	35 of 220 genes, 15.9%	1228of 36892 genes, 3.3%	3.55e-12	0.00%	0.00	COX41_RAT, KCRU _RAT, CDS2_RAT, VATI_RAT, M20M_RAT, EFTU_RA T, QCR8_RAT, NDU 54_RAT, DHE3 _RAT, MPCP_RAT, NDUSI _RAT, ACADL_RAT, DLDH_RAT, ECHM _RAT, COX2_RAT, TIM13_RAT, IVD_RAT, ATPA_ RAT, CX7A2 _RAT, SODM_RAT, NDUS2_RAT, MG STI _RAT, GSTKI_RAT, EC HB_RAT, KPCA_RAT, TRAPI_RAT, LETMI _RAT, DHSO_RAT, VDAC2_RAT, NDUV2_RAT, ATPG_RAT, SDHA_RAT, PYC_RAT, ATP8_RAT, SFXNS_RAT
organelle inner membrane	27of220 genes, 12.3%	699 of 36892 genes, 1.9%	3.91e-12	0.00%	0.00	COX41_RAT, KCRU_RAT, SODM_RAT, CDS2_RAT, NDU S2_RAT, M 20 M_RAT, QCR8_RAT, MGSTI_RAT, NDU54_RAT, GSTKI_RAT, DHE3 _RAT, ECHB_RAT, MPCP_RAT, NDUSI _RAT, LPPRC_RAT, LETMI _RAT, TRAPI_RAT, COX2_RAT, TIM 13_RAT, NDUV2_RAT, ATPG_RAT, SDHA_RAT, PYC_RAT, ATPA_RAT, CX7A2_RAT, ATP8_RAT, SFXN5_RAT
cytop lasm	146 of 220 genes, 66.4%	15447 of 36892 genes, 41.9%	3.72e-11	0.00%	0.00	COX41_RAT, KCRU _RAT, LANCI_RAT, BCATI_RAT, STIPI_RAT, VATEI_RAT, TCPG_RAT, 7B2_RAT, RL7 _RAT, IDHGI _RAT, EC HM _RAT, PRS7 _RAT, CLCB_RAT, PLPP_RAT, IVD_RAT, ATPA_RAT, NCSI _RAT, RS19_RAT, DDXI_RAT, DLG4_RAT, PTN2 3_RAT, AT1B3_RAT, NSDHL_RAT, RL32_ RAT, SEPT2_RAT, EL P2_RAT, TBA4A_RAT, SNAB_RAT, TPPP3_RAT, EFIAI_RAT, SCFDI_RAT, KPCA_RAT, SH3GI_RAT, VDAC2_RAT, GDIRI_RAT, NDUV2_RAT, HSI OS_RAT, PSB2_RAT, FSCNI _RAT, NUCL_RAT, RCN2_RAT, CNDP2_RAT, NFL_RAT, M20M_RAT, PPP6_RAT , QCR8_RAT, F213A_RA T, RASAI_RAT, CATD_RAT, PITMI _RAT, CN37_RAT, DLDH_RAT, TPMI_RAT, TIM13_RAT, KINH _RAT, RTCB _RAT, DECR_RAT, SODM_RAT, RL23A_RAT, RL18 _RAT, MGSTI_RAT, CDC42_RAT, GSTKI_RAT, ECHB _RAT, GSTA6_RAT, NAKD2_RAT, LISI _RAT, CALX_RAT, SFXN5_RAT, NMTI _RAT, LDHA _RAT, RPNI _RAT, CDS2_RAT, PSB8_ RAT, PAK2_RAT, CSK2B _RAT, NDU54_RAT, NUDT3_RAT, MPCP_RAT, SYN 2_RAT, COX2_RAT, GSTM4_RAT, RPGF2_RAT, RLI OA_RAT, CSKP_RAT, LGMN _RAT, MYOSA_RAT, SNAA_RAT, KPYM _RAT, SYTI_RAT, DHB4_RAT, ROCK2_RAT, VAPA_RAT, NDUS2_RAT , HCD2_RAT, LPPRC_RAT, TBCE_RAT, ILEUA _RAT, ULAI _RAT, GSTPI_RAT, ATPG_RAT, SDHA_RAT, ACSF2_RAT, DDAH2_RAT, PSA6_RAT, SCAMI_RAT, ATP8_RAT, GAK_RAT, RLAO_RAT, LTOR3_RAT, CRYLI_RAT, PPID_RAT, VATI _RAT, MA RE3 _RAT, EFTU_RAT, PACNI_RAT, PRS6A_RAT, DHE3_RAT, PLCLI_RAT, NDUSI _RA T, ACADL_RAT, ANMI _RAT, SAEI_RAT, RL9 _RAT, RL8 _RAT, M YH9_RAT, CX7A2_RAT, VGF_RAT, RL13_RAT, CDV3_RAT, PHOCN_RAT, RAB3C_RAT, CLCA_RA T, MTPN_RAT, DA□I _RAT, RLI S_RAT, LZTLI _RAT, DPYL4 _RAT, RL13A _RAT, EC HI _RAT , TRAPI _RAT, LETMI _RAT, DHSO_RAT, PYC_RAT, BIN2_RAT, CIT C_RAT
cytop lasm ic	117of220 genes, 53.2%	10942 of 36892 genes, 29.7%	5.08e-11	0.00%	0.00	COX41_RAT, KCRU _RAT, RPNI_RAT, CDS2_RAT, PAK2_RAT, BCATI_RAT, NDUS4_RAT, VATEI _RAT, SYN2_RAT, MP CP_RAT, 7B2_RAT, RL7 _RAT, IDHGI _RAT, ECHM _RAT, COX2_RAT, RPGF2_RAT, GSTM4_RAT, PRS7_RAT, CSKP_RAT, RLI OA_RAT, CLCB_RAT, LGMN _RAT, MYOSA_RAT, PLPP_RAT, IVD_RAT, ATPA_RAT, NCSI _RAT, SNAA_RAT, SYTI_RAT, RS19_RAT, DDXI _RAT, DLG4_RA T, PTN23_RAT, DHB4_RAT, AT1B3_ RAT, VAPA_RAT, NDUS2_RAT, NSDHL_RAT, RL32_ RAT, SEPT2_RAT, HCD2_RAT, SNAB_RAT, LPPRC_RAT, SCFDI_RAT, KPCA_RAT, SH3GI_RAT, VDAC2_RAT, ULAI _RAT, ATPG_RAT, GSTPI_RAT, NDUV2_RAT, SDHA_RAT, DDAH2_RAT, HS105 _RAT, ACSF2_RAT, PSA6_RAT, SCAMI_RAT, GAK_RAT, ATP8_RAT, FSCNI _RAT, RLAO_RAT, RCN 2_RAT, LTOR3 _RAT, CRYLI_RAT, NFL_RAT, VATI_RAT, MARE3_RAT, M20M_RAT, EFTU_RAT, PPP6_RAT, PACNI _RAT, QCR8_RAT, F213 A_RA T, PRS6A_RAT, DHE3 _RAT, CATD_RAT, NDUSI _RAT, PITMI _RAT, ACADL_RAT, SAEI_RAT, ANMI _RAT, CN37 _RAT, DLDH_RAT, RL9 _RAT, TIM13 _RAT, KINH _RAT, RL8 _RAT , MYH9 _RAT, VGF_RAT, CX7A2_RAT, DECR_RAT, RL13_RAT, SODM_RAT, PHOCN_RAT, RAB 3C_RAT, CLCA_RAT, RL23A_RAT, RL1 8_ RAT, MTPN_RAT, DADI _RAT, RLI S_RAT, MGSTI_RAT, CDC42_RAT, GSTKI _RAT, RL13A_RAT, ECHB_RAT, ECHI _RAT, LETMI _RAT, TRAPI _RAT, DHSO_RAT, NAKD2_RAT, LISI _RAT, CALX_RAT, PYC _RAT, BIN2_RAT, SFXN5_RAT, CITC_RAT
organe lle envelope	35 of 220 genes, 15.9%	1477 of 36892 genes, 4.0%	7.05e-10	0.00%	0.00	COX41_RAT, KCRU _RAT, CDS2_RAT, VATI_RAT, ROGDI_RAT, M20M_RAT, QCR8_RAT, NDU 54_RAT, DHE3 _RAT, MPCP_RAT, NDUSI _RAT, ACADL_RAT, COX2_RAT, TIM13_RAT, IVD_RAT, ATPA_ RAT, CX7A 2_RAT, SODM_RAT, NDUS2_RAT, MGSTI_RAT, GSTKI_RAT, EC HB_RAT, LPPRC_RAT, KPCA _RAT, TRAPI _RAT, LETMI _RAT, DHSO_RAT, VDAC2_RAT, ATPG_RAT, NDUV2_RAT, SDHA_RAT, LISI _RAT, PYC_RAT, ATP8_RAT, SFXNS_RAT
envelope	35 of 220 genes, 15.9%	1483 of 36892 genes, 4.0%	7.89e-10	0.00%	0.00	COX41_RAT, KCRU _RAT, CDS2_RAT, VATI_RAT, ROGDI_RAT, M20M_RAT, QCR8_RAT, NDU 54_RAT, DHE3 _RAT, MPCP_RAT, NDUSI _RAT, ACADL_RAT, COX2_RAT, TIM13_RAT, IVD_RAT, ATPA_RAT, CX7A 2_RAT, SODM_RAT, NDUS2_RAT, MGSTI_RAT, GSTKI_RAT, EC HB_RAT, LPPRC_RAT, KPCA _RAT, TRAPI _RAT, LETMI _RAT, DHSO_RAT, VDAC2_RAT, ATPG_RAT, NDUV2_RAT, SDHA_RAT, LISI _RAT, PYC_RAT, ATP8_RAT, SFXNS_RAT
mitochondrion	47 of 220 genes, 21.4%	2845 of 36892 genes, 7.7%	2.75e-08	0.00%	0.00	COX41_RAT, KCRU _RAT, CDS2_RAT, VATI_RAT, BCATI_RAT, M20M_RAT, EFTU_RAT, QCR8_RAT, F213 A_RAT, NDU54_RAT, DHE3 _RAT, MPCP_RAT, NDUSI _RAT, ACADL_RAT, IDH GI _RAT, DLDH_RAT, ECHM _RAT, COX2_RAT, TIM 13_RAT, IVD_RAT, ATPA_RAT, CX7A2 _RAT, DECR_RAT, SODM_RAT, DHB4_RAT, NDUS2_RAT, MGSTI_RAT, GSTKI _RAT, HCD2_RAT, ECHB _RAT, ECHI_RAT, LPPRC_RAT, LETMI _RAT, TRAPI _RAT, KPCA_RAT, DHSO_RAT, VDAC2_RAT, NDUV2_RAT, GSTPI _RAT, ATPG_RAT, SDHA_RAT, DDAH2_RAT, ACSF2_RAT, NAKD2_RAT, PYC_RAT, ATP8_RAT, SFXNS_RAT
intracellular	167 of 220 genes, 75.9%	20625 of 36892 genes, 55.9%	1.03e-07	0.00%	0.00	COX41_RAT, KCRU _RAT, LANCI_RAT, BCATI_RAT, STIPI_RAT, VATEI_RAT, TCPG_RAT, 7B2_RAT, RL7 _RAT, IDHGI _RAT, EC HM _RAT, PRS7 _RAT, CLCB_RAT, RS9_RAT, PLPP_RAT, IVD_RAT, ATPA_RAT, NCSI _RAT, RS19 _RAT, D□XI _RAT, RS26_RAT, DLG4_RAT, PTN23_RAT, MATR3_RAT, AT1B3_RAT, EL OB_RAT, NSDHL_RAT, RL32_RAT, SEPT2_RAT, ELP2_RAT, TBA4A_RAT, SNAB_RAT, TPPP3_ RAT, EFIAI_RAT, NSF_RAT, SCFDI_RAT, KPCA _RAT, SH3GI_RAT, VDAC2_RAT, GDIRI _RAT, NDUV2_RAT,HS10 5_RAT, PSB2_RAT, FSCNI_RAT, NUCL_RAT, RCN2_RAT, CNDP 2_RAT, NFL_RAT, M 20 M_RAT, PSMD2 _RAT, PPP6_RAT, QCR8_RAT, F213A_RAT, RASAI _RAT, CATD_RAT, PITMI_RAT, CN37_ RAT, DLDH_RAT, TPMI _RAT, GNAO_RAT, TIM13 _RAT, KI NH _RAT, RT CB_RAT, DECR_RAT, SODM _RAT, RL23A_RAT, RL18 _RAT, MGSTI_RAT, CDC42_RAT, GSTKI_RAT, ECHB_RAT, RS8 _RAT, GSTA6_RAT, NAKD2 _RAT, LISI _RAT, CALX_RAT, SFXN5_RAT, NMTI _RAT, LDHA _RAT, RPNI_RAT, CDS2_RAT, PSB8_RAT, PAK2_RAT, RBBP7 _RAT, CSK2B_RAT, NDU54_RAT, MGLL_RAT, FRI LI _RAT, NUD T3_RAT, MPCP_RAT, SYN2_RAT, COX2_RAT, GSTM4_RAT, RPGF2_RAT, RLIOA_RAT, CSKP_RAT, LGMN _RAT, MYOSA_RAT, FTO_RAT, SNAA_RAT, KPYM _RAT, SYTI_RAT, CSRPI_RAT, DHB4_RAT, ROCK2_RAT, VAPA_RAT, U2 AF4_RAT, NDUS 2_RAT, HCD2_RAT, IDH 3B_RAT, LPPRC_RAT, RS3_RAT, TBCE_RAT, ILEUA_RAT, ULAI _RAT, GSTPI_RAT, ATPG_RAT, SDHA_RAT, DDAH2_RAT, ACSF2_RAT, PSA6_RAT, SCAMI_RAT, ATP8_RAT, GAK_RAT, RLAO_RAT, LTOR3 _RAT, CRYLI _RAT, PPID_RAT, MYHIO _RAT, VATI _RAT, ROGDI_RAT , MARE3_RAT, EFTU_RAT, PA CNI _RAT, NCOAT_RAT, PRS6A_RAT, DHE3_RAT, PLCLI _RAT, NDUSI _RAT, ACADL_RAT, SAEI _RAT, ANMI_RAT, RL9_RAT, PSMEI _RAT, RL8 _RAT, PLCGI_RAT, MYH9_RAT, CX7A2_RAT, VGF_RAT, RL13 _RAT, CDV3_RAT, PHOCN_RAT, RAB3C_RAT, CLCA_RAT, MTPN_RAT, DADI _RAT, RL15_RAT, LZTLI_RAT, DPYL4_RAT, RL13A_RAT, ECHI _RAT, TRAPI _RAT, LETMI _RAT, DHSO_RAT, PYC_RAT, BIN2_RAT, CITC_RAT
organelle membrane	54 of 220 genes, 24.5%	3728 of 36892 genes, 10.1%	1.08e-07	0.00%	0.00	COX41_RAT, LTOR3_RAT, KCRU_RAT, RPNI_RAT, CDS2_RAT, VATI_RAT, M20 M _RAT, PACNI_RAT, QCR8_RAT, NDU 54_RAT, DHE3 _RAT, MPCP_RAT, NDUSI _RAT, PITMI _RAT, ACADL_RAT, COX2_RAT, TIM13_RAT, CSKP_RAT, CLCB_RAT, IVD_RAT, ATPA_RAT, NCSI _RAT, CX7A2_RAT, SYTI _RAT, SODM_RAT, PHOCN_RAT, DHB4_RAT, CLCA_RAT, VAPA_RAT, NDUS2_RA T, NSDHL_RAT, DADI _RAT, MGSTI_RAT, SEPT2_RAT, CDC42 _RAT, GSTKI_RAT, EC HB_RAT, LPPRC_RAT, SCFDI_RAT, LETMI_RAT, TRAPI_RAT, KPCA_RAT, SH3GI_RAT, DHSO_RAT, VDAC2_RAT, NDUV2_RAT, ATPG_RAT, SDHA_RAT, LISI _RAT, CALX_RAT, PYC_RAT, SCAMI_RAT, ATP8_RAT, SFXN5_RAT
	168of 220 genes, 76.4%	21266 of 36892 genes, 57.6%	9.13e-07	0.00%	0.00	COX41_RAT, KCRU _RAT, LANCI_RAT, BCATI_RAT, STIPI _RAT, VATEI_RAT, TCPG_RAT, 7B2_RAT, RL7 _RAT, IDHGI _RAT, EC HM _RAT, PRS7 _RAT, CLCB_RAT, RS9_RAT, PLPP_RAT, IVD_RAT, ATPA_RAT, NCSI _RAT, RS19 _RAT, D□XI _RAT, RS26_RAT, DLG4_RAT, PTN23_RAT, MATR3_RAT, AT1B3_RAT, EL OB_RAT, NSDHL_RAT, RL32_RAT, SEPT2_RAT, ELP2_RAT, TBA4A_RAT, SNAB_RAT, TPPP3_ RAT, EFIAI_RAT, NSF_RAT, SCFDI_RAT, KPCA _RAT, SH3GI_RAT, VDAC2_RAT, GDIRI _RAT, NDUV2_RAT,HS10 5_RAT, PSB2_RAT, FSCNI_RAT, NUCL_RAT, RCN2_RAT, CNDP 2_RAT, NFL_RAT, M 20 M_RAT, PSMD2 _RAT, PPP6_RAT, QCR8_RAT, F213A_RAT, RASAI _RAT, CATD_RAT, PITMI_RAT, CN37_ RAT, DLDH_RAT, TPMI _RAT, TIM13 _RAT, GNAO_RAT, KI NH _RAT, RT CB_RAT, DECR_RAT, SODM_RAT, RL23A_RAT, RL18 _RAT, MGSTI_RAT, CDC42_RAT, GSTKI_RAT, ECHB_RAT, RS8 _RAT, GSTA6_RAT, NAKD2 _RAT, LISI _RAT, CALX_RAT, SFXN5_RAT, NMTI _RAT, LDHA _RAT, RPNI_RAT, CDS2_RAT, PSB8_RAT, PAK2_RAT, RBBP7 _RAT, CSK2B_RAT, NDU54_RAT, MGLL_RAT, FRI LI _RAT, NUD T3_RAT, MPCP_RAT, SYN2_RAT, COX2_RAT, GSTM4_RAT, RPGF2_RAT, RLIOA_RAT, CSKP_RAT, LGMN _RAT, MYOSA_RAT, FTO_RAT, DCEI _RAT, SNAA_RAT, KPYM _RAT, SYTI_RAT, CSRPI_RAT, DHB4_RAT, ROCK2_RAT, VAPA_RAT, U2AF4_RAT, NDUS2_RAT, HCD 2_RAT, IDH 3B_RAT, LPPRC_RAT, RS3_RAT, TBCE_RAT, ILEUA_RAT, ULAI _RAT, GSTPI_RAT, ATPG_RAT, SDHA_RAT, DDAH2_RAT, ACSF2_RAT, PSA6_ RAT, SCAMI_RAT, ATPS_RAT, GAK_RAT, RLAO_RAT, LTOR3 _RAT, CRYLI _RAT, PPID_RAT, MYHIO_RAT, VATI_RAT, ROGDI_RAT, MARE3_RAT, EFTU_RAT, PA CNI _RAT, NCOAT_RAT, PRS6A_RAT, DHE3 _RAT, PLCLI _RAT, NDUSI _RAT , ACADL_RAT, SAEI_RAT, ANMI_RAT, RL9_RAT, PSMEI_RAT, RL8 _RAT, PLCGI_RAT, MY H9 _RAT, CX7A2_RAT, VGF_RAT, RL13 _RAT, CDV3_RAT, PHOCN_RAT, RAB3C_RAT, CLCA_RAT, MTPN_RAT, DADI_RAT, RL15_RAT, LZTLI_RAT, DPYL4_RAT, RL13A_ RAT, ECHI_RAT, TRAPI _RAT, LETMI _RAT, DHSO_RAT, PYC _RAT, BIN2_RAT, CITC_RAT

	160of 220 genes, 72.7%	19810of 36892 genes, 53.7%	1.0le-06	0.00%	0.00	COX41_RAT, KCRU_RAT, LANCI_RAT, BCATI_RAT, STIPI_RAT, VATEI_RAT, 7B2_RAT, RL7_RAT, IDHGI_RAT, ECHM_RAT, PRS7_RAT, CLCB_RAT, PLPP_RAT, IVD_RAT, ATPA_RAT, NCSI_RAT, RS19_RAT, DDXI_RAT, RS26_RAT, DLG4_RAT, PTN23_RAT, MATR3_RAT, AT1B3_RAT, ELOB_RAT, NSDHL_RAT, RL32_RAT, SEPT2_RAT, ELP2_RAT, TBA4A_RAT, TPPP3_RAT, EFIAI_RAT, NSF_RAT, SCFDI_RAT, KPCA_RAT, SH3GI_RAT, VDAC2_RAT, GDIRI _RAT, NDUV2_RAT, HS105_RAT, PSB2_RAT, CD166_RAT, NUCL_RAT, RCN2_RAT, CNDP2_RAT, CYTC_RAT, NFL_RAT, NCALD_RAT, M20M_RAT, PSMD2_RAT, QCR8_RAT, F213A_RAT, CATD_RAT, PITMI_RAT, CN37_RAT, DLDH_RAT, TPMI_RAT, TIM13_RAT, KINH_RAT, RTCB_RAT, DECR_RAT, 54A4_RAT, SODM_RAT, RL23A_RAT, RL18_RAT, MGSTI_RAT, CDC42_RAT, GSTKI_RAT, ECHB_RAT, RSB_RAT, NAKD2_RAT, LISI_RAT, CALX_RAT, SFXNS_RAT, NMTI_RAT, RPNI_RAT, CDS2_RAT, PSB8_RAT, S15A2_RAT, PAK2_RAT, RBBP7_RAT, CSK2B_RAT, NDUS4_RAT, MGLL_RAT, NUDT3_RAT, MPCP_RAT, SYN2_RAT, FIOAI_RAT, COX2_RAT, RPGF2_RAT, GSTM4_RAT, RLIOA_RAT, CSKP_RAT, GLYG_RAT, LGMN_RAT, MY05A_RAT, FTO_RAT, SNAA_RAT, KPYM_RAT, SYTI_RAT, CSRPI_RAT, BPNTI_RAT, DHB4_RAT, ROCK2_RAT, VAPA_RAT, U2AF4_RAT, NDUS2_RAT, LG3BP_RAT, HCD2_RAT, IDH3B_RAT, LPPRC_RAT, RS3_RAT, TBCE_RAT, ULAI_RAT, GSTPI_RAT, ATPG_RAT, SDHA_RAT, DDAH2_RAT, ACSF2_RAT, PSA6_RAT, SCAMI_RAT, ATPB_RAT, GAK_RAT, RLAO_RAT, LTOR3_RAT, CRYLI_RAT, PPID_RAT, MYHIO_RAT, VATI_RAT, ROGDI_RAT, MARE3_RAT, EFTU_RAT, PACNI_RAT, NCOAT_RAT, PRS6A_RAT, DHE3_RAT, ATIBI_RAT, NDUSI_RAT, ACADL_RAT, ANMI_RAT, SAEI_RAT, RL9_RAT, RL8_RAT, PLCGI_RAT, MYH9_RAT, CX7A2_RAT, VGF_RAT, RL13_RAT, PHOCN_RAT, RAB3C_RAT, CLCA_RAT, MTPN_RAT, DADI_RAT, RL15_RAT, RL13A_RAT, ECHI_RAT, TRAPI_RAT, LETMI_RAT, DHSO_RAT, PYC_RAT, BIN2_RAT
membrane-bounded vesicle	64 of 220 genes, 29.1%	5298 of 36892 genes, 14.4%	2.65e-06	0.00%	0.00	COX41_RAT, LANCI_RAT, RPNI_RAT, PSB8_RAT, S15A2_RAT, CSK2B_RAT, NUDT3_RAT, SYN2_RAT, FIOAI_RAT, 7B2_RAT, GSTM4_RAT, CLCB_RAT, GLYG_RAT, MY05A_RAT, PLPP_RAT, NCSI_RAT, SNAA_RAT, SYTI_RAT, RS26_RAT, PTN23_RAT, DLG4_RAT, CSRPI_RAT, BPNTI_RAT, AT1B3_RAT, LG3BP_RAT, SEPT2_RAT, TBA4A_RAT, TPPP3_RAT, GDIRI_RAT, DDAH2_RAT, HS105_RAT, PSA6_RAT, SCAMI_RAT, CD166_RAT, PSB2_RAT, LTOR3_RAT, CYTC_RAT, CNDP2_RAT, CRYLI_RAT, VATI_RAT, NCALD_RAT, EFTU_RAT, PSMD2_RAT, F213A_RAT, PACNI_RAT, ATIBI_RAT, CATD_RAT, CN37_RAT, KI NH _RAT, VGF_RAT, SODM_RAT, 54A4_RAT, RAB3C_RAT, CLCA_RAT, MTPN_RAT, DADI_RAT, GSTKI_RAT, CDC42_RAT, ECHB_RAT, ECHI_RAT, TRAPI_RAT, RSB_RAT, LISI_RAT, CALX_RAT
vesicle	64 of 220 genes, 29.1%	5460 of 36892 genes, 14.8%	8.44e-06	0.00%	0.00	COX41_RAT, LANCI_RAT, RPNI_RAT, PSB8_RAT, S15A2_RAT, CSK2B_RAT, NUDT3_RAT, SYN2_RAT, FIOAI_RAT, 7B2_RAT, GSTM4_RAT, CLCB_RAT, GLYG_RAT, MY05A_RAT, PLPP_RAT, NCSI_RAT, SNAA_RAT, SYTI_RAT, RS26_RAT, PTN23_RAT, DLG4_RAT, CSRPI_RAT, BPNTI_RAT, AT1B3_RAT, LG3BP_RAT, SEPT2_RAT, TBA4A_RAT, TPPP3_RAT, GDIRI_RAT, DDAH2_RAT, HS105_RAT, PSA6_RAT, SCAMI_RAT, CD166_RAT, PSB2_RAT, LTOR3_RAT, CYTC_RAT, CNDP2_RAT, CRYLI_RAT, VATI_RAT, NCALD_RAT, EFTU_RAT, PSMD2_RAT, F213A_RAT, PACNI_RAT, ATIBI_RAT, CATD_RAT, CN37_RAT, KI NH _RAT, VGF_RAT, SODM_RAT, 54A4_RAT, RAB3C_RAT, CLCA_RAT, MTPN_RAT, DADI_RAT, GSTKI_RAT, CDC42_RAT, ECHB_RAT, ECHI_RAT, TRAPI_RAT, RSB_RAT, LISI_RAT, CALX_RAT
in tracellular organe lle par t	93 of 220 genes, 42.3%	9439 of 36892 genes, 25.6%	1.00e-05	0.00%	0.00	COX41_RAT, KCRU_RAT, RPNI_RAT, CDS2_RAT, RBBP7_RAT, NDUS4_RAT, CSK2B_RAT, MPCP_RAT, ECHM_RAT, COX2_RAT, RLIOA_RAT, CLCB_RAT, FTO_RAT, IVD_RAT, ATPA_RAT, NCSI_RAT, SYTI_RAT, RS19_RAT, DDXI_RAT, RS26_RAT, PTN23_RAT, DLG4_RAT, ROCK2_RAT, DHB4_RAT, MATR3_RAT, VAPA_RAT, U2AF4_RAT, NDUS2_RAT, NSDHL_RAT, RL32_RAT, SEPT2_RAT, ELP2_RAT, TBA4A_RAT, LPPRC_RAT, TPPP3_RAT, EFIAI_RAT, NSF_RAT, SCFDI_RAT, KPCA_RAT, SH3GI_RAT, VDAC2_RAT, NDUV2_RAT, ATPG_RAT, SDHA_RAT, HS105_RAT, PSA6_RAT, SCAMI_RAT, ATP8_RAT, NUCL_RAT, RCN2_RAT, LTOR3_RAT, PPID_RAT, NFL_RAT, MYHIO_RAT, VATI_RAT, MARE3_RAT, M20M_RAT, ROGDI_RAT, EFTU_RAT, PACNI_RAT, QCRB_RAT, DHE3_RAT, NDUSI_RAT, ACADL_RAT, PITMI_RAT, ANMI_RAT, CN37_RAT, DLDH_RAT, TIM 13_RAT, RL8_RAT, KINH_RAT, MYH9_RAT, PLCGI_RAT, CX7A2_RAT, RL13_RAT, SODM_RAT, PHOCN_RAT, CLCA_RAT, MTPN_RAT, DADI_RAT, MGSTI_RAT, CDC42_RAT, GSTKI_RAT, RL13A_RAT, ECHB_RAT, LETMI_RAT, TRAPI_RAT, DHSO_RAT, LISI_RAT, CALX_RAT, PYC_RAT, BIN2_RAT, SFXN5_RAT
organelle par t	94 of 220 genes, 42.7%	9756 of 36892 genes, 26.4%	2.46e-05	0.00%	0.00	COX41_RAT, KCRU_RAT, RPNI_RAT, CDS2_RAT, RBBP7_RAT, NDUS4_RAT, CSK2B_RAT, MPCP_RAT, ECHM_RAT, COX2_RAT, CSKP_RAT, RLIOA_RAT, CLCB_RAT, FTO_RAT, IVD_RAT, ATPA_RAT, NCSI_RAT, SYTI_RAT, RS19_RAT, DDXI_RAT, RS26_RAT, PTN23_RAT, DLG4_RAT, ROCK2_RAT, DHB4_RAT, MATR3_RAT, VAPA_RAT, U2AF4_RAT, NDUS2_RAT, NSDHL_RAT, RL32_RAT, SEPT2_RAT, ELP2_RAT, TBA4A_RAT, LPPRC_RAT, TPPP3_RAT, EFIAI_RAT, NSF_RAT, SCFDI_RAT, KPCA_RAT, SH3GI_RAT, VDAC2_RAT, NDUV2_RAT, ATPG_RAT, SDHA_RAT, HSIOS_RAT, PSA6_RAT, SCAMI_RAT, ATPB_RAT, NUCL_RAT, RCN2_RAT, LTOR3_RAT, PPID_RAT, NFL_RAT, MYHIO_RAT, VATI_RAT, MARE3_RAT, M20M_RAT, ROGDI_RAT, EFTU_RAT, PACNI_RAT, QCRB_RAT, DHE3_RAT, NDUSI_RAT, ACADL_RAT, PITMI_RAT, ANMI_RAT, CN37_RAT, DLDH_RAT, TIM13_RAT, RLB_RAT, KINH_RAT, MYH9_RAT, PLCGI_RAT, CX7A2_RAT, RL13_RAT, SODM_RAT, PHOCN_RAT, CLCA_RAT, MTPN_RAT, DADI_RAT, MGSTI_RAT, CDC42_RAT, GSTKI_RAT, RL13A_RAT, ECHB_RAT, LETMI_RAT, TRAPI_RAT, DHSO_RAT, LISI_RAT, CALX_RAT, PYC_RAT, BIN2_RAT, SFXN5_RAT
intrace llular organelle	144of220 genes, 65.5%	17906 of 36892 genes, 48.5%	5.99e-05	0.00%	0.00	COX41_RAT, KCRU_RAT, LANCI_RAT, BCATI_RAT, STIPI _RAT, VATEI_RAT, 7B2_RAT, RL7_RAT, IDHGI_RAT, ECHM_RAT, PRS7_RAT, CLCB_RAT, PLPP_RAT, IVD_RAT, ATPA_RAT, NCSI_RAT, RS19_RAT, DDXI_RAT, RS26_RAT, DLG4_RAT, PTN23_RAT, MATR3_RAT, AT1B3_RAT, ELOB_RAT, NSDHL_RAT, RL32_RAT, SEPT2_RAT, ELP2_RAT, TBA4A_RAT, TPPP3_RAT, EFIAI_RAT, NSF_RAT, SCFDI_RAT, KPCA_RAT, SH3GI_RAT, VDAC2_RAT, NDUV2_RAT, HSIOS_RAT, PSB2_RAT, NUCL_RAT, RCN2_RAT, NFL_RAT, M20M_RAT, QCR8_RAT, F213A_RAT, CATD_RAT, PITMI_RAT, CN37_RAT, DLDH_RAT, TPMI_RAT, TIM13_RAT, KINH_RAT, RTCB_RAT, DECR_RAT, SODM_RAT, RL23A_RAT, RL18_RAT, MGSTI_RAT, CDC42_RAT, GSTKI_RAT, ECHB_RAT, RSB_RAT, NAKD2_RAT, LISI_RAT, CALX_RAT, SFXN5_RAT, NMTI_RAT, RPNI_RAT, CDS2_RAT, PSB8_RAT, PAK2_RAT, RBBP7_RAT, CSK2B_RAT, NDU54_RAT, MGLL_RAT, MPCP_RAT, SYN2_RAT, COX2_RAT, GSTM4_RAT, RPGF2_RAT, RLIOA_RAT, CSKP_RAT, LGMN_RAT, MY05A_RAT, FTO_RAT, KPYM_RAT, SYTI_RAT, CSRPI_RAT, DHB4_RAT, ROCK2_RAT, VAPA_RAT, U2AF4_RAT, NDUS2_RAT, HCD2_RAT, IDH3B_RAT, LPPRC_RAT, RS3_RAT, TBCE_RAT, ULAI_RAT, GSTPI_RAT, ATPG_RAT, SDHA_RAT, ACSF2_RAT, DDAH2_RAT, PSA6_RAT, SCAMI_RAT, ATP8_RAT, GAK_RAT, RLAO_RAT, LTOR3_RAT, PPID_RAT, MYHIO_RAT, VATI_RAT, ROGDI_RAT, MARE3_RAT, EFTU_RAT, PACNI_RAT, NCOAT_RAT, PRS6A_RAT, DHE3_RAT, NDUSI_RAT, ACADL_RAT, ANMI_RAT, SAEI_RAT, RL9_RAT, RLB_RAT, PLCGI_RAT, MYH9_RAT, CX7A2_RAT, VGF_RAT, RL13_RAT, PHOCN_RAT, RAB3C_RAT, CLCA_RAT, MTPN_RAT, DADI_RAT, RLIS_RAT, RL13A_RAT, ECHI_RAT, TRAPI_RAT, LETMI_RAT, DHSO_RAT, PYC_RAT, BIN2_RAT
membrane-bounded organelle	141of220 genes, 64.1%	17552 of 36892 genes, 47.6%	0.00011	0.00%	0.00	COX41_RAT, KCRU_RAT, LANCI_RAT, BCATI_RAT, STIPI_RAT, VATEI_RAT, 7B2_RAT, IDHGI_RAT, ECHM_RAT, CLCB_RAT, PLPP_RAT, IVD_RAT, ATPA_RAT, NCSI_RAT, RS19_RAT, DDXI_RAT, RS26_RAT, DLG4_RAT, PTN23_RAT, MATR3_RAT, AT1B3_RAT, ELOB_RAT, NSDHL_RAT, SEPT2_RAT, ELP2_RAT, TBA4A_RAT, TPPP3_RAT, EFIAI_RAT, SCFDI_RAT, KPCA_RAT, SH3GI_RAT, VDAC2_RAT, GDIRI_RAT, NDUV2_RAT, HSIOS_RAT, PSB2_RAT, CD166_RAT, NUCL_RAT, RCN2_RAT, CNDP2_RAT, CYTC_RAT, NCALD_RAT, M20M_RAT, PSMD2_RAT, QCRB_RAT, F213A_RAT, CATD_RAT, PITMI_RAT, CN37_RAT, DLDH_RAT, TIM13_RAT, KINH_RAT, RTCB_RAT, DECR_RAT, S4A4_RAT, SODM_RAT, RL23A_RAT, MGSTI_RAT, CDC42_RAT, GSTKI_RAT, ECHB_RAT, RSB_RAT, NAKD2_RAT, LISI_RAT, CALX_RAT, SFXN5_RAT, RPNI_RAT, CDS2_RAT, PSB8_RAT, S15A2_RAT, PAK2_RAT, RBBP7_RAT, CSK2B_RAT, NDU54_RAT, NUDT3_RAT, MPCP_RAT, SYN2_RAT, FIOAI_RAT, COX2_RAT, GSTM4_RAT, RPGF2_RAT, CSKP_RAT, GLYG_RAT, LGMN_RAT, MYOSA_RAT, FTO_RAT, SNAA_RAT, KPYM_RAT, SYTI_RAT, CSRPI_RAT, BPNTI_RAT, DHB4_RAT, ROCK2_RAT, VAPA_RAT, U2AF4_RAT, NDUS2_RAT, LG3BP_RAT, HCD2_RAT, IDH3B_RAT, LPPRC_RAT, RS3_RAT, ULAI_RAT, GSTPI_RAT, ATPG_RAT, SDHA_RAT, ACSF2_RAT, DDAH2_RAT, PSA6_RAT, SCAMI_RAT, ATPB_RAT, GAK_RAT, RLAO_RAT, LTOR3_RAT, PPID_RAT, CRYLI_RAT, VATI_RAT, ROGDI_RAT, EFTU_RAT, PACNI_RAT, NCOAT_RAT, PRS6A_RAT, DHE3_RAT, ATIBI_RAT, NDUSI_RAT, ACADL_RAT, ANMI_RAT, SAEI_RAT, PLCGI_RAT, MYH9_RAT, CX7A2_RAT, VGF_RAT, PHOCN_RAT, RAB3C_RAT, CLCA_RAT, MTPN_RAT, DADI_RAT, ECHI_RAT, TRAPI_RAT, LETMI_RAT, DHSO_RAT, PYC_RAT
proteasome complex	8of220 genes,3. 6%	142 of 36892 genes, 0.4%	0.00049	0.00%	0.00	PSMD2_RAT, PRS6A_RAT, ELP2_RAT, PSA6_RAT, PSB8_RAT, PRS7_RAT, PSB2_RAT, PSMEI_RAT
extracellular vesicular exosome	49 of 220 genes, 22.3%	4233 of 36892 genes, 11.5%	0.00077	0.09%	0.02	COX41_RAT, LTOR3_RAT, LANCI_RAT, CRYLI_RAT, CNDP2_RAT, CYTC_RAT, PSBB_RAT, S15A2_RAT, NCALD_RAT, VATI_RAT, PSMD2_RAT, EFTU_RAT, F213A_RAT, CSK2B_RAT, NUDT3_RAT, ATIBI_RAT, FIOAI_RAT, CN37_RAT, GSTM4_RAT, GLYG_RAT, PLPP_RAT, NCSI_RAT, SNAA_RAT, 54A4_RAT, SODM_RAT, CSRPI_RAT, RS26_RAT, BPNTI_RAT, AT1B3_RAT, MTPN_RAT, LG3BP_RAT, DADI_RAT, SEPT2_RAT, CDC42_RAT, GSTKI_RAT, TBA4A_RAT, ECHB_RAT, ECHI_RAT, TPPP3_RAT, TRAPI_RAT, RSB_RAT, GDIRI_RAT, DDAH2_RAT, HSIOS_RAT, LISI_RAT, CALX_RAT, PSA6_RAT, PSB2_RAT, CD166_RAT
extracellular organelle	49 of 220 genes, 22.3%	4238 of 36892 genes, 11.5%	0.00079	0.09%	0.02	COX41_RAT, LTOR3_RAT, LANCI_RAT, CRYLI_RAT, CNDP2_RAT, CYTC_RAT, PSBB_RAT, S15A2_RAT, NCALD_RAT, VATI_RAT, PSMD2_RAT, EFTU_RAT, F213A_RAT, CSK2B_RAT, NUDT3_RAT, ATIBI_RAT, FIOAI_RAT, CN37_RAT, GSTM4_RAT, GLYG_RAT, PLPP_RAT, NCSI_RAT, SNAA_RAT, 54A4_RAT, SODM_RAT, CSRPI_RAT, RS26_RAT, BPNTI_RAT, AT1B3_RAT, MTPN_RAT, LG3BP_RAT, DADI_RAT, SEPT2_RAT, CDC42_RAT, GSTKI_RAT, TBA4A_RAT, ECHB_RAT, ECHI_RAT, TPPP3_RAT, TRAPI_RAT, RSB_RAT, GDIRI_RAT, DDAH2_RAT, HSIOS_RAT, LISI_RAT, CALX_RAT, PSA6_RAT, PSB2_RAT, CD166_RAT

extracellular membrane-bounded organelle	49 of 220 genes, 22.3%	4238 of 36892 genes, 11.5%	0.00079	0.08%	0.02	COX41_RAT, LTOR3_RAT, LANCI_RAT, CRYLI_RAT, CNDP 2_RAT, CYTC_RAT, PSB8 _RAT, S15A2_RAT, NCA LD_RAT, VATI _RAT, PSM D2_RAT, EFTU_RAT, F213A_RAT, CSK2B_RAT, NUDT3 _RAT, ATIBI_RAT, FIOAI_RAT, CN37_RAT, GSTM4_RAT, GLYG_RAT, PLPP_RAT, NCSI _RAT, SNAA_RAT, S4A4_RAT, SODM_RAT, CSRPI_RAT, RS26_RAT, BPNTI_RAT, AT1 B3_ RAT, MTPN_RAT, LG3BP_RAT, DADI _RAT, SEPT2_RAT, CDC42 _RAT, GSTKI_RAT, TBA4A_RAT, ECHB _RAT, ECHI_RAT, TPPP3_RAT, TRAPI_RAT, RS8 _RAT, GDIRI_RAT, DDAH2_RAT, HSI OS_RAT, LISI _RAT, CALX._RAT, PSA6_RAT, PSB2_RAT, CD166_RAT
respiratory chain	8of220 enes, 3. 6% g	155 of 36892 enes, 0. 4%	0.00094	0.08%	0.02	NDUSI _RAT, COX2_RAT, NDU S2_RAT, NDUV2_RAT,SDHA_RAT, QCR8_RAT, NDU S4_RAT, CX7A 2_RAT
intracellular membrane-bounded organelle	122of220 genes, 55.5%	15481 of 36892 genes, 42.0%	0.00752	0.08%	0.02	COX41_RAT, KCRU _RAT, LANCI_RAT, RPNI_RAT, CDS2_RAT, PSB8_RAT, PAK2_RAT, BCATI_RAT, RBBP7 _RAT, STIPI_RAT, NDUS4_RAT , CSK2B_RAT, VATEI _RAT, SYN2_RAT, MPCP_RAT, 7B2_RAT, IDH GI _RAT, ECHM _RAT, COX2_RAT, RPGF2_RAT, GSTM4_RAT, CSKP_RAT, CLCB_RAT, LGMN _RAT, MY05A_RAT, FTO_RAT, IVD_RAT, ATPA_RAT, NCSI _RAT, KPYM_RAT, SYTI_RAT, RS19 _RAT, DDXI _RAT, RS26_RAT, CSRPI _RAT, DLG4_RAT, PTN23_RAT, ROCK2_RAT, DHB4_RAT, MATR3_RAT, AT1 B3_ RAT, ELOB_RAT, VAPA_RAT, U2AF4 _RAT, NDUS2_RAT, NSDHL_RAT, SEPT2_RAT, EL P2_RAT, HCD2_RAT , IDH3B _RAT, LPPRC_RAT, EFIAI_RAT, SCFDI_RAT, KPCA_RAT, RS3_RAT, SH3GI_RAT, VDAC2_RAT, ULAI _RAT, ATPG_RAT, GSTPI_RAT, NDUV2_RAT, SDHA_RAT, DDAH2_RAT, HS105 _RAT, ACSF2_RAT, PSA6_RAT, SCAMI_RAT, GAK_RAT, PSB2 _RAT, ATP8_ RAT, NU CL_RAT, RLAO_RAT , RCN 2_RAT, LTOR3 _RAT, PPID_RAT, VATI _RAT, ROGDI_RAT, M20M_RAT, EFTU_RAT, PACNI_RAT, NCOAT_RAT, QCR8_RAT, F213A_RAT, PRS6A_RAT, DHE3 _RAT, CATD _RAT, NDUSI _RAT, PITMI_RAT, ACADL_RAT, SAEI_RAT, ANMI _RAT, CN37 _RAT, DLDH_RAT, TIM13 _RAT, KINH_RAT, RT CB_RAT, MYH9 _RAT, PLCGI _RAT, VGF_RAT, CX7A2_RAT, DECR_RAT, SODM_RAT, PHOCN_RAT, RAB3C_RAT, CLCA_RAT, RL23A_RAT, MTPN_RAT, DADI _RAT, MGSTI_RAT, CDC42 _RAT , GSTKI_RAT, ECHB _RAT, ECHI_RAT, LETMI_RAT, TRAPI_RAT, DHSO_RAT, RS8 _RAT, NAKD2_RAT, LISI _RAT, CALX._RAT, PYC_RAT, SFXNS_RAT
